# Aligning climate and air quality goals demands early VOC control for China’s ozone pollution

**DOI:** 10.1038/s41467-026-75387-w

**Published:** 2026-07-09

**Authors:** Yujie Zhang, Jian Gao, Rui Gao, Xiaohui Du, Chao Yu, Xinlu Zhang, Yuqiang Zhang, Yuhong Liu, Yujing Mu, Liang Wen, Guannan Geng, Abdelwahid Mellouki, Fahe Chai, Hartmut Herrmann, Yujiao Zhu, Fang Bi, Yan Yang, Shuai Wang, Qian Wang, Jinpu Zhang, Hong Li, Hongli Wang, Jinhua Tao, Yixin Han, Mei He, Shijie Liu, Zhaoqi Gao, Bin Luo, Haisheng Li, Likun Xue

**Affiliations:** 1 State Key Laboratory of Environmental Criteria and Risk Assessment, Chinese Research Academy of Environmental Sciences, Beijing, China; 2 State Key Laboratory of Remote Sensing Science and Digital Earth, Aerospace Information Research Institute, Chinese Academy of Sciences, Beijing, China; 3 Environment Research Institute, Shandong University, Qingdao, China; 4 Research Center for Eco-Environmental Sciences, Chinese Academy of Sciences, Beijing, China; 5 State Key Laboratory of Regional Environment and Sustainability, School of Environment, Tsinghua University, Beijing, China; 6 University Mohammed VI Polytechnic (UM6P), Ben Guerir, Rehamna, Morocco; 7 Atmospheric Chemistry Department (ACD), Leibniz Institute for Tropospheric Research (TROPOS), Permoserstraße 15, Leipzig, Germany; 8 China National Environmental Monitoring Centre, Beijing, China; 9 State Environmental Protection Key Laboratory of Formation and Prevention of Urban Air Pollution Complex, Shanghai Academy of Environmental Sciences, Shanghai, China; 10 Guangzhou Sub-branch of Guangdong Ecological and Environmental Monitoring Center, Guangzhou, China; 11 State Key Laboratory of Organic Geochemistry, Guangdong Key Laboratory of Environmental Protection and Resources Utilization, Guangzhou Institute of Geochemistry, Chinese Academy of Sciences, Guangzhou, China; 12 Hubei Key Laboratory of Petroleum Geochemistry and Environment, Yangtze University, Wuhan, China

**Keywords:** Atmospheric chemistry, Atmospheric science

## Abstract

Global surface ozone (O_3_), intensified by climate change, poses increasing health and ecosystem threats. Despite stringent air policies, China’s persistent O_3_ pollution exemplifies a global challenge intertwined with climate actions reshaping emission pathways. Optimal mitigation remains contentious, primarily due to inconsistent conclusions regarding the sensitivity of summer regional O_3_ formation. Here we show the path dependency in O_3_ mitigation strategies for synergistic clean air and climate action goal achievement over multidecade scales. This path dependence is validated by observed concurrent plateaus (2020-2023) in deweathered O_3_ concentrations and sensitivity trends across Chinese megacity clusters. Leveraging this understanding of path dependency, we quantitatively reveal that the optimal future strategy involves prioritizing early volatile organic compound reductions, as their high effectiveness for regional O_3_ mitigation gradually diminishes towards 2050. This challenges prevailing nitrogen oxides priority paradigms. Our work reframes O_3_ control, providing a paradigm for resilient air quality-climate governance.

## Abstract (web summary)

This study identifies a time-limited “AVOC bonus window” for China’s ozone control. Uncovering photochemical path dependency shifts the paradigm from static sensitivity to dynamic optimization, aligning climate and air quality goals.

## Introduction

Surface ozone (O_3_) pollution, a pervasive and intensifying global environmental crisis, poses threats to human and ecosystem health^1^. This crisis is further exacerbated by and closely coupled with climate change^1–3^. China experiences frequent O_3_ level exceedance and has experienced one of the most rapid urban O_3_ level increase trends globally, with its national average warm-season maximum daily 8 h average (MDA8) O_3_ concentration increasing by 2.6 μg m^−3^ yr^−1^ between 2014 and 2020^4^. Paradoxically, this persistent O_3_ surge has occurred even as China’s stringent clean air policies, implemented since 2013, have successfully reduced nitrogen oxides (NO*x*) emissions^5–7^. This outcome contrasts with the general experiences in the United States, where NO*x-*targeted controls have proven effective for O_3_ mitigation^3,8,9^. Further complicating this picture, China’s ambitious climate actions (carbon neutrality goals) will trigger long-term, and asynchronous transformations in anthropogenic emission structures over the coming decades, with the projected NO*x* emission reductions significantly outweighing those of anthropogenic volatile organic compounds (AVOCs)^10^. This combined confluence of clean air policies and climate actions, which drive rapid and uncertain emission changes, presents a substantial challenge in the formulation of effective O_3_ mitigation strategies.

Traditionally, formulating O_3_ control strategies has largely depended on identifying O_3_ formation sensitivity to its precursors, specifically by delineating into VOC-limited, Transitional or NO*x*-limited regimes (termed sensitivity zoning)^11,12^. However, its application in China has yielded inconsistent conclusions of summer regional O_3_ sensitivity across ground, satellite, and model-based studies^13–18^, a discrepancy stemming not only from methodological limitations^12,16^, but, more fundamentally, from an inherent flaw in the traditional sensitivity zoning (Supplementary Note 1). These approaches rely on steady-state and short-timescale assumptions, rendering them incapable of capturing signals from the uncertain multidecadal dynamic evolution of precursor emissions driven by clean air and climate policies. This inability, in turn, hinders the formulation of optimal long-term O_3_ control strategies responsive to unprecedented emission dynamics^19–21^. This impasse leads directly to an unresolved scientific question: How can we move beyond the constraints of static sensitivity analysis to evaluate and identify the optimal O_3_ control pathway within the long-term, dynamic context of climate and air quality co-management in a timely manner?

Here, we address this challenge through the lens of path dependency: early choices in precursor emission controls shape the multi-decadal trajectory of O_3_ photochemistry (deweathered concentration and sensitivity), thereby dictating the synergistic achievement of both air quality and climate goals. To capture this critical dynamic, we develop and apply a Coupled Dynamic Trend Response (CDTR) analytical framework (Methods). This approach moves beyond ambiguous sensitivity zones by focusing on the robust, coupled trends of deweathered O_3_ concentrations and key sensitivity indicators (e.g., satellite-derived formaldehyde-to NO_2_ ratios, FNR) over multidecade timescales. It thus provides a tool to dynamically evaluate how the O_3_ photochemical system responds to different emission control pathways.

In this work, we first leverage the policy-driven emission changes in China over a two-decade period (2005–2024) to probe the evolution of summer regional O_3_ photochemistry. This field observation reveals a synchronous plateau in both regional O_3_ deweathered concentrations and their sensitivity trends from 2020 to 2023, providing empirical validation for our CDTR framework’s ability to capture dynamic signals inaccessible to traditional sensitivity zoning methods. Analysis of underlying drivers of the O_3_ photochemistry trajectories reveals real-world evidence of path dependency, indicating that prioritizing reductions in AVOCs is now the more effective strategy for mitigating summer O_3_ in China’s three megacity clusters (North China Plain (NCP), Yangtze River Delta (YRD), and Pearl River Delta (PRD); Supplementary Fig. 7). Building on this observation-derived directive, we apply the CDTR framework to quantitatively project optimal future O_3_ mitigation strategies in the context of China’s climate actions and clean air policies. These projections quantitatively identify a critical, time-limited AVOC reduction bonus window, establishing that an early and strengthened AVOC control strategy is imperative. These findings challenge the conventional strategy of rapidly transitioning O_3_ chemistry to a NO*x*-limited regime. This study provides a trend-based perspective on long-term O_3_ control strategies in a climate-changing world by highlighting the role of path dependency and establishes an analytical framework for designing adaptive air quality-climate co-governance.

## Results

### Synchronized plateau signal O_3_ path dependency

**Fig. 1:**
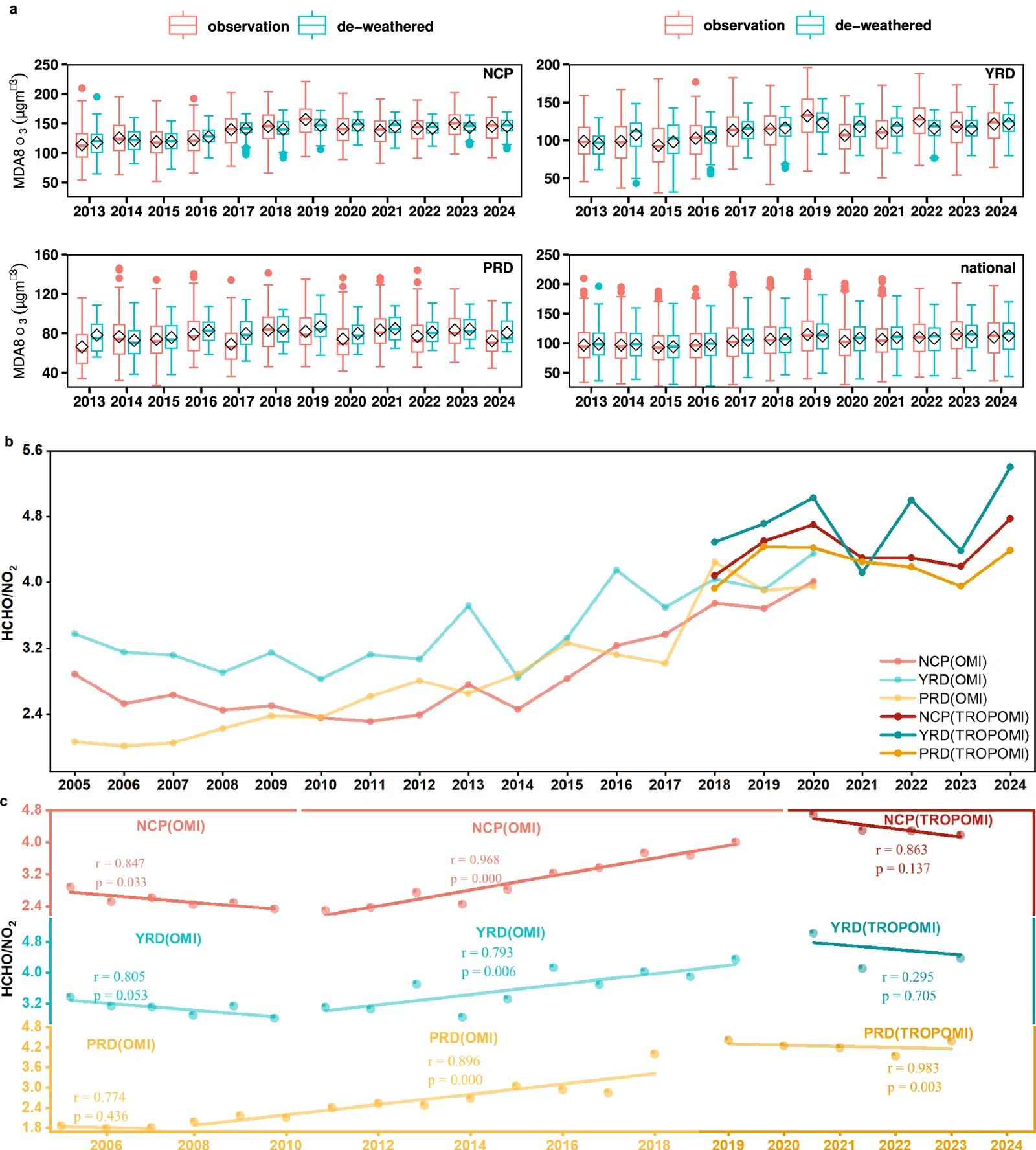
Observed historical trends in summer regional maximum daily 8 h average (MDA8) O_3_ concentrations and sensitivity until 2024. **a** Interannual variations of observed (orange) and deweathered (green) summer MDA8 O_3_ concentrations from 2013 to 2024 over the North China Plain (NCP), Yangtze River Delta (YRD), Pearl River Delta (PRD), and China. In the box plots, the center line, box limits, and whiskers represent the median, 25th (75th) percentiles, and 1st (99th) percentiles, respectively; rhombuses denote mean values and dots represent outliers. **b** Interannual variations of summer satellite-derived formaldehyde-to-NO_2_ ratios (FNR, HCHO/NO_2_) across the three megacity clusters (2005–2024). TROPOMI data are presented from 2018 onwards to account for OMI sensor degradation. **c** Linear fits of FNR across the three megacity clusters, segmented by two trend change points.

Figure 1 presents a coupled analysis of long-term trends in observed summer MDA8 O_3_ concentrations (2013–2024) and satellite-retrieved O_3_ sensitivity indicators (FNR, 2005–2024) across China’s three major megacity clusters. A phenomenon emerged from 2020–2023: the preceding nearly decade-long trends of rising deweathered MDA8 O_3_ concentrations (Supplementary Note 2) and shifting sensitivity towards the NO*x*-limited regime (indicated by increasing FNR) simultaneously entered a plateau or stagnation phase. This represents an interruption to the rise in deweathered MDA8 O_3_ concentrations in China since nationwide O_3_ observations began in 2013 (corroborated by independent research^13^). Concurrently, the FNR values ceased to rise or even slightly declined (Fig. 1b, c), indicating the impedance of the sensitivity trend in the progression towards the NO*x*-limited regime. This stagnation is further supported by interannual variations in the area fractions of different sensitivity regimes classified by both uniform and region-specific thresholds (Supplementary Figs. 1–3 and Supplementary Tables 1–6), as evidenced by a notable decrease in the percentage of NO*x*-limited areas approximately 2020. This field observation of a co-occurring plateau in the evolutionary trends of the deweathered O_3_ concentration and sensitivity indicators provides real-world validation of the feasibility of our proposed CDTR analytical framework for capturing dynamic O_3_ photochemical system behavior signals. The PRD’s O_3_ sensitivity trend change points occurred earlier than in the other two megacity clusters. We primarily attribute this to the more synchronized implementation of precursor control policies in the geographically adjacent NCP and YRD, which led to a more consistent evolution of their O_3_ sensitivity trends. This explanation is corroborated by the regional emission inventories. Specifically, the PRD experienced a significant decline in summer NO_2_ emissions post-2008, whereas a similar declining trend in the other two regions emerged after 2011. In contrast, AVOC emissions generally increased across all regions until 2017. Therefore, the pronounced post-2008 reduction in NO_2_ emissions was the principal driver for the turning point observed in the PRD’s O_3_ sensitivity trend around 2007. Owing to the well-documented issue of the Ozone Monitoring Instrument (OMI) satellite sensors aging^22^, characterized by pronounced striping noise in HCHO retrievals (Supplementary Fig. 6), Tropospheric Monitoring Instrument (TROPOMI) data have been adopted for the period since 2018. And the sensitivity trends derived from TROPOMI and OMI (detailed in Supplementary Note 3) were approximately consistent (Fig. 1 and Supplementary Tables 1, 3, 5).

**Fig. 2:**
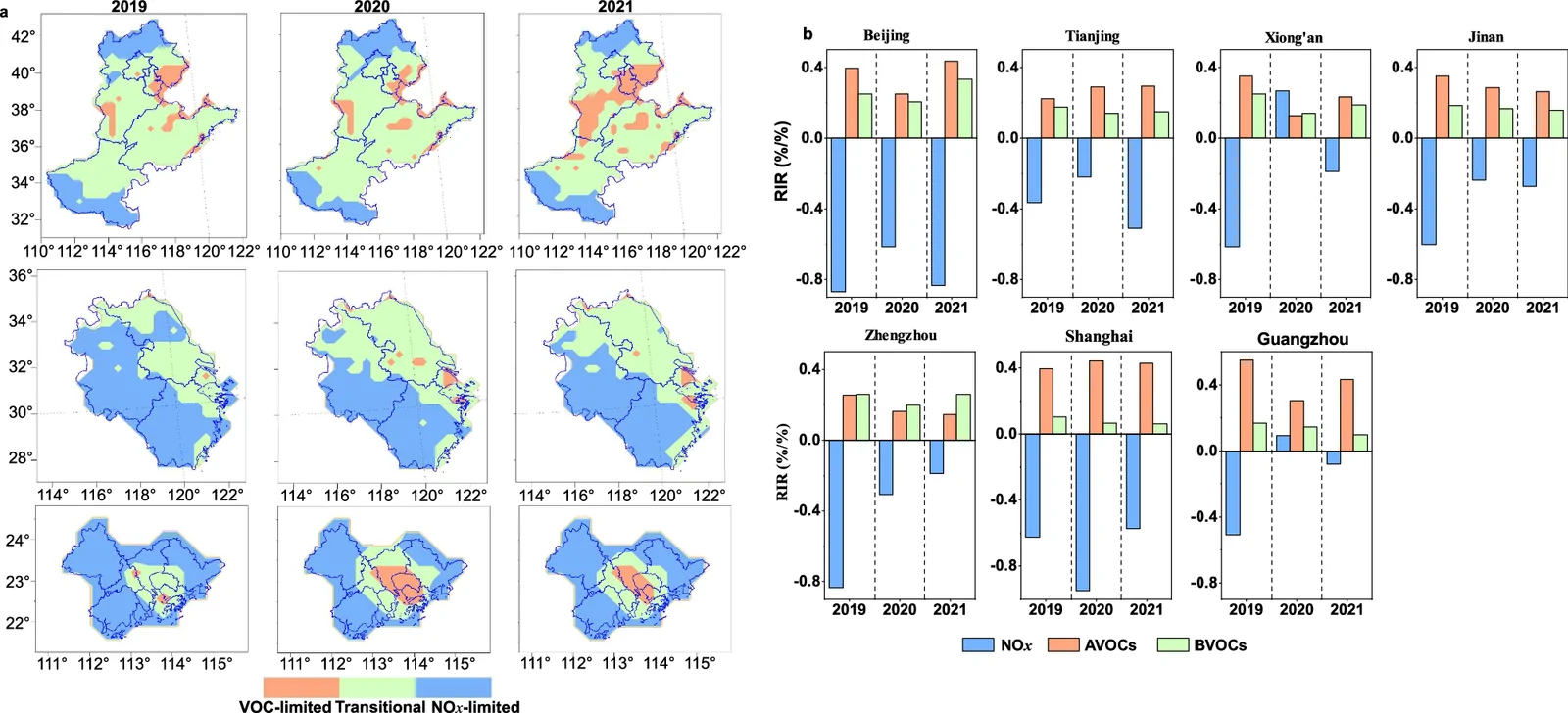
Multi-source evidence confirms the robustness of the sensitivity trend stagnation. **a** Maps illustrating O_3_ formation regimes across three megacity clusters—the North China Plain (NCP), Yangtze River Delta (YRD), and Pearl River Delta (PRD)—from 2019 to 2021. The results are derived from WRF-CAMx model simulations at a 36-km resolution. Administrative boundaries are based on the Standard Map Service of the Ministry of Natural Resources of the People’s Republic of China (http://bzdt.ch.mnr.gov.cn/), with map approval number GS(2024)0650. **b** Relative incremental reactivity (RIR) of O_3_ precursors—specifically NO*x*, anthropogenic VOC (AVOC), and biogenic VOC (BVOC)—in typical cities during the summers of 2019–2021, calculated using an observation-based model (OBM).

To ensure that the regional sensitivity trend plateau revealed by satellite FNR (Fig. 1) was not an artifact of a single metric, we further conducted a multi-scale cross-validation spanning 2019–2021 using two widely applied independent methods (Fig. 2). First, at the regional scale, congruent with our primary satellite-based finding, chemical transport model (the Weather Research and Forecasting-Comprehensive Air quality Model with extensions (WRF-CAMx) detailed in Supplementary Note 4) simulations revealed that NO*x*-limited areas peaked and that VOC-limited areas were minimized in 2020 (NCP) or 2019 (PRD, YRD). Furthermore, to stress-test these regional findings, we deployed an Observation-Based Model (OBM; detailed in Supplementary Note 5) at the urban-core scale. Given the well-documented spatial heterogeneity wherein urban centers typically exhibit a stronger sensitivity to VOCs relative to the broader regional background^14^, observing an evolutionary trend across these local sites that aligns with the broader regional trajectory provides additional auxiliary validation. Constrained by in-situ hourly measurements of VOCs and NO*x* (Supplementary Note 6), the model diagnoses sensitivity via relative incremental reactivity (RIR). This analysis (Fig. 2b) was conducted in seven cities, selected from the three megacity clusters (Supplementary Fig. 7), possessing robust data foundations, and representing diverse economic development levels in China. The inter-annual RIR trends corroborated this stagnation: the progression towards a NO*x*-limited regime stalled in 2020 for Beijing, Tianjin, Jinan, Xiong’an (NCP), and Guangzhou (PRD), and as early as 2019 for Shanghai (YRD). In the case of Zhengzhou, although its O_3_ sensitivity continued to trend towards NO*x*-limited conditions during the 2019–2021 summers, it remained persistently within the VOC-limited regime, thus still necessitating the prioritization of AVOC controls. While differing in sensitivity regime classification (Supplementary Table 7, Supplementary Figs. 1–3, and Fig. 2a), which are the limitations of static sensitivity zoning methods, all three approaches (satellite FNR, WRF-CAMx, OBM) converged on the same sensitivity trend: the progression towards the NO*x*-limited regime stalled around 2020 across the three megacity clusters. This multi-method convergence strengthens the robustness of the observed sensitivity trend plateau phenomenon. The specific period (2019–2021) was selected to scientifically bracket the 2020 inflection point and was determined by the availability of comprehensive, ground-based speciated VOC measurements required for the OBM analysis, as well as the emission inventory data available for CTM simulations.

**Fig. 3:**
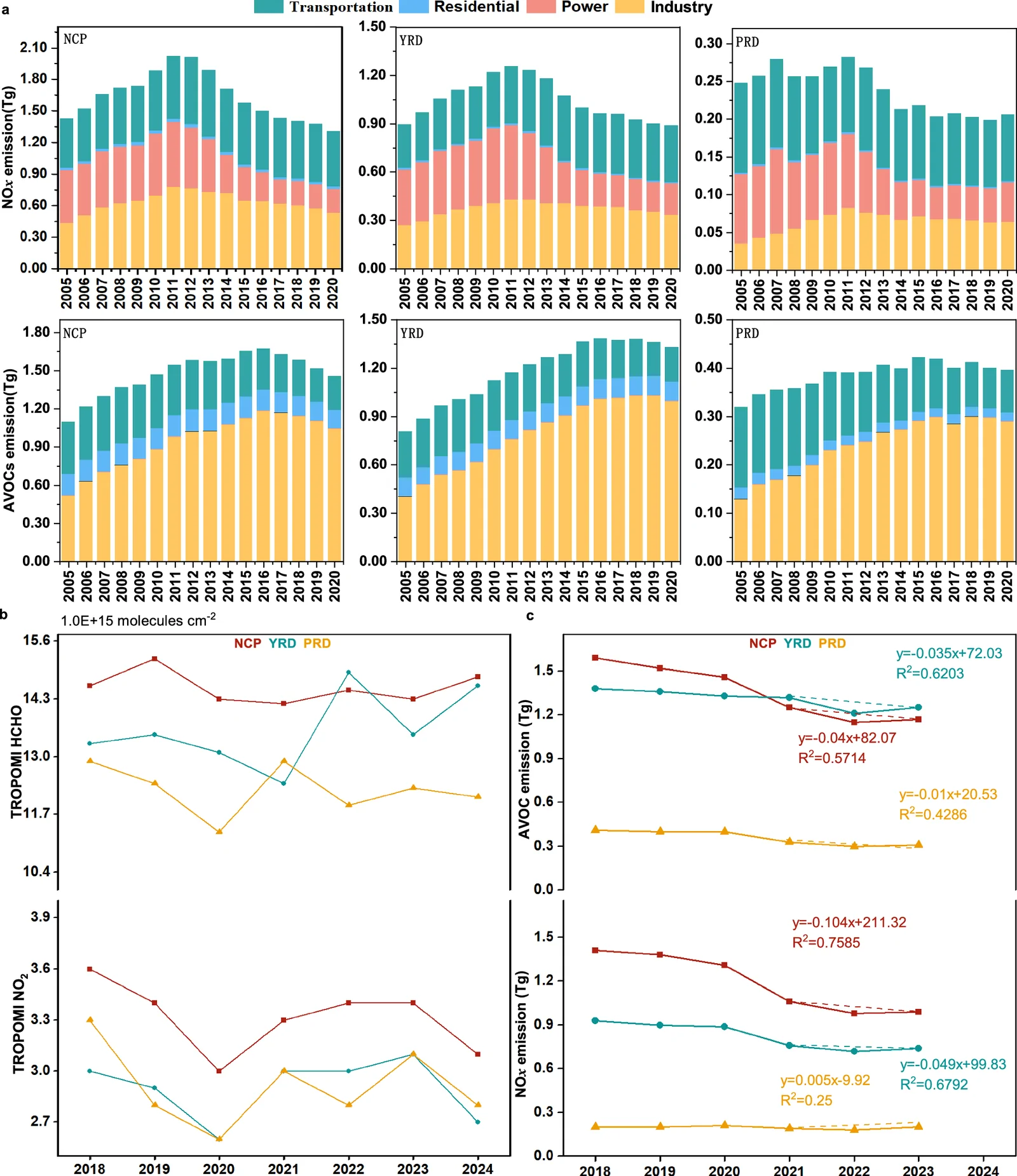
Policy-driven emission pathways explaining the observed sensitivity plateau and demonstrating path dependency. **a** Sectoral contributions to summer regional anthropogenic NO*x* and VOC emissions (2005–2020) from the Multi-resolution Emission Inventory for China (MEIC; http://www.meicmodel.org/). Note that the complete 2021 inventory was unavailable online at the time of this study. **b** Monthly mean summer tropospheric column densities of TROPOMI HCHO and NO_2_ (2018–2024). **c** Trends in aggregated summer anthropogenic NO*x* and VOC emissions (2018–2023). Linear fits for 2021–2023 quantify distinct reduction rates for each region. Aggregated emission data for 2021–2023 were obtained from the MEIC development team at Tsinghua University.

To identify the drivers of this synchronous plateau, we analyzed historical emission inventories (2005–2023) and TROPOMI satellite NO_2_ and HCHO (a VOC proxy) data (2018–2024), contextualized by China’s evolving clean air policies (Fig. 3). Policy-driven precursor emission changes determined for the trajectory of the O_3_ photochemical system over the past two decades. Four phases from 2005 were delineated based on distinct control strategies. (1): economic growth drove synchronous increases in both NO*x* and AVOC emissions, pushing O_3_ sensitivity progress towards the VOC-limited regime (indicated by decreasing FNR, Fig. 1). This occurred alongside a reported decrease in ground-level O_3_ reanalysis concentrations in most megacities in China from Copernicus atmosphere monitoring^23^. (2) 2011–2016 (12th Five-Year-Plan): a stringent clean air policy triggered rapid NO*x* decline, while AVOC emissions continued to rise^4^, initiating a shift towards the NO*x*-limited regime (increasing FNR) and driving an increase in the deweathered MDA8 O_3_ concentration. (3) 2017–2020 (Three-Year Action Plan for Winning the Blue Sky Defense Battle^23^): The pace of NO*x* reduction slowed as AVOC emissions began to decrease. O_3_ sensitivity continued to progress towards the NO*x*-limited regime. Although the deweathered MDA8 O_3_ concentration continued to increase, its anthropogenic-driven increase slowed notably—from 5.2 μg m^−3^ (2013–2017) to 1.2 μg m^−3^ (2017–2020)^4^—implying the emerging benefits of AVOC reduction. (4) The 2020–2023 synchronous plateau in the deweathered MDA8 O_3_ concentration and sensitivity coincided with distinct precursor emission features shaped by the lingering effect of COVID-19^20,24^ and the promulgation of specific AVOC control strategies since the summer of 2020^25^. Specifically, the summer total NO*x* and AVOC emissions (Fig. 3c) experienced sharp declines in 2020 or 2021, followed by further decreases in NO*x* emission reduction rates and continued AVOC emission reduction (the emission reduction rates during the summers of 2017–2020 were −0.040, −0.015, and −0.001 Tg/year for NO*x* and −0.058, −0.015, and −0.002 Tg/year for AVOCs in the NCP, YRD and PRD, respectively). This is reflected in the precursor column densities: satellite-derived NO_2_ column densities increased, whereas satellite HCHO was stable or increased slightly during the observed plateau period. This correlation between emission pathway dynamics and the observed O_3_ photochemical system response not only implies the potential effectiveness of prioritizing AVOC reductions for regional summer O_3_ abatement at the current stage but also provides a real-world demonstration of path dependency. The policy timing and reduction intensity of the precursors guide the O_3_ photochemical system into distinct response states with varied O_3_ mitigation efficacy. In contrast to conventional sensitivity zoning methods, which rely on inferences from models (such as emission-driven chemical transport models and observation-based box models) or on empirical non-linear fits (e.g., for satellite threshold determination; see Supplementary Note 3), the CDTR analysis framework is grounded directly in real-world observations. By integrating the co-evolution of deweathered MDA8 O_3_ concentrations and their sensitivity trends, the framework dynamically captures the response of the O_3_ control to divergent precursor reduction pathways, thereby providing timely and actionable qualitative feedback for the adaptive management of O_3_ abatement strategies.

### Quantitative AVOC priority under climate synergy

**Fig. 4:**
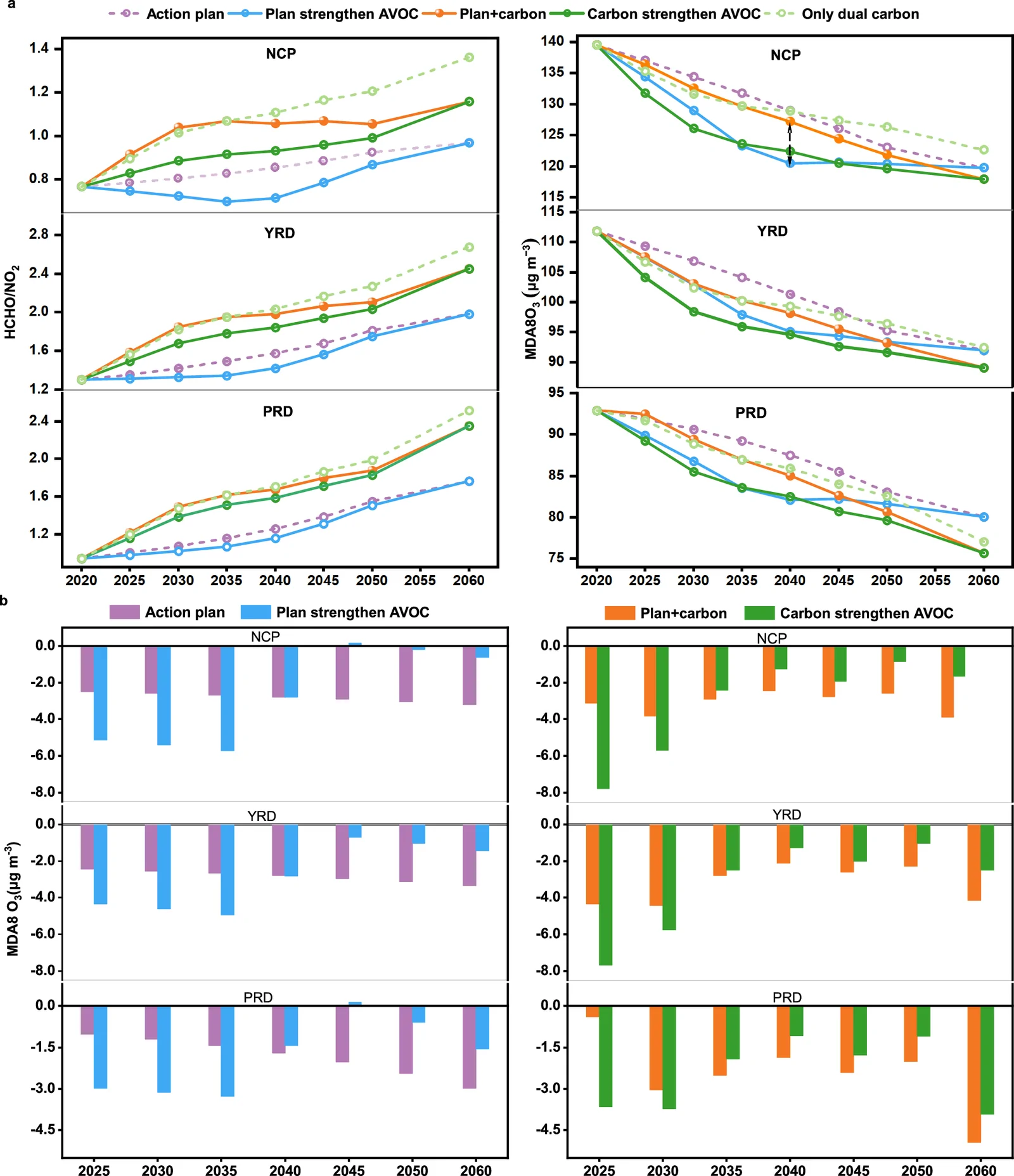
Coupled dynamic trend response of the model-derived maximum daily 8 h average (MDA8) O_3_ concentration and sensitivity from 2020–2060 quantitatively reveals path dependency and confirms the optimality of early Anthropogenic VOC (AVOC) reductions. **a** Projected changes in the interannual summer variations in model-derived formaldehyde-to-NO_2_ ratios (FNR, HCHO/NO_2_) values and the corresponding MDA8 O_3_ concentrations across the three megacity clusters from 2020–2060. The black arrow conceptually illustrates the diminishing advantage of reducing equivalent proportions of AVOCs over NO*x* on the MDA8 O_3_ decrease, shown here for NCP in 2040 as an example quantified in Table 2. **b** Projected MDA8 O_3_ concentration reductions relative to the previous five years under the paired scenarios, highlighting the initial phase differences. The meteorological field and BVOC emission inputs are fixed at the 2020 level.

To quantify the optimal O_3_ mitigation strategy under synergistic climate action and clean air policies, we applied our CDTR framework, implemented via WRF-CAMx simulations, to project the long-term (2020–2060) co-evolution of MDA8 O_3_ concentration and sensitivity trend (Fig. 4a). Five precursor reduction scenarios (Table 1) were designed to represent different reduction pathways (detailed in Methods). In brief, two benchmark scenarios, the action plan, reflect current clean air policies^26^, and the only dual carbon, refer to an ambitious NO*x*-targeted pathway designed to meet China’s climate neutrality goals within the Dynamic Projection Model for Emissions (DPEC)^27,28^. In light of historical observations and findings that underscore the significance of AVOC reduction for O_3_ mitigation at the current stage, and considering that the only dual carbon scenarios already adopted the most ambitious NO*x* reduction measures, we designed two additional scenarios: the plan strengthen AVOCs and the carbon strengthen AVOCs. These scenarios maintain the respective NO*x* reduction trajectories of the benchmark scenarios but incorporate intensified reductions in AVOCs. Finally, a hybrid scenario (plan + carbon) was designed to directly compare the efficacy of AVOC versus NO*x* controls on MDA8 O_3_ reduction. This scenario combines the NO*x* emission reduction trajectory from the only dual carbon (climate-driven) scenario with the AVOC reduction trajectory from the action plan scenario.

**Table 1:** The reduction proportions of NO*x* to Anthropogenic VOC (AVOC) from 2020 to 2060 under five different scenarios

Year	Action plan	Plan strengthen AVOCs	Plan+carbon	Carbon strengthen AVOCs	Only dual carbon
2025	10%:10%	10%:20%	30%:10%	30%:30%	30%:15%
2030	20%:20%	20%:40%	50%:20%	50%:50%	50%:25%
2035	30%:30%	30%:60%	58%:30%	58%:58%	58%:30%
2040	40%:40%	40%:70%	62%:40%	62%:62%	62%:31%
2045	50%:50%	50%:70%	68%:50%	68%:68%	68%:34%
2050	60%:60%	60%:70%	72%:60%	72%:70%	72%:36%
2060	70%:70%	70%:70%	84%:70%	84%:70%	84%:42%

The base emission amount is fixed at 2020.

Supplementary Fig. 13 provides a visual demonstration of the path dependency inherent in O_3_ abatement, revealing that the choice of emission reduction pathway dictates the evolution trajectory of the O_3_ photochemical system. Specifically, under identical reduction pathways, the evolution trajectories across the three megacity clusters exhibit convergence; however, when different precursor reduction scenarios (i.e., distinct reduction pathways) are implemented, their O_3_-FNR evolution trajectories significantly diverge. To quantitatively demonstrate the path dependency of O_3_ control (Fig. 4a, b), we designed two sets of paired scenarios with the same 2060 endpoint via distinct precursor control pathways. Specifically, the action plan scenario vs. the plan strengthens the AVOC scenario, and the plan + carbon scenario vs. the carbon strengthen the AVOC scenario. Although the total precursor reduction percentages, MDA8 O_3_ concentrations, and sensitivity indicator (FNR) values are identical for each paired scenario in 2020 and 2060, the pathways emphasizing early enhanced AVOC reductions (plan strengthen AVOCs and carbon strengthen AVOCs) yield greater MDA8 O_3_ reduction benefits in the initial 15–20 years (Fig. 4b). For example, under the plan strengthen AVOC (AVOCs:NO*x* reduction ratio = 2:1) scenario, simulated summer MDA8 O_3_ in 2025 was projected to decrease by 5.1, 4.4, and 3.0 μg m^−3^ in the NCP, YRD, and PRD, respectively, relative to 2020 levels (with meteorology fixed at 2020). These accelerated reductions are approximately double those achieved under the action plan scenario (AVOCs:NO_x_ = 1:1), which yield decreases of 2.5, 2.4, and 1.0 μg m^−3^, respectively. This enhanced early benefit is imperative, as the gains are essential for offsetting the O_3_ climate penalty (1.3-6.3 μg m^−3^) from reported more frequent heatwaves^3,4,29,30^, making the early AVOC priority pathway fundamental for achieving air quality goals in a warming world.

**Fig. 5:**
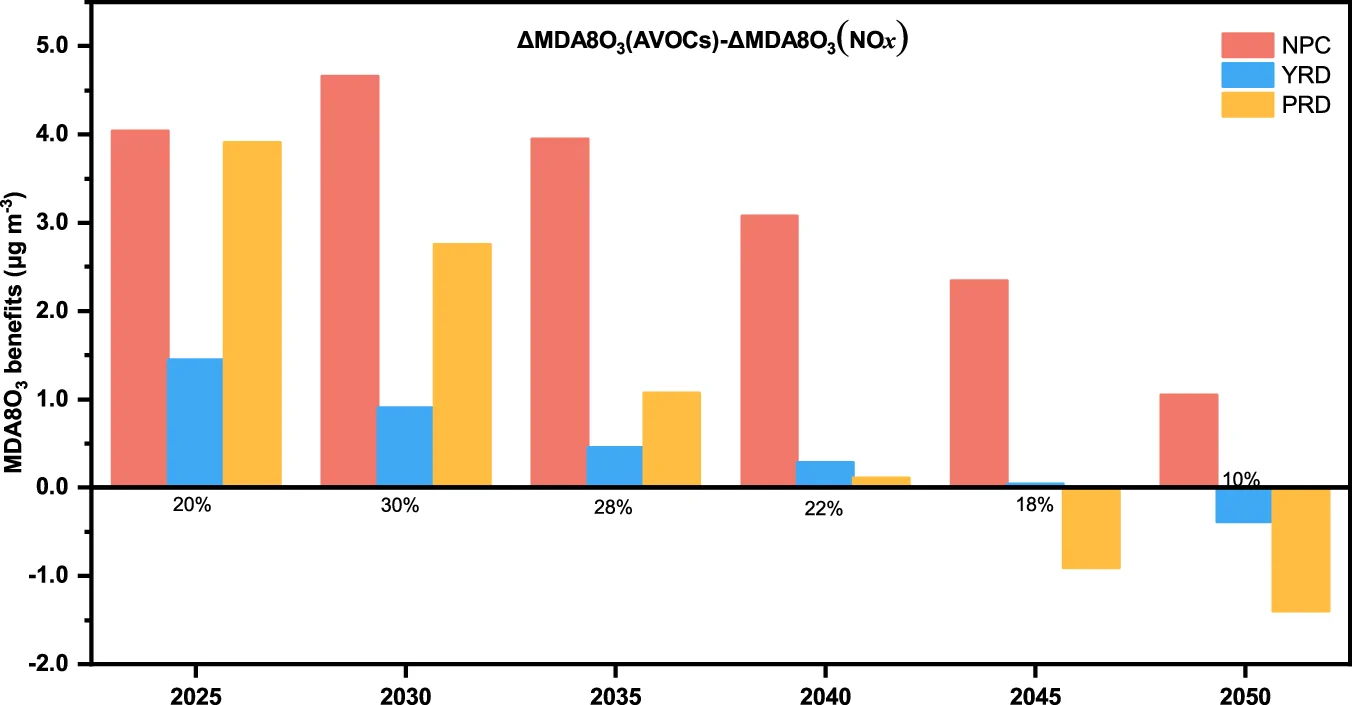
Dynamics of the anthropogenic VOCs bonus window in China’s three megacity clusters. The bonus window for prioritizing anthropogenic VOCs (AVOCs) is defined as closed when their net maximum daily 8 h average (MDA8) O_3_ mitigation benefits, calculated as the difference in MDA8O_3_ reduction between AVOCs reduction and equivalent proportion of NO*x* reductions, becomes negative. Percentages denote the precursor reduction fraction for the corresponding year. The three megacity clusters are as follows: the North China Plain (NCP), the Yangtze River Delta (YRD) and the Pearl River Delta (PRD).

This early mitigation advantage signifies a time-limited bonus window for AVOC reductions. To elucidate the dynamics of this window and its underlying photochemistry, it is essential to distinguish between two critical tipping points. The first tipping point is the crossing of the O_3_ isopleth ridgeline, marking the transition from the NO*x*-disbenefit regime to a VOC-NO*x* transition regime, where reductions in both precursors effectively lower O_3_ concentrations. The second tipping point, which signals the closure of our bonus window, is the moment when the O_3_ reduction benefit from NO*x* control surpasses that from an equivalent fractional reduction in AVOCs (NO*x*-limited regime). Our projections (Fig. 5 and Supplementary Fig. 14) reveal that China’s megacity clusters cross these tipping points asynchronously. The NCP and YRD have already crossed the ridgeline by 2025 (corroborated by independent research^31^), entering the transition regime, while the PRD follows suit later (Supplementary Fig. 14h). More critically, the bonus window for AVOC control is projected to close earliest for the PRD around 2045, followed by the YRD around 2050. In contrast, the NCP region maintains a persistent, though diminishing, AVOC control bonus beyond 2050, indicating a more prolonged window of opportunity (Fig.5). This quantitative comparison is made possible by paired scenarios to disentangle the respective contributions of equiproportional AVOC and NO*x* reductions on MDA8 O_3_ mitigation (see “Methods” and Supplementary Fig.14 for detailed derivation).

Our simulations further quantify the mechanism driving this pathway dependence: with the O_3_ sensitivity trends in all scenarios evolving towards the NO*x*-limited regime (featuring the highest FNR values in 2060), the MDA8 O_3_ mitigation advantage of reducing AVOCs over NO*x* gradually diminishes, as illustrated by the MDA8 O_3_ concentration difference between the plan +  carbon and plan strengthen AVOCs scenarios (the black arrow in Fig. 4a is an example). Quantitative analysis (Table 2) revealed that a smaller proportion of AVOC reduction than NO*x* reduction achieved greater MDA8 O_3_ reduction across the three regions before 2035. By 2050, a 10% AVOC reduction achieved a 1.4 μg m^−3^ greater MDA8 O_3_ reduction than a 12% NO*x* reduction in the NCP, showed little difference in the YRD, and yielded a 1.0 μg m^−3^ lower reduction in the PRD, indicating the declining effectiveness of reducing AVOCs.

**Table 2:** Comparison of the efficacy of Anthropogenic VOC (AVOC) and NO*x* emission reduction on maximum daily 8 h average (MDA8) O_3_ mitigation

Reduction difference	2025		2030	
	Plan+carbon-action plan	Plan strengthen AVOC—action plan	Plan+carbon-action plan	Plan strengthen AVOC—action plan
NOx	20%	0	30%	0
AVOCs	0	10%	0	20%
ΔO_3_^①^	NCP: 2.0 µg m^−3^ YRD: 0.01 µg m^−3^ PRD: 2.6 µg m^−3^		NCP: 3.6 µg m^−3^ YRD: 0.2 µg m^−3^ PRD: 2.7 µg m^−3^	
Reduction difference	2035		2040	
	Plan+carbon-action plan	Plan strengthen AVOC—action plan	Plan+carbon-action plan	Plan strengthen AVOC—action plan
NO*x*	28%	0	22%	0
AVOCs	0	22%	0	30%
ΔO_3_^①^	NCP: 6.4 µg m^−3^ YRD: 2.3 µg m^−3^ PRD: 3.4 µg m^−3^		NCP: 6.7 µg m^−3^ YRD: 3.0 µg m^−3^ PRD: 3.0 µg m^−3^	
Reduction difference	2045		2050	
	Plan+carbon-action plan	Plan strengthen AVOC—action plan	Plan+carbon-action plan	Plan strengthen AVOC—action plan
NO*x*	18%	0	12%	0
AVOCs	0	20%	0	10%
ΔO_3_^①^	NCP: 3.8 µg m^−3^ YRD: 1.1 µg m^−3^ PRD: 0.4 µg m^−3^		NCP: 1.4 µg m^−3^ YRD: −0.1 µg m^−3^ PRD: −1.0 µg m^−3^	

^①^ΔO_3_ represents the additional MDA8 O_3_ reduction achieved by the plan-strengthen AVOCs scenario relative to the plan +  carbon scenario after subtracting the common action plan baseline, calculated as Δ(plan-strengthen AVOCs—action plan) − Δ(plan + carbon—action plan). Positive values indicate a larger O_3_ mitigation benefit from additional AVOC reductions than from additional NO*x* reductions.

These findings challenge the conventional strategy that favors rapid transition to a NO*x*-limited regime for O_3_ attainment, with synergistic AVOC reductions merely offsetting NO*x*-reduction-induced O_3_ rebounds in the VOC-limited regime^32–34^. Our findings challenge this paradigm by demonstrating that the only dual carbon scenario—which engineers the fastest transition to a NO*x*-limited regime—yields the least overall O_3_ improvement (PRD except, Fig. 4a). Adhering to the traditional strategy that overemphasizes NO*x* reductions and neglects this time-limited AVOC reduction bonus window risks an O_3_ dilemma of persistently high O_3_ despite deep NO*x* emission reductions. Even with an 84% NO*x* emission reduction by 2060, the projected summer MDA8 O_3_ concentrations in the NCP far exceed the World Health Organization recommended guideline (100 μg m^−3^), highlighting the necessity of fully leveraging the AVOC reduction bonus window. This phenomenon may offer a new perspective on the O_3_ challenges faced by some developed countries, despite substantial historical NO*x* reductions^3^.

## Discussion

**Fig. 6:**
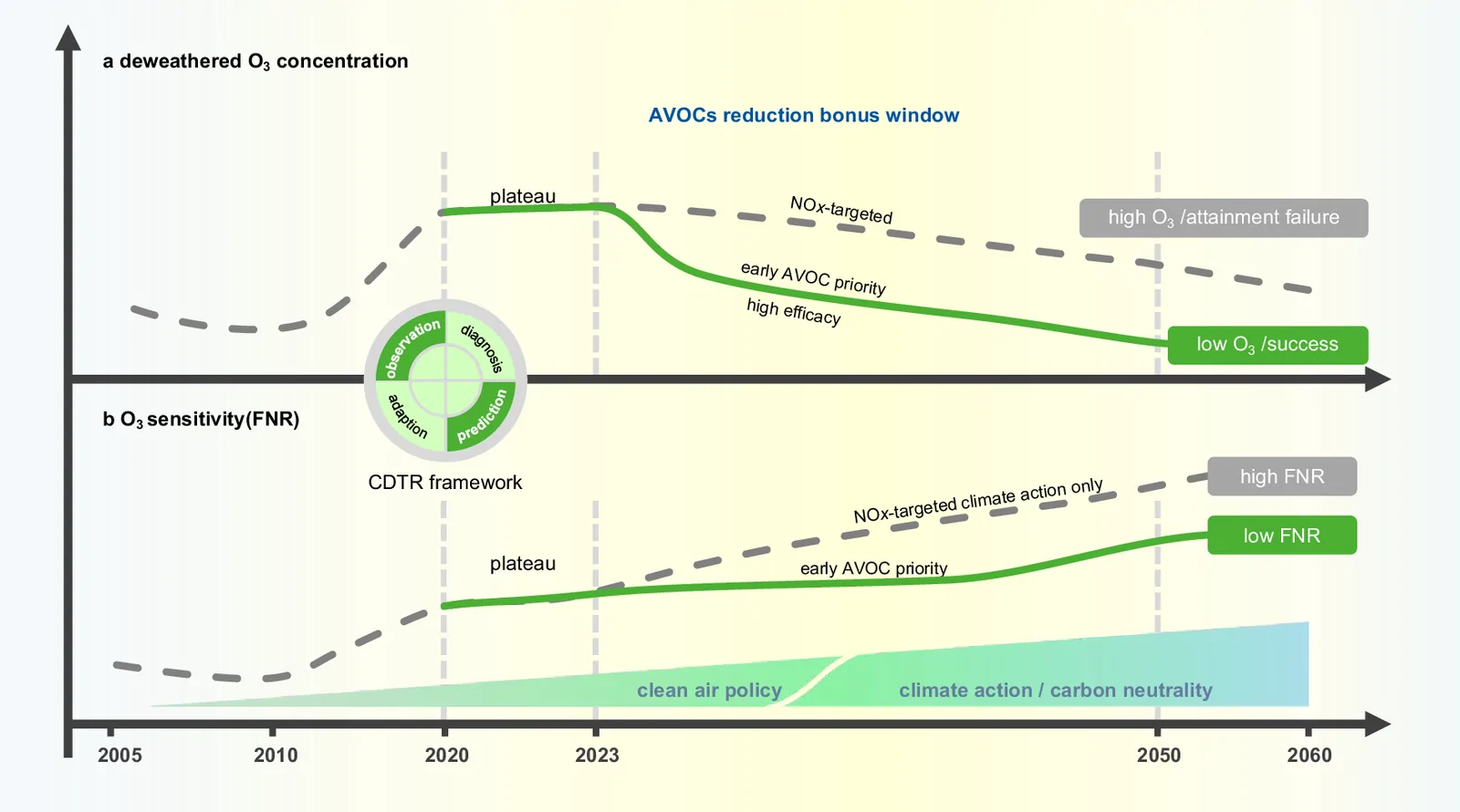
Observation-constrained optimal O_3_ control pathway for synergistic air-quality and climate goals. The schematic summarizes the historical evolution and future control direction of the regional summertime O_3_ photochemical system in China under different policy-driven emission pathways. **a** Evolution of deweathered O_3_ concentrations from 2005 to 2060 (China’s carbon-neutrality target year) under different control pathways. **b** Corresponding evolution of the O_3_ formation sensitivity indicator FNR, defined as the formaldehyde-to-NO_2_ ratio. Higher FNR values indicate a faster shift towards a NO*x*-limited regime. Compared with the NO*x*-priority climate-action-only pathway that accelerates this transition, early strengthened reductions in anthropogenic VOCs (AVOCs) yield larger O_3_ mitigation benefits while the system remains at lower FNR values. The circular schematic illustrates the workflow of the coupled dynamic trend response (CDTR) framework, which utilizes observational feedback of the co-evolution of O_3_ concentrations and sensitivity to diagnose control priorities and project the optimal future control strategy. The synchronous plateauing of deweathered O_3_ concentrations and sensitivity trends during 2020–2023 provides observational evidence that strengthened AVOC controls should be prioritized at the current stage. Constrained by this historical signal, future scenario simulations indicate that AVOC controls should be further intensified beyond existing clean-air policies and climate actions until around 2050. By maximizing the time-limited “bonus window” for AVOC reductions, this strategy not only secures deeper long-term O_3_ mitigation but also accelerates near-term O_3_ improvements to counteract the rebound risks associated with increasingly frequent heatwaves. This blueprint highlights the need to shift regional summer O_3_ governance from static sensitivity zoning toward an adaptive, pathway-dependent co-management paradigm.

Figure 6 synthesizes our findings into a dynamic policy blueprint for an optimal O_3_ mitigation strategy that aligns clean air and climate objectives. This schematic goes beyond depicting the past coevolution of the deweathered O_3_ concentration and sensitivity driven by different clean air policies. More importantly, leveraging our findings of path dependency and the directive of early intensified AVOC abatement, it proposes a recommended control pathway under current (near-term with clean air policy dominant) and future (medium-to-long-term with deeply synergistic climate-clean air policy) contexts. Specifically, for approximately the next 25 years (to ~2050), AVOC reductions should be prioritized for further strengthening beyond existing clean air policies and climate actions. This strategic emphasis aims to fully leverage the identified AVOC reduction bonus window period, thereby achieving the fastest near-term O_3_ improvements, which are required for meeting clean air policy targets and effectively countering additional challenges caused by climate change. As climate action naturally drives substantial NO*x* emission reductions over time, the O_3_ photochemical system will robustly transition towards being increasingly NO*x-*limited. The control priority should be adjusted based on real-time atmospheric responses observed via the CDTR analysis framework. The timeline in Fig. 6 is based on current policy projections, and the adaptability of the CDTR analysis framework allows for iterative updates based on real-time observational feedback and evolving policies, thus maintaining relevance in a rapidly changing world. The optimal O_3_ mitigation strategy proposed in our study is defined on the basis of photochemical efficacy (i.e., the MDA8 O_3_ abatement achieved per equivalent proportional reduction in precursors), independent of techno-economic complexities. However, for the design and implementation of real-world emission control policies, integrating this photochemical optimization with practical economic cost assessments is essential.

This research addresses a challenge: how to overcome the limitations of the traditional static sensitivity zoning method to formulate effective O_3_ control strategies amid complex long-term dynamic emission changes driven by multiobjective policies. By the proposed CDTR framework, we identify path dependency as the key mechanism and quantitatively demonstrate that early intensified AVOC control based on current clean air policy and climate actions, rather than prematurely pushing the sensitivity to become NO*x*-limited by aggressive NO*x* reduction, is the optimal strategy. This challenges prevalent O_3_ control policies and underscores the urgency and importance of formulating dynamic, adaptive strategies. The observed synchronous plateauing of the deweathered MDA8 O_3_ concentration and sensitivity not only serves as strong real-world evidence of policy-driven path dependency but also highlights the value of the CDTR framework in capturing dynamic responses that traditional static sensitivity zoning methods inherently miss. The CDTR analysis framework and the AVOC reduction bonus window concept offer immediate and actionable insights for policymakers, fostering more resilient, effective and forward-looking air quality‒climate management over several decades.

A critical methodological consideration in O_3_ sensitivity assessment is that discrepancies in the absolute values of derived FNR across different methodologies—whether from satellite retrievals or chemical transport models— remain systematic, multi-sourced, and inherently challenging to reconcile^13–18^. Even among different retrieval products derived from the same satellite sensor, substantial FNR value offsets persist (e.g., as demonstrated by Jin et al.^35^). Our direct column-to-column FNR comparison (Supplementary Fig. 4) during summer 2020 (baseline year) confirms that while such discrepancies are substantially reduced compared to column-to-surface assessments—with both maintaining consistent temporal variations—a systematic baseline offset persists. Rather than viewing this inherent FNR variability as a limitation, it essentially highlights the necessity and the core rationale of our CDTR analytical framework. We posit that historical reliance on static absolute FNR thresholds for sensitivity zoning largely drives the inconsistent conclusions regarding summer regional O_3_ sensitivity in China (Supplementary Note 1). By shifting focus to the robust relative evolutionary trends of sensitivity indicators (Supplementary Note 3), our CDTR framework insulates core policy insights from these absolute FNR baseline biases. This dynamic perspective yields fundamental insights: by aligning with the sensitivity evolution direction, the observed synchronous plateau provides an empirical directive that prioritizing AVOC control is the more effective strategy at present and guides the intensification focus of our future scenarios. Crucially, our simulations (Fig. 4) quantitatively demonstrate that O_3_ mitigation efficacy does not necessarily align with the speed of transition towards a NO*x*-limited regime (the only dual carbon scenario in Fig. 4a), thereby challenging the traditional paradigm that advocates for the fastest possible entry into NO*x*-limited conditions. Ultimately, we define the closure of the bonus window—the tipping point into a NO*x*-limited regime—by comparing the O_3_ abatement efficacy of equivalent proportional reductions in AVOCs versus NO*x*, successfully bypassing the utilization of FNR absolute values (Fig. 5). Consequently, the robustness of our conclusions regarding the optimal mitigation strategy is independent of systematic methodological offsets in absolute baseline FNR determinations.

To isolate the independent effects of anthropogenic emission abatement, our future projections fixed meteorological fields and BVOC emissions at 2020 baseline levels. However, future climate change, characterized by projected rising temperatures and more frequent heatwaves, is expected to increase BVOC emissions^30,36^. This would drive the photochemical environment towards a more VOC-rich and thus more NO*x*-limited state. This means future increases in biogenic volatile organic compound (BVOC) would likely accelerate the transition to a NO*x*-limited regime and thus result in a shorter period for AVOC reduction bonus window. However, this prospect does not undermine our core conclusion; rather, it magnifies the strategic urgency of capitalizing on this window of opportunity for AVOCs abatement before it closes. More complex climate-O_3_ interactions, such as temperature effects on agricultural NO*x* and AVOCs emissions^36^, and anthropogenic NO*x* and AVOCs emission pattern change caused by heatwave-driven shifts in energy structure^37^, remain critical areas for future investigation. Similarly, methane (CH_4_) concentration in our simulations adopted the CAMx model’s default setting of 1.85 ppmv. This simplification aims to focus on the direct impacts of regional precursor controls. CH_4_ is reported to influence global O_3_ background levels^38^. If future global CH_4_ concentrations rise as predicted^39^, this will inevitably increase background O_3_, adding additional pressure on O_3_ attainment. This rising baseline reinforces the need to leverage the AVOC bonus window to maximize abatement efficacy. While our simplification facilitates a clear assessment of regional strategies, future work could integrate coupled Earth System Models to explore the synergy between global CH_4_ evolution and regional O_3_ policies.

Similarly, we acknowledge that the evolving speciation of AVOCs can influence the predicted efficacy of O_3_ abatement. Given the differential photochemical reactivities among VOC species^40–42^, the strategic focus of reduction—for instance, prioritizing highly ozone formation potential species (high-OFP) like alkenes or aromatics—would undoubtedly influence the duration of the AVOC reduction bonus window and the MDA8 O_3_ mitigation effect. Although the emission scenarios in Fig. 4 were simulated by applying mass-based reductions to the 2020 AVOC emission inventory, the inherent adaptability of our CDTR framework equips it to address the challenge posed by evolving species. It is not a static, one-off prediction tool but a dynamic analytical framework. Its observational component continuously integrates multi-source real-time data, including satellite and ground-based monitoring, to evaluate the response of deweathered MDA8 O3 concentrations and sensitivity trends to evolving precursors caused by different policies and activity patterns (e.g., COVID-19). Simultaneously, its modeling component can be iteratively updated by incorporating updated emission inventories and leveraging the DPEC model. The DPEC model is specifically tasked with predicting future dynamic emissions by integrating anticipated socioeconomic development, technological advancements, and evolving clean air and climate policies into the CDTR analytical framework (see “Methods”). To illustrate this, we employed the CDTR framework to further explore the impacts of reactivity-based AVOC control strategies. We calculated precursor reduction ratios of prioritizing completely removal of high-OFP species (alkenes and aromatics), and then compared the outcomes with those from the mass-based AVOC reductions control strategies with equal-proportion reduction of total AVOCs (see “Methods,” Supplementary Figs. 15–16, and Supplementary Tables 11–13). The results indicate that prioritizing high-OFP species significantly accelerates the evolution of O_3_ sensitivity towards the NO*x*-limited regime, as evidenced by markedly higher FNR values across all scenarios compared to equal-proportion reduction (Supplementary Fig. 15). However, this did not uniformly translate into superior O_3_ concentration mitigation benefits (Supplementary Fig. 16). Specifically, only under the plan+carbon scenario, which involves deep NO*x* reductions, did this strategy show a clear advantage in reducing MDA8 O_3_ concentrations in the NCP and YRD. Conversely, in scenarios with less aggressive NO*x* control, its efficacy was even inferior to that of the mass-based control strategy. In the PRD megacity cluster under the plan+carbon scenario, the MDA8 O_3_ mitigation benefit of reactivity-based reduction proved less effective than that of the mass-based reduction, potentially due to the PRD entering the O_3_ formation transition regime later than the other two megacity clusters (Supplementary Fig. 14h). The varying effectiveness of the reactivity-based reduction strategy reveals a strong path dependency, quantitatively demonstrating that the efficacy of control strategies is highly contingent on the evolutionary stage of the O_3_ photochemical system. In the early stages of the AVOC reduction bonus window (Fig. 5), the concentrated reduction of high-OFP species may be less efficient than the broad reduction of a larger mass of various AVOCs. Therefore, these findings do not negate the value of reactivity-based strategies but rather underscore the necessity of precision timing in their implementation. This validates the adaptive nature of our proposed CDTR framework: it provides an essential dynamic diagnostic capability to assess the state of the photochemical system in time, thereby determining the optimal timing to transition from mass-based control to more refined, reactivity-based control, ensuring scientific and efficient policy adjustments.

Finally, situating our findings within a broader spatiotemporal context reveals additional strategic implications. Our finding of a long-term summer AVOC control priority extending to approximately 2050, coupled with the recognized seasonality in O_3_ sensitivity (i.e., VOC-limited in winter and increasingly NO*x*-limited in summer due to elevated biogenic emissions at higher temperatures, etc.^43^), collectively suggests that implementing year-round AVOC strengthening constitutes a win-win strategy for China, fostering both sustained air quality improvement and climate change mitigation. This year-round AVOC intensification offers cobenefits to address increasingly frequent high O_3_ episodes in late spring and persistent PM_2.5_ exceedances in winter^6,7,44^. Furthermore, the spatial heterogeneity of O_3_ sensitivity indicates that urban centers will require differentiated AVOC control efforts to counteract the O_3_ rebound risks triggered by the substantial NO*x* reductions inherent to climate actions (Supplementary Figs. 17–19). An illustration is observed in the PRD (Supplementary Fig. 19), although the broader PRD region is projected to pioneer the transition to a NO*x*-limited regime by 2045, these substantial NO*x* curtailments sustain localized MDA8 O_3_ rebounds—relative to the 2020 baseline—in sporadic urban hotspots even as late as 2060, underscoring that such micro-environments remain entrenched in a VOC-limited state. While our current regional modeling framework captures these macro-scale chemical transitions, future iterations of the CDTR framework will benefit from deploying nested air quality simulations at high urban-centric resolutions (e.g., 4 km or 1 km) to precisely resolve such fine-scale spatial heterogeneity in O_3_ responses and to design differentiated urban vs. regional strategies. The future emission scenarios and model setups used in this study (e.g., fixed 2020 meteorology and biogenic emissions; maximum VOC and NO*x* reduction potentials set at 70% and 84% of 2020 levels, respectively, based on literature^45,46^) represent reasonable estimates based on current knowledge. The adaptability of the CDTR method provides a robust tool for navigating future uncertainties by allowing projections to be updated with new observational data, different air quality models and future technological advancements.

## Methods

### Coupled dynamic trend response (CDTR) analysis framework

To address the limitations of traditional static sensitivity zoning methods in formulating effective O_3_ mitigation strategies amidst multi-decadal, dynamic changes in precursor emissions (including both NO*x* and VOCs), we developed, validated, and applied the CDTR analysis framework. The CDTR is not a custom computational algorithm but a structured, two-phase analytical approach that integrates multiple established techniques to quantitatively assess the path dependence of O_3_ control strategies. By focusing on the co-evolution of the O_3_ photochemical system (deweathered O_3_ concentrations and sensitivity) across multi-decadal timescales, this framework identifies optimal control strategies by evaluating the coupled dynamic response of the O_3_ photochemical system to various emission reduction strategies. The framework comprises the following reproducible steps:

### Phase I: Historical validation of pathway dependence

This phase empirically validates the coupled response of the O_3_ photochemical system to policy-driven emission changes, using long-term field observation data as a real-world testbed.

Step 1a: Isolating emission-driven O_3_ trends. We applied meteorological normalization techniques, such as Generalized Boosted Models (GBMs)^47^, to surface summer O_3_ monitoring data (2013–2024) to generate deweathered O_3_ concentration time series. This process isolates the signal driven by precursor emission changes by normalizing meteorological effects (details in Supplementary Note 2).

Step 1b: Characterization of long-term sensitivity trends. Using OMI and TROPOMI satellite measurements of O_3_ precursors (NO_2_ and HCHO) (2005-2024), we analyze the interannual variations of O_3_ sensitivity indicators (details in Supplementary Note 3), including the satellite-derived FNR and the area proportion of sensitivity regimes (2005–2024). To validate the robustness of the synchronous plateauing phenomenon observed from 2020-2023, sensitivity trends for 2019-2021 were additionally investigated using both a chemical transport model (WRF-CAMx in this study; details in Supplementary Note 4) and an Observation-Based Model (OBM; details in Supplementary Note 5). Results from all three methods consistently revealed a stagnation in the trend of O_3_ sensitivity towards NO*x*-limited regimes in either 2019 or 2020. This trend-oriented approach circumvents the inconsistencies inherent in conventional sensitivity zoning methods, which arise from the use of static, absolute thresholds. Detailed information on sensitivity trends and their robust performance is provided in Supplementary Note 3.

Step 1c: Analysis of drivers of coupled response. “Coupling” refers to the synchronous analysis of deweathered O_3_ concentration trends and sensitivity trends, examining their dynamic response to various emission pathways across multi-decadal scales. Interpreting their coupled behavior (e.g., the synchronous plateauing observed from 2020 to 2023) in the context of historical emission pathways provides real-world evidence for path dependence and validates the framework’s capability to timely capture dynamic signals. This framework implies that AVOCs reduction is more effective in the current phase, guiding the design of future quantitative prediction scenarios for Phase II (reinforcing AVOCs control beyond current regulatory strategies).

### Phase II: Quantitative prediction of optimal future O_3_ mitigation strategy

This phase applies the framework in a predictive mode, utilizing a chemical transport model (WRF-CAM*x*) to quantitatively identify optimal long-term control strategies.

Step 2a: Design of policy-relevant scenarios. We designed five future emission reduction scenarios (2020–2060) representing distinct control pathways, based on insights from field observations, current clean air policies, and long-term carbon neutrality targets (details in the following Setting scenarios). The integration of the DPEC model into our scenario design also equips the CDTR analytical framework with the capability for iterative updates, allowing it to dynamically reflect evolving socioeconomic trajectories, advancements in pollution control technologies, and evolving clean air policies and climate actions.

Step 2b: Simulation of synergistic evolution of O_3_ concentration and sensitivity trends. For each scenario, we simulated future summer MDA8 O_3_ concentrations and FNR values using WRF-CAMx. To isolate the impact of anthropogenic emissions, meteorological conditions and BVOC emissions were fixed at 2020 levels, thereby directly yielding model outputs under identical meteorology (analogous to the deweathered state of field observations).

Step 2c: Identification of optimal strategy via comparative analysis. We plotted the simulated evolution trajectories of MDA8 O_3_ concentrations and FNR values under identical meteorological influences for all scenarios from 2020–2060 (as shown in Fig. 4a). By quantitatively comparing the coupled response of the O_3_ photochemical system across different emission trajectories, we identified the optimal mitigation pathway—one that achieves both near-term national air quality targets and long-term WHO guidelines. This analysis also enabled us to quantify the duration of the AVOC reduction bonus window under the current policy trajectory (details in the Quantification method for the duration of AVOCs reduction bonus window section), thus providing a quantitative basis for comparing strategic choices. This comparative analysis constitutes the quantitative core of the CDTR framework.

The application of the CDTR to historical observations, shaped by various implemented clean air policies, revealed its capacity to capture dynamic signals that traditional sensitivity zoning methods miss. These dynamic signals provide qualitative insights for optimizing O_3_ mitigation. When applied to future simulations, the CDTR serves as a quantitative tool to compare the efficacy of various control strategies in achieving the desired air quality and climate goals at multidecadal scales, and to assess the optimal O_3_ control strategy for the coming decades. This approach reframes O_3_ mitigation from a static problem to an adaptive management process. This adaptability allows projections to be iteratively updated with real-time observational feedback and evolving policy and technologies, ensuring that the framework remains a relevant and robust tool for navigating future air quality and climate challenges.

### Setting scenarios

Five emission reduction scenarios (Table 1) were developed to align with China’s ongoing clean air policy^22^ and its climate action commitments (carbon neutrality target)^27^. These scenarios are based on two distinct approaches. Two scenarios, based on current air quality policies (action plan and plan strengthen AVOCs), adopt a conventional static reduction proportion relative to a base year (2020). In contrast, our three climate-oriented scenarios (only dual carbon, carbon-strengthened AVOCs and plan +  carbon) employ dynamic precursor reduction proportions relative to 2020 levels from the DPEC in China^27,28^. These dynamic reduction proportions reflect anticipated future emission changes driven by socioeconomic development, climate actions, and advancements in pollution control technology. Specifically, because NO*x* emissions are more closely linked to energy transitions towards renewables under carbon neutrality actions than to AVOCs^10^, we determined NO*x* emission reduction trajectories by referencing the *Ambitious-Pollution-Neutral-Goals* (APNG) scenario within the DPEC model^27,28^. The DPEC-APNG scenario projects that China will achieve carbon neutrality by 2060, with a CO_2_ emission trajectory between representative concentration pathways 1.9 and 2.6. This approach ensures that our emission reduction scenarios better represent potential real-world dynamics. The following describes the design of these five emission reduction scenarios.

We designed two scenarios to represent pathways based on current air quality policies. The action plan scenario simulates the implementation of China’s current clean air policy^22^. It assumes a linear reduction of 10% every five years for both NO*x* and AVOCs relative to 2020 levels, extending through 2060. This establishes a baseline policy pathway with a constant 1:1 reduction ratio between the two precursors. The plan strengthens the VOCs scenario by investigating the impact of prioritizing AVOC abatement. It retains the same NO*x* reduction trajectory as the action plan but doubles the AVOC reduction rate, thereby enforcing a 1:2 NO*x*-to-AVOC reduction ratio. This intensified AVOC control is applied until the cumulative reduction reaches 70% (projected for 2040). This scenario explores the impact of prioritizing AVOC control within the scope of the clean air policy.

We further defined two scenarios that represent pathways based on climate actions towards carbon neutrality. The only dual carbon scenario represents a future where emission reductions are driven by decarbonization. The NO*x* emission reduction trajectory is derived from the DPEC-APNG scenario within the DPEC model, which adopted the most ambitious NO*x* pollution control measures. Under this scenario, the ratio of NO*x* to AVOC emissions was set to 2:1, reflecting partially shared emission sources. This ratio was set for two reasons: first, Shi et al.^46^ projected that the projected emission reductions of NO*x* and AVOCs would be 90% and 61% in 2060 compared with 2020, respectively; and second, United States Environmental Protection Agency (US EPA) historical data show NO*x* and AVOC emission reductions of 71% and 48%, respectively, between 1990 and 2020 in the U.S. (https://gispub.epa.gov/air/trendsreport/2023/, accessed on 29 April 2024). The carbon-strengthened AVOCs scenario builds upon the NO*x* reductions of the only dual carbon scenario but includes additional AVOC control, resulting in a 1:1 NO*x*-to-AVOC reduction ratio. This scenario represents a synergistic climate and clean air policy.

Finally, a hybrid scenario (the plan + carbon) was designed for comparison. This scenario combines the NO*x* emission reduction trajectory from the only dual carbon scenario (climate-driven) with the AVOC reduction trajectory from the action plan scenario. Compared with the plan strengthen AVOCs scenario, this scenario allows a comparison of the efficacy of AVOC and NO*x* reductions on O_3_ reduction. This occurred because the total reduction amounts of AVOCs and NO*x* in these two scenarios from 2030 to 2050 are similar, despite the different relative emphasis on NO*x* versus AVOCs. Crucially, the design of this hybrid emission reduction scenario enables clear visualization of the AVOC bonus window for O_3_ mitigation.

The grounding of these scenarios in policy-relevant pathways synergistic with clean air and climate actions, combined with the CDTR’s ability to evaluate the resulting long-term dynamic responses, provides the foundation for its adaptability and relevance.

### Derivation of NO*x*-to-AVOC reduction ratios for reactivity-based O_3_ control strategy tests

To quantitatively assess the differential impacts of reactivity-based versus mass-based O_3_ control strategies from 2020 to 2060, we designed a series of tests to compare the effects of the two strategies on O_3_ sensitivity trends and on MDA8 O_3_ concentrations under fixed meteorological and biogenic emission conditions (Supplementary Figs. 15–16) across different scenarios (action plan, plan strengthen AVOCs, and plan + carbon). In the tests of reactivity-based strategies, we targeted the reduction of high Ozone Formation Potential (OFP) species (alkenes and aromatics), and determined the corresponding NO*x* and AVOC reduction ratios according to the following procedure:

First, based on the MEIC inventory for June–August 2020, we calculated the total emissions of alkenes and aromatics in the three megacity clusters (NCP, YRD, and PRD). Note that alkenes here refer exclusively to anthropogenic sources, excluding biogenic isoprene.

Subsequently, the emissions of these high-OFP species were divided by the total AVOC emissions for the same period to derive the mass fraction for alkenes and aromatics in each megacity cluster.

Finally, based on the NO*x*:AVOC reduction ratios for different scenarios as defined in Table 1, we determined the corresponding NO*x* reduction targets when prioritizing the reduction of these high-OFP species (see Supplementary Tables 11–13). Firstly, for the action plan scenario, this scenario adheres to a 1:1 coordinated NO*x*:AVOC reduction ratio. Accordingly, when alkenes (or aromatics) were preferentially abated, the NO*x* reduction percentage was set to be equal to the mass fraction of that specific species group. Secondly, for the plan strengthen AVOCs scenario, this scenario adheres to a 1:2 coordinated NO*x*:AVOC reduction ratio. The NO*x* reduction percentage was therefore set to half the mass fraction of the targeted alkene (or aromatic) group. Thirdly, for the plan + carbon scenario, as the NO*x* reduction pathway for this scenario is derived from the only dual carbon scenario, we employed a trajectory mapping approach. Specifically, we matched the 2020 mass fraction of alkenes or aromatics to the year in the AVOC reduction trajectory of the only dual-carbon scenario with the closest reduction percentage. The NO*x* reduction percentage from that matched year was then adopted as the corresponding NO*x* reduction target. For instance, in the summer of 2020, aromatics in the NCP region constituted 30.3% of total AVOC emissions. This value most closely matches the AVOC reduction level for the year 2030 in the only dual carbon scenario. Therefore, the corresponding NO*x* reduction target of 58% for that year was adopted.

### Quantification method for the duration of the AVOCs reduction bonus window

To quantitatively assess the duration bonus window of a prioritized AVOCs control strategy, we calculated the difference in O_3_ reduction efficacy resulting from equivalent proportional reductions of AVOCs versus NO*x* (Fig. 5). This calculation is based on a comparative analysis of simulation results from three scenarios, whose O_3_ photochemical system evolutionary trajectories are shown in Supplementary Fig. 14a–f. Specifically, the plan+carbon scenario shares an identical AVOC reduction trajectory with the action plan scenario but implements greater NO*x* reductions. Therefore, subtracting the plan+carbon scenario from that of the action plan scenario isolates the MDA8 O_3_ reduction attributable to the additional NO*x* abatement in that year. Conversely, the carbon strengthen AVOCs scenario maintained the same NO*x* reduction targets as the plan+carbon scenario but implements greater AVOC reductions. Thus, subtracting the carbon strengthen AVOCs scenario from that of the plan+carbon scenario isolates the MDA8 O_3_ reduction attributable to the additional AVOC abatement. The AVOCs reduction bonus window closes when the MDA8 O_3_ mitigation benefit from equiproportional NO*x* reduction exceeds that of AVOCs reduction. We quantify this by subtracting the MDA8 O_3_ benefit caused by more AVOCs reduction (Supplementary Fig. 14g) from those under the parallel more NO*x* reduction (Supplementary Fig. 14h). The point at which this difference (Fig. 5) becomes negative marks the transition to a NO*x*-limited regime, where NO*x* control becomes the more effective strategy.

This derivation, explicitly illustrated in Supplementary Fig. 14 and Fig. 5, involves the following three steps:

First, we isolated the O_3_ reduction benefit from AVOCs reduction by subtracting the carbon strengthen AVOCs scenario from those of the plan+carbon scenario. This difference represents the net MDA8 O_3_ reduction solely attributable to the enhanced AVOC control measures (Supplementary Fig. 14g). The percentages shown in the figure represent the proportion of additional AVOC reduction for that year.

Second, we isolated the O_3_ reduction benefit from NO*x* reduction by subtracting the plan+carbon scenario from that of the action plan scenario. This isolates the net MDA8 O_3_ reduction solely attributable to the additional NO*x* abatement (Supplementary Fig. 14h). The percentages shown in the figure represent the proportion of additional NO*x* reduction for that year.

Third, we calculated the difference in benefits by subtracting the O_3_ reduction benefit derived from AVOC control from the benefit derived from NO*x* control. The resulting value, denoted as ΔMDA8 O_3_(AVOCs) − ΔMDA8 O_3_(NO*x*), represents the net advantage—the bonus—of prioritizing an equivalent reduction in AVOCs over NO*x*. This final time series constitutes the data presented in Fig. 5 of the main text.

Therefore, in this study, we emphasize that the shift in regional summer O_3_ formation regimes—and the definition of the AVOCs reduction bonus window—is quantified based exclusively on the relative mitigation efficacy of equivalent proportional reductions in precursors on simulated MDA8 O_3_ concentrations within paired emission scenarios. Crucially, this quantification relies entirely on the direct response of surface O_3_ concentrations (ΔO_3_), thereby not only circumventing the uncertainties associated with absolute FNR baseline values, but also intrinsically mitigating systematic model biases—such as those originating from underlying chemical mechanisms or baseline emission inventories—as these systemic errors largely cancel out through the differential analysis of paired scenarios.

### Data acquisition and preprocessing

The comprehensive methodologies used in this study, including data acquisition and processing for ground-based observations (O_3_, NO_2_, VOCs, meteorology), satellite-derived products (OMI, TROPOMI HCHO and NO_2_), the deweathering procedure employing generalized boosted models (GBMs), configurations for the observation-based box model (OBM), and the setup and validation of the WRF-CAMx model, are detailed in the Supplementary Information (Supplementary Notes 2–6; Supplementary Tables 1–13 and Supplementary Figs. 8–12). Briefly, nationwide hourly O_3_ and NO_2_ concentration data for Chinese cities from 2013-2024 were obtained from the National Urban Air Quality Real-Time Publishing Platform (https://air.cnemc.cn:18007/, last accessed: March 1, 2025) of the China Ministry of Ecology and Environment. The meteorological parameter data (temperature, pressure, wind speed, and precipitation) at the same air quality monitoring stations during the same period were obtained from the China National Environmental Monitoring Center. Continuous hourly concentrations of 57 speciated VOCs for the summers (June–August) of 2019–2021 in seven representative cities (Beijing, Tianjin, Xiong’an, Jinan, Zhengzhou, Shanghai and Guangzhou) were sourced from the CNEMC, the Shanghai Academy of Environmental Sciences and Guangzhou Ecological and Environmental Monitoring Center, respectively. All instrument operation, maintenance, data assurance, and quality control procedures conformed to established Chinese environmental protection standards^48,49^. To ensure data quality and minimize uncertainties, HCHO and NO_2_ retrievals from both OMI and TROPOMI were filtered and subjected to quality assurance detailed in Supplementary Note 3.

## Supplementary information


Supplementary Information
Transparent Peer Review file


## Data Availability

The data supporting the findings of this study are available in Figshare at https://doi.org/10.6084/m9.figshare.29753249^50^. The surface measurements for O_3_ from China’s Ministry of Ecology and Environment can be downloaded from https://air.cnemc.cn:18007/. Emission data were obtained from the Multi-resolution Emission Inventory for China (MEIC): http://www.meicmodel.org. The Dynamic Projection model for Emissions in China (DPEC) was also obtained from http://www.meicmodel.org. Source data are provided with this paper.

## Code availability

The main computational models and software packages used in this study are publicly available third-party tools. Deweathering of O_3_ concentrations was performed using the third-party deweather R package, which is publicly available on GitHub (https://github.com/davidcarslaw/deweather). This package utilizes the gbm (Generalized Boosted Models) R package, available from the Comprehensive R Archive Network (CRAN) at https://CRAN.R-project.org/package=gbm. The simulations were conducted using the Weather Research and Forecasting (WRF) model (v3.9.1) and the Comprehensive Air Quality Model with extensions (CAMx) (v7.10). Both are community models and are publicly available from their respective official websites: WRF at https://www.mmm.ucar.edu/weather-research-and-forecasting-model and CAMx at https://www.camx.com. Biogenic emissions were calculated using the Model of Emissions of Gases and Aerosols from Nature (MEGAN v3.1), available at https://bai.ess.uci.edu/megan. The emissions processing was handled by the Sparse Matrix Operator Kernel Emissions (SMOKE) modeling system, available at https://www.cmascenter.org/smoke/. The Master Chemical Mechanism (MCM) is publicly accessible via the MCM website at http://mcm.leeds.ac.uk/MCM. Details of the Dynamic Projection model for Emissions in China (DPEC) are available at http://www.meicmodel.org. No custom codes or mathematical algorithms that require a separate DOI deposition were generated in this study.
