# Supplementary material for: Aligning climate and air quality goals demands early VOC control for China’s ozone pollution
Source: Nat Commun. 2026 Jul 9;17:9307. doi: 10.1038/s41467-026-75387-w (PMC13530134; doi:10.1038/s41467-026-75387-w)
Supplement: Supplementary file 1 [file 41467_2026_75387_MOESM1_ESM.pdf]

Supplementary Information for

Aligning climate and air quality goals demands early VOC control for China's ozone pollution

Yujie Zhang[1], Jian Gao[1*], Rui Gao[1], Xiaohui Du[1], Chao Yu[2], Xinlu Zhang[1], Yuqiang Zhang[3], Yuhong Liu[3], Yujing Mu[4], Liang Wen[1], Guannan Geng[5], Abdelwahid Mellouki[6], Fahe Chai[1], Hartmut Herrmann[7], Yujiao Zhu[3], Fang Bi[1], Yan Yang[1], Shuai Wang[8], Qian Wang[9], Jinpu Zhang[10,11], Hong Li[1], Hongli Wang[9], Jinhua Tao[2], Yixin Han[12], Mei He[12], Shijie Liu[1], Zhaoqi Gao[3], Bin Luo[3], Haisheng Li[1*], Likun Xue[3*]

[1]State Key Laboratory of Environmental Criteria and Risk Assessment, Chinese Research Academy of Environmental Sciences, Beijing 100012, China

[2]State Key Laboratory of Remote Sensing Science and Digital Earth, Aerospace Information Research Institute, Chinese Academy of Sciences, Beijing 100101, China

[3]Environment Research Institute, Shandong University, Qingdao 266237, China

[4]Research Center for Eco-Environmental Sciences, Chinese Academy of Sciences, Beijing 100085, China

[5]State Key Laboratory of Regional Environment and Sustainability, School of Environment, Tsinghua University, Beijing 100084, China

[6]University Mohammed VI Polytechnic (UM6P), Ben Guerir, Rehamna 43150, Morocco.

[7]Atmospheric Chemistry Department (ACD), Leibniz Institute for Tropospheric Research (TROPOS), Permoserstraße 15, Leipzig 04318, Germany

[8]China National Environmental Monitoring Centre, Beijing 100012, China

[9]State Environmental Protection Key Laboratory of Formation and Prevention of Urban Air Pollution Complex, Shanghai Academy of Environmental Sciences, Shanghai 200233, China

[10]Guangzhou Sub-branch of Guangdong Ecological and Environmental Monitoring Center, Guangzhou 510006, China

[11]State Key Laboratory of Organic Geochemistry, Guangdong Key Laboratory of Environmental Protection and Resources Utilization, Guangzhou Institute of Geochemistry, Chinese Academy of Sciences, Guangzhou 510640, China

[12]Hubei Key Laboratory of Petroleum Geochemistry and Environment, Yangtze University, Wuhan 430100, China

*Corresponding authors

Jian Gao

E-mail: gaojian@craes.org.cn

Haisheng Li

E-mail: lihs@craes.org.cn

Likun Xue

E-mail: xuelikun@sdu.edu.cn

This file contains 7 supplementary notes, 19 supplementary figures, 13 supplementary tables, and supplementary references.

Supplementary Items List

Supplementary Notes

Supplementary Note 1 Inconsistent conclusions on the summer regional O_3 sensitivity 7

Supplementary Note 2 De-weathering of O_3 concentrations using generalized boosted models 18

Supplementary Note 3 Satellite-derived O_3 formation sensitivity trends using NO_2 and HCHO column densities 20

Supplementary Note 4 WRF-CAMx model configuration and performance evaluation 29

Supplementary Note 5 Observation-based model (OBM) simulations 55

Supplementary Note 6 Ground-based data and quality control 57

Supplementary Note 7 Software, map sources and satellite data attribution........60

Supplementary Figures

Supplementary Figure 1. Summer O_3 formation sensitivity trends over the North China Plain (NCP). 9

Supplementary Figure 2. Summer O_3 formation sensitivity trends over the Yangtze River Delta (YRD). 10

Supplementary Figure 3. Summer O_3 formation sensitivity trends over the Pearl River Delta (PRD) 11

Supplementary Figure 4. Consistent monthly variations in column formaldehyde-to-nitrogen dioxide ratio (FNR) derived from CAMx simulations and TROPOMI observations across representative cities during 2020 summer. 22

Supplementary Figure 5. Interannual variation of summer TROPOMI satellite-based

formaldehyde-to-nitrogen dioxide ratios (FNR) derived from two processing pathways in the three megacity clusters of China during 2018–2024. 23
Supplementary Figure 6. Visual comparison of OMI and TROPOMI HCHO column densities illustrating OMI sensor aging. 25
Supplementary Figure 7. Schematic diagram of the master grid and monitoring networks. 30
Supplementary Figure 8. Spatial distribution of monitored versus simulated maximum daily 8 h average (MDA8) O_3 and daily NO_2 concentrations in the North China Plain (NCP) region during the summer of 2020. 33
Supplementary Figure 9. Spatial distribution of monitored versus simulated daily maximum daily 8 h average (MDA8) O_3 and daily NO_2 concentrations in the Yangtze River Delta (YRD) region during the summer of 2020. 34
Supplementary Figure 10. Spatial distribution of monitored versus simulated maximum daily 8 h average (MDA8) O_3 and daily NO_2 concentrations in the Pearl River Delta (PRD) region during the summer of 2020. 34
Supplementary Figure 11. Comparison of monitored and simulated daily maximum daily 8 h average (MDA8) O_3 concentrations for the three megacity clusters during the summer of 2020. 35
Supplementary Figure 12. Comparison of monitored and simulated daily NO_2 concentrations for the three megacity clusters during the summer of 2020. 36
Supplementary Figure 13. Path dependency in O_3 mitigation revealed by 2020–2060 model simulations. 40
Supplementary Figure 14. Derivation of isolated maximum daily 8 h average (MDA8) O_3 reduction benefits from AVOC versus equivalent proportional NO_x reduction. ..41
Supplementary Figure 15. Formaldehyde-to-nitrogen dioxide ratio (FNR) trajectory of

different megacity clusters under different NO*x* to anthropogenic VOC (AVOC) reduction ratios. 43

Supplementary Figure 16. The maximum daily 8 h average (MDA8) O_3 concentrations trajectory of different megacity clusters under different NO*x* to anthropogenic VOC (AVOC) reduction ratios. 45

Supplementary Figure 17. Spatial distribution of summer maximum daily 8 h average (MDA8) O_3 reduction over the North China Plain (NCP) relative to 2020 levels 47

Supplementary Figure 18. Spatial distribution of summer maximum daily 8 h average (MDA8) O_3 reduction over the Yangtze River Delta (YRD) relative to 2020 levels. . 49

Supplementary Figure 19. Spatial distribution of summer maximum daily 8 h average (MDA8) O_3 reduction over the Pearl River Delta (PRD) relative to 2020 levels 51

Supplementary Tables

Supplementary Table 1. Annual average area fractions of ozone formation regimes in the North China Plain (NCP) region during summers of 2005–2024. 12

Supplementary Table 2. Annual average area fractions of ozone formation regimes in the North China Plain (NCP) region during summers of 2018–2024. 13

Supplementary Table 3. Annual average area fractions of ozone formation regimes in the Yangtze River Delta (YRD) during summers of 2005–2024. 14

Supplementary Table 4. Annual average area fractions of ozone formation regimes in the Yangtze River Delta (YRD) region during summers of 2018–2024. 15

Supplementary Table 5. Annual average area fractions of ozone formation regimes in the Pearl River Delta (PRD) region during summers of 2005–2024. 16

Supplementary Table 6. Annual average area fractions of ozone formation regimes in the Pearl River Delta (PRD) region during summers of 2018–2024. 17

Supplementary Table 7. Comparison of the annual average area fractions of ozone formation regimes in the three megacity clusters during summers of 2019–2021 derived from WRF-CAMx model and TROPOMI formaldehyde-to-NO_2 ratios (FNR)..17

Supplementary Table 8. Mean Gradient Boosting Machine (GBM) model performance statistics for the testing set in each megacity cluster. 19

Supplementary Table 9. Model performance statistics for O_3 and NO_2 in China from June to August 2020. 38

Supplementary Table 10. Model performance statistics for meteorological data in China from June to August 2020. 38

Supplementary Table 11. The reduction proportions of NO*x* to anthropogenic VOCs (AVOCs) from 2020 to 2060 under three different scenarios in the North China Plain (NCP) megacity cluster. 52

Supplementary Table 12. The reduction proportions of NO*x* to anthropogenic VOCs (AVOCs) from 2020 to 2060 under three3 different scenarios in the Yangtze River Delta (YRD) megacity cluster. 53

Supplementary Table 13. The reduction proportions of NO*x* to anthropogenic VOCs (AVOCs) from 2020 to 2060 under three different scenarios in the Pearl River Delta (PRD) megacity cluster. 54

Supplementary References..61

Supplementary Notes

Supplementary Note 1 Inconsistent conclusions on the summer regional O_3 sensitivity

Various methods, including satellite-derived formaldehyde-to-NO_2 ratio (HCHO/NO_2, FNR), source-oriented chemical transport models (CTM), and ground-observation-based box models (e.g., OBM), have been widely applied to investigate regional O_3 formation sensitivity[1-10]. However, each of these approaches possesses limitations that can lead to inconsistencies in their findings.

OBM, for instance, offers accurate in situ diagnoses of O_3 sensitivity at specific locations and times. Nevertheless, their application is limited in spatial and temporal extent due to the stringent requirement for comprehensive, simultaneous hourly observational data for key species (e.g., O_3, NO_x, CO, VOCs) and related parameters (e.g., JNO_2, temperature, humidity, pressure), which are hardly available over large domains or long periods[2,6]. CTMs provide descriptions across broader spatial and temporal scales. However, uncertainties primarily come from anthropogenic and biogenic emission inventories, as well as the representation of photochemical reaction mechanisms[3,6]. The satellite-based method can provide globalscale photochemical information with good time continuity and little human disturbance. Yet, their reliance on FNR thresholds to distinguish O_3 formation regimes introduces a crucial challenge. The satellite thresholds were reported to vary with the different regions, periods, and O_3 pollution severity, even using the same determination methods. And these different thresholds yield different sensitivity classifications for the same spatial and temporal conditions[4-10].

Despite the valuable insights from prior studies, the inconsistent conclusions on the identification of the summer regional sensitivity of the three megacity clusters in China due to the limitation of different methods, hinder to some extent the identification of the most effective strategy. Illustrative examples of these discrepancies include: Ren et al.[6], using satellite-derived FNR, reported prevalent VOC-limited regimes across all three megacity clusters primarily during the O_3 pollution period (predominantly from May to October), whereas Li et al.[9] concluded that the Yangtze River Delta (YRD) and the Pearl River Delta (PRD) were predominantly NO_x-limited, while the North China Plain (NCP) remained VOC-limited. Conversely, Du et al.[3] suggested a predominant shift from VOC-limited towards transitional regimes in all three clusters during summer by CTM. Furthermore, Zhang et al.[2] employed the OBM approach and found that over 90% of urban areas in the NCP located in either VOC-limited or transitional regimes during the warm season (June–September). Beyond inconsistencies in the dominant regime identification, the spatial distributions and relative fractions of different sensitivity regimes derived by different methods (e.g., CTM versus satellite FNR thresholds) or even by the same satellite method using different thresholds also exhibit notable disparities. These inconsistencies in spatial mapping of different sensitivity regimes are also explicitly demonstrated by our own analyses (Fig. 2, Supplementary Figures 1-3, and Supplementary Tables 1-7).

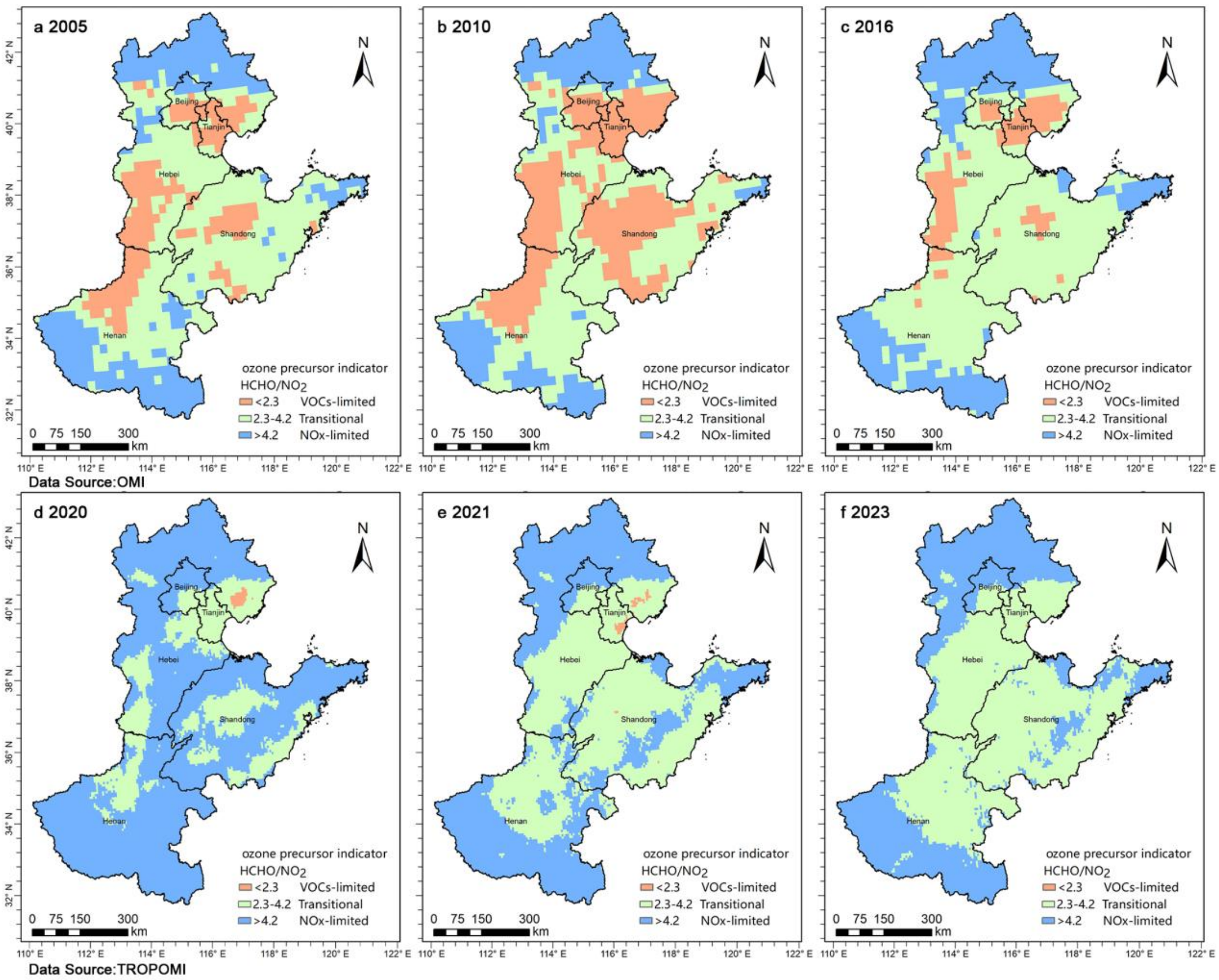

Supplementary Figure 1. Summer O_3 formation sensitivity trends over the North China Plain (NCP). a-f O_3 formation regimes for the different representative years. The maps illustrate the O_3 formation regime derived from satellite formaldehyde-to-nitrogen dioxide ratio (FNR, $HCHO/NO_2$) data for representative years from 2005 to 2023. A uniform FNR threshold of [2.3, 4.2] was adopted. Administrative boundaries are based on the Standard Map Service of the Ministry of Natural Resources of the People's Republic of China (http://bzdt.ch.mnr.gov.cn/), with map approval number GS(2024)0650.

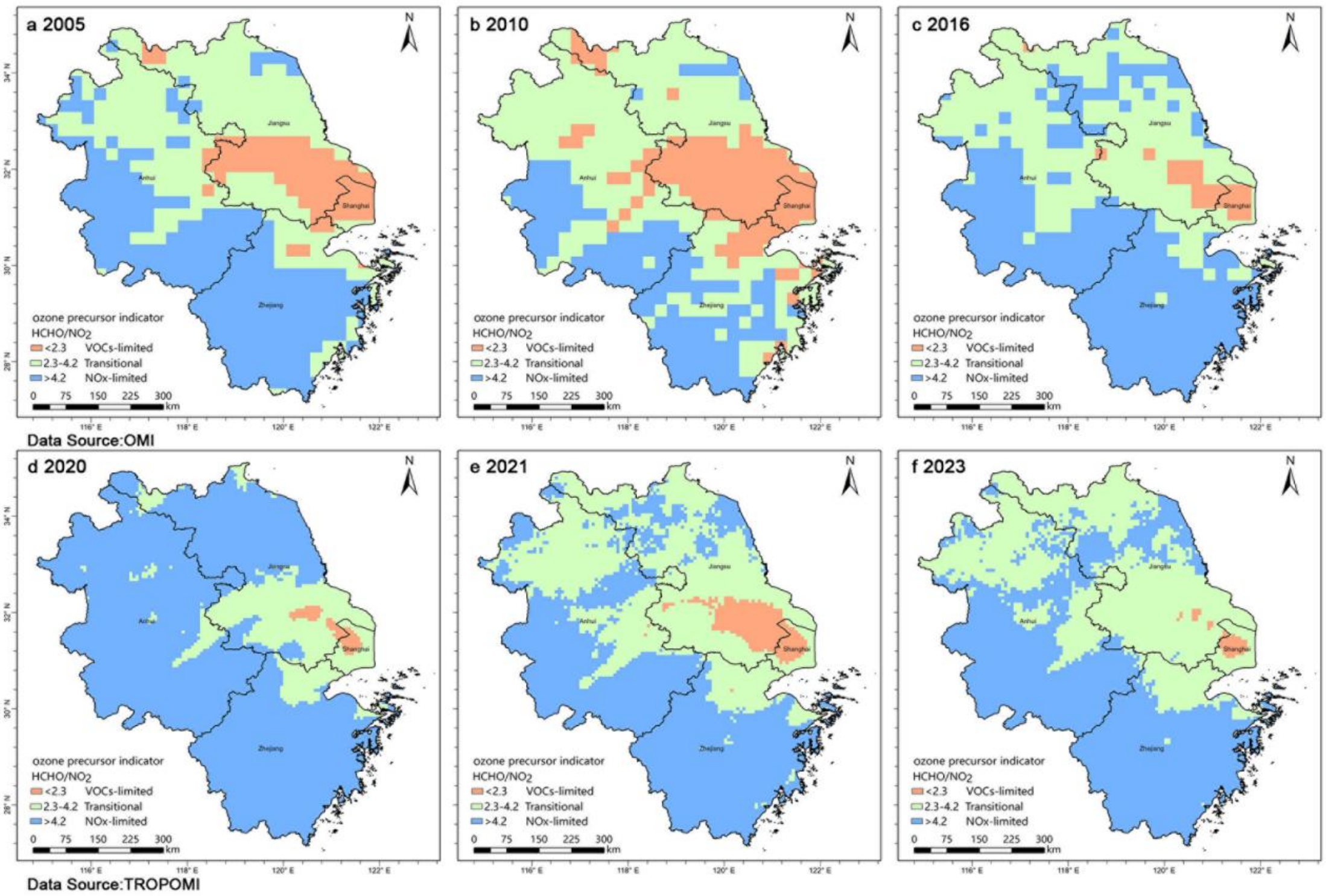

Supplementary Figure 2. Summer O_3 formation sensitivity trends over the Yangtze River Delta (YRD). a-f O_3 formation regimes for the different representative years. The maps illustrate the O_3 formation regime derived from satellite formaldehyde-to-nitrogen dioxide ratio (FNR, $HCHO/NO_2$) data for representative years from 2005 to 2023. A uniform FNR threshold of [2.3, 4.2] was adopted. Administrative boundaries are based on the Standard Map Service of the Ministry of Natural Resources of the People's Republic of China (http://bzdt.ch.mnr.gov.cn/), with map approval number GS(2024)0650.

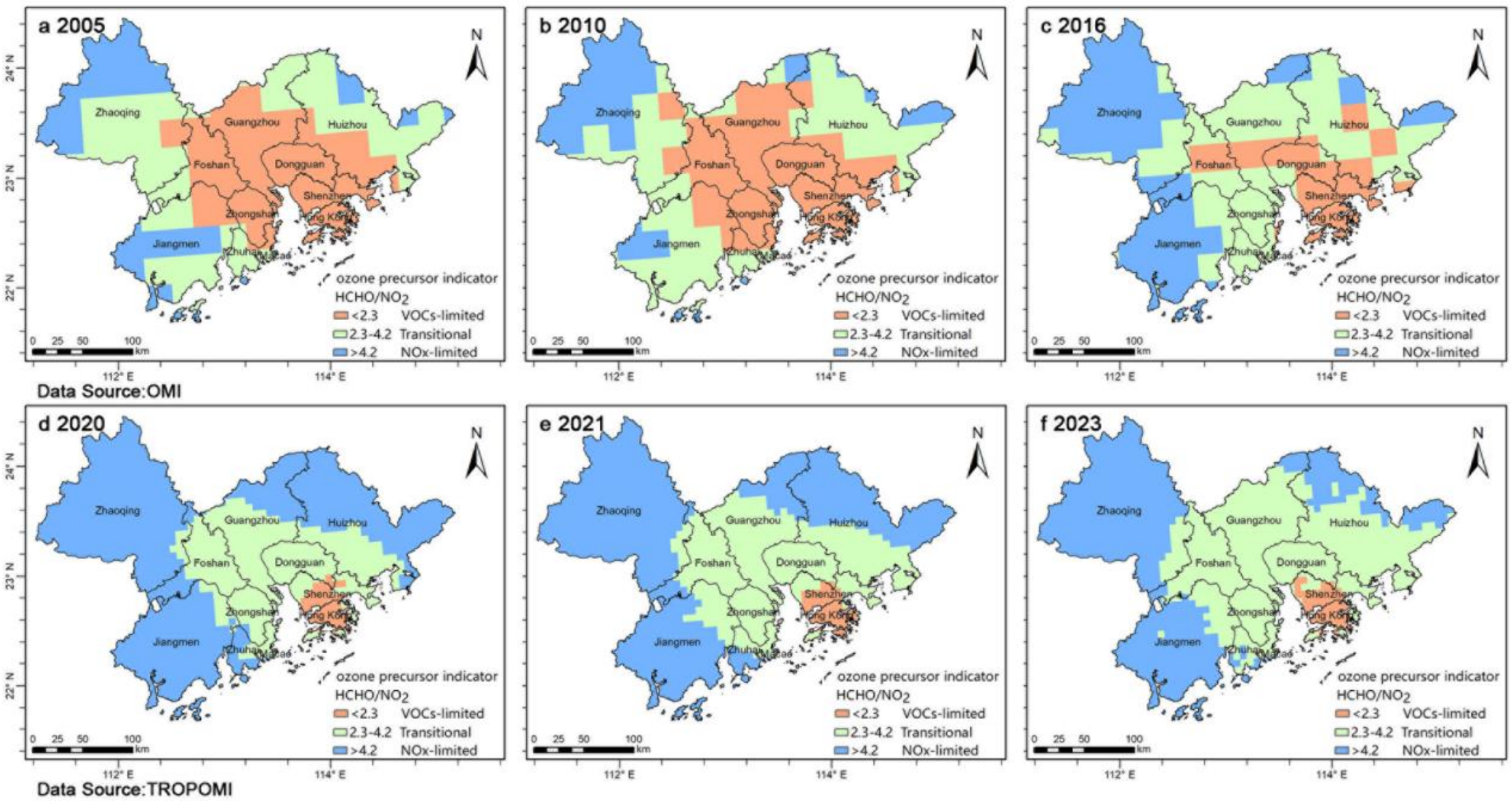

Supplementary Figure 3. Summer O_3 formation sensitivity trends over the Pearl River Delta (PRD). a-f O_3 formation regimes for the different representative years. The maps illustrate the O_3 formation regime derived from satellite formaldehyde-to-nitrogen dioxide ratio (FNR, $HCHO/NO_2$) data for representative years from 2005 to 2023. A uniform FNR threshold of [2.3, 4.2] was adopted. Administrative boundaries are based on the Standard Map Service of the Ministry of Natural Resources of the People's Republic of China (http://bzdt.ch.mnr.gov.cn/), with map approval number GS(2024)0650.

Supplementary Table 1. Annual average area fractions of ozone formation regimes in the North China Plain (NCP) region during summers of 2005–2024. A uniform formaldehyde-to-NO_2 ratios (FNR) transition regime thresholds of [2.3,4.2] is applied.

NCP-OMI	VOC-limited regime （%）	Transitional regime （%）	NO*x*- limited regime （%）
2005	20.09	49.88	30.02
2006	29.10	48.15	22.75
2007	26.21	46.65	27.14
2008	30.83	48.38	20.79
2009	29.79	47.69	22.52
2010	35.10	42.38	22.52
2011	33.26	49.08	17.67
2012	32.45	47.23	20.32
2013	23.56	45.38	31.06
2014	33.37	43.42	23.21
2015	19.63	59.47	20.90
2016	11.32	58.55	30.14
2017	9.01	61.09	29.91
2018	3.00	60.28	36.72
2019	2.89	60.39	36.72
2020	1.27	51.73	**47.00**
NCP-TROPOMI	VOC-limited regime （%）	Transitional regime （%）	NO*x*- limited regime （%）
2018	2.40	42.71	54.89
2019	1.25	28.91	69.84
2020	0.89	24.48	**74.63**
2021	1.51	42.13	56.36
2022	0.39	44.82	54.79
2023	0.16	53.12	46.67
2024	0.02	25.08	74.70

Supplementary Table 2. Annual average area fractions of ozone formation regimes in the North China Plain (NCP) region during summers of 2018–2024. A regional-oriented uniform formaldehyde-to-NO_2 ratios (FNR) transition regime thresholds of [3.5, 5.1] is applied.

NCP-TROPOMI	VOC-limited regime (%)	Transitional regime (%)	NO*x*- limited regime (%)
2018	19.15	51.26	29.59
2019	11.08	52.07	36.85
2020	9.32	47.79	**42.89**
2021	19.15	47.33	33.52
2022	16.37	53.11	30.52
2023	15.66	59.62	24.71
2024	0.04	58.67	36.87

Supplementary Table 3. Annual average area fractions of ozone formation regimes in the Yangtze River Delta (YRD) during summers of 2005–2024. A uniform formaldehyde-to-NO_2 ratios (FNR) transition regime thresholds of [2.3,4.2] is applied.

YRD-OMI	VOC-limited regime （%）	Transitional regime （%）	NO*x*- limited regime （%）
2005	12.92	43.54	43.54
2006	15.87	46.31	37.82
2007	16.97	45.39	37.64
2008	19.19	47.60	33.21
2009	16.79	45.39	37.82
2010	21.59	50.92	27.49
2011	19.00	48.71	32.29
2012	18.27	50.37	31.37
2013	8.86	40.96	50.18
2014	20.11	54.24	25.65
2015	12.92	54.06	33.03
2016	3.87	44.28	51.85
2017	7.56	50.00	42.44
2018	5.35	47.79	46.86
2019	5.17	50.92	43.91
2020	2.03	41.51	**56.46**
YRD-TROPOMI	VOC-limited regime （%）	Transitional regime （%）	NO*x*- limited regime （%）
2018	3.73	31.54	64.73
2019	3.48	21.21	75.31
2020	2.33	16.87	**80.79**
2021	6.03	35.90	58.07
2022	0.79	22.19	77.02
2023	1.94	37.87	60.13
2024	0.49	14.01	85.45

Supplementary Table 4. Annual average area fractions of ozone formation regimes in the Yangtze River Delta (YRD) region during summers of 2018–2024. A regional-oriented uniform formaldehyde-to-NO_2 ratios (FNR) transition regime thresholds of [2.0, 4.0] is applied.

YRD-TROPOMI	VOC-limited regime （%）	Transitional regime （%）	NO*x*- limited regime （%）
2018	1.76	27.91	70.33
2019	2.26	18.02	79.72
2020	1.25	15.08	**83.67**
2021	3.69	31.89	64.41
2022	0.27	17.34	82.40
2023	0.80	31.15	68.05
2024	0.21	10.98	88.76

Supplementary Table 5. Annual average area fractions of ozone formation regimes in the Pearl River Delta (PRD) region during summers of 2005–2024. A uniform formaldehyde-to-NO_2 ratios (FNR) transition regime thresholds of [2.3,4.2] is applied.

PRD-OMI	VOC-limited regime（%）	Transitional regime（%）	NO*x*- limited regime（%）
2005	37.50	41.25	21.25
2006	37.50	33.75	28.75
2007	36.25	38.75	25.00
2008	40.00	38.75	21.25
2009	28.75	35.00	36.25
2010	40.00	37.50	22.50
2011	28.75	45.00	26.25
2012	26.25	45.00	28.75
2013	26.25	48.75	25.00
2014	26.25	43.75	30.00
2015	18.75	37.50	43.75
2016	17.50	42.50	40.00
2017	21.25	50.00	28.75
2018	1.25	41.25	**57.50**
2019	6.25	42.50	51.25
2020	8.75	41.25	50.00
PRD-TROPOMI	VOC-limited regime（%）	Transitional regime（%）	NO*x*- limited regime（%）
2018	8.48	28.63	62.89
2019	4.03	25.36	**70.61**
2020	4.79	27.88	67.34
2021	5.96	29.05	64.99
2022	4.95	31.49	63.56
2023	5.79	36.78	57.93
2024	3.02	29.14	68.36

Supplementary Table 6. Annual average area fractions of ozone formation regimes in the Pearl River Delta (PRD) region during summers of 2018–2024. A regional-oriented uniform formaldehyde-to-NO_2 ratios (FNR) transition regime thresholds of [1.7, 3.5] is applied.

PRD-TROPOMI	VOC-limited regime (%)	Transitional regime (%)	NO*x*- limited regime (%)
2018	2.18	25.69	72.12
2019	0.42	22.08	**77.50**
2020	1.09	23.09	75.82
2021	1.76	23.76	74.48
2022	1.60	24.10	74.31
2023	1.43	25.69	73.38
2024	0.42	18.64	81.44

Supplementary Table 7. Comparison of the annual average area fractions of ozone formation regimes in the three megacity clusters during summers of 2019–2021 derived from WRF-CAMx model and TROPOMI formaldehyde-to-NO_2 ratios (FNR). The FNR thresholds of transition regime are [2.3,4.2]. The three megacity clusters are as follows: the North China Plain (NCP), the Yangtze River Delta (YRD) and the Pearl River Delta (PRD).

CAMx	VOC-limited			Transitional			NOx-limited		
	NCP	YRD	PRD	NCP	YRD	PRD	NCP	YRD	PRD
2019	8.4	0.4	1.6	65.0	32.6	13.1	26.5	67.0	85.2
2020	7.5	2.9	9.8	68.4	45.9	23.0	24.1	51.3	67.2
2021	19.2	2.5	6.6	58.6	43.4	18.0	22.1	54.1	75.4

TROPOMI	VOC-limited			Transitional			NOx-limited		
	NCP	YRD	PRD	NCP	YRD	PRD	NCP	YRD	PRD
2019	1.2	3.5	4.0	28.9	21.2	25.4	69.8	75.3	70.6
2020	0.9	2.3	4.8	24.5	16.9	27.9	74.6	80.8	67.3
2021	1.5	6.0	6.0	42.1	35.9	29.0	56.4	58.1	65.0

Supplementary Note 2 De-weathering of O_3 concentrations using generalized boosted models

To isolate the impact of meteorological variability from O_3 concentrations and thus reveal emission-driven trends, we employed Generalized Boosted Regression Models (GBM). The GBM machine learning algorithm is a meteorological normalisation technique that can be used to control for meteorology over time in air quality time series by reducing variability using statistical modelling[11,12]. The deweather R package was used for this process[13]. It uses a two-step procedure to normalise air quality data. First, models based on the Boosted Regression Tree (BRT) approach for daily concentration of selected air pollutants from different predictors are fitted using the "gbm" package[14]. Second, a meteorological averaging procedure is applied by predicting many times with a random sampling of weather conditions[13], using the "metSim" function.

Models were trained on an 12-year dataset (2013−2024) encompassing daily O_3 concentrations and key meteorological variables known to significantly influence O_3 formation and removal processes. These predictors included maximum daily temperature, minimum daily relative humidity, average 2-minute wind speed, total daily precipitation, average atmospheric pressure, and month. The meteorological and pollutant measurements collected over the entire period were randomly partitioned into an 80% subset for training the BRT models and a 20% subset for evaluating their performance. Model skill was automatically assessed using standard metrics: Pearson's correlation coefficient (r), root mean square error (RMSE), and mean bias (MB). High r values (approaching 1) and low RMSE and MB values (approaching 0) indicate strong predictive performance and minimal bias, respectively.

The model training was performed on a region basis, utilizing a forest composed of 500 trees, with bootstrap simulations for partial dependence plots of 100. The number of features that may split at each node was set as the square root of the number of all features, similar to the previous literature[14]. Five hundred predictions were used to calculate the meteorologically normalised trend. Our model exhibited robust performance in the O_3 concentration prediction in all the three megacity clusters (Supplementary Table 8). These statistics specifically reflect the model's performance on the 20% testing subset, demonstrating its strong generalization capability and absence of severe overfitting. The high R values (ranging from 0.78 to 0.83 for the mean) and low RMSE/MB values for the testing set confirm the model's robustness in capturing emission-driven O_3 trends while accounting for meteorological variability. The ensemble nature of the GBM algorithm, which combines multiple weak learners, inherently mitigates the risk of overfitting by aggregating diverse predictions.

Supplementary Table 8. Mean Gradient Boosting Machine (GBM) model performance statistics for the testing set in each megacity cluster. Values are presented as mean (minimum, maximum) over the 12-year analysis period (2013-2024). R denotes Pearson's correlation coefficient, RMSE is Root Mean Square Error (μg m^{-3}), and MB is Mean Bias (μg m^{-3}). The three megacity clusters are as follows: the North China Plain (NCP), the Yangtze River Delta (YRD) and the Pearl River Delta (PRD).

	R	RMSE	MB
NCP	0.83(0.66, 0.9)	24.31(19.58, 31.25)	-0.06(-3.95, 4.53)
YRD	0.82(0.72, 0.9)	23.58(14.39, 30.29)	0.37(-2.22, 5.47)
PRD	0.78(0.67, 0.87)	24.62(17.39, 34.64)	-0.22(-2.73, 5.26)

Supplementary Note 3 Satellite-DERIVED O_3 formation sensitivity trends using NO_2 and HCHO column densities

Satellite NO_2 and HCHO data products. Tropospheric vertical column densities (VCDs) of nitrogen dioxide (NO_2) and formaldehyde (HCHO) were retrieved from two satellite instruments: the Ozone Monitoring Instrument (OMI) and the Tropospheric Monitoring Instrument (TROPOMI). OMI provides observations with a spatial resolution of 24 × 13 km^2, while TROPOMI offers enhanced resolution at 7 × 7 km^2, facilitating detailed spatial characterization of atmospheric parameters. Both instruments provide daily global coverage with overpass times (approximately 13:30–13:50 local time) that coincide with peak O_3 photochemical production. At this time, the planetary boundary layer is generally elevated and the solar zenith angle is small, which maximizes instrumental sensitivity to HCHO and NO_2 in the lower troposphere[4].

OMI HCHO and NO_2 products spanning 2005–2020 were developed under the Quality Assurance for Essential Climate Variables (QA4ECV) project (http://www.qa4ecv.eu/; last accessed: 6 May 2021). OMI tropospheric HCHO VCDs were retrieved by the Belgian Institute for Space Aeronomy (BIRA-IASB). Data were filtered using processing_quality_flags = 0 or > 255, which provides a selection of observations that is considered optimal[10]. The QA4ECV HCHO datasets are available with a spatial resolution of 0.05° × 0.05°. We regridded the monthly vertical columns data to 0.25°×0.25° resolution to bring the precision below 1×10^{15} molecules cm^{-2}. NO_2 data were compiled by the Royal Netherlands Meteorological Institute (KNMI) with a spatial resolution of 0.125° × 0.125°. The monthly OMI NO_2 data were also regridded to 0.25° × 0.25° resolution to match the monthly OMI HCHO data. For the post-2020 OMI HCHO data used only for the sensor-aging comparison were obtained

from NASA Earthdata (https://www.earthdata.nasa.gov/data/).

Tropospheric NO_2 and HCHO vertical column densities (VCDs) spanning the summers of 2018–2024 were sourced from the TROPOspheric Monitoring Instrument (TROPOMI) onboard the Sentinel-5 Precursor (S5P) satellite. To rigorously validate our findings, data were compiled through two distinct processing pathways (Supplementary Figure 4 and Supplementary Figure 5). The first pathway (TROP-ESA) used official Sentinel-5P/TROPOMI Level-2 offline products obtained from the Copernicus Data Space Ecosystem (https://browser.dataspace.copernicus.eu/; accessed: 9 January 2025). Data were filtered using a highly stringent quality assurance (QA) threshold (QA > 0.75) for both NO_2 and HCHO, which provides a selection of observations that is considered optimal by effectively excluding retrievals compromised by heavy cloud cover or aerosol pollution. These filtered L2 orbital data were subsequently regridded onto a uniform 7 × 7 km spatial resolution for regional mapping. Alternatively, the second pathway (TROP-GEE) employed operational Level-3 (L3) offline products accessed via the Google Earth Engine (GEE) cloud computing platform (https://earthengine.google.com/; accessed: 9 January 2025). Within the GEE framework, the original time-binned L2 orbital swaths are converted into L3 spatial grids utilizing the harpconvert tool (via the bin_spatial operation), with antimeridian-crossing orbits systematically split to preserve spatial continuity. Quality filtering for this GEE-derived dataset is standardized, restricting observations to pixels where QA values exceed 0.75 for NO_2 and 0.50 for HCHO. Compared to the highly rigorous filtering applied in the TROP-ESA method, the TROP-GEE approach offers a streamlined and reproducible pipeline; however, we note that the GEE data record is available from 2019 onwards. A side-by-side comparison of the results derived from both data processing pathways is presented in Supplementary Figure 5.

It can be seen that although the absolute FNR values across the three megacity clusters differ slightly from 2019 to 2024 due to divergent filtering criteria, they exhibit concordant interannual variations. This alignment demonstrates that the relative FNR trend—rather than its static absolute value—provides a more robust indicator for diagnosing O_3 sensitivity.

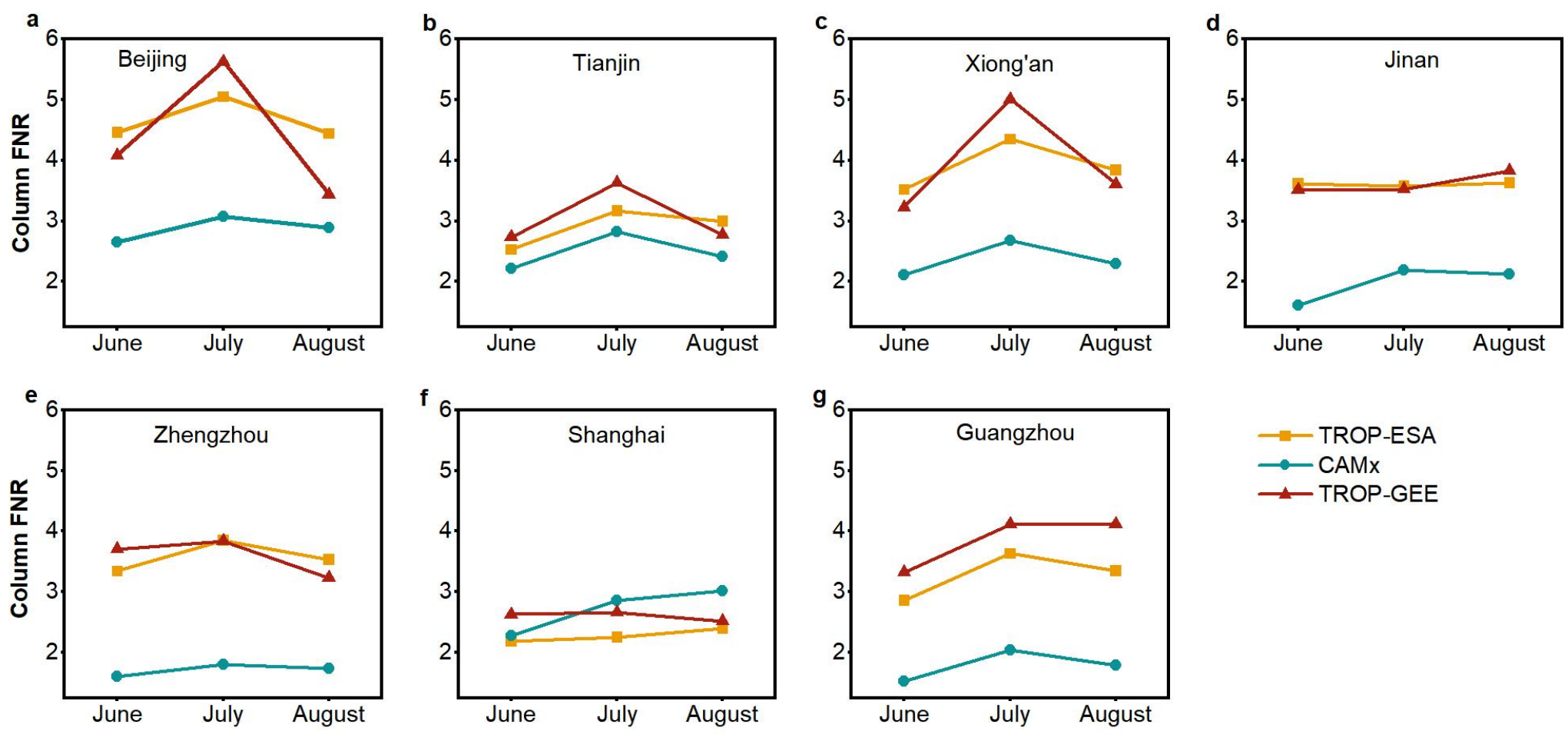

Supplementary Figure 4. Consistent monthly variations in column formaldehyde-to-nitrogen dioxide ratio (FNR) derived from CAMx simulations and TROPOMI observations across representative cities during 2020 summer. **a-g** results for different representative city. The satellite data were processed via two approaches (TROP-ESA and TROP-GEE).

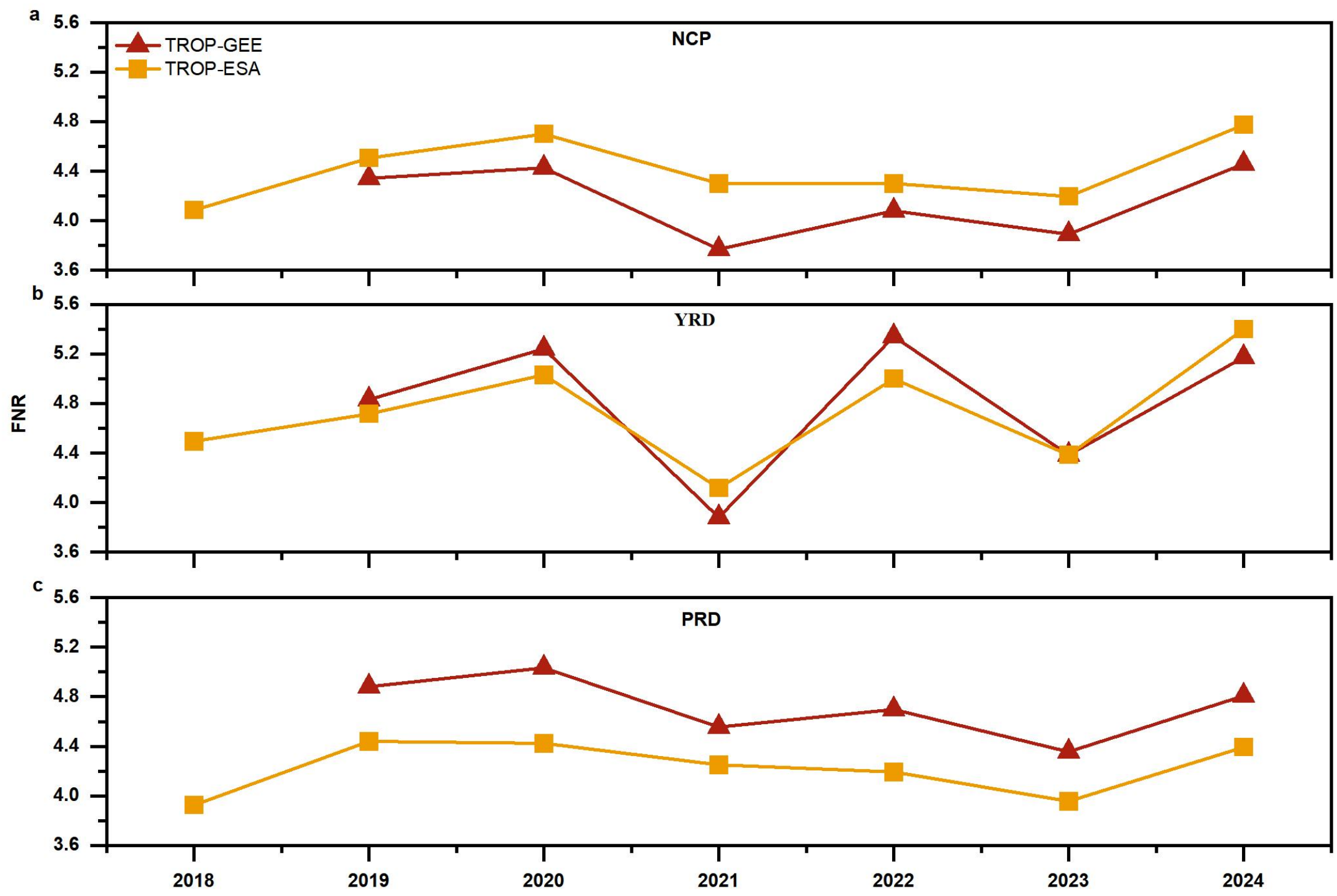

Supplementary Figure 5. Interannual variation of summer TROPOMI satellite-based formaldehyde-to-nitrogen dioxide ratios (FNR) derived from two processing pathways in the three megacity clusters of China during 2018–2024. The three megacity clusters are as follows: **a** the North China Plain (NCP), **b** the Yangtze River Delta (YRD) and **c** the Pearl River Delta (PRD). The TROPOMI data from GEE platform start from 2019.

The transition from OMI to TROPOMI data in 2018 is a deliberate and critical methodological choice driven by stringent data quality control. While the OMI instrument has provided an invaluable long-term record, its performance has degraded after more than a decade of operation. The retrieval of trace gases with weak optical absorption, such as HCHO, necessitates an exceptionally high signal-to-noise ratio. In recent years, particularly post-2020, detector aging has manifested as severe striping noise in OMI HCHO products (Supplementary Figure 6), which critically compromises the usability of the data for precise trend analysis. Consequently, we

transitioned to TROPOMI data for the period of 2018–2024. As the successor to OMI, TROPOMI features significantly improved signal-to-noise ratios and finer spatial resolution (7×7 km^2 versus 13×24 km^2 for OMI). Therefore, to ensure the highest possible accuracy and reliability for our analysis, particularly for resolving the fine-scale dynamics of O_3 sensitivity after 2018, we exclusively use the TROPOMI dataset for the recent period.

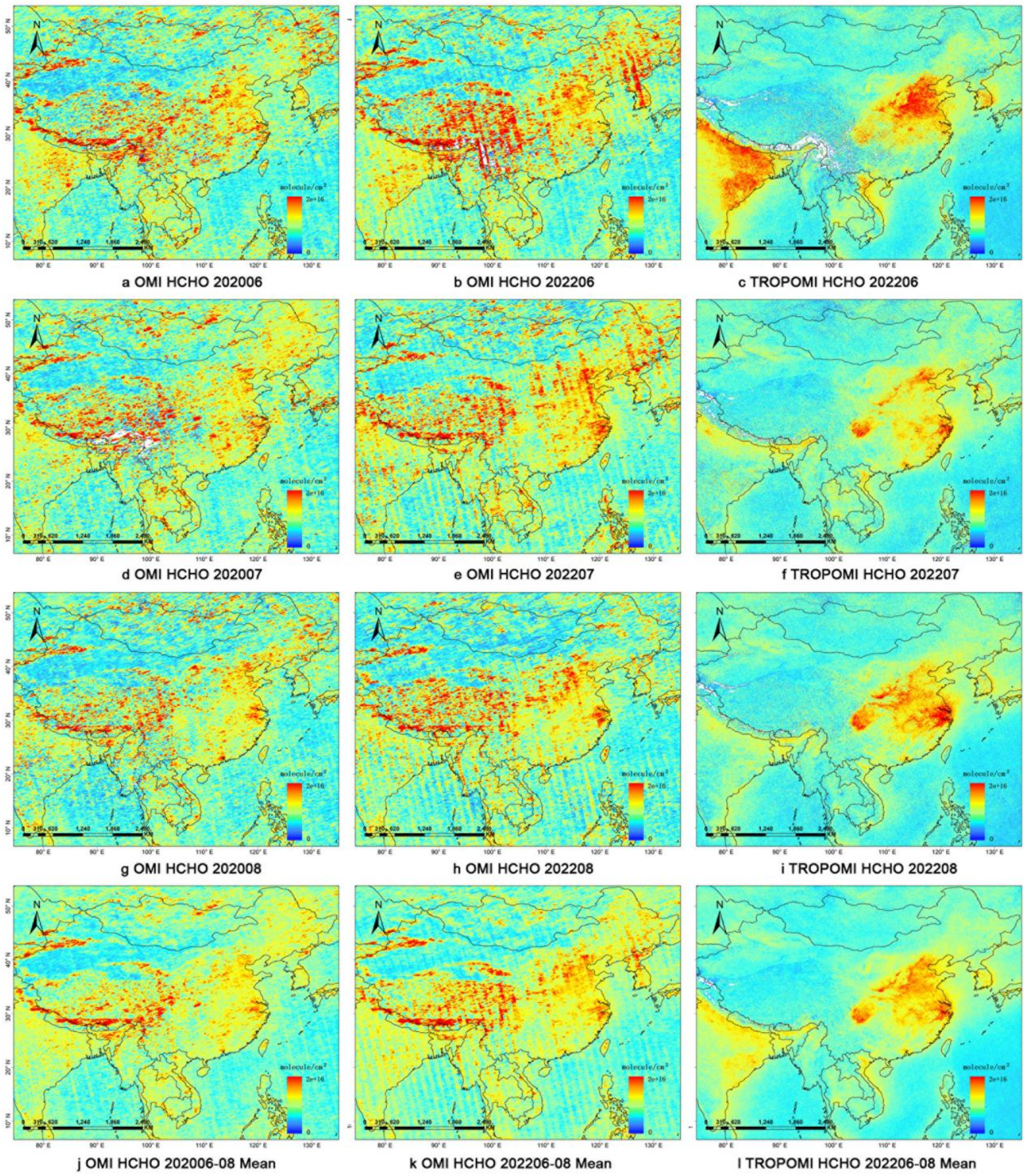

Supplementary Figure 6. Visual comparison of OMI and TROPOMI HCHO column densities illustrating OMI sensor aging. a, d, g, j OMI HCHO column densities for June, July, August, and the summer mean in 2020. **b, e, h, k** OMI HCHO column densities for June, July, August, and the summer mean in 2022. **c, f, i, l** TROPOMI HCHO column densities for June, July, August, and the summer mean in 2022. Distinct striping noise is evident in the OMI data from 2022 compared to the cleaner retrievals from OMI in 2020 and TROPOMI in 2022. Administrative boundaries are based on the Standard Map Service of the Ministry of Natural Resources of the People's Republic of China (http://bzdt.ch.mnr.gov.cn/), with map approval number GS(2024)0650.

Traditional O_3 Formation Sensitivity zoning method using the Satellite-Retrieved HCHO/NO_2 Ratio (FNR). The ratio of HCHO to NO_2 VCDs (FNR) derived from satellite observations is a widely employed diagnostic for O_3 formation sensitivity, offering global-scale photochemical insights with high temporal continuity and minimal anthropogenic disturbance[10]. The classification of O_3 formation regimes (VOC-limited, NOx-limited, or transitional) relies on FNR thresholds that delineate these regimes. Two methodologies exist for determining these thresholds: (1) derivation from chemical transport models (CTM) that explicitly link HCHO/NO_2 ratios to surface O_3 sensitivity[3, 4, 15, 16], and (2) empirical derivation from concurrent ground-based observations using non-linear statistical models [7,9,10]. The first approach[3,4], utilizes a CTM as a "virtual laboratory". First, driven by emission inventories and meteorological fields, the CTM simulates the dynamic evolution of the O_3-NO_x-VOC photochemical system in a three-dimensional domain. Second, through specific numerical techniques, the response of O_3 concentrations to precursor changes was quantified within the model environment to identify the

transitional state between NOx-limited and VOC-limited regimes. Finally, the FNR value simulated by the model at this specific transitional state is extracted and defined as the sensitivity threshold. Therefore, the fundamental commonality of these methods is their intrinsic dependence on the model's representation of chemical mechanisms, the accuracy and completeness of emission inventories, and the parameterization of atmospheric physical processes. The reliability of the derived threshold is entirely contingent on the model's fidelity in reproducing the complex, non-linear relationships of atmospheric chemistry. The latter approach[7,9,10], involves calculating the probability of surface O_3 exceeding a certain concentration threshold (e.g., 70 ppbv) for given satellite-derived FNR values. This is achieved by examining their statistical relationship, typically using a third-order polynomial model. The peak of this polynomial curve is assumed to mark the transition point between VOC-limited and NOx-limited O_3 formation regimes, and the transitional regime is subsequently defined as the range of FNR values spanning the top 10% of this high-O_3 probability distribution. To align with satellite overpass times, ground-based O_3 observations from 13:00 to 14:00 local time were selected, after eliminating invalid daily O_3 monitoring data. The FNR thresholds employed in this study were derived using this observation-based statistical method, as detailed in our prior publications[9,10].

A significant challenge in FNR-based sensitivity assessment is the lack of universal agreement on threshold values. These values depend on the analytical approach used to define O_3-precursor relationships (i.e. derivation from the CTM or ground-based observation) and the specific criteria applied (e.g., the range of FNR values spanning the top 10% of the high-O_3 probability distribution)[6,7,17]. Observationally derived thresholds for the transitional regime often yield higher HCHO/NO_2 values compared to model-derived thresholds. Moreover, even when

employing identical methodologies, threshold values can exhibit regional variability due to differences in meteorological conditions, ambient O_3 levels, and precursor composition[6,8]. This variability contributes to different conclusions in the literature regarding dominant regional sensitivities, the spatial distribution of O_3 formation regimes, and their respective areal fractions under similar spatiotemporal conditions (detailed in Supplementary Note 1)[5-8].

The robustness of sensitivity trends investigation. To avoid the uncertainties associated with the threshold determination, our analysis focused on trends in O_3 formation sensitivity. This was achieved by examining inter-annual variations in the FNR itself and by tracking yearly changes in the areal fractions of different O_3 formation regimes.

Previous studies have demonstrated that the trend in FNR is less sensitive to the choice of satellite product, as long-term multi-year averaging attenuates inter-product differences, and different products generally exhibit high consistency in their long-term spatiotemporal variations[3,18]. The robustness of the FNR trends in this study is corroborated by two key lines of evidence: (1) the consistent month-to-month evolution of column FNR between TROPOMI observations and air quality model simulations across representative cities in the summer of 2020 (Supplementary Figure 4); and (2) the consistent interannual variations in TROPOMI FNR derived via two independent data processing pathways across the three megacity clusters during the summers of 2019 – 2024 (Supplementary Figure 5).

Similarly, while areal fractions may differ, the trend in the areal fraction of a specific sensitivity regime within a given region and season is expected to be robust to the choice of threshold. This premise is supported by literature showing similar evolutionary patterns of regime shifts between years despite the use of different

thresholds derived from various satellite products and criteria[18]. We further validated this premise by: (1) comparing trends in regime area fractions derived from OMI and TROPOMI data (summers 2005–2024) using a uniform FNR threshold, and (2) comparing trends derived from TROPOMI data (summers 2018–2024) using both uniform and region-specific FNR thresholds (Supplementary Tables 1-6). While areal fractions differed across these comparisons, the trends in the variation of areal fractions remained consistent. For instance, the maximum areal fraction of the VOC-limited regime consistently coincided with the first FNR trend inflection point (e.g., ~2010 for NCP and YRD), and the maximum NO*x*-limited regime areal fraction aligned with the second FNR inflection point (e.g., ~2020). Consequently, for simplicity and to focus on robust trend analysis, we applied the uniform FNR threshold values established in our prior work[10] to investigate trends in the areal fractions of different O_3 formation sensitivity regimes across the three megacity clusters during summers in China (Supplementary Figures 1-3). The methodologies for determining these uniform and region-specific thresholds are detailed in our previous studies[9,10].

Additionally, the consistency of the O_3 formation trend during 2019-2021 derived from satellite and two model simulations (WRF-CAMx and OBM) also proves that the application of a uniform threshold across different regions has little impact on the comparison of the sensitivity evolution trend over multiple years in the same region and season (Fig.2 and Supplementary Table 7).

Supplementary Note 4 WRF-CAMx model configuration and performance evaluation

Model overview and domain setup. We employed the coupled Weather Research and Forecasting (WRF) model (v3.9.1) and the Comprehensive Air quality Model with extensions (CAMx) (v7.10) to simulate atmospheric composition, with a focus on O_3 formation sensitivity over China's three key megacity clusters: the NCP, the YRD, and the PRD (Supplementary Figure 7). The selected map projection for the model simulations is the Lambert Conic Conformal (LCC) projection, with a simulated grid resolution of 36 km × 36 km. The vertical structure comprised 20 non-uniform layers, extending up to approximately 20 km. Boundary conditions for the chemical simulations were provided by the Model for Ozone and Related chemical Tracers (MOZART). The gas-phase chemistry was simulated with the Statewide Air Pollution Research Center (SAPRC07TC) mechanism. Photolysis rates were calculated based on Ozone Monitoring Instrument (OMI) observations of O_3 column concentrations.

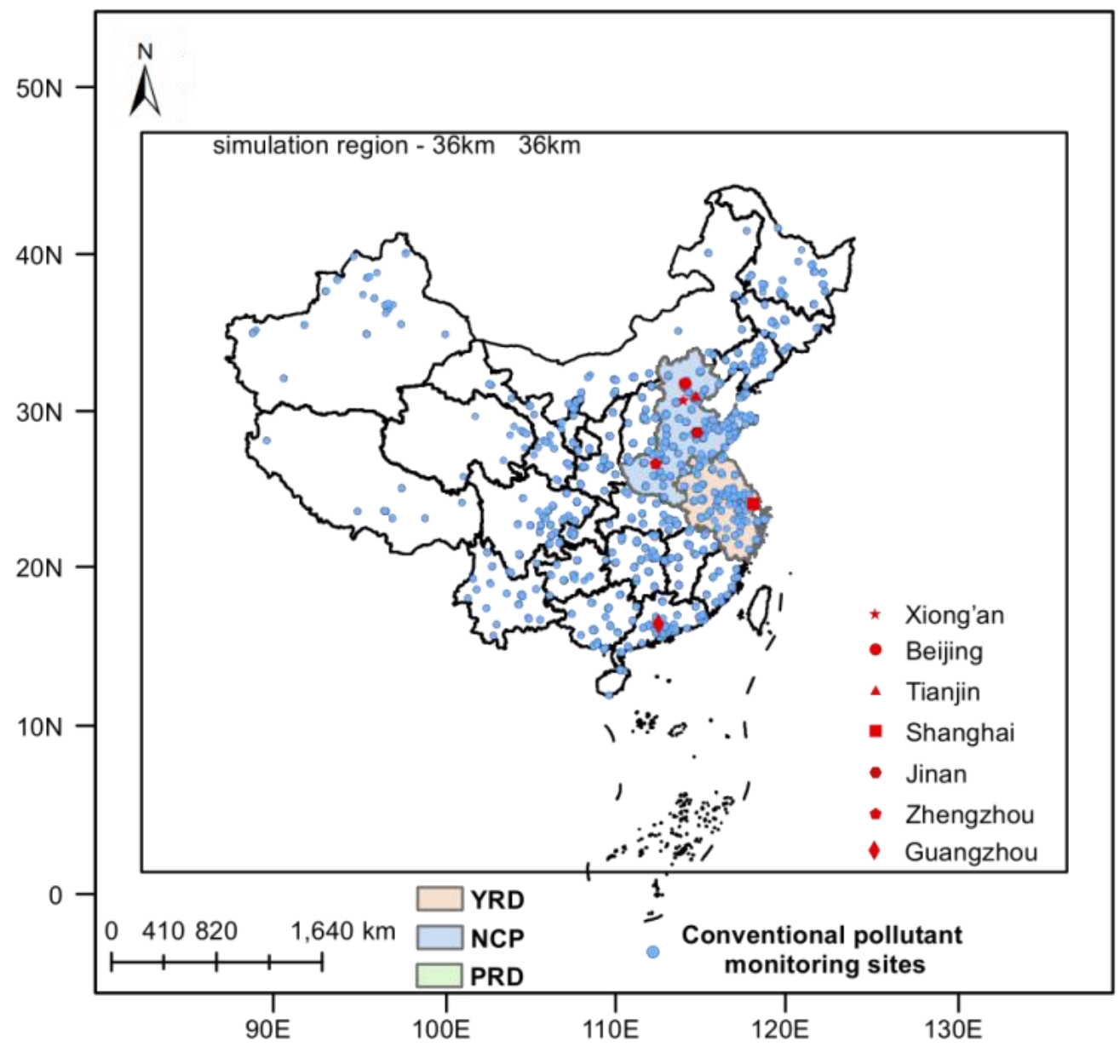

Supplementary Figure 7. Schematic diagram of the master grid and monitoring networks. The grid (36 km × 36 km) is configured for CAMx simulations. The map shows the three megacity clusters designated by the Chinese government as targets for air pollution abatement, the spatial distribution of national monitoring stations for conventional pollutants (e.g., O_3 and NO_2), and specific stations equipped with volatile organic compound (VOC) monitoring (red dots). The three megacity clusters are defined as follows: the North China Plain (NCP, blue, 31°–42° N, 112°–123° E, 368 sites), the Yangtze River Delta (YRD, pink, 27°–35° N, 114°–122° E, 223 sites), and the Pearl River Delta (PRD, green, 21.5°–25° N, 111°–115.5° E, 81 sites). The administrative boundaries are based on the Standard Map Service of the Ministry of Natural Resources of the People's Republic of China (http://bzdt.ch.mnr.gov.cn/), with map approval number GS(2024)0650.

Initial meteorological fields and boundary conditions for the WRF simulations were derived from the National Centers for Environmental Prediction (NCEP) Global Reanalysis Data (Final Analysis, FNL) (http://rda.ucar.edu/datasets/ds083.2/). Land use data for the simulation were obtained from the Moderate Resolution Imaging Spectroradiometer (MODIS) at medium resolution. Key atmospheric physical processes were parameterized using widely adopted schemes: convective cloud processes by the Kain-Fritsch shallow cumulus scheme (new Eta), microphysics by the WRF Single-Moment 3-class Microphysics scheme (WSM3), land surface interactions by the Noah scheme, longwave radiation by the Rapid Radiative Transfer Model (RRTM), shortwave radiation by the Dudhia scheme, and the atmospheric boundary layer by the ACM2 scheme.

Anthropogenic emission inventories for China during 2019-2020 were based on the multiresolution emission inventory for China version 1.4 (MEIC v1.4)[19], provided at an original spatial resolution of 0.25° × 0.25°. Emissions outside of China were derived from the Regional Emission Inventory in Asia version 2.1 (REAS v2.1) (original resolution 0.25° × 0.25°)[20]. For 2021, as the official MEIC v1.4 inventory was updated only up to 2020 at the time of this study, we refined the emission data to reflect the activity levels of that year. This involved using the MEIC 2020 inventory as a spatial distribution baseline and collecting provincial-level total atmospheric pollutant emission data for 2021. We then calculated the provincial total change ratios for each major precursor (2021 vs. 2020) based on these latest statistics. These ratios served as scaling factors, which were applied to adjust the grid-level emissions of the corresponding provinces within the MEIC 2020 inventory. This approach updated the total emissions to levels more representative of 2021 while preserving the validated fine spatial distribution of the MEIC inventory. This method assumes relatively stable internal spatial emission distributions within each province over a single year, a common and reasonable approximation when high-resolution, updated inventories are unavailable. These inventories were converted to the LCC projection coordinate system using geographic distribution factors, including land area, population, industrial and agricultural land use, and road network distribution. The Sparse Matrix Operator Kernel Emissions was employed for sectoral allocation and species assignment of VOCs and particulate matter[21]. Biogenic emissions were calculated using the Model of Emissions of Gases and Aerosols from Nature (MEGAN v3.1)[22].

To assess O_3-NO_x-VOC sensitivity, the Direct Decoupled Method (DDM) tool within CAMx was applied to simulate the boreal summer periods (June–August) of 2019–2021. This allowed us to quantify the response of O_3 concentrations to emission

perturbations of NO_x and anthropogenic VOCs (AVOCs). Building on these simulations and consistent with previous work[3], we also examined the interannual variations of model-derived summer FNR ($HCHO/NO_2$) values from 2020 to 2060 to assess the long-term trends in O_3 formation sensitivity under various emission scenarios.

Model performance evaluation. We evaluated the model's performance against ground-based observations for key pollutants and meteorological variables during the simulation period, focusing on the summer of 2020. Atmospheric pollutant simulation performance was assessed by comparing simulated daily maximum 8-hour average (MDA8) O_3 and daily average NO_2 concentrations with observations from key monitoring sites across the targeted regions. The spatial distribution of simulated versus observed long-term average concentrations demonstrated generally good agreement across most monitoring stations, although overestimation was observed for NO_2 and O_3 in some areas within the NCP and YRD regions. The PRD region showed relatively consistent levels between simulations and observations (Supplementary Figures 8-12).

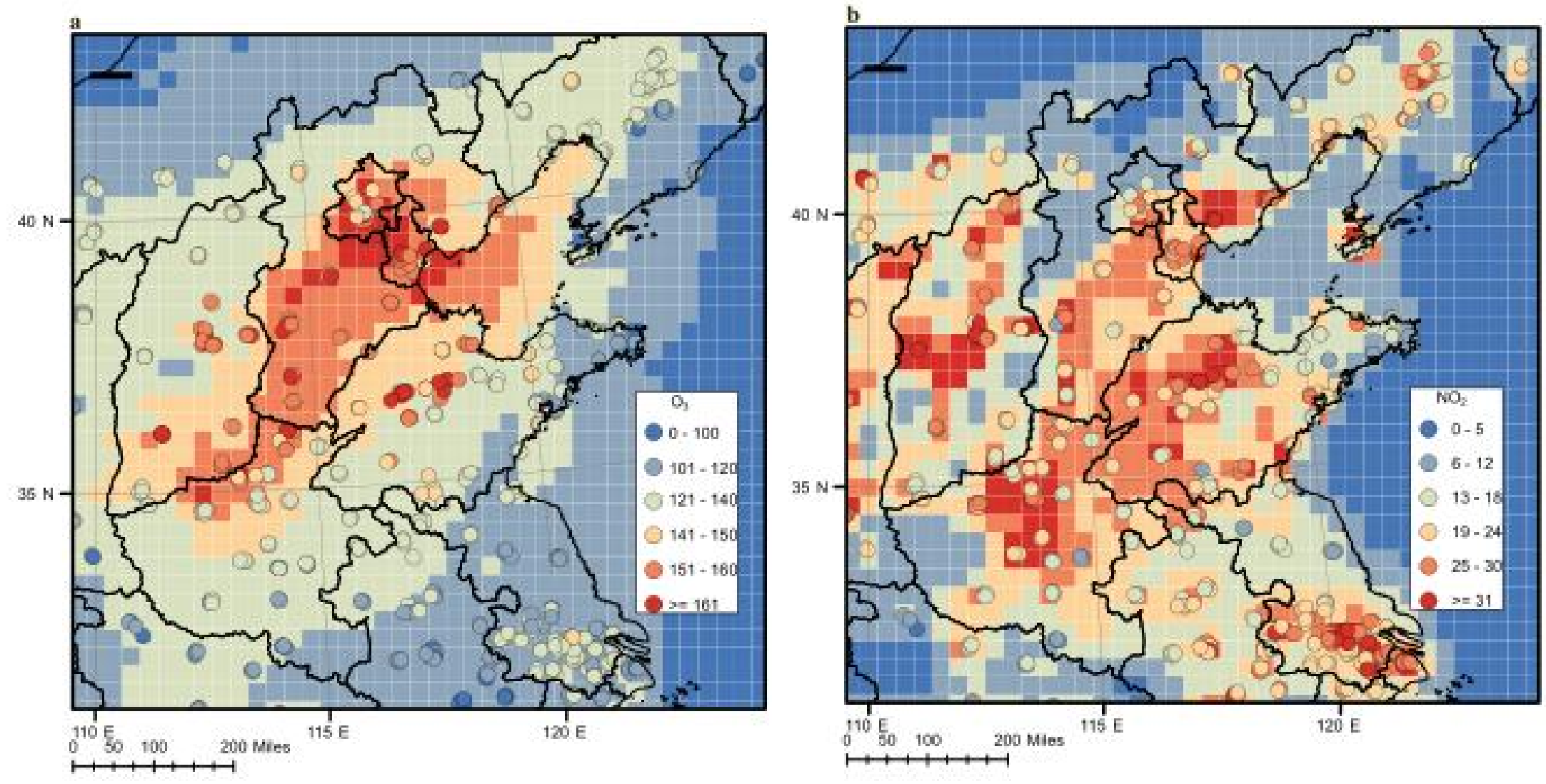

Supplementary Figure 8. Spatial distribution of monitored versus simulated maximum daily 8 h average (MDA8) O_3 and daily NO_2 concentrations in the North China Plain (NCP) region during the summer of 2020. a MDA8O_3 concentrations. **b** NO_2 concentration. Colored dots indicate air pollutant concentrations at monitoring stations; colored matrix grid indicates simulated air pollutant concentrations. Administrative boundaries are based on the Standard Map Service of the Ministry of Natural Resources of the People's Republic of China (http://bzdt.ch.mnr.gov.cn/), with map approval number GS(2024)0650.

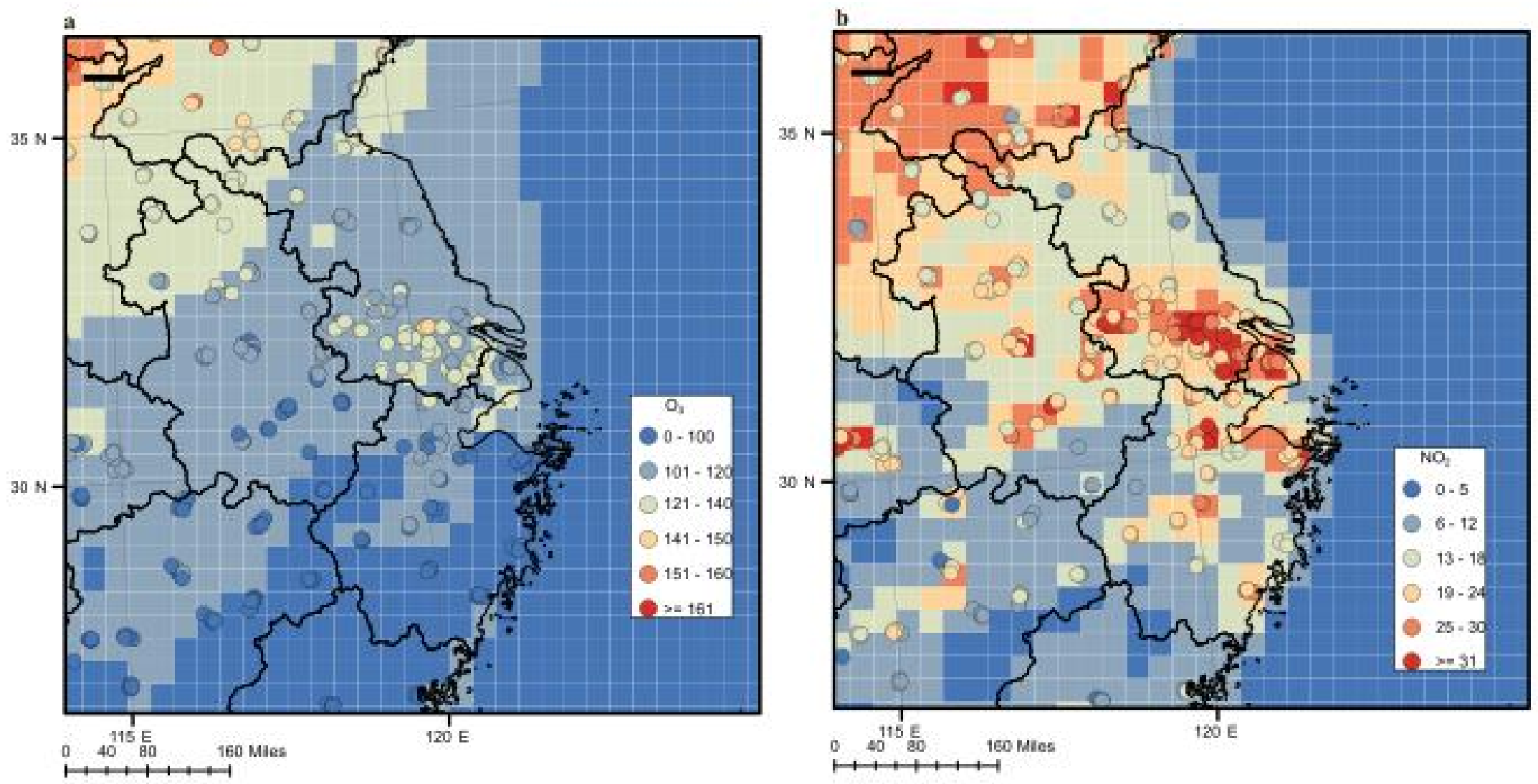

Supplementary Figure 9. Spatial distribution of monitored versus simulated daily maximum daily 8 h average (MDA8) O_3 and daily NO_2 concentrations in the Yangtze River Delta (YRD) region during the summer of 2020. a MDA8O_3 concentrations. **b** NO_2 concentration. Colored dots indicate air pollutant concentrations at monitoring stations; colored matrix grid indicates simulated air pollutant concentrations. Administrative boundaries are based on the Standard Map Service of the Ministry of Natural Resources of the People's Republic of China (http://bzdt.ch.mnr.gov.cn/), with map approval number GS(2024)0650.

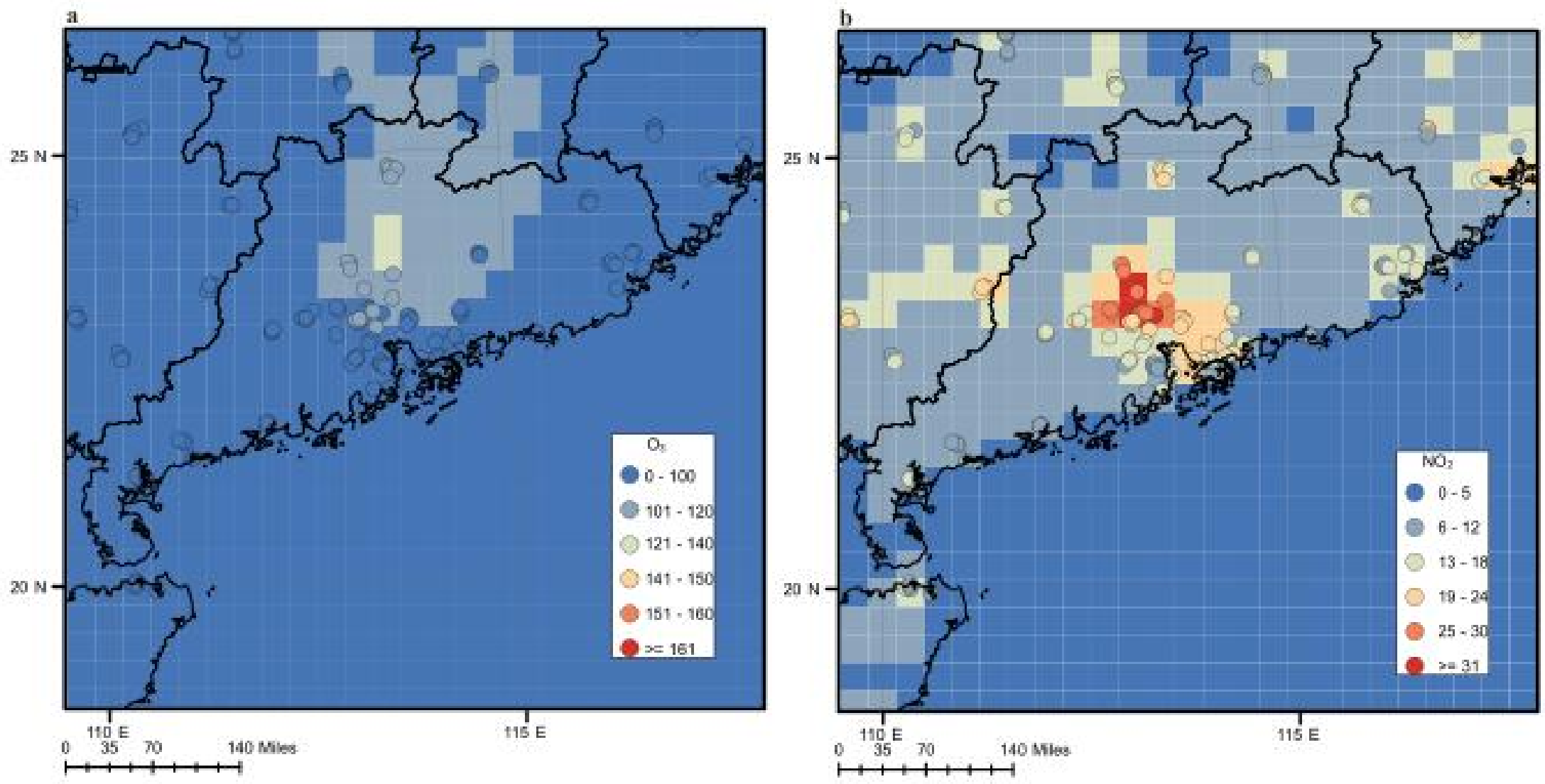

Supplementary Figure 10. Spatial distribution of monitored versus simulated maximum daily 8 h average (MDA8) O_3 and daily NO_2 concentrations in the Pearl River Delta (PRD) region during the summer of 2020. a MDA8O_3 concentrations. **b** NO_2 concentration. Colored dots indicate air pollutant concentrations at monitoring stations; colored matrix grid indicates simulated air pollutant concentrations. Administrative boundaries are based on the Standard Map Service of the Ministry of Natural Resources of the People's Republic of China (http://bzdt.ch.mnr.gov.cn/), with map approval number GS(2024)0650.

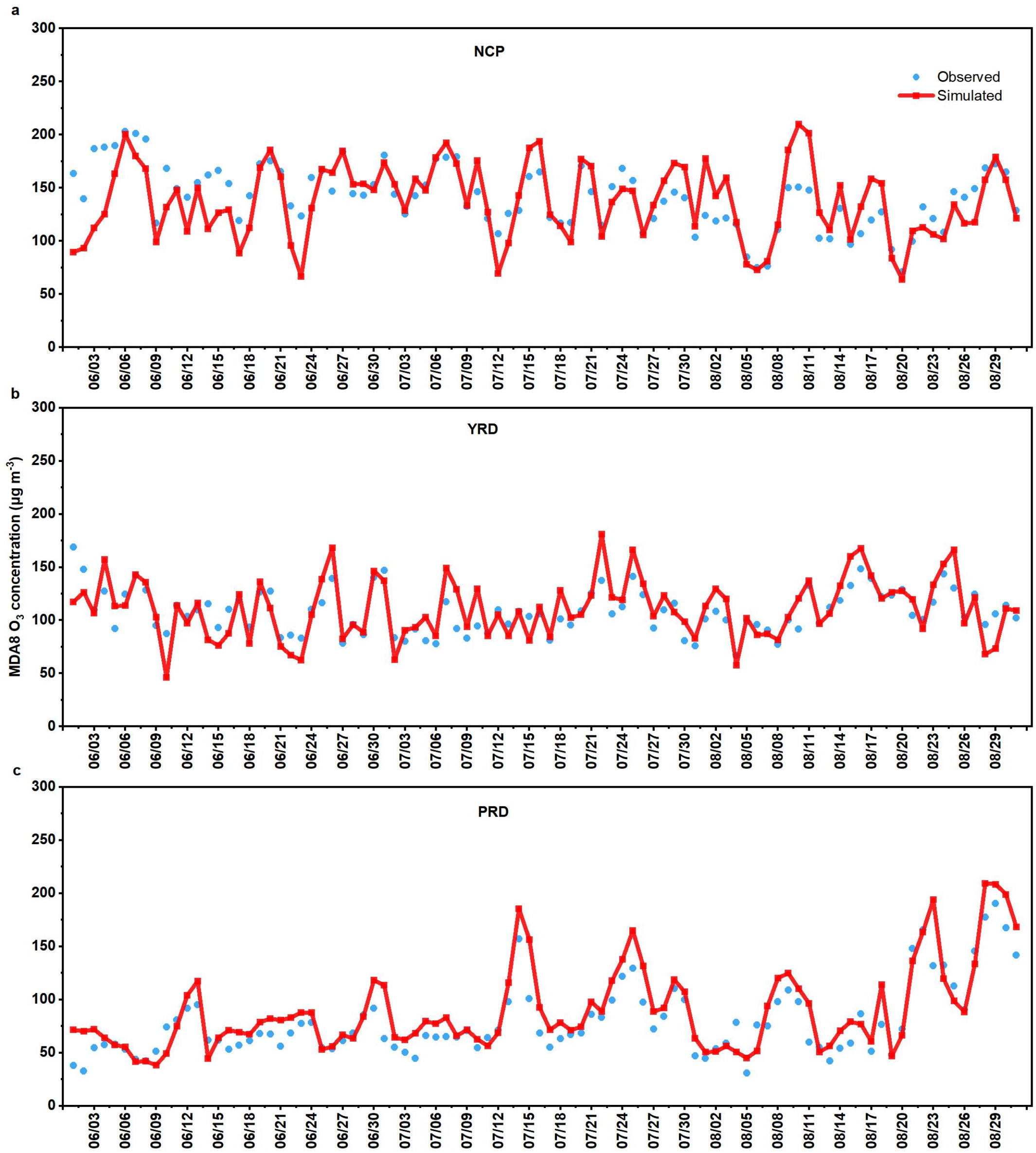

Supplementary Figure 11. Comparison of monitored and simulated daily maximum daily 8 h average (MDA8) O_3 concentrations for the three megacity clusters during the summer of 2020. The three megacity clusters are as follows: **a** the North China Plain (NCP), **b** the Yangtze River Delta (YRD) and **c** the Pearl River Delta (PRD).

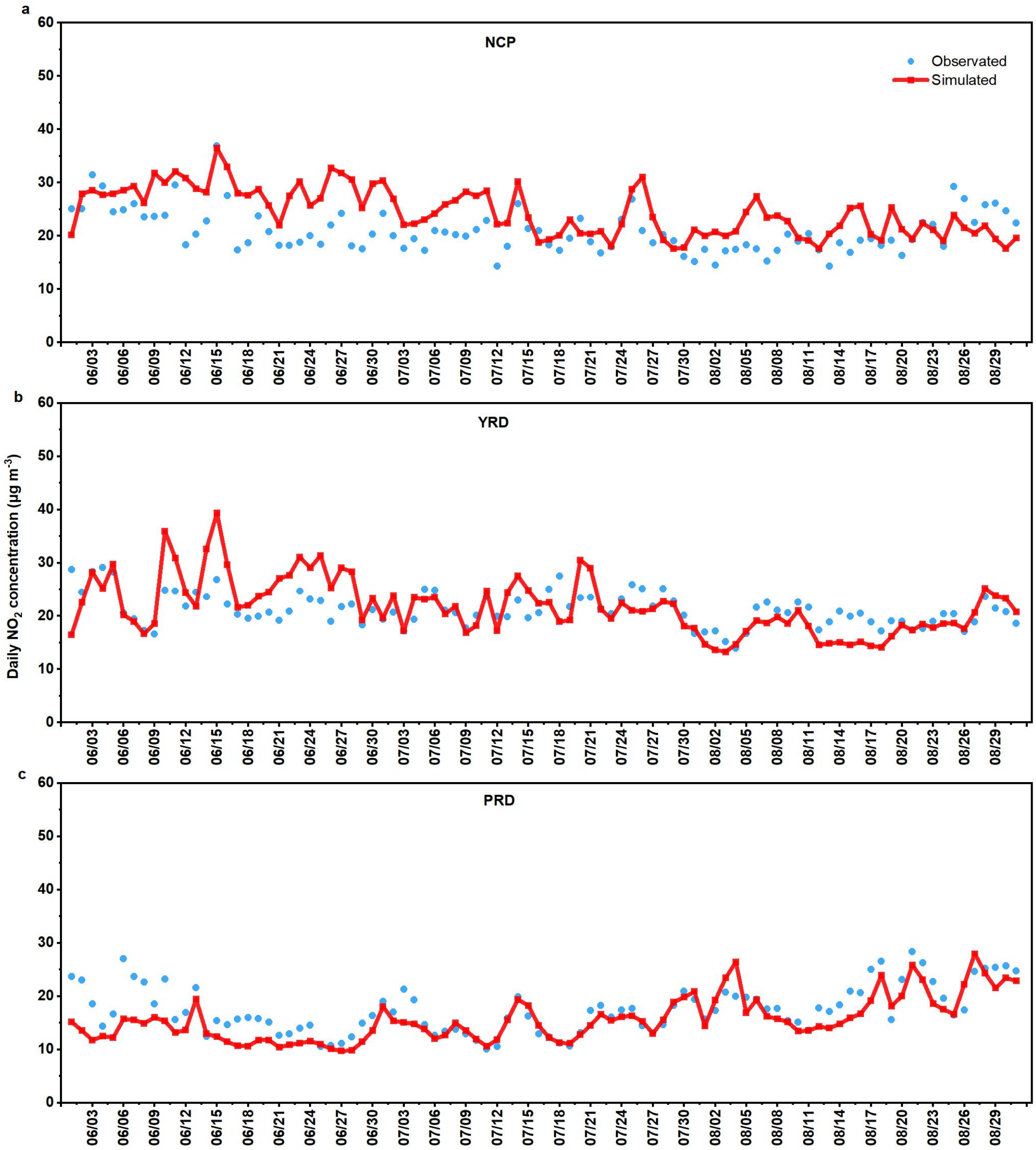

Supplementary Figure 12. Comparison of monitored and simulated daily NO_2 concentrations for the three megacity clusters during the summer of 2020. The three megacity clusters are as follows: **a** the North China Plain (NCP), **b** the Yangtze River Delta (YRD) and **c** the Pearl River Delta (PRD).

Quantitative model performance was further evaluated using five statistical indicators: Mean Bias (MB), Mean Error (ME), Normalized Mean Bias (NMB), Normalized Mean Error (NME), and Pearson's correlation coefficient (*r*) (Supplementary Table 9). For MDA8 O_3 concentrations, the NMB ranged from

-0.79% to 14.94%, MB from 0.56 to 10.42 μg/m^3, and r from 0.68 to 0.88. The PRD region exhibited the strongest correlation. For daily average NO_2 concentrations, NMB ranged from -11.69% to 25.86%, MB from -0.03 to 4.98 μg/m^3, and *r* from 0.45 to 0.72, with the PRD also showing the best correlation. Overall, the *r* values for simulated O_3 concentrations in each region met or exceeded the standard range (>0.5) recommended by Emery et al.[23], with PRD exceeding the target standard (>0.75). The NMB and NME values were within the standard ranges (±15% and <25%, respectively). The spatial distribution and performance statistics are consistent with evaluation results reported in other studies[24, 25], providing evidence of the model's good performance in simulating both NO_2 and O_3 concentrations. Furthermore, while hourly observational data for key VOC species were available for several urban sites and used to constrain the OBM, a direct statistical validation of WRF-CAMx-simulated VOCs was not included in Supplementary Table 10. This is primarily due to the mismatch in spatial representativeness: the point-based urban measurements may not accurately reflect the average concentrations across a 36 km × 36 km model grid cell, which integrates a heterogeneous mix of emission sources. Nevertheless, the model's robust performance in simulating O_3 and NO_2, which are intrinsically linked to VOC chemistry, provides indirect confidence in the overall representation of the regional photochemical environment. This approach is consistent with prior regional modeling studies where comprehensive VOC validation is often limited by data availability and representativeness.

Supplementary Table 9. Model performance statistics for O_3 and NO_2 in China from June to August 2020. Values are presented in percentages (%) for Normalized Mean Bias (NMB) and Normalized Mean Error (NME).

		R	NMB	NME	MB	ME	RMSE
	NCP	0.45	25.86	39.60	4.98	8.06	9.99
NO_2	YRD	0.47	1.53	29.70	-0.03	6.06	7.87
	PRD	0.72	-11.69	24.26	-1.89	4.09	5.12
	NCP	0.68	0.79	21.89	0.56	30.58	39.00
O_3	YRD	0.73	1.52	21.96	0.82	23.46	30.00
	PRD	0.88	14.94	23.88	10.42	18.10	24.20

Supplementary Table 10. Model performance statistics for meteorological data in China from June to August 2020. Values are presented in percentages (%) for Normalized Mean Bias (NMB) and Normalized Mean Error (NME).

		R	NMB	NME	MB	ME	RMSE
	NCP	0.99	-0.80	0.84	-7.70	8.14	8.21
Pressure	YRD	0.98	-0.73	0.84	-7.22	8.29	8.37
	PRD	0.97	-0.64	0.70	-6.38	7.04	7.08
	NCP	0.88	-13.92	15.50	-9.75	10.82	12.94
Relative humidity	YRD	0.81	2.36	7.17	1.81	5.86	7.27
	PRD	0.66	-4.15	8.95	-3.31	7.17	8.52
	NCP	0.82	-0.76	5.11	-0.17	1.29	1.58
Temperature	YRD	0.89	-5.71	6.47	-1.57	1.78	2.06
	PRD	0.76	-2.10	4.65	-0.61	1.37	1.60
	NCP	0.57	-0.19	37.57	-0.05	0.89	1.10
Wind speed	YRD	0.60	14.29	39.76	0.22	0.99	1.23
	PRD	0.59	17.63	37.27	0.52	1.12	1.39

The accuracy of the simulated meteorological fields, crucial for atmospheric pollutant formation, was also verified by comparing WRF-simulated temperature, relative humidity, atmospheric pressure, and wind speed against observations from available weather stations in the NCP, YRD, and PRD regions during the summer of 2020. Daily average simulated meteorological variables for the model grids containing observation stations were aggregated to city-wide averages for comparison (Supplementary Table 10). The WRF model reasonably reproduces the spatiotemporal

variations of these meteorological variables within the study regions. Daily average simulated temperature and atmospheric pressure exhibit high consistency with observations, with Pearson's correlation coefficients (r) ranging from 0.76 to 0.89 and 0.97 to 0.99 for the three regions, respectively, although NMB indicated a slight underestimation. Simulated relative humidity also shows good consistency ($r \approx 0.66$–0.88, average MB ≈ -3.75%). Although wind speed correlations are slightly lower ($r \approx 0.57$–0.60), the overall robust simulation performance provides reliable meteorological field inputs for the chemical transport model.

In summary, the model version, physicochemical schemes, and parameterization configurations used in this study are fully consistent with our previous work[3, 26-28], and this extensively validated WRF-CAMx system has demonstrated robust performance in simulating NO_2 and O_3 concentrations across multiple years and various emission control scenarios, providing strong evidence of its generalization ability and reliability under diverse conditions.

The detailed reduction proportions and the subsequent O_3 mitigation trajectories under different scenarios are further illustrated in Supplementary Figures 13–19, and Supplementary Tables 11–13.

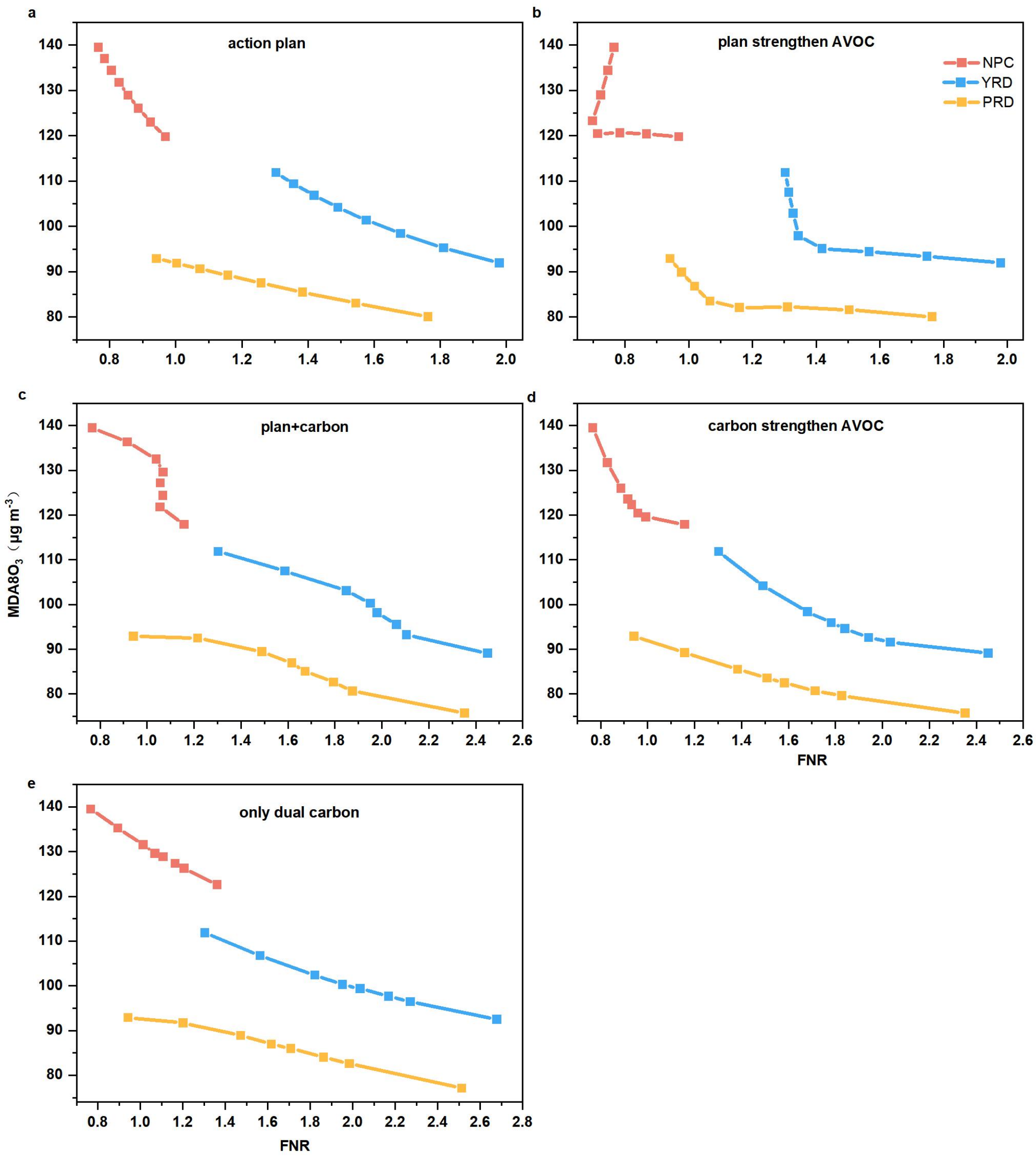

Supplementary Figure 13. Path dependency in O_3 mitigation revealed by 2020–2060 model simulations. Distinct emission reduction pathways drive the O_3 photochemical system along divergent evolution trajectory. **a–e** Results for different emission-reduction scenarios. Colored dots indicate the years of 2020, 2025, 2030, 2040, 2045, 2050 and 2060, respectively. To isolate the impact of anthropogenic emission changes, meteorological conditions and biogenic VOC (BVOC) emissions were fixed at 2020 levels.

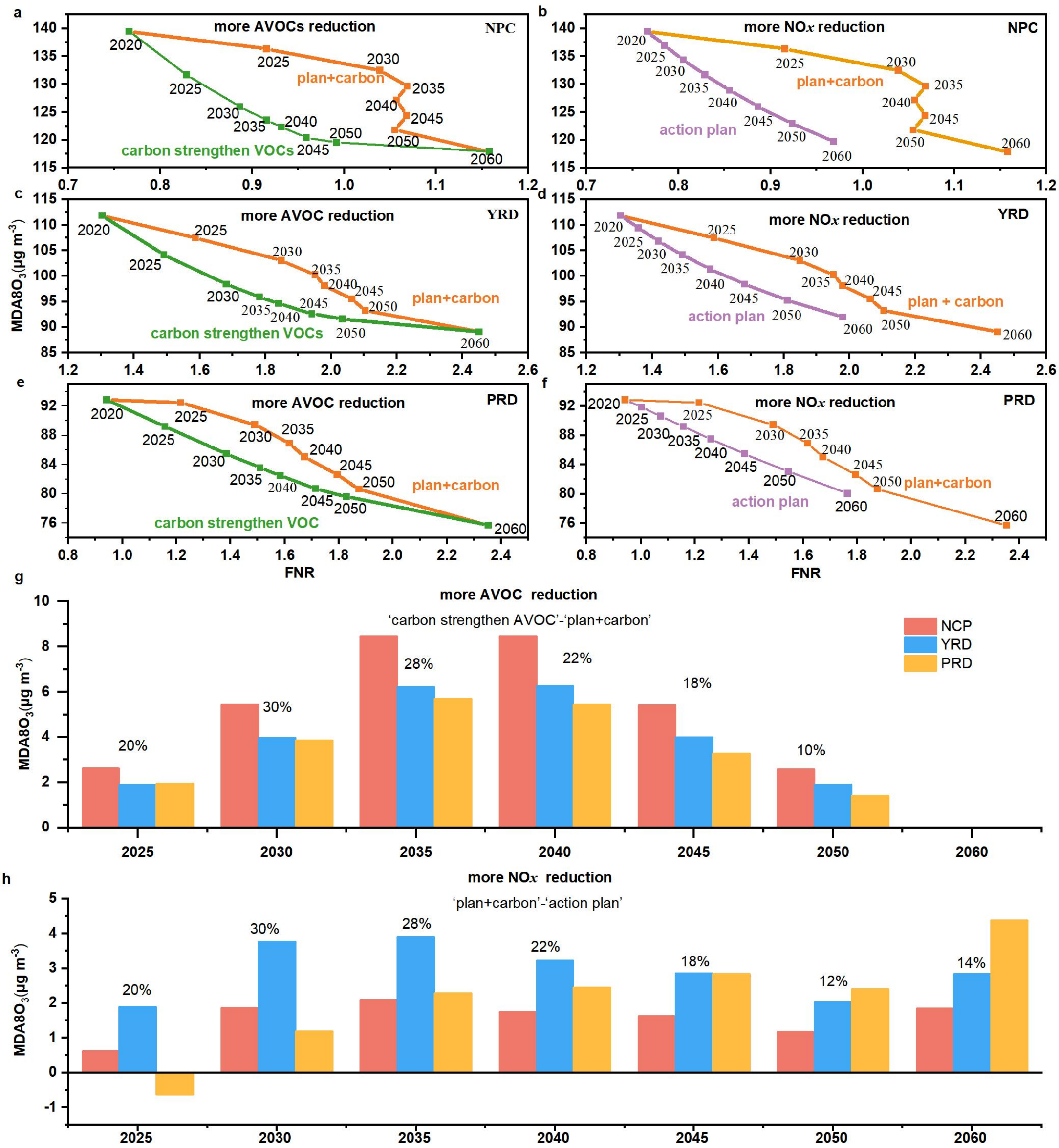

Supplementary Figure 14. Derivation of isolated maximum daily 8 h average (MDA8) O_3 reduction benefits from AVOC versus equivalent proportional NO*x* reduction. a–f evolution trajectories of O_3 photochemical system, represented by O_3 versus the formaldehyde-to-nitrogen dioxide ratio (FNR), under paired-scenario ('carbon strengthen AVOCs' *vs* 'plan+carbon' and 'plan+carbon' *vs* 'action plan') designed to decouple the effects of anthropogenic VOC (AVOC) versus NO*x* reductions in China's three megacity clusters from 2020 to 2060. The 'More AVOCs reduction' comparison isolates the impact of AVOCs by comparing the 'carbon strengthen AVOCs' and 'plan+carbon' scenarios, which share an identical NO*x*

trajectory. The ‘More NO*x* reduction’ comparison isolates the impact of NO*x* by comparing the ‘plan+carbon’ and ‘action plan’ scenarios, which share an identical AVOC trajectory. **g** Isolated MDA8O_3 reduction from dedicated AVOC controls, calculated as the MDA8O_3 difference between the ‘carbon strengthen AVOCs’ and ‘plan+carbon’ scenarios. Percentages indicate the additional AVOC reduction for that year. **h** Isolated MDA8 O_3 reduction from dedicated NO*x* controls, calculated as the MDA8 O_3 difference between the ‘plan+carbon’ and ‘action plan’ scenarios. Percentages indicate the additional NO*x* reduction for that year.

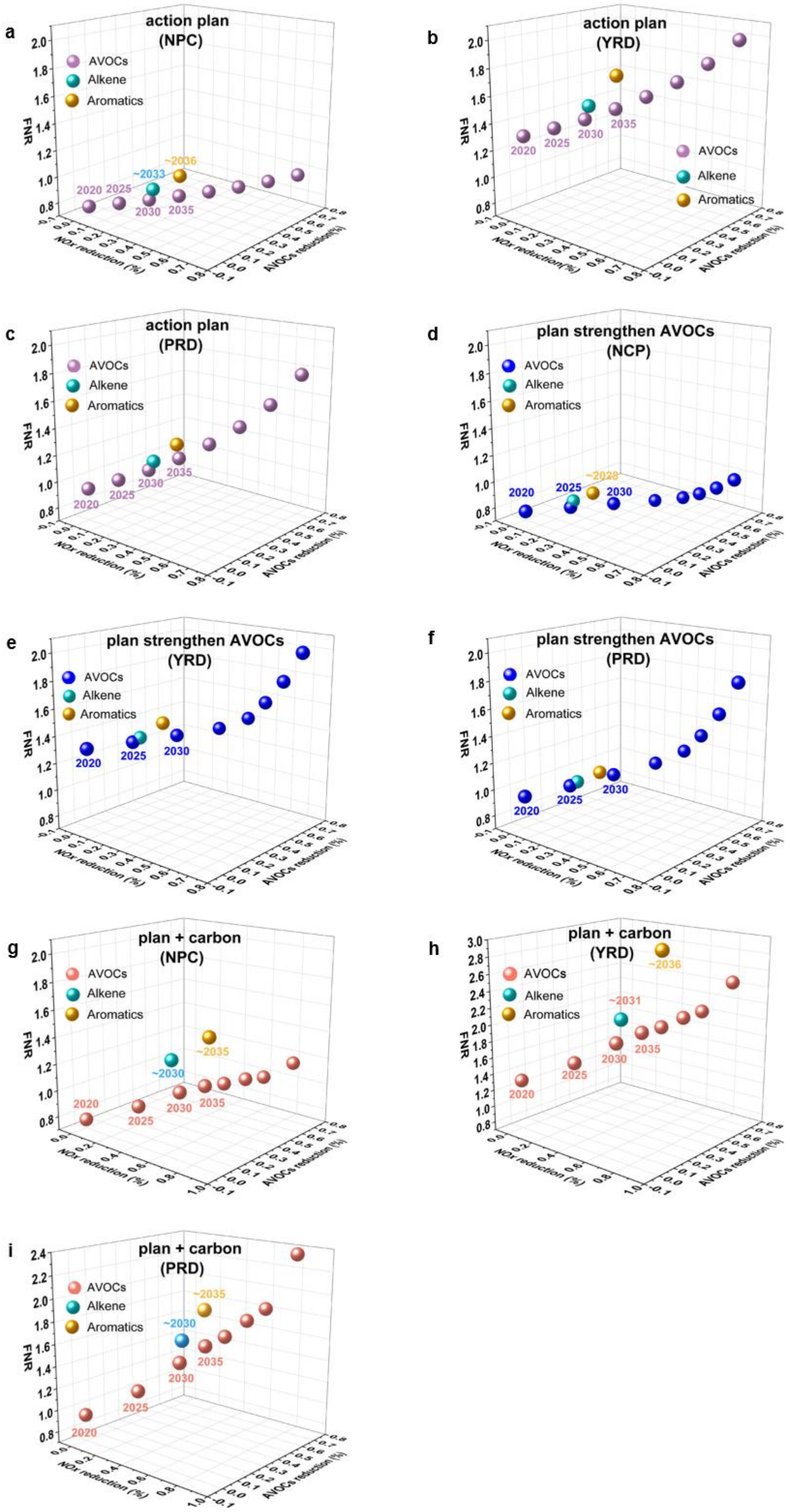

Supplementary Figure 15. Formaldehyde-to-nitrogen dioxide ratio (FNR) trajectory of different megacity clusters under different NO*x* to anthropogenic VOC (AVOC) reduction ratios. a–c FNR values in different years under 'action

plan' scenario of the three megacity clusters: the North China Plain (NCP), the Yangtze River Delta (YRD) and the Pearl River Delta (PRD). **d–f** FNR values in different years under 'plan strengthen AVOCs' scenario of NCP, YRD and PRD. **g–i** FNR values in different years under 'plan + carbon' scenario of NCP, YRD and PRD. AVOCs represent equal reduction all species, Alkene represents all alkene species of MEIC inventory were reduced, and Aromatics represent all aromatic compounds were reduced. The biogenic VOC (BVOC) emission and meteorological field input are fixed at the 2020 level.

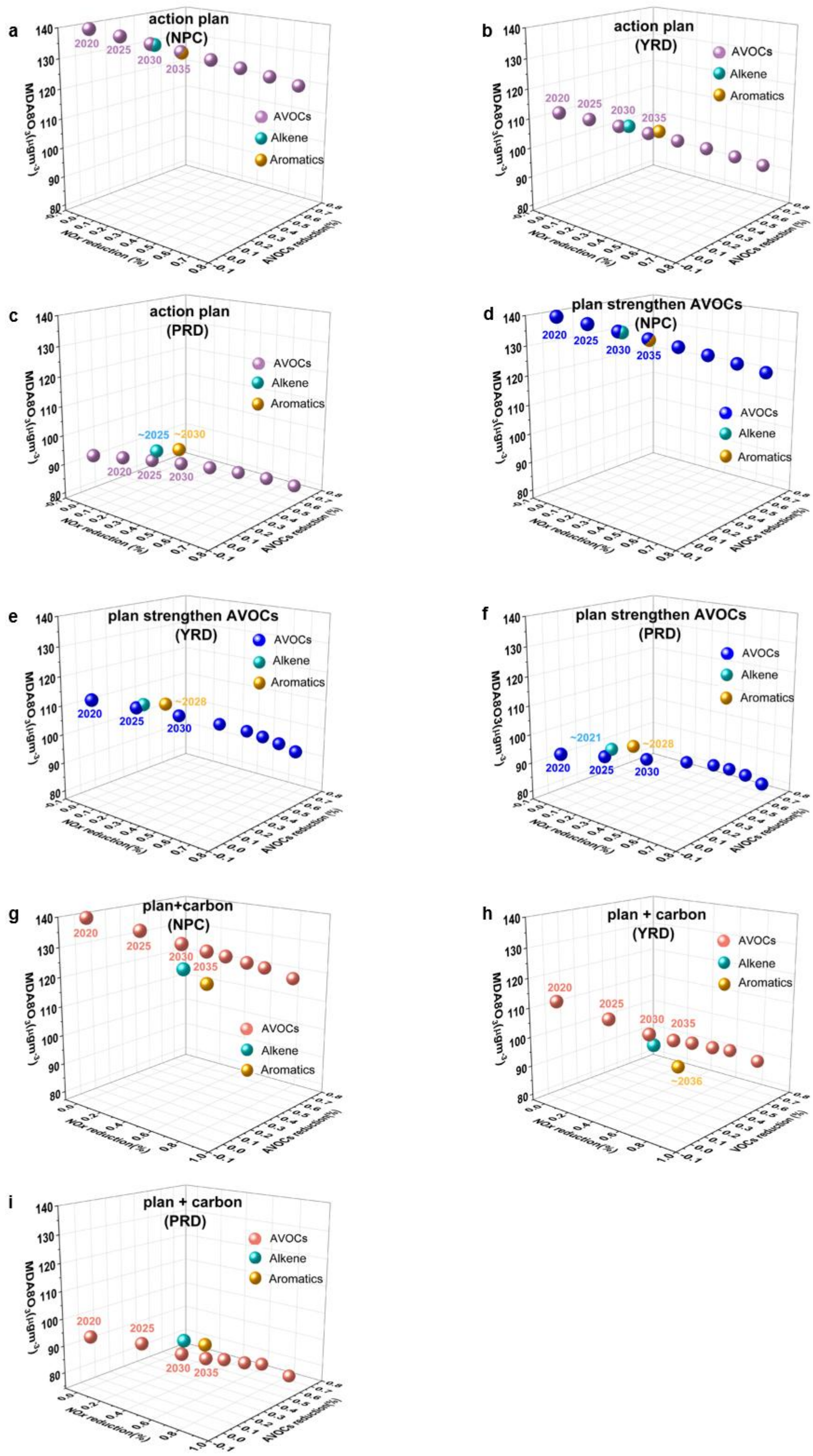

Supplementary Figure 16. The maximum daily 8 h average (MDA8) O_3 concentrations trajectory of different megacity clusters under different NO*x* to anthropogenic VOC (AVOC) reduction ratios. a–c MDA8 O_3 concentrations in different years under 'action plan' scenario of the three megacity clusters: the North

China Plain (NCP), the Yangtze River Delta (YRD) and the Pearl River Delta (PRD). **d–f** MDA8 O_3 concentrations in different years under 'plan strengthen AVOCs' scenario of NCP, YRD and PRD. **g–i** MDA8 O_3 concentrations in different years under 'plan + carbon' scenario of NCP, YRD and PRD. AVOCs represent equal reduction all species, Alkene represents all alkene species of MEIC inventory were reduced, and Aromatics represent all aromatic compounds were reduced. The biogenic VOC (BVOC) emission and meteorological field input are fixed at the 2020 level.

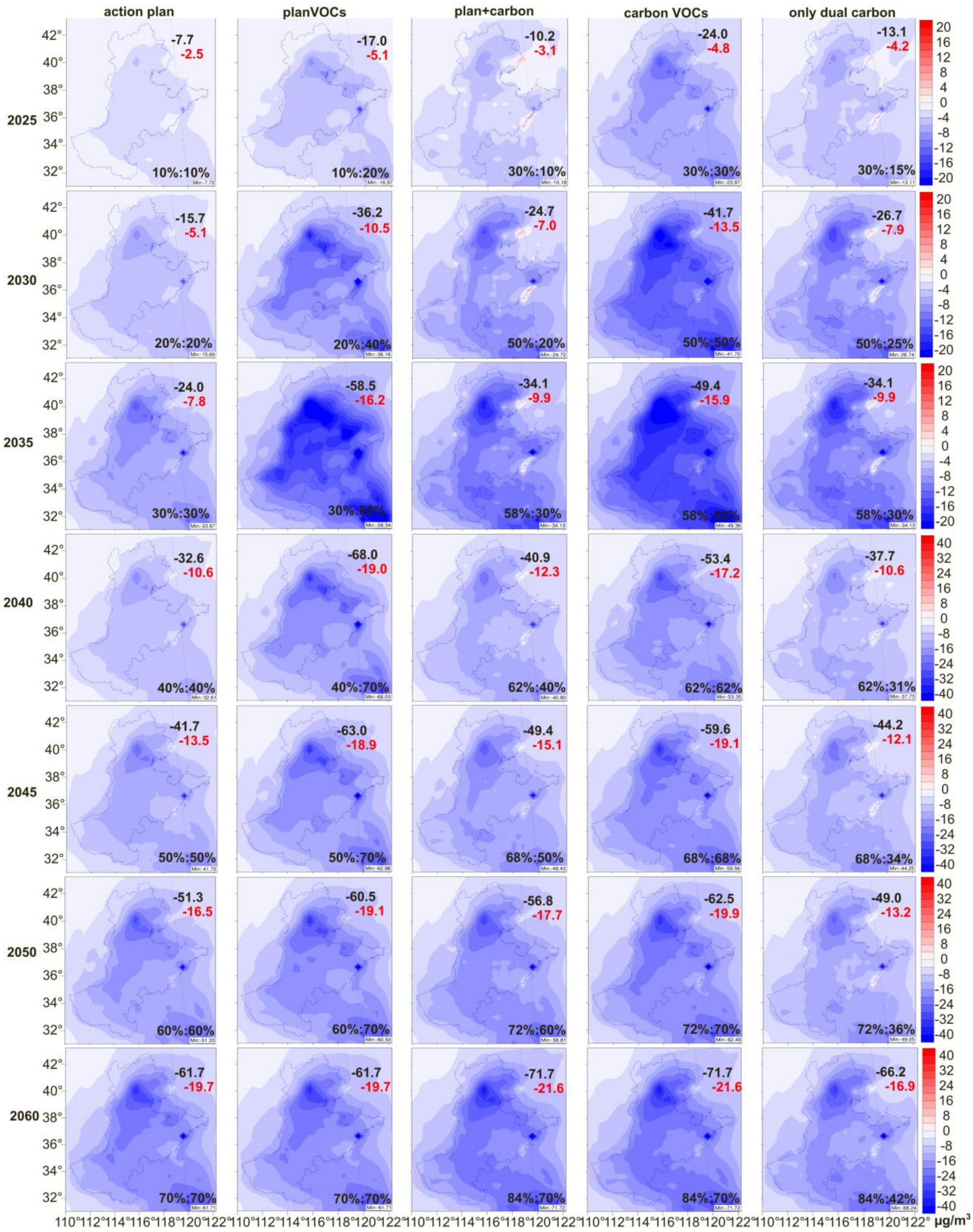

Supplementary Figure 17. Spatial distribution of summer maximum daily 8 h average (MDA8) O_3 reduction over the North China Plain (NCP) relative to 2020 levels. Numerical values within the panels denote the reduction ratios of NO*x* to VOCs, along with the corresponding maximum (black) and area-averaged (red) summer MDA8 O_3 reduction concentrations. Meteorological inputs are fixed at 2020 levels. Administrative boundaries are based on the Standard Map Service of the

Ministry of Natural Resources of the People's Republic of China (http://bzdt.ch.mnr.gov.cn/), with map approval number GS(2024)0650.

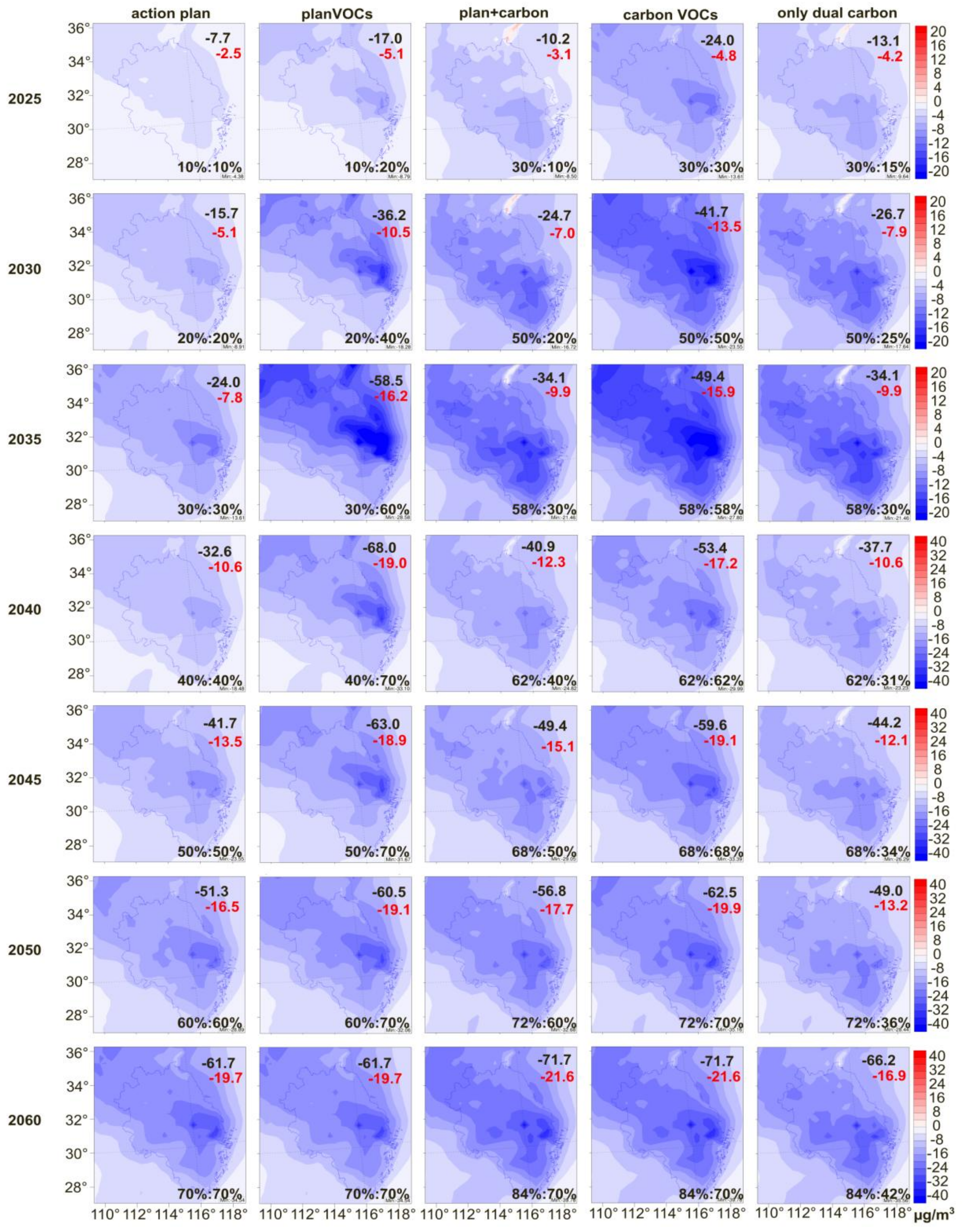

Supplementary Figure 18. Spatial distribution of summer maximum daily 8 h average (MDA8) O_3 reduction over the Yangtze River Delta (YRD) relative to 2020 levels. Numerical values within the panels denote the reduction ratios of NO*x* to VOCs, along with the corresponding maximum (black) and area-averaged (red) summer MDA8 O_3 reduction concentrations. Meteorological inputs are fixed at 2020 levels. Administrative boundaries are based on the Standard Map Service of the

Ministry of Natural Resources of the People's Republic of China (http://bzdt.ch.mnr.gov.cn/), with map approval number GS(2024)0650.

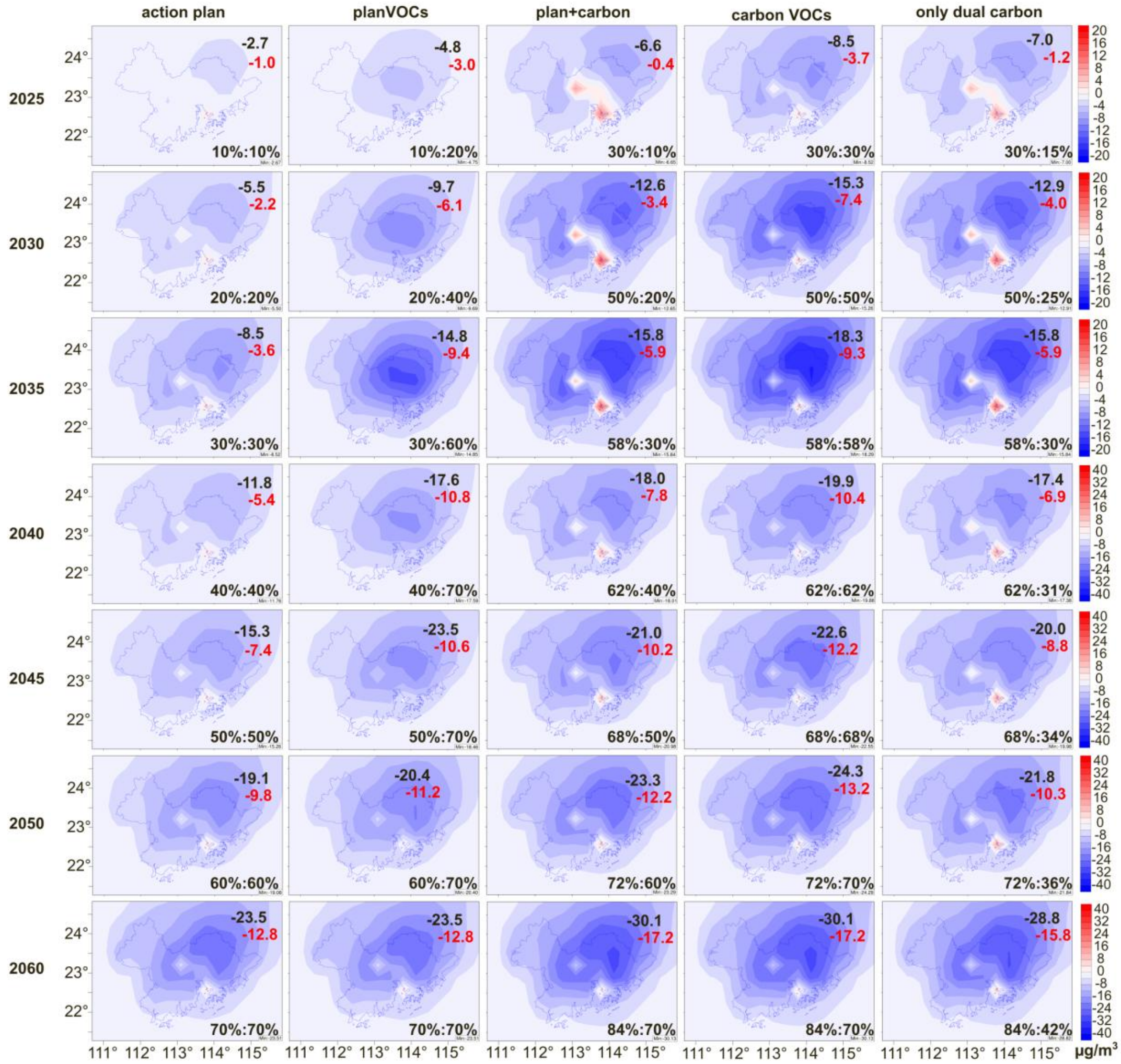

Supplementary Figure 19. Spatial distribution of summer maximum daily 8 h average (MDA8) O_3 reduction over the Pearl River Delta (PRD) relative to 2020 levels. Numerical values within the panels denote the reduction ratios of NO*x* to VOCs, along with the corresponding maximum (black) and area-averaged (red) summer MDA8 O_3 reduction concentrations. Meteorological inputs are fixed at 2020 levels. Administrative boundaries are based on the Standard Map Service of the Ministry of Natural Resources of the People's Republic of China (http://bzdt.ch.mnr.gov.cn/), with map approval number GS(2024)0650.

Supplementary Table 11. The reduction proportions of NO*x* to anthropogenic VOCs (AVOCs) from 2020 to 2060 under three different scenarios in the North China Plain (NCP) megacity cluster. AVOCs scenario represents an equal reduction across all species; Alkene indicates reductions applied exclusively to all alkene species in the MEIC inventory; Aromatics indicates reductions applied exclusively to all aromatic compounds. The base emission inventory is fixed at 2020 levels.

Year	Species reduction	action plan	Year	Species reduction	plan strengthen AVOCs	Year	Species reduction	plan+carbon
2025	AVOCs	10%:10%	2025	AVOCs	10%:20%	2025	AVOCs	30%:10%
2030	AVOCs	20%:20%		Alkene	10.6%:21.3%	2030	AVOCs	50%:20%
	Alkene	21.3%:21.3%		Aromatics	15.2%:30.3%		Alkene	50%:21.3%
2035	AVOCs	30%:30%	2030	AVOCs	20%:40%	2035	AVOCs	58%:30%
	Aromatics	30.3%:30.3%	2035	AVOCs	30%:60%		Aromatics	58%:30.3%
2040	AVOCs	40%:40%	2040	AVOCs	40%:70%	2040	AVOCs	62%:40%
2045	AVOCs	50%:50%	2045	AVOCs	50%:70%	2045	AVOCs	68%:50%
2050	AVOCs	60%:60%	2050	AVOCs	60%:70%	2050	AVOCs	72%:60%
2060	AVOCs	70%:70%	2060	AVOCs	70%:70%	2060	AVOCs	84%:70%

Supplementary Table 12. The reduction proportions of NO*x* to anthropogenic VOCs (AVOCs) from 2020 to 2060 under three different scenarios in the Yangtze River Delta (YRD) megacity cluster. AVOCs scenario represents an equal reduction across all species; Alkene indicates reductions applied exclusively to all alkene species in the MEIC inventory; Aromatics indicates reductions applied exclusively to all aromatic compounds. The base emission inventory is fixed at 2020 levels.

Year	Species reduction	action plan	Year	Species reduction	plan strengthen VOCs	Year	Species reduction	plan+carbon
2025	AVOCs	10%:10%	2025	AVOCs	10%:20%	2025	AVOCs	30%:10%
2030	AVOCs	20%:20%		Alkene	11.6%:23.3%	2030	AVOCs	50%:20%
	Alkene	23.3%:23.3%		Aromatics	16.8%:33.6%		Alkene	50%:23.3%
2035	AVOCs	30%:30%	2030	AVOCs	20%:40%	2035	AVOCs	58%:30%
	Aromatics	33.6%:33.6%	2035	AVOCs	30%:60%		Aromatics	58%:33.6%
2040	AVOCs	40%:40%	2040	AVOCs	40%:70%	2040	AVOCs	62%:40%
2045	AVOCs	50%:50%	2045	AVOCs	50%:70%	2045	AVOCs	68%:50%
2050	AVOCs	60%:60%	2050	AVOCs	60%:70%	2050	AVOCs	72%:60%
2060	AVOCs	70%:70%	2060	AVOCs	70%:70%	2060	AVOCs	84%:70%

Supplementary Table 13. The reduction proportions of NO*x* to anthropogenic VOCs (AVOCs) from 2020 to 2060 under three different scenarios in the Pearl River Delta (PRD) megacity cluster. AVOCs scenario represents an equal reduction across all species; Alkene indicates reductions applied exclusively to all alkene species in the MEIC inventory; Aromatics indicates reductions applied exclusively to all aromatic compounds. The base emission inventory is fixed at 2020 levels.

Year	Species reduction	action plan	Year	Species reduction	plan strengthen VOCs	Year	Species reduction	plan+carbon
2025	AVOCs	10%:10%	2025	AVOCs	10%:20%	2025	AVOCs	30%:10%
2030	AVOCs	20%:20%		Alkene	10.8%:21.6%	2030	AVOCs	50%:20%
	Alkene	21.6%:21.6%		Aromatics	14.6%:29.3%		Alkene	50%:21.6%
	Aromatics	29.3%:29.3%	2030	AVOCs	20%:40%	2035	AVOCs	58%:30%
2035	AVOCs	30%:30%	2035	AVOCs	30%:60%		Aromatics	58%:29.3%
2040	AVOCs	40%:40%	2040	AVOCs	40%:70%	2040	AVOCs	62%:40%
2045	AVOCs	50%:50%	2045	AVOCs	50%:70%	2045	AVOCs	68%:50%
2050	AVOCs	60%:60%	2050	AVOCs	60%:70%	2050	AVOCs	72%:60%
2060	AVOCs	70%:70%	2060	AVOCs	70%:70%	2060	AVOCs	84%:70%

Supplementary Note 5 Observation-based model (OBM) simulations

To investigate in-situ O_3 formation chemistry and sensitivity in urban core environments, we employed an Observational Box Model (OBM). This approach is widely utilized for quantifying local O_3 production rates and their sensitivity to precursor concentrations[2]. The OBM simulations were constrained by hourly observational data for key atmospheric constituents (O_3, NO_x, CO, VOCs) and meteorological parameters (temperature, humidity, pressure), along with estimated NO_2 photolysis rates (JNO_2) derived from solar radiation data. These comprehensive datasets were acquired from a selection of seven representative urban sites across the study regions (see Supplementary Note 6 for quality control procedures).

The core chemical mechanism within the OBM was the Master Chemical Mechanism (MCM v3.3.1), which provides a near-explicit description of the gas-phase degradation pathways for 143 primary VOCs and their subsequent reaction products[29,30]. The model also incorporated key physical processes essential for simulating urban boundary layer chemistry, including diurnal variations in solar radiation intensity, planetary boundary layer height evolution, dry deposition of reactive species, and turbulent exchange with background air. To ensure that unmeasured, short-lived species (e.g., OH radicals) reached a pseudo-steady state representative of the simulation period, the model was pre-run for four days preceding each simulation interval. A detailed description of the OBM configuration can be found in previous work[2].

Calculation of Relative Incremental Reactivity (RIR): The RIR is the widely utilized metric for assessing the sensitivity of O_3 production to its precursors. RIR for a given precursor (X) is defined as the normalized change in the net O_3 production

rate in response to a proportional perturbation in the precursor's concentration. In this study, RIRs were determined through OBM sensitivity simulations by applying a uniform 20% reduction to the concentration of specific O_3 precursors, including NO_x, anthropogenic VOCs (AVOCs), and biogenic VOCs (BVOCs). Summer daytime average (09:00–17:00 local time) RIRs for these major O_3 precursors were calculated daily from 2019 to 2021 across the six selected urban locations.

The RIR for each precursor (X) was calculated using the supplementary equation (1):

$$\mathrm{RIR}^{(s)}(X)=\frac{\frac{\mathrm{P}^{S}_{\mathrm{O_3\text{-}NO}}(X)-\mathrm{P}^{S}_{\mathrm{O_3\text{-}NO}}([X]-\triangle[X])}{\mathrm{P}^{S}_{\mathrm{O_3\text{-}NO}}(X)}}{\frac{\triangle S(X)}{S(X)}} \quad (1)$$

where $\mathrm{P}_{\mathrm{O_3\text{-}NO}}(X)$ is the mean formation rate of the O_3 to precursor (X) from 9:00 to 17:00, and the relative change in the precursor ($\triangle S(X)/S(X)$) was set to 20% reduction in the concentration of X.

Trends in urban O_3 formation sensitivity were subsequently analyzed based on the interannual variation of these calculated averaged daily RIR values for different O_3 precursors. Given that O_3 formation in all six cities was VOC-limited during the study period (indicated by negative $\mathrm{RIR}_{\text{-}NO_x}$ values), the directional change in sensitivity can be inferred from the relative shifts in RIRs. An increase in $\mathrm{RIR}_{\text{-}NO_x}$ concurrent with a decrease in $\mathrm{RIR}_{\text{-AVOCs}}$ suggests a progression towards NO_x-limited regime direction. Conversely, a decrease $\mathrm{RIR}_{\text{-}NO_x}$ coupled with an increase $\mathrm{RIR}_{\text{-AVOCs}}$ indicates a movement towards the more profoundly VOC-limited regime direction. These local OBM-derived trends provide ground-level evidence supporting the regional sensitivity trends and the observed plateau phenomenon discussed in the main text.

Supplementary Note 6 Ground-based data and quality control

Hourly O_3, NO_2, and meteorological data. Nationwide hourly concentration data for surface O_3 and NO_2 in Chinese cities from 2013 to 2024 were acquired from the National Urban Air Quality Real-Time Publishing Platform (https://air.cnemc.cn:18007/, last accessed: 1 March 2025), maintained by the China Ministry of Ecology and Environment (CMEE). Meteorological parameters (temperature, pressure, wind speed, and precipitation) for the same air quality monitoring stations and period were obtained from the China National Environmental Monitoring Centre (CNEMC).

The national monitoring network expanded since its inception in 2013, growing from approximately 450 stations in 74 major cities during the summer of 2013 to over 1600 stations across 367 cities by 2020. This study focused on three megacity clusters, which are focal areas of China's Clean Air Action Plan[31]: the extended North China Plain (NCP; encompassing Beijing-Tianjin-Hebei-Shandong-Henan; 31°–42°N, 112°–123°E; 368 sites), the Yangtze River Delta (YRD; 27°–35°N, 114°–122°E; 223 sites), and the Pearl River Delta (PRD; 21.5°–25°N, 111°–115.5°E; 81 sites). The geographical domains of these regions are delineated in Supplementary Figure 7.

CMEE revised the monitoring of pollutants to a new reference condition (298 K, 101.325 kPa) since 1 September 2018. O_3 and NO_2 measurements were reported in micrograms per cubic meter and the mass concentrations for all years were standardized to the reference state (298.15 K, 1013.25 hPa) in this study. Rigorous data quality control procedures were implemented to identify and exclude unreliable outliers. City-level daily NO_2 and O_3 concentrations were represented by the average of daily mean NO_2 and the daily maximum 8-hour average (MDA8) O_3 concentrations, respectively, across all national monitoring sites within that city.

MDA8 O_3 was defined as the highest 8-hour running average calculated between 00:00 and 23:00 China Standard Time (CST; UTC+8). A site's MDA8 O_3 value was considered missing if data availability was less than 60% (i.e., <5 hours for an 8-hour average or <15 hours for a daily average). For seasonal metrics such as the seasonal average or median MDA8 O_3, values for a site were treated as missing if fewer than 60% of daily MDA8 values were available within that season (i.e., <55 MDA8 values for a 90-day season).

Hourly VOC concentrations. Hourly concentrations of 57 speciated VOCs, encompassing 29 alkanes, 10 alkenes, 1 alkyne, and 17 aromatics, were obtained for the summers (June–August) of 2019–2021 from seven representative cities: Beijing, Tianjin, Xiong'an, Zhengzhou and Jinan within the NCP region, Shanghai in the YRD region, and Guangzhou in the PRD region. In the NCP megacity clusters, where high-quality hourly VOC data were relatively more abundant, the five selected cities represent diverse levels of economic development: Beijing as a highly developed metropolis, Xiong'an as an emerging city, and Tianjin, Zhengzhou and Jinan as moderately developed cities. Data were sourced from the CNEMC, the Shanghai Academy of Environmental Sciences and Guangzhou Ecological and Environmental Monitoring Center, respectively. These VOCs were monitored continuously using commercial automated gas chromatography systems coupled with flame ionization detection and/or mass spectrometry (GC-FID/MS), adhering to stringent quality assurance and control (QA/QC) protocols[32].

Instrumentation and QA/QC procedures. All pollutant monitoring instruments were housed in temperature-controlled indoor environments (maintained at 18–22 °C) to ensure optimal operational stability, particularly for the chromatographic retention times of VOC species. Ambient air was sampled via dedicated inlet ports. O_3, NO_x,

and CO were measured using a UV photometric O_3 analyzer (detection limit: 1.0 ppbv), a chemiluminescence NO*x* analyzer (detection limit: 1.0 ppbv), and a non-dispersive infrared CO analyzer (detection limit: 0.04 ppmv), respectively. O_3 analyzers underwent annual calibration against a Standard Reference Photometer (SRP) traceable to the Institute of Environmental Reference Materials of the MEE. Other gaseous pollutant analyzers were calibrated monthly using certified reference standards. Online VOC instrumentation (GC-FID/MS) included automated internal calibration every 24 hours. Manual external multi-point calibrations were performed routinely using PAMS (Photochemical Assessment Monitoring Stations) standard gas mixtures (Linde Gas, USA). These external calibrations involved a six-point concentration gradient, with each concentration level measured in triplicate. Acceptable calibrations required coefficients of determination (R^2) > 0.99 for calibration curves and relative standard deviations (RSDs) for replicate measurements typically below 10%. All instrument operation, maintenance, data assurance, and quality control procedures conformed to established China environmental protection standards[32,33].

Specific QA/QC criteria were applied to the VOC dataset. Outlier VOC data points were identified and removed if they exhibited abrupt changes (a threefold or greater deviation from temporally adjacent measurements). Inter-compound consistency checks were performed: samples were flagged or excluded if correlation coefficients between isomers (isobutane vs. n-butane; isopentane vs. n-pentane; m/p-xylene vs. o-xylene) fell below 0.8. The concentration ratios of ethane/ethylene, propane/propylene, toluene/benzene ratios are generally greater than 1, otherwise these samples will be deleted.

Supplementary Note 7 Software, map sources and satellite data attribution

Main Fig. 2a and Supplementary Figures 17 – 19 were generated using the NCAR Command Language, NCL (http://dx.doi.org/10.5065/D6WD3XH5). ArcGIS (Esri Inc.) was used only for cartographic layout and figure assembly for Supplementary Figures 1 – 3, Supplementary Figures 6 – 7 and Supplementary Figures 8 – 10. No ArcGIS Online basemap, Esri basemap, Esri administrative-boundary dataset, or third-party map layer from ArcGIS was used. Administrative boundaries shown in the figures were obtained from the Standard Map Service of the Ministry of Natural Resources of the People's Republic of China (http://bzdt.ch.mnr.gov.cn/), with map approval number GS(2024)0650. Official Sentinel-5P/TROPOMI Level-2 offline products were obtained from the Copernicus Data Space Ecosystem (https://browser.dataspace.copernicus.eu/), and the Level-3 (L3) TROPOMI offline products were accessed via the Google Earth Engine (GEE) cloud computing platform (https://earthengine.google.com/). GEE was used only to access and process publicly available TROPOMI Level-3 data products in Supplementary Figure 4; no Google basemap or Google Maps layer was used in the figure. OMI data up to and including 2020 were obtained from the QA4ECV project (http://www.qa4ecv.eu/), and OMI data after 2020 were obtained from NASA Earthdata (https://www.earthdata.nasa.gov/data/).

Supplementary References

1. Wang, T. et al. Ozone pollution in China: a review of concentrations, meteorological influences, chemical precursors, and effects. *Sci. Total Environ.* **575**, 1582–1596 (2017).
2. Zhang, Y. et al. Evolution of ozone formation sensitivity during a persistent regional ozone episode in Northern–Eastern China, and its implication for a control strategy. *Environ. Sci. Technol.* **58***,* 617–627 (2024).
3. Du, X. et al. Modeling of spatial and temporal variations of ozone-NO*x*-VOC sensitivity based on photochemical indicators in China. *J. Environ. Sci.* **114**, 454–464 (2022).
4. Jin, X. et al. Evaluating a space-based indicator of surface ozone-NO*x*-VOC sensitivity over midlatitude source regions and application to decadal trends. *J. Geophys. Res.-Atmos.* **122**, 10439–10461 (2017).
5. He, H. et al. The long-term trend and production sensitivity change in the US ozone pollution from observations and model simulations. *Atmos. Chem. Phys.* **20**, 3191–3208 (2020).
6. Ren, J. et al. Diagnosing ozone–NO*x*–VOC sensitivity and revealing causes of ozone increases in China based on 2013–2021 satellite retrievals. *Atmos. Chem. Phys.* **22**, 15035–15047 (2022).
7. Jin, X. et al. Inferring changes in summertime surface ozone−NO*x*−VOC chemistry over U.S. urban areas from two decades of satellite and ground-based observations. *Environ. Sci. Technol.* **54**, 6518–6529 (2020).
8. Schroeder, J. R. et al. New insights into the column CH_2O/NO_2 ratio as an indicator of near-surface ozone sensitivity. *J. Geophys. Res.-Atmos.* **122**, 8885–8907 (2017).
9. Li, Y. et al. Analysis of ozone formation sensitivity in Chinese representative regions using satellite and ground-based data. *Remote Sens.* **16**, 316 (2024).
10. Wang, W. et al. Spatial and temporal changes of the ozone sensitivity in China based on satellite and ground-based observations. *Atmos. Chem. Phys.* **21**, 7253–7269 (2021).
11. Grange, S. K. et al. Using meteorological normalisation to detect interventions in air quality time series. *Sci. Total Environ.* **653**, 578–588 (2019).
12. Hou, L. et al. Revealing drivers of haze pollution by explainable machine

learning. *Environ. Sci. Technol. Lett.* **9**, 112–119 (2022).

13. Carslaw, D.C. Deweather—An R package to remove meteorological variation from air quality data. https://github.com/davidcarslaw/deweather (2021).
14. Ridgeway, G. gbm—Generalized boosted models. R package. https://CRAN.R-project.org/package=gbm (2017).
15. Choi, Y. et al. Summertime weekly cycles of observed and modeled NO*x* and O_3 concentrations as a function of satellite-derived ozone production sensitivity and land use types over the Continental United States. *Atmos. Chem. Phys.* **12**, 6291–6307 (2012).
16. Duncan, B. N. et al. Application of OMI observations to a space-based indicator of NO*x* and VOC controls on surface ozone formation. *Atmos. Environ.* **44**, 2213–2223 (2010).
17. Koplitz, S. et al. Changes in ozone chemical sensitivity in the United States from 2007 to 2016. *ACS Environ. Au* **2**, 206–222 (2022) .
18. Wang, Y. et al. Sustained emission reductions have restrained the ozone pollution over China. *Nat. Geosci.* **16**, 967–974 (2023).
19. Zheng, B. et al. Trends in China's anthropogenic emissions since 2010 as the consequence of clean air actions. *Atmos. Chem. Phys.* **18**, 14095–14111 (2018).
20. Kurokawa, J. et al. Emissions of air pollutants and greenhouse gases over Asian regions during 2000–2008: Regional Emission inventory in Asia (REAS) version 2. *Atmos. Chem. Phys.* **13**, 11019–11058 (2013).
21. Coats, Jr C. J. & Houyoux, M. R. Fast Emissions Modeling With The Sparse Matrix Operator Kernel Emissions (Smoke) Modeling System. (Air & Waste Management Association, Pittsburgh, 1996).
22. Guenther, A. et al. Model of Emissions of gases and aerosol from nature version 3 (MEGAN3) for estimating biogenic emissions. In *Air Pollution Modeling and its Application XXVI* 36. 187–192. (Springer, 2020).
23. Emery, C. et al. Recommendations on statistics and benchmarks to assess photochemical model performance. *J. Air Waste Manage. Assoc.* **67**, 582–598 (2017).
24. Zhang, Y. et al. An atmospheric vulnerability assessment framework for environment management and protection based on CAMx. *J. Environ. Manage.* **207**, 341–354 (2018).
25. Kong, L. et al. Evaluation and uncertainty investigation of the NO_2, CO and NH_3

modeling over China under the framework of MICS-Asia III. *Atmos. Chem. Phys.* **20**, 181–202 (2020).

26. Du, X. et al. Sensitivity modeling of ozone and its precursors over the Chengdu metropolitan area. Atmos. Environ. **277**, 119071 (2022).
27. Du, X. et al. Improving photochemical indicators for attributing ozone sensitivities in source apportionment analysis. J. Environ. Sci. **143**, 235–246 (2024).
28. Du, X. et al. Responses of ozone concentrations to the synergistic control of NO*x* and VOCs emissions in the Chengdu metropolitan area. Front. Environ. Sci. **10**, 1024795 (2022).
29. Saunders, S. M. et al. Protocol for the development of the Master Chemical Mechanism, MCM v3 (Part A): tropospheric degradation of non-aromatic volatile organic compounds. *Atmos. Chem. Phys.* **3**, 161–180 (2003).
30. Jenkin, M. E. et al. Protocol for the development of the master chemical mechanism, MCM v3 (Part B): tropospheric degradation of aromatic volatile organic compounds. *Atmos. Chem. Phys.* **3**, 181–193 (2003).
31. State Council of the People's Republic of China. Action Plan for Continued Improvement of Air Quality. https://www.gov.cn/zhengce/content/202312/content_6919000.htm (2023).
32. China National Environmental Monitoring Centre (CNEMC). Technical Guide for Automatic Monitoring Data Review of National Atmospheric Photochemical Monitoring Network. http://www.cnemc.cn/jcgf/dqhj/202109/t20210924_953111.shtml (2021).
33. Chinese Ministry of Ecology and Environment (CMEE). Technical Specifications for Installation and Acceptance of Ambient air Quality Continuous Automated Monitoring System for SO_2, NO_2, O_3 and CO. http://www.mee.gov.cn/ywgz/fgbz/bz/bzwb/jcffbz/201308/W020130802493970989627.pdf (2013).
