# Supplementary material for: Aligning climate and air quality goals demands early VOC control for China’s ozone pollution
Source: Nat Commun. 2026 Jul 9;17:9307. doi: 10.1038/s41467-026-75387-w (PMC13530134; doi:10.1038/s41467-026-75387-w)
Supplement: Supplementary file 2 [file 41467_2026_75387_MOESM2_ESM.pdf]

nature portfolio

Peer Review File

Aligning climate and air quality goals demands early VOC control for China's ozone pollution

Corresponding Author: Professor Jian Gao

Parts of this Peer Review File have been redacted as indicated to remove third-party material.

This file contains all reviewer reports in order by version, followed by all author rebuttals in order by version.

Attachments originally included by the reviewers as part of their assessment can be found at the end of this file.

Version 0:

Reviewer comments:

Reviewer #1

(Remarks to the Author)
see the attachment.

Reviewer #2

(Remarks to the Author)
Review: Aligning climate and air quality goals demands early VOC control for China's ozone pollution
Authors: Y. Zhang et al.

Summary: This study combines historical observations of ozone response to NOx/VOC concentrations with future emissions pathways to analyze approaches to reduce O3 concentrations while simultaneously achieving climate action goals. The major conclusion of the study is that early VOC reductions are the most effective pathway to achieve these objectives.

Overall Recommendation: Major revisions required to resolve discrepancy between formaldehyde to NO2 ratios observed by TROPOMI and predicted by models that are then used to evaluate optimal future pathways. See especially comment 5 below.

Comments:

1. The "coupled dynamic trend response" (CDTR) method at the heart of the analysis needs a clearer explanation. It appears that this method simply examines relationships between de-weathered O3 concentrations and either satellite-observed formaldehyde to NOx ratios, or predictions of O3 sensitivity from chemical transport models. A single sentence is used to summarize the method in the main text. The methods section of the paper does not clearly explain the method but rather contains paragraphs describing the benefits of the model and defers any explanation to supplementary text. This lack of clarity makes it difficult for the reader to judge the strengths and weaknesses of the technique and therefore reduces confidence in the conclusions of the study.

2. Assuming the CDTR method is based primarily on historical observations of O3 concentrations and formaldehyde to NOx ratios, it is somewhat uncertain to project these changes into the future where the mix of VOCs may be changing over time, resulting in a shifting transition point in the O3 sensitivity. Likewise, the analysis of the trends during the COVID-19 period is somewhat ambiguous because activity patterns changed significantly, such as when industrial facilities shut down but food cooking and home heating continued, leading to changes in VOC composition, not just VOC amount. The changing nature of VOC composition over time makes it difficult to project past trends to the future.

3. Figure 1 shows that formaldehyde to NOx ratios are increasing over time. This would suggest that the O3 formation chemistry is shifting to a NOx-limited chemical regime, where additional NOx control would reduce O3 concentrations. It is

not clear whether the authors considered the possibility that NOx control programs may be about to cross the ridgeline in the O3 isopleth, meaning that further NOx reductions would reduce O3 concentrations. A clearer analysis of this possibility would increase confidence in the study conclusions that VOC controls are more effective than NOx controls.

4. WRF-CAMx simulations conducted to analyze the effects of various emissions scenarios appear to have been carried out at 36km resolution (see extended data Fig 1). This would appear to be very coarse for a city-specific analysis of ozone trends. The authors should clarify what spatial resolution was used to produce the results in Fig 2.

5. The formaldehyde to NO2 ratios observed by TROPOMI at NCP, YRD, and PRD are in the range of 4-4.5 for the year 2020 (see Fig 1). The formaldehyde to NO2 ratio predicted for these same locations using the WRF-CAMx framework in 2020 are in the range 0.8 – 1.3 (see Fig 4). This calls into question the accuracy of the analysis for the future pathways, since they appear to be starting much deeper in the VOC-limited regime than what measurements would indicate. A more complete validation of the modeling system and the basecase emissions inventory seems warranted given this discrepancy. The overall conclusions of the study remain uncertain without a more complete analysis.

Reviewer #3

(Remarks to the Author)
This study presents an interesting result that the trends in O3 in China are, for the first time since national measurements began in 2013, not increasing between 2021-2023 (an observed plateau). The authors also observe that the traditional drive to a NOx-limited regime may not be the most effective way of reducing O3 long-term, and that reducing VOCs early within a short "VOC window" would be the most effective route.
There are many studies that come to different conclusions on chemical regimes across China, but the work presented here aims to bring together multiple methods and techniques to derive a more definitive decision on the chemical regimes in various locations across the country. Having a more robust method of assessing chemical regime, both in the present day and future, is of great interest to the air quality community, and to policy makers.
Whilst the results are of great interest to the scientific community, the current version of the manuscript does not go into sufficient detail for this work to be reproduced. A new "CDTR" method is described by the authors as "a quantitative tool to compare the efficacy of various control strategies in achieving the desired air quality and climate goals at multidecadal scales and provides a quantitative assessment of the optimal O3 control strategy for the coming decades." However, it is still unclear to me exactly what this is. Is it a new way of calculating trends, if so how? Does it bring together multiple pre-existing methods to determine the chemical regime? If so, how? What is the equation / calculation to make this a quantitative method? If such a technique exists, it would be useful for the reader to be able to reproduce this method. As it currently stands, this is not possible. There is extended discission on what this method is capable of, but not how it works.

Some additional comments:
It is difficult to follow all the different scenarios i.e. "action plan" vs. "plan strengthen VOC" vs. "plan + carbon". It would be helpful to include a table in the manuscript which contains each scenario, and a brief description of how this scenario is unique / what it means from an emissions perspective.
The focus of the VOC reductions is anthropogenic, as emissions of these are easier for policy makers to control. However, it is important that the authors comment on the impact biogenic VOCs may have in the future on their results. How sensitive are the author's results to potential future changes in biogenic VOCs?
Line 316: "The meteorological field and BVOC emission inputs are fixed at the 2020 level". Is this realistic? What is the sensitivity of your study to changes in this?
Line 361: Completely out of the blue, the authors state that further considerations into O3 seasonality suggest that a year-round VOC strengthening is a win-win strategy for China, but I don't really know what this means. There is no additional reference to seasonality in the results, and "VOC strengthening" sounds like VOCs should be increases, which is contradictory to the overall message. The manuscript is very "conclusions" heavy, with not enough attention given to presenting and discussing the data itself.

Reviewer #4

(Remarks to the Author)
Zhang et al. investigate the long-term effectiveness of ozone mitigation strategies in China up to 2060. Focusing on how emission pathways shape the relative benefits of VOC versus NOx controls, the study challenges the conventional NOx-first control paradigm and shows that early VOC reductions are particularly effective for lowering regional ozone. It also critiques the commonly used "sensitivity zoning," which relies on steady-state and short-timescale assumptions, and proposes an alternative method, the "coupled dynamic trend response." Despite addressing a relevant topic, this manuscript requires major revisions before it can be considered for publication.
First, several previous studies have extensively investigated O_3 mitigation strategies. It would therefore be valuable for the authors to clearly highlight the unique findings or novel contributions of this work. Furthermore, the quality of the figures needs significant improvement. Each figure contains multiple subpanels, but they are not labeled with numbers or letters, which makes them difficult to reference in the text. The axes should be clearly defined and large enough to be easily legible, and the figure legends should be enlarged for readability. In addition, some figures include shaded areas, but these are not explained in the figure captions and should be clarified.

Other general comments:
• When defining the emission reduction scenarios, for the “strengthen VOC” scenario is the reduction is set over total VOCs? If so, wouldn’t it be interesting to explore the impact of targeting specific VOC species, particularly the most reactive ones that contribute most to ozone formation, or specific sectors? Several studies in China and elsewhere have highlighted the benefits of reactivity-based mitigation strategies. For example, Ou et al. (2015) and Derwent et al. (2007) demonstrated that focusing on high-OFP species provides more effective ozone control than emission-based approaches. It would be interesting to see the impact when incorporating such an approach, as it could strengthen the narrative of the paper by demonstrating even greater effectiveness of VOC control strategies.

Refs:
Jiamin Ou, Junyu Zheng, Rongrong Li, Xiaobo Huang, Zhuangmin Zhong, Liuju Zhong, Hui Lin, Speciated OVOC and VOC emission inventories and their implications for reactivity-based ozone control strategy in the Pearl River Delta region, China, Science of The Total Environment, Volumes 530–531, 2015, Pages 393-402, ISSN 0048-9697, https://doi.org/10.1016/j.scitotenv.2015.05.062.
R.G. Derwent, M.E. Jenkin, N.R. Passant, M.J. Pilling, Reactivity-based strategies for photochemical ozone control in Europe, Environmental Science & Policy, Volume 10, Issue 5, 2007, Pages 445-453, ISSN 1462-9011, https://doi.org/10.1016/j.envsci.2007.01.005.

• Assuming fixed biogenic VOC (BVOC) emissions up to 2060 represents an important limitation, as BVOCs are strongly temperature-dependent. Under warming scenarios, their contribution to ozone formation is likely to increase, particularly as anthropogenic VOCs are reduced, which could alter the long-term sensitivity of ozone to VOC controls. Could the authors comment on this? Additionally, L.504 the authors mentions that the method relies on dynamic emission changes, it should be clarified that these dynamics apply only to AVOCs.

• Several studies have shown that future increases in CH_4 can significantly influence O_3 production. Could you clarify how CH_4 was treated in your modeling framework? For instance, was CH_4 held constant, or varied according to projected scenarios?

• Model validation for key VOCs is essential when analyzing NO_x–VOC–O_3 chemistry. In the Supplementary Information, you state that “The OBM simulations were constrained by hourly observational data for key atmospheric constituents (O_3, NO_x, CO, VOCs).” If these observational data (e.g., VOC and CO) are available, why are they not used to validate the model performance statistics and included in Supplementary Table 10?

Minor comments:

• Lines 61-63 “This outcome contrasts sharply…”: The statement is quite strong and could be softened or better supported with references. For example, studies in Europe have shown mixed O_3 responses to NOx-targeted controls across different regions.

• Lines 77-79 “This inconsistency…”: This sentence appears to repeat the idea from the previous lines and could be removed to improve conciseness.

• Figure 1: too big (it might be better to split into different figures). Also, in Fig 1c the x-axis does not align between the three regions, which makes the figure confusing. Consider plotting all three regions in the same panel (i.e. sharing the same x-axis) for easier comparison. Additionally, why the PRD region shows earlier trends (increase starting in 2008 versus 2011 for NCP and YRD) and also an earlier decrease?

• Line 259: In the text you are refering to “Methods”, but then the section is named “Online methods”.

• Do you miss the y-axis in Figure 5? Please give more information in caption, this figure is the main figure to describe this study.

In the supplement:

• Lines 221-226: Could you provide additional details on the methodology used to determine the threshold?

• Supplementary Figs 4 and 5: could you use the same range in the y-axis

• Line 302 “Emission data for 2021 were subsequently refined by incorporating the latest local measurements”. Could you elaborate on that?

• Lines 350-353: “Daily average simulated temperature and atmospheric pressure showed high consistency with observations, with r values consistently above 0.75 and 0.97, respectively” and then in Supplementary Table 11, NCP correlation for the temperature is 0.57. Could you check those statistics, please?

Reviewer #5

(Remarks to the Author)
I co-reviewed this manuscript with one of the reviewers who provided the listed reports. This is part of the Nature

Communications initiative to facilitate training in peer review and to provide appropriate recognition for Early Career Researchers who co-review manuscripts.

Version 1:

Reviewer comments:

Reviewer #1

(Remarks to the Author)
I appreciate the authors' thorough response to my comments. The additional data and revised discussion have clarified the previous ambiguities. I have no further concerns and support the publication of this paper in its current form.

Reviewer #2

(Remarks to the Author)
Second Review: Aligning climate and air quality goals demands early VOC control for China's ozone pollution
Authors: Y. Zhang et al.

Summary: This study combines historical observations of ozone response to NOx/VOC concentrations with future emissions pathways to analyze approaches to reduce O3 concentrations while simultaneously achieving climate action goals. The major conclusion of the study is that early VOC reductions are the most effective pathway to achieve these objectives. Authors have successfully revised the manuscript to better explain the CDTR method and they have also performed additional analysis to explore how evolving VOC composition could affect the results. These updates address two of the major issues with the original manuscript, but several key issues remain.

Overall Recommendation: Additional work should be performed to run model calculations at an appropriate spatial resolution for urban studies and to resolve discrepancy between formaldehyde to NO2 ratios observed by TROPOMI and predicted by models.

Comments:
1. Figure 1 shows that formaldehyde to NOx ratios are increasing over time. This would suggest that the O3 formation chemistry is shifting to a NOx-limited chemical regime, where additional NOx control would reduce O3 concentrations. The authors acknowledge this in the revised manuscript but go on to define a target where VOC controls are preferred in the "AVOCs reduction bonus window" where one kg of AVOC reduction is more effective at reducing O3 concentrations than one kg of NOx reduction. This analysis seems overly simplistic since the effort required to reduce AVOC emissions by one kg is not equal to the effort required for each kg of NOx reduction. Emissions control programs typically encounter increasing costs per kg of pollutant reduction since they take advantage of easy controls first. The definition of the "AVOCs reduction bonus window" is overly simplistic and does not account for the increasing cost of AVOCs controls. Since the goal of the manuscript is to map out an optimal O3 control pathway, this issue merits more discussion. It isn't clear to the reader that the recommendation for heavy reliance on AVOC controls through the end of the "AVOCs reduction bonus window" is truly the optimal path. Furthermore, calculating the size of the "AVOC reduction bonus window" requires very accurate modeling tools but significant uncertainties remain in the validation of the WRF/Chem model. See comments below.

2. WRF-CAMx simulations conducted to analyze the effects of various emissions scenarios were carried out at 36km resolution (see extended data Fig 1). This is very coarse for a city-specific analysis of ozone trends. The authors show results from observational based methods based on point measurements made at major cities to build confidence in those model results, but they do not directly compare model predictions to measurements. At best, the current analysis shows that model results and measurements predict qualitatively similar trends for the effectiveness of AVOC vs. NOx controls, but they do not prove that the modeling system is capable of the refined analysis needed for the analysis of "AVOCs reduction bonus windows" that are used in the rest of the analysis. A direct quantitative comparison should be made between model predictions for O3 sensitivity and measurements of O3 sensitivity. This will likely require a nested model approach that scales down to 4km or even 1km resolution over major cities. The WRF/Chem model is perfectly capable of these calculations.

3. The formaldehyde to NO2 ratios observed by TROPOMI at NCP, YRD, and PRD are in the range of 4-4.5 for the year 2020 (see Fig 1). The formaldehyde to NO2 ratio predicted for these same locations using the WRF-CAMx framework in 2020 are in the range 0.8 – 1.3 (see Fig 4). This calls into question the accuracy of the analysis for the future pathways, since they appear to be starting much deeper in the VOC-limited regime than what measurements would indicate. The authors state that this model vs. measurement discrepancy stems from the TROPOMI column measurement vs. the WRF/Chem surface prediction. This explanation is problematic for two reasons. First, the TROPOMI measurements serve as the basis for the observational analysis of O3 sensitivity inherent in the CDTR method. If the satellite measurements are not representative of surface conditions, then how can they be used to diagnose O3 chemical regime at the surface. Second, the WRF/Chem model is a 3D simulation of the atmosphere. The column concentrations of formaldehyde and NO2 can be easily extracted from WRF/Chem results for comparison to TROPOMI results. This critical validation of the modeling system is necessary before the paper can be published, since the accuracy of the model calculations is a critical determinant of the validity of the study conclusions.

Reviewer #3

(Remarks to the Author)
In Aligning climate and air quality goals demands early VOC control for China's ozone pollution, the authors present an interesting study which reveals that O3 trends in China are starting to plateau, as well as highlighting the opportunity for a short "VOC window" in which reducing VOCs would be most effective.

Whilst reducing VOCs now to reduce O3 most effectively is not a new idea, this study brings together multiple different techniques from the literature, to more fully describe the chemical regimes leading to O3 formation in China. A more robust way of bringing together all these techniques, such as the one presented here, is of great interest to the air quality community since using one technique alone can often lead to conflicting or inconsistent results. This study is ambitious. As well as combining multiple analysis approaches, it isn't observation-site-specific, covering multiple megacity clusters across China. Whilst individual techniques employed are not particularly novel, the way they have been combined, coupled with spatial coverage of the study, make for a very interesting manuscript to the air quality community.

The authors have made comprehensive changes to the manuscript in response to my previous reviewer comments, and there is now much more detail in the description of the methodology. I am satisfied that the work presented here is detailed enough for the work to be reproduced and would like to thank the authors for their detailed response and subsequent revisions, leading to a greatly improved manuscript.

Minor correction:
The authors describe policy-driven emission changes across China as a "natural experiment", a term often used to describe phenomena such as the COVID-19 pandemic. I don't think this terminology is quite correct in the context of this study and suggest that this is simply removed.

Reviewer #4

(Remarks to the Author)
The authors have adequately addressed the comments made by the reviewers in the revised version of the manuscript. Therefore, I have no further comments.

Reviewer #5

(Remarks to the Author)
I co-reviewed this manuscript with one of the reviewers who provided the listed reports. This is part of the Nature Communications initiative to facilitate training in peer review and to provide appropriate recognition for Early Career Researchers who co-review manuscripts.

Version 2:

Reviewer comments:

Reviewer #2

(Remarks to the Author)
The authors have adequately addressed my previous comments. I do not completely agree with their interpretation of the study results, but the updated manuscript now contains sufficient information for a knowledgeable reader to draw their own conclusions. I have no further comments and support publication in the current form.

Open Access This Peer Review File is licensed under a Creative Commons Attribution 4.0 International License, which permits use, sharing, adaptation, distribution and reproduction in any medium or format, as long as you give appropriate credit to the original author(s) and the source, provide a link to the Creative Commons license, and indicate if changes were made.
In cases where reviewers are anonymous, credit should be given to 'Anonymous Referee' and the source.
The images or other third party material in this Peer Review File are included in the article's Creative Commons license, unless indicated otherwise in a credit line to the material. If material is not included in the article's Creative Commons license and your intended use is not permitted by statutory regulation or exceeds the permitted use, you will need to obtain permission directly from the copyright holder.
To view a copy of this license, visit https://creativecommons.org/licenses/by/4.0/

A point-by-point response to the reviewer’s

We are very thankful to the reviewers for the valuable comments and thoughtful suggestions. We have carefully addressed all of the review comments and revised the manuscript accordingly. The followings are our point-by-point responses to the comments. The comments of the reviewers are shown in black in *italics*, our responses to the comments are presented in black, and the added or revised texts are provided in blue. As the response letter is quite lengthy, we have added a Table of Contents for your convenience in locating specific comments

Table of Contents

Response to Reviewer #2 2

Response to Reviewer #3 22

Response to Reviewer #4 43

Response to Reviewer #5 78

Response to Reviewer #2

Summary: This study combines historical observations of ozone response to NOx/VOC concentrations with future emissions pathways to analyze approaches to reduce O_3 concentrations while simultaneously achieving climate action goals. The major conclusion of the study is that early VOC reductions are the most effective pathway to achieve these objectives.

Overall Recommendation: Major revisions required to resolve discrepancy between formaldehyde to NO_2 ratios observed by TROPOMI and predicted by models that are then used to evaluate optimal future pathways. See especially comment 5 below.

Reply: We are sincerely grateful for your thorough review and insightful comments on our manuscript. We agree with the reviewer that the $HCHO/NO_2$ ratios of baseline year (2020 in this study) represents a critical link between observational data and model projections, directly impacting the robustness of our future pathway evaluations. We greatly appreciate this opportunity to further clarify our CDTR method and elaborate on the robustness of our conclusions. A detailed response is provided under Comment 5.

Comments:

1. The "coupled dynamic trend response" (CDTR) method at the heart of the analysis needs a clearer explanation. It appears that this method simply examines relationships between de-weathered O_3 concentrations and either satellite-observed formaldehyde to NOx ratios, or predictions of O_3 sensitivity from chemical transport models. A single sentence is used to summarize the method in the main text. The methods section of the paper does not clearly explain the method but rather contains paragraphs describing the benefits of the model and defers any explanation to supplementary text. This lack of clarity makes it difficult for the reader to judge the strengths and weaknesses of the technique and therefore reduces confidence in the conclusions of the study.

Reply: We deeply appreciate the reviewer's constructive comments regarding the clarity and comprehensive explanation of our Coupled Dynamic Trend Response (CDTR) analytical framework. We agree that a clearer, more structured exposition of the CDTR method is crucial for readers to fully grasp its novelty, judge its strengths and weaknesses, and ultimately enhance confidence in our study's conclusions. The reviewer accurately noted the initial brevity in the main text and the over-reliance on supplementary material for methodological details in the original submission.

To address this, we have undertaken substantial revisions to the **Methods** (lines 555-640) and the **Introduction** (lines 92-101) of the main manuscript. Our aim was to provide a comprehensive, step-by-step explanation of the CDTR framework directly within the main text, while ensuring that the supplementary information (SI) still contains all necessary details for reproducibility.

Specifically, in the revised **Methods** section, we have now introduced a dedicated and detailed subsection titled "Coupled Dynamic Trend Response (CDTR) Analysis Framework" (lines 555-640). This section now explicitly defines CDTR as a structured, two-phase analytical approach that integrates multiple established techniques to quantitatively assess the path dependency of O_3 control strategies. By focusing on the co-evolution of the O_3 photochemical system (de-weathered O_3 concentrations and chemical sensitivity) across multi-decadal timescales, this framework identifies optimal control strategies by evaluating its coupled dynamic response to diverse emission reduction strategies (resulting in varying changes in precursor emission reductions). The revised content no longer merely describes its advantages. Each phase of the framework now includes clear, reproducible steps:

- Phase I: Historical validation of pathway dependence

Step 1a: Describes the use of meteorological normalization techniques (GBMs) to isolate emission-driven $MDA8O_3$ concentration trends.

Step 1b: Details the characterization of long-term sensitivity trends using multi-source data (satellite FNR, WRF-CAMx, OBM) and emphasizes robustness validation.

Step 1c: Explains the "coupling" aspect—synchronous analysis of deweathered $MDA8O_3$ concentration and sensitivity trends—to provide real-world evidence for path dependency.

- Phase II: Quantitative prediction of optimal future pathways

Step 2a: Outlines the design of policy-relevant future scenarios and clarifies the role of the integrated DPEC model.

Step 2b: Describes the simulation of synergistic evolution of $MDA8O_3$ concentration and sensitivity trends using WRF-CAMx under fixed meteorological conditions and BVOCs emission.

Step 2c: Explains the identification of optimal pathways through comparative analysis of these simulated trajectories, including the quantification of the duration of the 'AVOCs reduction bonus window' etc..

Each step now provides a concise yet comprehensive overview within the main **Methods**, with precise references to the relevant Supplementary Texts for further in-depth details (e.g., "details in Supplementary Text 3," "details in Supplementary Text 4," etc.). This structured approach clarifies what CDTR is, how it works, and why it is suitable for addressing the research question.

Furthermore, we have revised the "**Introduction**" section to provide a more robust conceptual understanding of CDTR before delving into the detailed methods.

We hope that these extensive revisions can directly address the reviewer's concern. The updated **Methods** section now offers a transparent and reproducible description of the CDTR framework, enabling the readers to critically evaluate our methodology and enhance our findings. We sincerely thank the reviewer for prompting this significant improvement to our manuscript.

The specific revisions are as follows:

Introduction (lines 92-101)

"Here, we address this challenge through the lens of path dependency: early choices in precursor emission controls decisively shape the multi-decadal trajectory of O_3 photochemistry (deweathered concentration and sensitivity), thereby dictating the synergistic achievement of both air quality and climate goals. To capture this critical dynamic, we develop and apply a Coupled Dynamic Trend Response (CDTR) analytical framework (Methods). This approach moves beyond ambiguous sensitivity 'zones' by focusing on the robust, coupled trends of deweathered O_3 concentrations and key sensitivity indicators (e.g., satellite-derived formaldehyde-to NO_2 ratios, FNR) over multidecade timescales. It thus provides a tool to dynamically evaluate how the O_3 photochemical system responds to different emission control pathways."

Methods (lines 555-640**)**

Coupled Dynamic Trend Response (CDTR) Analysis Framework

To address the limitations of traditional static sensitivity zoning methods in formulating effective O_3 mitigation strategies amidst multi-decadal, dynamic changes in precursor emission (including both NO*x* and VOCs), we developed, validated, and applied the CDTR analysis framework. The CDTR is not a novel computational algorithm but a structured, two-phase analytical approach that integrates multiple established techniques to quantitatively assess the path dependence of O_3 control strategies. By focusing on the co-evolution of the O_3 photochemical system (deweathered O_3 concentrations and sensitivity) across multi-decadal timescales, this framework identifies optimal control strategies by evaluating its coupled dynamic response to various emission reduction strategies. The framework comprises the following reproducible steps:

Phase I: Historical validation of pathway dependence.

This phase empirically validates the coupled response of the O_3 photochemical system to policy-driven emission changes, utilizing long-term field observation data as a "natural experiment."

Step 1a: Isolating emission-driven O_3 trends. We applied meteorological normalization techniques, such as Generalized Boosted Models (GBMs)[46], to surface summer O_3 monitoring data (2013–2024) to generate deweathered O_3 concentration time series. This process isolates the signal driven by precursor emission changes by normalizing meteorological effects (details in Supplementary Text 3).

Step 1b: Characterization of long-term sensitivity trends. Using OMI and TROPOMI satellite measurements of O_3 precursors (NO_2 and HCHO) (2005-2024), we analyze the interannual variations of O_3 sensitivity indicators (details in Supplementary Text 4), including the satellite-derived FNR and the area proportion of sensitivity regimes (2005–2024). To validate the robustness of the ‘synchronous plateauing’ phenomenon observed from 2020-2023, sensitivity trends for 2019-2021 were additionally investigated using both a chemical transport model (WRF-CAMx in this study; details in Supplementary Text 5) and an Observation-Based Model (OBM; details in Supplementary Text 6). Results from all three methods consistently revealed a stagnation in the trend of O_3 sensitivity towards NO*x*-limited regimes in either 2019 or 2020. This trend-oriented approach circumvents the inconsistencies inherent in conventional sensitivity zoning methods, which arise from the use of static, absolute

thresholds. Detailed information on sensitivity trends and their robust performance is provided in Supplementary Text 4.

Step 1c: Analysis of drivers of coupled response. "Coupling" refers to the synchronous analysis of deweathered O_3 concentration trends and sensitivity trends, examining their dynamic response to various emission pathways across multi-decadal scales . Interpreting their coupled behavior (e.g., the synchronous plateauing observed from 2020-2023) under the context of historical emission pathways provides real-world evidence for path dependence and validates the framework's capability to timely capture dynamic signals. This framework implies that AVOCs reduction is more effective in the current phase, guiding the design of future quantitative prediction scenarios for Phase II (reinforcing AVOCs control beyond current regulatory strategies).

Phase II: Quantitative prediction of optimal future O_3 mitigation strategy.

This phase applies the framework in a predictive mode, utilizing a chemical transport model (WRF-CAMx) to quantitatively identify optimal long-term control strategies.

Step 2a: Design of policy-relevant scenarios. We designed five future emission reduction scenarios (2020–2060) representing distinct control pathways, based on insights from field observations, current clean air policies, and long-term carbon neutrality targets (details in the following 'Setting scenarios'). The integration of the DPEC model into our scenario design also equips the CDTR analytical framework with the capability for iterative updates, allowing it to dynamically reflect evolving socioeconomic trajectories, advancements in pollution control technologies, and evolving clean air polices and climate actions.

Step 2b: Simulation of synergistic evolution of O_3 concentration and sensitivity trends. For each scenario, we simulated future summer MDA8 O_3 concentrations and FNR values using WRF-CAMx. To isolate the impact of anthropogenic emissions, meteorological conditions and BVOC emissions were fixed at 2020 levels, thereby directly yielding model outputs under identical meteorology (analogous to the deweathered state of field observations).

Step 2c: Identification of optimal strategy via comparative analysis. We plotted the simulated evolution trajectories of MDA8 O_3 concentrations and FNR values under identical meteorological influences for all scenarios from 2020–2060 (as shown in Figure 4a). By quantitatively comparing the coupled response of the O_3

photochemical system across different emission trajectories, we identified the optimal mitigation pathway—one that achieves both near-term national air quality targets and long-term WHO guidelines. This analysis also enabled us to quantify the duration of the 'AVOC reduction bonus window' under the current policy trajectory (details in the 'Quantification method for the duration of AVOCs reduction bonus window' section), thus providing a definitive strategic choice. This comparative analysis constitutes the quantitative core of the CDTR framework.

The application of the CDTR to historical observations, shaped by various implemented clean air policies, revealed its capacity to capture dynamic signals that traditional sensitivity zoning methods miss. These dynamic signals provide qualitative insights for optimising O_3 mitigation. When subsequently applied to future simulations, the CDTR serves as a quantitative tool to compare the efficacy of various control strategies in achieving the desired air quality and climate goals at multidecadal scales and provides a quantitative assessment of the optimal O_3 control strategy for the coming decades. This approach reframes O_3 mitigation from a static problem to an adaptive management process. This adaptability allows projections to be iteratively updated with real-time observational feedback and evolving policy and technologies, ensuring that the framework remains a relevant and robust tool for navigating future air quality and climate challenges.

2. Assuming the CDTR method is based primarily on historical observations of O_3 concentrations and formaldehyde to NOx ratios, it is somewhat uncertain to project these changes into the future where the mix of VOCs may be changing over time, resulting in a shifting transition point in the O_3 sensitivity. Likewise, the analysis of the trends during the COVID-19 period is somewhat ambiguous because activity patterns changed significantly, such as when industrial facilities shut down but food cooking and home heating continued, leading to changes in VOC composition, not just VOC amount. The changing nature of VOC composition over time makes it difficult to project past trends to the future.

Reply: We are sincerely grateful to the reviewer for raising this profound and pivotal point. The reviewer astutely identifies a fundamental challenge in long-term air quality forecasting: the dynamic evolution of VOC speciation and its impact on O_3

photochemistry. We fully concur that this is a critical consideration. In response, we have taken two major actions: (1) we have substantially expanded the **Discussion** section to explicitly address this uncertainty and to highlight how our CDTR framework is designed to accommodate it; and (2) we have performed new, reactivity-based simulations to quantitatively explore this issue. Finally, we further explained the role of the analysis of the trends during COVID-19 period.

(1) On the uncertainty of future VOC speciation and the "adaptive" nature of the CDTR framework:

We fully agree with the reviewer that a simple extrapolation of past trends is insufficient, because the speciation of VOCs will evolve. The primary future scenarios in our study are indeed based on mass-based reductions from the 2020 inventory, a necessary simplification to establish a baseline. However, the core strength of our CDTR framework lie in its **"**adaptability,**"** which was designed specifically to overcome this very limitation that traditional static methods cannot address.

As we now explicitly detail in the revised **Discussion** section (main text, lines 494-495), the CDTR framework "is not a static, one-off prediction tool but a dynamic analytical framework." Its modeling phase is designed to be iteratively updated by incorporating the latest emission inventories (reflecting changes in speciation) and by leveraging dynamic emissions projection models like DPEC, which integrate projections of socioeconomic shifts, advancements in end-of-pipe control technologies, and domain evolving policies. We have clarified this in the revised manuscript:

Revised main text lines 486-503

"Similarly, we acknowledge that the evolving speciation of AVOCs can influence the predicted efficacy of O_3 abatement. Given the differential photochemical reactivities among VOC species[39-41], the strategic focus of reduction—for instance, prioritizing highly ozone formation potential species (high -OFP) like alkenes or aromatics—would undoubtedly influence the duration of the 'AVOC reduction bonus window' and the MDA8 O_3 mitigation effect. Although the emission scenarios in Fig. 4 were simulated by applying mass-based reductions to the 2020 AVOC emission inventory, the inherent adaptability of the our CDTR framework equips it to address the challenge posed by evolving species. It is not a static, one-off prediction tool but a dynamic analytical framework. Its observational

component continuously integrate multi-source real-time data, including satellite and ground-based monitoring, to evaluate the response of deweathered MDA8 O_3 concentrations and sensitivity trends to evolving precursors caused by different policy and activity pattern (e.g. COVID-19). Simultaneously, its modeling component can be iteratively updated by incorporating updated emission inventories and leveraging the DPEC model. The DPEC model is specifically tasked to predict dynamic future emissions by integrating anticipated socioeconomic development, technological advancements, and evolving clean air and climate policies into the CDTR analytical framework (see Methods). "

(2) Quantitative exploration of reactivity impacts:

To provide quantitative evidence on the impact of evolving VOC speciation, we designed new reactivity-based reduction tests. In these tests, we completely removed highly reactive AVOC groups (i.e., alkenes or aromatics) and compared the results to equivalent total-mass reduction scenarios. These new results, presented in the revised manuscript and new Supplementary Figs. 7-8, confirm the significant influence of VOC composition. This analysis, as we now state, "This outcome underscores the adaptability of the CDTR framework, enabling it to continuously assess and refine the optimal control pathway as real-world atmospheric composition evolves."

The specific revision is as follows:

Revised main text lines 503-533:

"To illustrate this, we employed the CDTR framework to further explore the impacts of reactivity-based AVOC control strategies. We calculated precursor reduction ratios of prioritizing completely removal of high-OFP species (alkenes and aromatics), and then compared the outcomes with those from the mass-based AVOC reductions control strategies with equal-proportion reduction of total AVOCs (see Methods, Supplementary Figs. 8–9, and Supplementary Tables 9–11). The results indicate that prioritizing high-OFP species significantly accelerates the evolution of O_3 sensitivity towards the NO_x-limited regime, as evidenced by markedly higher FNR values across all scenarios compared to equal-proportion reduction (Supplementary Fig. 8). However, this did not uniformly translate into superior O_3 concentration mitigation benefits (Supplementary Fig. 9). Specifically, only under the 'plan+carbon' scenario, which involves deep NO_x reductions, did this strategy show a clear advantage in reducing MDA8 O_3 concentrations in the NCP and YRD. Conversely, in scenarios with less aggressive NO_x control, its efficacy was even inferior to that of

the mass-based control strategy. In the PRD megacity cluster under the 'plan+carbon' scenario, the MDA8 O_3 mitigation benefit of reactivity-based reduction proved less effective than that of the mass-based reduction, potentially due to the PRD entering the O_3 formation transition regime later than the other two megacity clusters (Supplementary Fig. 7c). The varying effectiveness of the reactivity-based reduction strategy reveals a strong path dependency, quantitatively demonstrating that the efficacy of control strategies is highly contingent on the evolutionary stage of the O_3 photochemical system. In the early stages of the AVOC reduction bonus window (Fig. 5), the concentrated reduction of high-OFP species may be less efficient than the broad reduction of a larger mass of various AVOCs. Therefore, these findings do not negate the value of reactivity-based strategies but rather underscore the necessity of precision timing in their implementation. This validates the adaptive nature of our proposed CDTR framework: it provides an essential dynamic diagnostic capability to assess the state of the photochemical system in time, thereby determining the optimal timing to transition from mass-based control to more refined, reactivity-based control, ensuring scientific and efficient policy adjustments."

Furthermore, regarding the reviewer's point on the ambiguity of the COVID-19 period: We agree the period was complex, involving significant and differentiated changes in activity patterns and VOC speciation. However, our study leverages this period as a large-scale "natural experiment" to validate the fundamental capability of the CDTR framework. It demonstrates that the framework can capture dynamic responses to complex emission evolutions, even amidst the unique confluence of events during the COVID-19 lockdown and the implementation of new clean air strategies—a capability inherently beyond the scope of traditional, static sensitivity-zoning methods. We interpret the synchronously observed plateau period (2020-2023) during this unique time as powerful, real-world empirical evidence for the principle of path dependency. This field-observation-based validation strengthen our confidence in applying the CDTR framework to future scenarios (or other regions), where emission evolutions will likewise involve dynamic speciation and policy-driven shifts, as such dynamic signals can be captured in time by the observational phase of the CDTR framework.

In conclusion, we are very grateful for the reviewer's comments, which have prompted us to more clearly articulate the adaptive capacity of the CDTR framework

and to reinforce the robustness of our manuscript's conclusions with new reactive-based AVOC control strategies tests. We hope these revisions have fully addressed the reviewer's legitimate concerns and have significantly enhanced the quality of our manuscript.

3. ***Figure 1 shows that formaldehyde to NOx ratios are increasing over time. This would suggest that the O_3 formation chemistry is shifting to a NOx-limited chemical regime, where additional NOx control would reduce O_3 concentrations. It is not clear whether the authors considered the possibility that NOx control programs may be about to cross the ridgeline in the O_3 isopleth, meaning that further NOx reductions would reduce O_3 concentrations. A clearer analysis of this possibility would increase confidence in the study conclusions that VOC controls are more effective than NOx controls.***

Reply: We are profoundly grateful for this valuable comment from the reviewer, which prompted us to delve deeper into the findings of our study. We fully agree that understanding the crossing of the O_3 isopleth 'ridge line' and the subsequent NO*x*-limited regime is crucial for drawing robust conclusions regarding emission reduction strategies.

Our revised manuscript explicitly delineates two critical turning points in the evolution of the O_3 photochemical system to address this point, as detailed in the **Results** section, particularly in the paragraphs describing Fig. 5 and Supplementary Fig. 7 (lines 338-355).

We define the 'first turning point' as 'the crossing of the O_3 isopleth 'ridge line,' which marks the transition from a VOC-limited regime where NO*x* reductions lead to disbenefits, to a 'transition regime' where both AVOC and NO*x* reductions contribute to O_3 reduction.' Our projections indicate that the NCP and PRD megacity clusters have already crossed this ridge line into the transition regime before 2025, with the YRD following shortly thereafter (Supplementary Fig. 7 provides additional details). This explicitly suggests that for these regions, NO*x* reductions indeed contribute to O_3 mitigation.

Crucially, our study goes further to identify a 'second turning point,' which we term the closure of the 'AVOCs reduction bonus window.' This juncture is defined as

'the moment when the O_3 reduction benefits from NO*x* control surpass those from equiproportional AVOC reductions (i.e., O_3 sensitivity becomes the NO*x*-limited regime).' Our analysis in Fig. 5 quantitatively illustrates the dynamic evolution of this 'bonus window,' demonstrating that while a complete transition to a NO*x*-limited regime is an unavoidable long-term outcome under climate and clean air policies for all megacity clusters, there exists a critical, decades-long opportunity window for AVOCs reduction prior to this. During this period, prioritizing deeper AVOCs reductions will yield substantial and rapid O_3 mitigation benefits than that of NO*x*. For instance, the AVOCs control 'bonus window' in the PRD is projected to close earliest, around 2045, followed by the YRD around 2050, while the NCP continues to exhibit a sustained, albeit gradually diminishing, AVOCs control benefit beyond 2050. This quantitative comparison, achieved by separating the respective benefits of equiproportional AVOC and NO*x* reductions, is further elaborated in the Methods section under 'Quantification of AVOCs reduction bonus window duration' (lines 742-780) and visually presented in Supplementary Fig. 7.

Therefore, our revised manuscript provides a more detailed and explicit elucidation of the evolution considering both the ridge line crossing and the transition towards NO*x*-limited conditions. Our conclusion regarding the optimality of early AVOCs control is underpinned by this detailed dynamic analysis, emphasizing the existence and duration of the 'AVOCs reduction bonus window.' We believe that this comprehensive analysis of both turning points significantly bolsters the robustness and confidence in our study's conclusions. We once again thank the reviewer for prompting us to highlight these crucial aspects more clearly.

The specific revisions are as follows:

Lines 338-355

To elucidate the dynamics of this window and its underlying photochemistry, it is essential to distinguish between two critical tipping points. The first tipping point is the crossing of the O_3 isopleth 'ridgeline', marking the transition from the NO*x*-disbenefit regime to a VOC-NO*x* transition regime, where reductions in both precursors effectively lower O_3 concentrations. The second tipping point, which signals the closure of our 'bonus window', is the moment when the O_3 reduction benefit from NO*x* control surpasses that from an equivalent fractional reduction in AVOCs (NO*x*-limited regime). Our projections (Fig. 5 and Supplementary Fig. 7) reveal that China's megacity clusters cross these tipping points asynchronously. The

NCP and PRD have already crossed the 'ridgeline' by 2025 , entering the transition regime, while the YRD follows suit later (Supplementary Fig. 7c). More critically, the 'bonus window' for AVOC control is projected to close earliest for the PRD around 2045, followed by the YRD around 2050. In contrast, the NCP region maintains a persistent, though diminishing, AVOC control bonus beyond 2050, indicating a more prolonged window of opportunity (Fig.5). This quantitative comparison is made possible by paired scenarios to disentangle the respective contributions of equiproportional AVOC and NO*x* reductions on MDA8 O_3 mitigation (see Methods and Supplementary Fig.7 for detailed derivation).

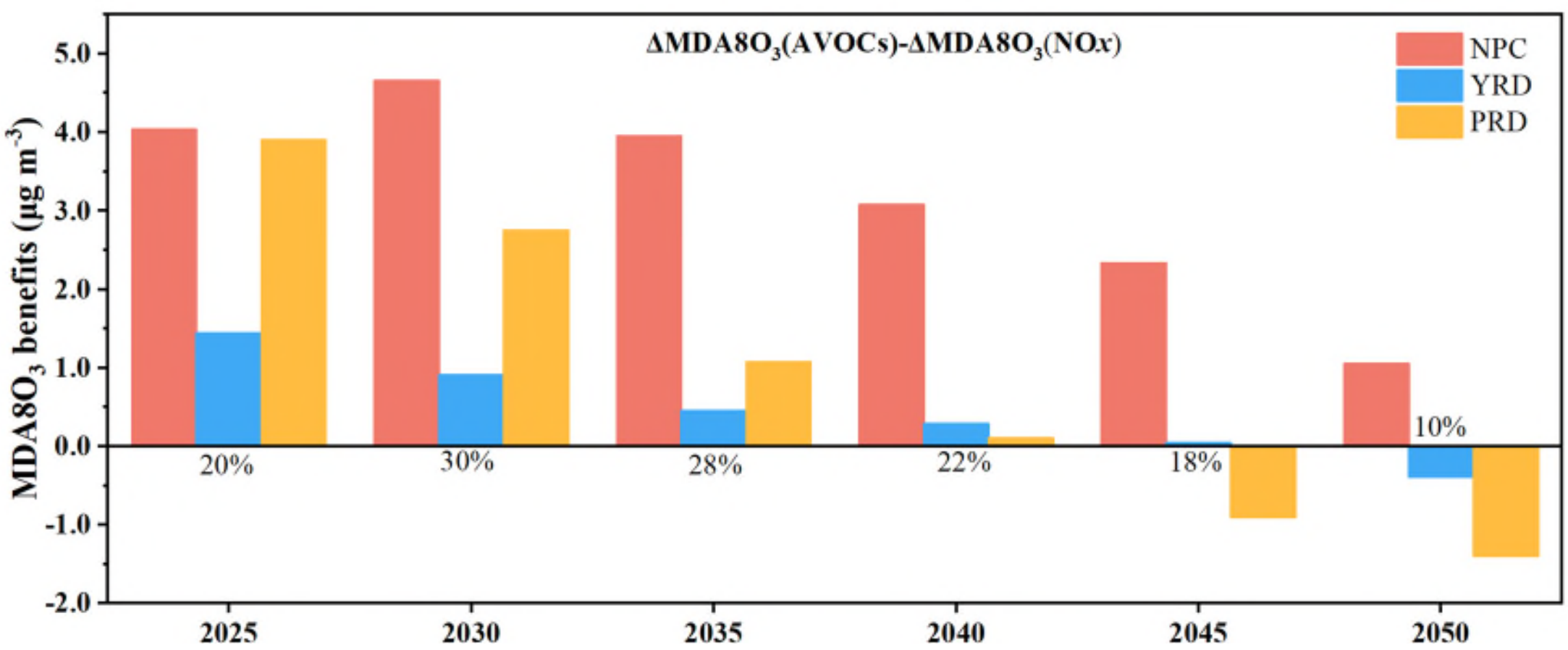

Fig. 5 | Dynamics of the AVOC reduction bonus window in China's three megacity clusters. The bonus window for prioritizing AVOCs is considered closed when its net MDA8O_3 mitigation benefit—defined as the difference in O_3 reduction between reduction AVOCs versus an equivalent proportion of NO*x*—becomes negative. Percentages denote the precursor reduction fraction for the corresponding year.

Lines 742-780

Quantification method for the duration of the AVOCs reduction bonus window

To quantitatively assess the duration 'bonus window' of a prioritized AVOCs control strategy, we calculated the difference in O_3 reduction efficacy resulting from equivalent proportional reductions of AVOCs versus NO*x* (Fig. 5). This calculation is based on a comparative analysis of simulation results from three scenarios, whose O_3 photochemical system evolutionary trajectories are shown in Supplementary Fig. 7a. Specifically, the 'plan+carbon' scenario shares identical AVOC reduction trajectory

with the ‘action plan’ scenario but implements greater NO*x* reductions. Therefore, subtracting the ‘plan+carbon’ scenario from that of the ‘action plan’ scenario isolates the MDA8 O_3 reduction attributable to the additional NO*x* abatement in that year . Conversely, the ‘carbon strengthen AVOCs’ scenario maintained the same NO*x* reduction targets as the ‘plan+carbon’ scenario but implements greater AVOC reductions. Thus, subtracting the ‘carbon strengthen AVOCs’ scenario from that of the ‘plan+carbon’ scenario isolates the MDA8 O_3 reduction attributable to the additional AVOC abatement. The 'AVOCs reduction bonus window' closes when the MDA8 O_3 mitigation benefit from equiproportional NO*x* reduction exceeds that of AVOCs reduction. We quantify this by subtracting the MDA8 O_3 benefit caused by more AVOCs reduction (Extended Data Fig. 2b) from those under the parallel more NO*x* reduction (Extended Data Fig. 2c). The point at which this difference (Fig. 5) becomes negative marks the transition to a NO*x*-limited regime, where NO*x* control becomes the more effective strategy.

This derivation, explicitly illustrated in Supplementary Fig. 7 and Fig. 5, involves the following three steps:

1. Isolating the O_3 reduction benefit from NO*x* reduction: We subtracted the ‘plan+carbon’ scenario from those of the ‘action plan’ scenario. This isolates the net MDA8 O_3 reduction solely attributable to the additional NO*x* abatement (Supplementary Fig. 7b). The percentages shown in the figure represent the proportion of additional NO*x* reduction for that year.

2. Isolating the O_3 reduction benefit from AVOCs reduction: We subtracted the ‘carbon strengthen AVOCs’ scenario from those of the ‘plan+carbon’ scenario. This difference represents the net MDA8 O_3 reduction solely attributable to the enhanced AVOC control measures (Supplementary Fig. 7c). The percentages shown in the figure represent the proportion of additional AVOC reduction for that year.

3. Calculating the difference in benefits: Finally, we subtracted the O_3 reduction benefit derived from AVOC control (result of Step 2) from the benefit derived from NO*x* control (result of Step 1). The resulting value, ΔMDA8 O_3(AVOCs) − ΔMDA8 O_3(NO*x*), represents the net advantage of prioritizing an equivalent reduction in AVOCs over NO*x*—the ‘bonus’. This final time series constitutes the data presented in Fig. 5 of the main text.

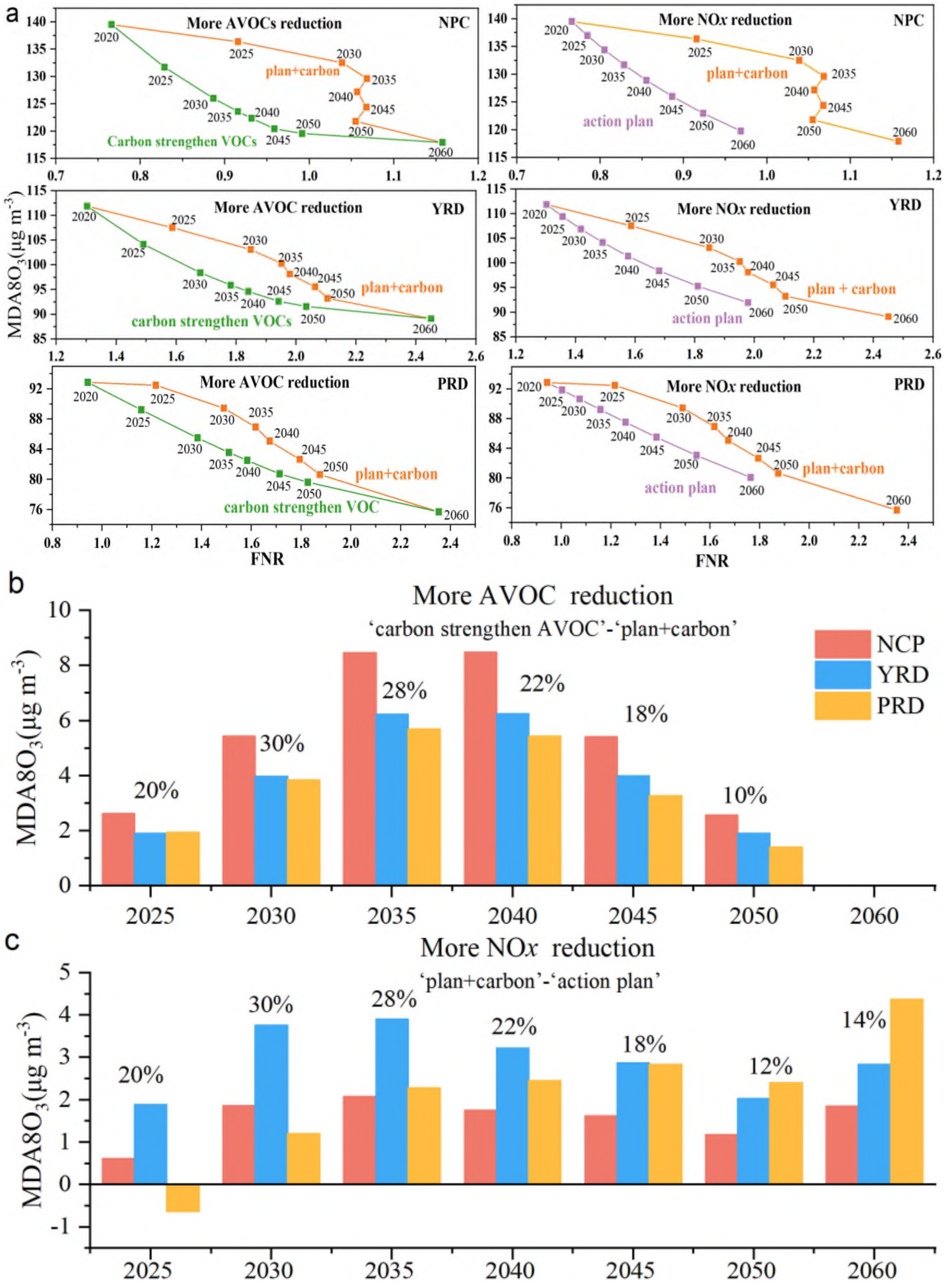

Supplementary Fig. 7. Derivation of isolated O_3 reduction benefits from AVOC versus equivalent proportional NO*x* reduction. a, the evolution trajectory of O_3 photochemical system (O_3-FNR) of paired-scenario designed to decouple the effects of AVOC versus NO*x* reductions in China's three megacity clusters from 2020 to 2060. The 'More AVOCs reduction' comparison isolates the impact of AVOCs by comparing the 'carbon strengthen AVOCs' and 'plan+carbon' scenarios,

which share an identical NO*x* trajectory. The 'More NO*x* reduction' comparison isolates the impact of NO*x* by comparing the 'plan+carbon' and 'action plan' scenarios, which share an identical AVOC trajectory. **b, Isolated MDA8O_3 reduction from dedicated AVOC controls**, calculated as the MDA8O_3 difference between the 'carbon strengthen AVOCs' and 'plan+carbon' scenarios. Percentages indicate the additional AVOC reduction for that year. **c, Isolated MDA8 O_3 reduction from dedicated NO*x* controls**, calculated as the MDA8 O_3 difference between the 'plan+carbon' and 'action plan' scenarios. Percentages indicate the additional NO*x* reduction for that year.

4. WRF-CAMx simulations conducted to analyze the effects of various emissions scenarios appear to have been carried out at 36km resolution (see extended data Fig 1). This would appear to be very coarse for a city-specific analysis of ozone trends. The authors should clarify what spatial resolution was used to produce the results in Fig 2.

Reply: We are thankful to the reviewer for the comment regarding the resolution of our WRF-CAMx simulations and its applicability to city-scale analysis. We fully concur with the reviewer that a 36 km resolution is indeed too coarse for a robust city-specific trend analysis. This ambiguity stems from the unclear explanation of the results from two distinct yet complementary modeling approaches of Fig. 2. We have now thoroughly clarified in the revised manuscript.

Specifically:

(1) Fig. 2a (WRF-CAMx Simulation Results): The regional sensitivity maps shown in Fig. 2a were indeed generated using the WRF-CAMx model at a 36 km resolution. This resolution is appropriate and widely used for assessing regional-scale (e.g., megacity cluster) photochemical regimes and their spatial patterns, which was the objective of our analysis.

(2) Fig. 2b (OBM Simulation Results): The city-scale trend analysis, which the reviewer identifies as necessary, was conducted using an Observation-Based Model (OBM), with results presented in Fig. 2b. These panels show the Relative Incremental

Reactivity (RIR) values for O_3 precursors (NO_x, AVOC, BVOC) in selected typical cities. Unlike the spatially gridded WRF-CAMx, the OBM is an *in-situ* point-based analytical tool that investigates O_3 formation chemistry and sensitivity at urban sites, driven by hourly ground-based observations. The results in Fig. 2b therefore effectively complement the regional perspective from the WRF-CAMx simulations, thereby achieving a multi-scale validation.

To preclude any future ambiguity, we have revised the manuscript to explicitly delineate the distinct roles of the modeling tools used for Fig. 2a and Fig. 2b. These clarifications are now integrated into:

- The main text (Lines 180–188).
- The figure caption for Fig. 2.
- Detailed, separate descriptions of the WRF-CAMx and OBM methodologies in Supplementary Text 5 and Supplementary Text 6.

We are confident that these clarifications fully address the reviewer's concerns and enhance the methodological transparency of our manuscript.

The specific revisions are as follows:

Lines 180-188

Additionally, to complement the regional-scale satellite and model analyses, and to resolve the trends in O_3 sensitivity at the urban-core scale—a domain where satellite observation and CTM simulation have known limitations[15]—we employed an Observation-Based Model (OBM, detailed in Supplementary Text 6). Constrained by in-situ hourly measurements of VOCs and NO_x (Supplementary Text 2), the model diagnoses sensitivity via relative incremental reactivity (RIR). This analysis (Fig. 2b) was conducted in seven cities, selected from the three megacity clusters (Supplementary Fig. 1), possessing robust data foundations, and representing diverse economic development levels in China.

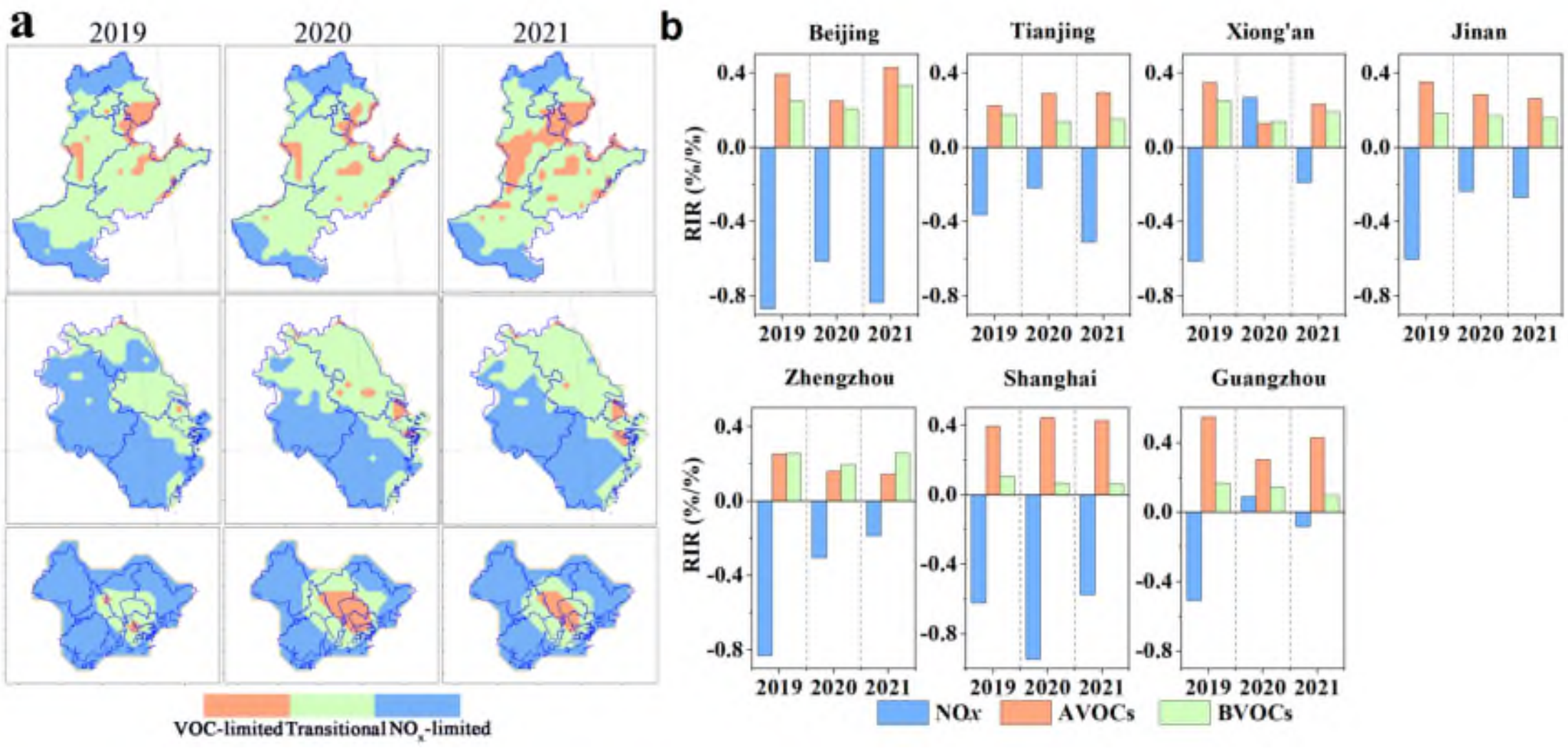

Fig. 2. Multi-source evidence confirms the robustness of the sensitivity trend stagnation . a, Maps illustrating O_3 formation regimes across the three megacity clusters (NCP, YRD and PRD from top to bottom) from 2019–2021 investigated by the WRF-CAMx model (the simulation resolution is 36km). **b,** relative increment reactivity (RIR) calculated by OBM model for O_3 precursors (specifically, NO_x, AVOC, and Biogenic VOC (BVOC)) of typical cities during the summers of 2019-2021.

5. The formaldehyde to NO_2 ratios observed by TROPOMI at NCP, YRD, and PRD are in the range of 4-4.5 for the year 2020 (see Fig 1). The formaldehyde to NO_2 ratio predicted for these same locations using the WRF-CAMx framework in 2020 are in the range 0.8 – 1.3 (see Fig 4). This calls into question the accuracy of the analysis for the future pathways, since they appear to be starting much deeper in the VOC-limited regime than what measurements would indicate. A more complete validation of the modeling system and the basecase emissions inventory seems warranted given this discrepancy. The overall conclusions of the study remain uncertain without a more complete analysis.

Reply: We sincerely thank the reviewer for this insightful and critical comment on the discrepancy between the absolute FNR values from TROPOMI observations (Fig. 1) and our WRF-CAMx model predictions (Fig. 4) for the 2020 base year. This is a well-documented challenge within the atmospheric chemistry community, with multifaceted origins, including differences in vertical sensitivity (satellite total column vs. model surface layer), inherent uncertainties in emission inventories, and potential

biases in satellite retrieval algorithms or model chemical mechanism descriptions (detailed in Supplementary Text.1 lines 51-65). The primary reason for the higher TROPOMI-based FNR compared to the modeled FNR in 2020 is that the column-based FNR (Fig. 1) represents a tropospheric column-integrated value, whereas the modeled FNR (Fig. 4) reflects concentrations at the surface. Furthermore, as detailed in Supplementary Text 4 (lines 281-282), observation-based FNR thresholds used to delineate sensitivity regimes typically yield higher $HCHO/NO_2$ values than model-derived thresholds.

Indeed, this very inconsistency served as a primary motivation for the development and application of our "Coupled Dynamic Trend Response (CDTR)" analytical framework. As detailed in Supplementary Text 1 and 4, employing static, absolute FNR thresholds for "sensitivity zoning" is fraught with significant uncertainty and has led to inconsistent, even contradictory, conclusions in the literature regarding regional O_3 sensitivity in China during summer.

Our framework deliberately circumvents the pitfalls of relying on absolute FNR values by focusing on more robust metrics: relative evolutionary trends and the comparative analysis of different control pathways. The validity of our core arguments and conclusions rests upon two key principles that are unaffected by the absolute FNR value in the base year:

(1) Robustness of Trends: Despite differences in absolute FNR values, our multi-method cross-validation (Fig. 2) consistently captures the same fundamental trend across various methods (satellite, WRF-CAMx, OBM)—specifically, the observed stagnation in the evolution towards NO*x*-limited regimes around 2020. This convergence in trend dynamics validates the robustness of our approach in capturing the dynamic response of the photochemical system.

(2) Robustness of Comparative Analysis: Our future projections (Figs. 4, 5) are not derived from the absolute FNR value of any single scenario but from the quantitative comparison between scenarios. All scenarios originate from the same model baseline. Therefore, the differential O_3 abatement efficacy between pathways and the relative shifts in FNR trajectories are robust. Our identification of the "AVOC reduction bonus window" is based on the relative effectiveness of equivalent proportional precursor reductions（Fig.5，Supplementary Fig.7，Table 2）, which is a differential calculation inherently insensitive to the initial absolute values.

To explicitly address this pivotal point and enhance the methodological transparency of our manuscript, we have added a dedicated paragraph in the revised Discussion section (now lines 440-463). This new text directly confronts the model-observation divergence and reinforces the scientific rationale for our trend-based CDTR framework.

The WRF-CAMx modeling system employed in this study is not a temporary construction but a mature and stable platform developed, tested, and applied extensively by our research team. Beyond the robust statistical evaluation for MDA8 O_3, daily mean NO_2 concentrations, and meteorological fields in 2020, which indicates its good performance (Supplementary Text 5, lines 377-433: Model performance evaluation), the system's stability and reliability have been thoroughly validated in numerous prior publications by our group (Supplementary References 3,31-33). The detailed information about the MEIC and DPEC and their prior publications can be available at http://www.meicmodel.org/

In the revised manuscript, we explicitly acknowledge the discrepancy in absolute FNR values at the joint year (2020) between field observations and model simulations within the CDTR analytical framework. We leverage this discrepancy to further elucidate the innovativeness of the CDTR framework and the robustness of its derived conclusions. Our conclusions are not undermined by model baseline bias; on the contrary, our research stems from an analytical method specifically designed to withstand such uncertainties. We hope this revision fully addresses the reviewer's pertinent concern. We sincerely thank the reviewer for prompting us to make this important clarification.

The specific revisions are as follows:

Supplementary Text.1 lines 51-65

OBM, for instance, offers accurate in situ diagnoses of O_3 sensitivity at specific locations and times. Nevertheless, their application is limited in spatial and temporal extent due to the stringent requirement for comprehensive, simultaneous hourly observational data for key species (e.g., O_3, NO_x, CO, VOCs) and related parameters (e.g., JNO_2, temperature, humidity, pressure), which are hardly available over large domains or long periods[2]. CTM provide descriptions across broader spatial and temporal scales. However, uncertainties primarily come from anthropogenic and biogenic emission inventories, as well as the representation of photochemical reaction mechanisms[3]. The satellite-based method can provide globalscale photochemical

information with good time continuity and little human disturbance. Yet, their reliance on FNR thresholds to distinguish O_3 formation regimes introduces a crucial challenge. The satellite thresholds were reported to be vary with the different regions, periods, and O_3 pollution severity, even using the same determination methods. And these different thresholds yield different sensitivity classifications for the same spatial and temporal conditions[4-10].

Supplementary Text 4, Lines 281-282

Observationally derived thresholds for the transitional regime often yield higher $HCHO/NO_2$ values compared to model-derived thresholds.

Discussion （Lines 440-463）

Notably, FNR values derived from different methodologies often exhibit significant discrepancies[13-18]. For instance, satellite column-based FNR in 2020 (Fig. 1) is substantially higher than that simulated at the surface level by the WRF-CAMx model in this study (Fig. 4). Even when employing the same method, the FNR thresholds used for sensitivity zoning are inconsistent across the literature (see Supplementary Text 1). Such variability necessitates caution when using absolute FNR values as sensitivity indicators. We posit that the reliance on such absolute thresholds for static 'sensitivity zoning' is a fundamental reason for the inconsistent and even contradictory conclusions regarding summer regional O_3 sensitivity in China. Our CDTR framework circumvents this methodological challenge by focusing on the more robust metric of relative evolutionary trends in sensitivity (robustness detailed in Supplementary Text 4). This approach dynamically resolves the evolving efficacy of different precursor control pathways by aligning them with the sensitivity evolution direction and pace, thereby providing more robust and adaptive policy insights. For example, the observed synchronous plateau provides an empirical directive that prioritising AVOC control is the more effective strategy at present and guides the intensification focus of our future scenarios. Crucially, our simulations (Fig. 4) quantitatively demonstrate that O_3 mitigation efficacy is not directly correlated with the speed of transition towards a NO*x*-limited regime (the 'only dual carbon' scenario in Fig. 4a), thereby challenging the traditional paradigm that advocates for the fastest possible entry into NO*x*-limited conditions. Ultimately, we define the closure of the

'bonus window'—the tipping point into a NO*x*-limited regime—by comparing the O_3 abatement efficacy of equivalent proportional reductions in AVOCs versus NO*x* (Fig. 5).

Response to Reviewer #3

1. This study presents an interesting result that the trends in O_3 in China are, for the first time since national measurements began in 2013, not increasing between 2021-2023 (an observed plateau). The authors also observe that the traditional drive to a NOx-limited regime may not be the most effective way of reducing O3 long-term, and that reducing VOCs early within a short "VOC window" would be the most effective route.

There are many studies that come to different conclusions on chemical regimes across China, but the work presented here aims to bring together multiple methods and techniques to derive a more definitive decision on the chemical regimes in various locations across the country. Having a more robust method of assessing chemical regime, both in the present day and future, is of great interest to the air quality community, and to policy makers.

Reply: We sincerely thank the reviewer for this encouraging summary and positive assessment of our work's significance. We agree that the observed plateau, the early VOCs reduction within a short "VOC window", and the introduction of a more robust framework for assessing O_3 control strategy are key contributions of this study. Our aim is indeed to provide a more definitive and actionable understanding for the air quality community and policymakers, especially in the context of dynamic emission changes driven by synergistic climate and air quality goals.

1.Whilst the results are of great interest to the scientific community, the current version of the manuscript does not go into sufficient detail for this work to be reproduced. A new "CDTR" method is described by the authors as "a quantitative tool to compare the efficacy of various control strategies in achieving the desired air quality and climate goals at multidecadal scales and provides a quantitative

assessment of the optimal O_3 control strategy for the coming decades." However, it is still unclear to me exactly what this is. Is it a new way of calculating trends, if so how? Does it bring together multiple pre-existing methods to determine the chemical regime? If so, how? What is the equation / calculation to make this a quantitative method? If such a technique exists, it would be useful for the reader to be able to reproduce this method. As it currently stands, this is not possible. There is extended discission on what this method is capable of, but not how it works.

Reply: We deeply appreciate the reviewer's positive recognition of our research findings and the critical and constructive feedback regarding the Coupled Dynamic Trend Response (CDTR) analytical framework. We fully agree that methodological reproducibility is fundamental to any scientific study. The reviewer noted that the current manuscript's description of the CDTR framework was insufficiently detailed for reproducibility, and we have made significant revisions to address this.

We understand the reviewer's questions regarding the working principles of the CDTR framework. In the revised manuscript, particularly in the **Methods** section, we have substantially expanded and structured the description of the CDTR framework (Methods, lines 555-640). Herein, we provide further clarification:

(1) The Essence of the CDTR Framework – "Is it a new method for calculating trends？"

The CDTR framework is not a novel trend calculation algorithm but rather a structured two-stage analytical framework that strategically integrates multiple established and validated techniques to quantitatively assess the path dependency of O_3 control strategies (Methods, lines 559-562). Its core lies in analyzing the coevolution of deweathered O_3 concentration trends and O_3 sensitivity trends over multi-decadal scales. The initial stage of the CDTR framework—the field observation component involve: Deweathered O_3 concentration trends are calculated using Generalized Boosted Models (GBMs) (Supplementary Text 3), while sensitivity trends are characterized through an integrated analysis of satellite remote sensing data (FNR), chemical transport models (WRF-CAMx), and observation-based box models (OBM) (Supplementary Text 4-6). These are all recognized and reproducible methodologies. The second stage of the CDTR framework, the quantitative projection of optimal future pathways, is performed using WRF-CAMx model simulations.

Methods, lines 559-562

"The CDTR is not a novel computational algorithm but a structured, two-phase analytical approach that integrates multiple established techniques to quantitatively assess the path dependence of O_3 control strategies. By focusing on the co-evolution of the O_3 photochemical system (de-weathered O_3 concentrations and sensitivity) across multi-decadal timescales, this framework identifies optimal control strategies by evaluating its coupled dynamic response to various emission reduction strategies."

(2) How the CDTR Framework Integrates Existing Methods to Determine Chemical Sensitivity Regimes – "Does it combine multiple pre-existing methods to determine the chemical regime?" and "What is the equation / calculation to make this a quantitative method?"

Yes, in Phase I (historical path dependency validation), the CDTR framework strategically integrates three independent O_3 sensitivity diagnostic methods (satellite FNR, WRF-CAMx model simulations, and OBM) to cross-validate the robustness of O_3 sensitivity trends (Methods, lines 567-599 and Figs. 1, 2). Crucially, the innovation of the CDTR framework is that it transcends the reliance on static absolute thresholds in traditional "sensitivity zoning," focusing instead on the relative evolution trends of sensitivity (Discussion, lines 446-453). We found that while the absolute classification of sensitivity regimes may differ across methods, they consistently demonstrate high agreement in their sensitivity evolution trends (Fig. 2a, Supplementary Figs. 2-4 and Supplementary Table 8). This is key to the CDTR framework's ability to capture dynamic signals "missed" by traditional methods (lines195-200).

Methods, lines 567-599

"Phase I: Historical validation of pathway dependence.

This phase empirically validates the coupled response of the O_3 photochemical system to policy-driven emission changes, utilizing long-term field observation data as a "natural experiment."

Step 1a: Isolating emission-driven O_3 trends. We applied meteorological normalization techniques, such as Generalized Boosted Models (GBMs)[46], to surface summer O_3 monitoring data (2013–2024) to generate "de-weathered" O_3 concentration time series. This process isolates the signal driven by precursor emission changes by normalizing meteorological effects (details in Supplementary Text 3).

Step 1b: Characterization of long-term sensitivity trends. Using OMI and TROPOMI satellite measurements of O_3 precursors (NO_2 and HCHO) (2005-2024), we analyze the interannual variations of O_3 sensitivity indicators (details in Supplementary Text 4), including the satellite-derived FNR and the area proportion of sensitivity regimes (2005–2024). To validate the robustness of the ‘synchronous plateauing’ phenomenon observed from 2020-2023, sensitivity trends for 2019-2021 were additionally investigated using both a chemical transport model (WRF-CAMx in this study; details in Supplementary Text 5) and an Observation-Based Model (OBM; details in Supplementary Text 6). Results from all three methods consistently revealed a stagnation in the trend of O_3 sensitivity towards NO*x*-limited regimes in either 2019 or 2020. This trend-oriented approach circumvents the inconsistencies inherent in conventional sensitivity zoning methods, which arise from the use of static, absolute thresholds. Detailed information on sensitivity trends and their robust performance is provided in Supplementary Text 4.

Step 1c: Analysis of drivers of coupled response. "Coupling" refers to the synchronous analysis of de-weathered O_3 concentration trends and sensitivity trends, examining their dynamic response to various emission pathways across multi-decadal scales . Interpreting their coupled behavior (e.g., the synchronous plateauing observed from 2020-2023) under the context of historical emission pathways provides real-world evidence for path dependence and validates the framework's capability to timely capture dynamic signals. This framework implies that AVOCs reduction is more effective in the current phase, guiding the design of future quantitative prediction scenarios for Phase II (reinforcing AVOCs control beyond current regulatory strategies).”

Discussion, lines 446-453

“We posit that the reliance on such absolute thresholds for static 'sensitivity zoning' is a fundamental reason for the inconsistent and even contradictory conclusions regarding summer regional O_3 sensitivity in China. Our CDTR framework circumvents this methodological challenge by focusing on the more robust metric of relative evolutionary trends in sensitivity (robustness detailed in Supplementary Text 4). This approach dynamically resolves the evolving efficacy of different precursor control pathways by aligning them with the sensitivity evolution direction and pace, thereby providing more robust and adaptive policy insights. ”

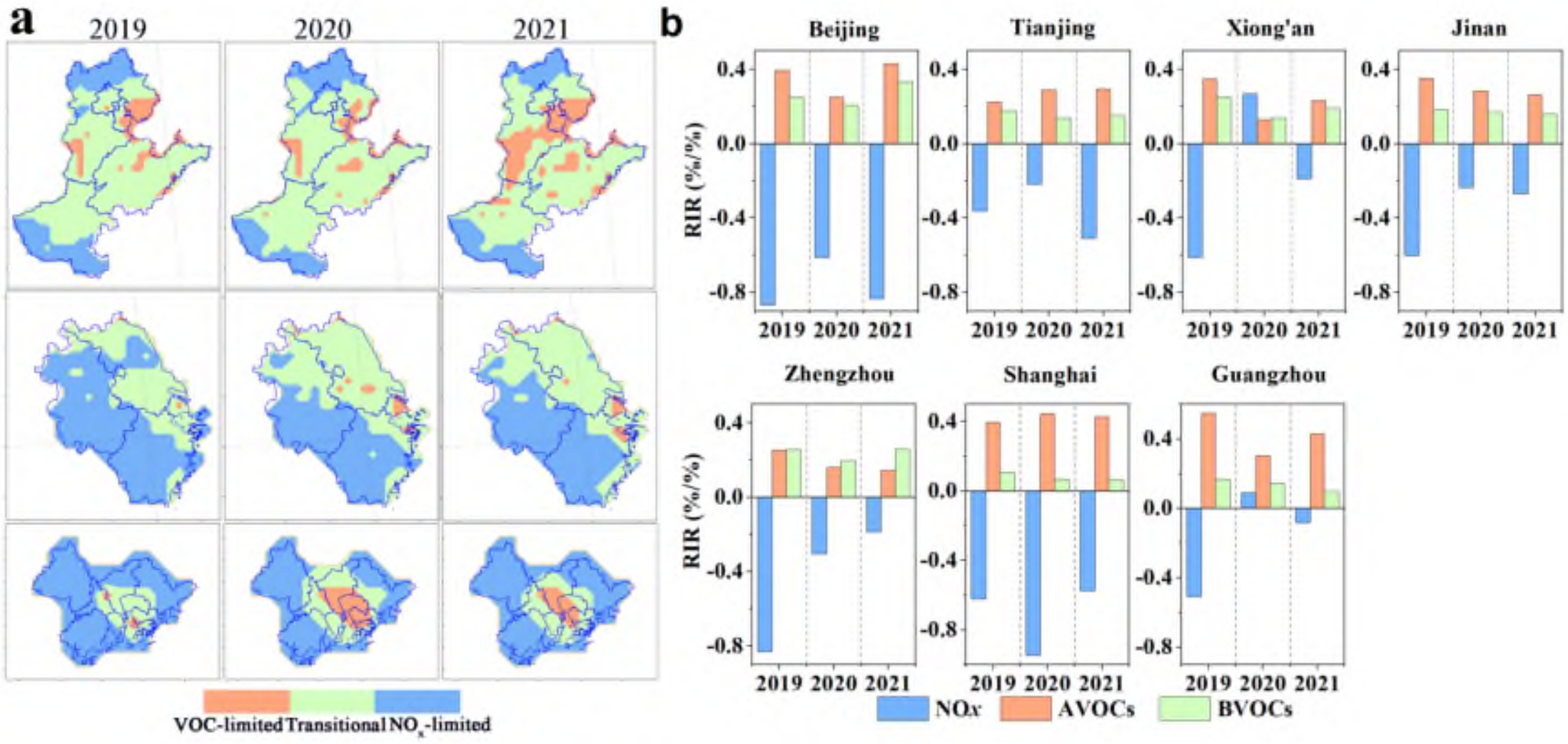

Fig. 2. Multi-source evidence confirms the robustness of the sensitivity trend stagnation . a, Maps illustrating O_3 formation regimes across the three megacity clusters (NCP, YRD and PRD from top to bottom) from 2019–2021 investigated by the WRF-CAMx model (the simulation resolution is 36km). **b,** relative increment reactivity (RIR) calculated by OBM model for O_3 precursors (specifically, NO_x, AVOC, and Biogenic VOC (BVOC)) of typical cities during the summers of 2019-2021.

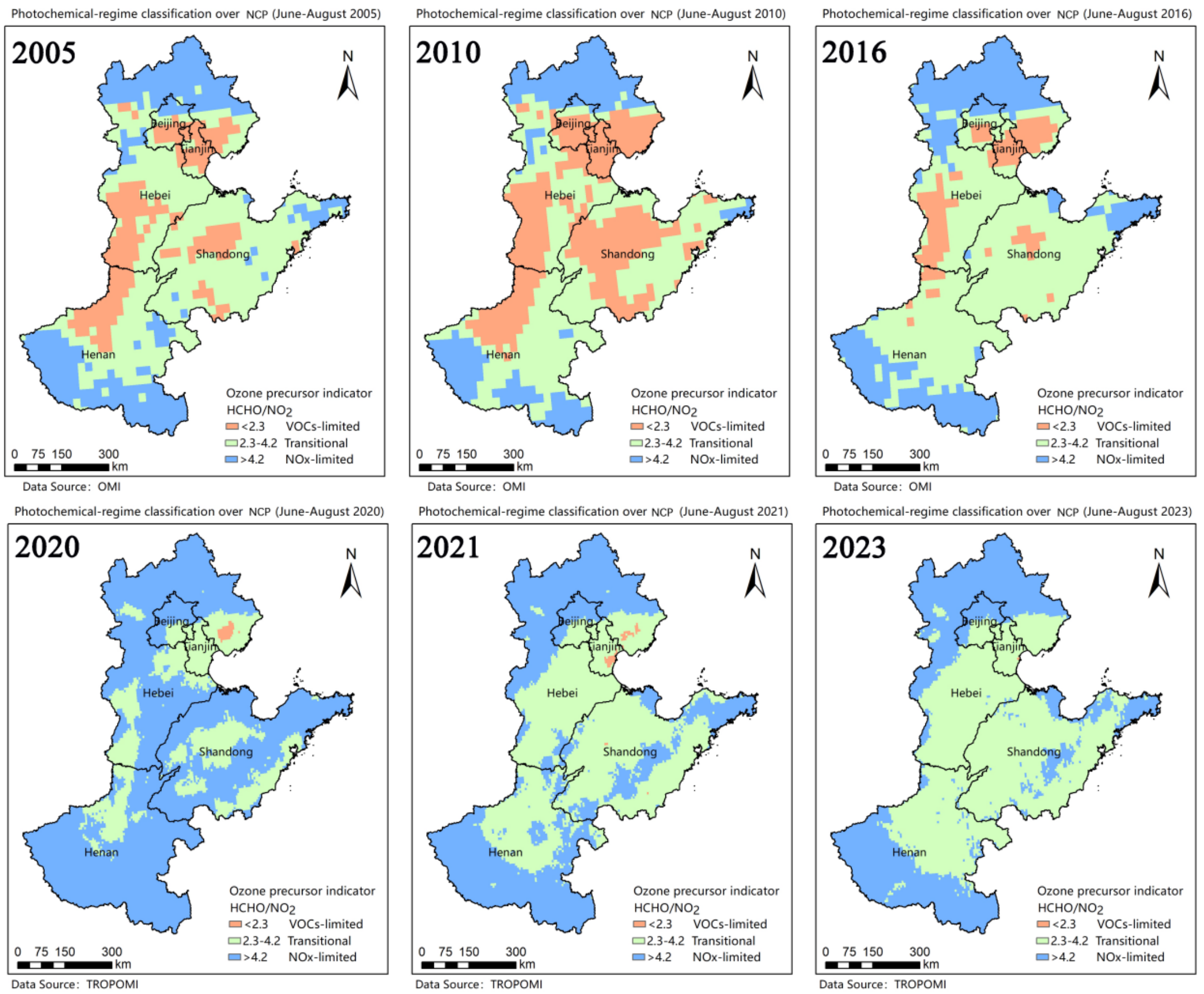

Supplementary Fig. 2. Summers O_3 formation sensitivity trend over the NCP illustrated by maps of the O_3 formation regime derived from satellite FNR ($HCHO/NO_2$) data of representative years from 2005–2023. A uniform FNR threshold [2.3-4.2] was adopted.

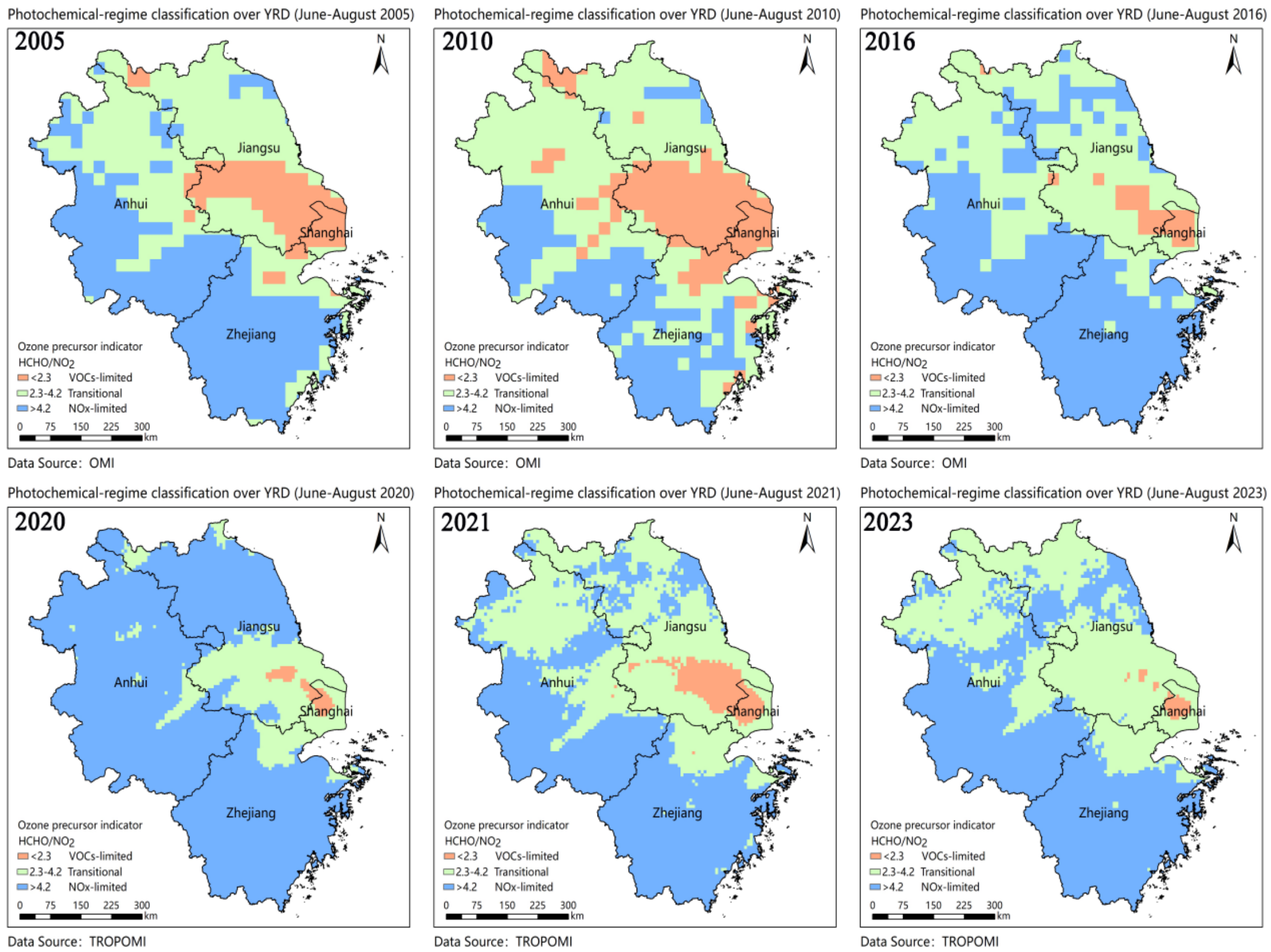

Supplementary Fig. 3. Summers O_3 formation sensitivity trend over the YRD illustrated by maps of the O_3 formation regime derived from satellite FNR ($HCHO/NO_2$) data of representative years from 2005–2023. A uniform FNR threshold [2.3-4.2] was adopted.

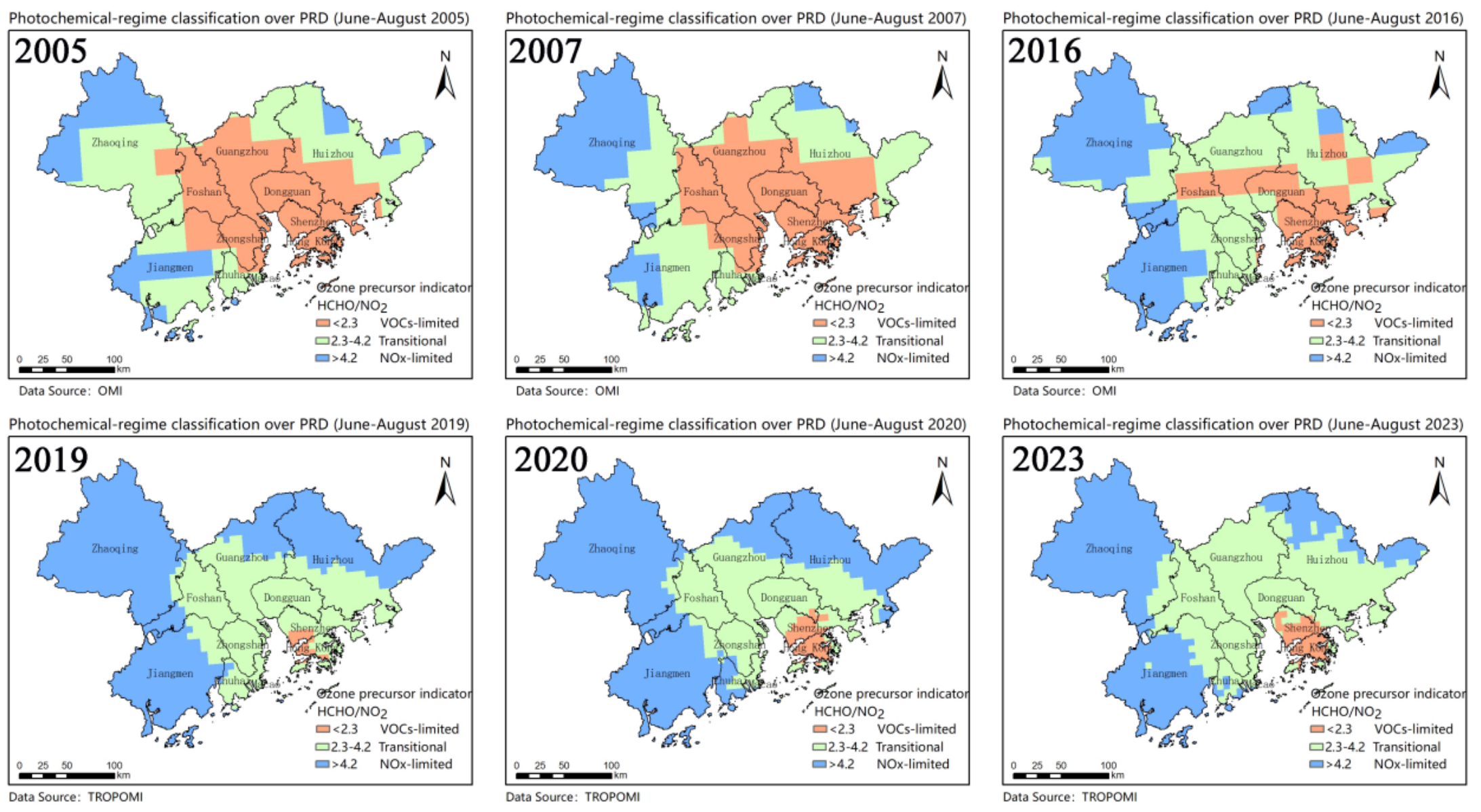

Supplementary Fig. 4. Summers O_3 formation sensitivity trend over the PRD illustrated by maps of the O_3 formation regime derived from satellite FNR ($HCHO/NO_2$) data of representative years from 2005–2023. A uniform FNR threshold [2.3-4.2] was adopted.

Supplementary Table 8. Comparison of the annual average area fractions of ozone formation regimes in the three megacity clusters during summers of 2019–2021 derived from WRF-CMAx and TROOMI. The thresholds of transition regime are [2.3,4.2]

CAMx	VOC-limited			Transitional			NOx-limited		
	NCP	YRD	PRD	NCP	YRD	PRD	NCP	YRD	PRD
2019	8.4	0.4	1.6	65.0	32.6	13.1	26.5	67.0	85.2
2020	7.5	2.9	9.8	68.4	45.9	23.0	24.1	51.3	67.2
2021	19.2	2.5	6.6	58.6	43.4	18.0	22.1	54.1	75.4

TROPOMI	VOC-limited			Transitional			NOx-limited		
	NCP	YRD	PRD	NCP	YRD	PRD	NCP	YRD	PRD
2019	1.2	3.5	4.0	28.9	21.2	25.4	69.8	75.3	70.6
2020	0.9	2.3	4.8	24.5	16.9	27.9	74.6	80.8	67.3
2021	1.5	6.0	6.0	42.1	35.9	29.0	56.4	58.1	65.0

Revised main Text, lines195-200:

“While differing in sensitivity regime classification (Supplementary Table 8, Supplementary Figs. 2-4 and Fig. 2a), which are the limitations of static sensitivity zoning methods, all three approaches (satellite FNR, WRF-CAMx, OBM) converged on the same sensitivity trend: the progression towards the NO*x*-limited regime stalled around 2020 in the three megacity clusters. This multi-method convergence strengthens the robustness of the observed sensitivity trend plateau phenomenon.”

The quantitative core of the CDTR framework lies in its Phase II (quantitative projection of optimal future pathways). This phase utilizes WRF-CAMx model simulations to project the long-term (2020-2060) coevolutionary trajectories of deweathered O_3 concentrations and sensitivity indicators (FNR) under different emission reduction scenarios (**Results**, lines 275-278 and Fig. 4a). Its quantitative assessment is primarily achieved through two approaches:

a. Scenario Comparative Analysis: By directly comparing the differences in de-weathered O_3 concentration and FNR evolution trajectories under various emission reduction scenarios, we are able to quantitatively identify optimal mitigation pathways. For instance, in Main Text, Fig. 4b, we demonstrate the significant additional O_3 reduction benefits derived from early intensified AVOCs reduction scenarios relative to NO*x* reduction scenarios within the initial 15-20 years. This is crucial for counteracting O_3 concentration increases due to intensifying heatwave events driven by global warming, thereby ensuring a sustained decline in O_3 concentrations to achieve clean air policy objectives.

lines 275-278

“To quantify the optimal O_3 mitigation strategy under synergistic climate action and clean air policies, we applied our CDTR framework, implemented via WRF-CAMx simulations, to project the long-term (2020–2060) co-evolution of O_3 concentration and sensitivity (Fig. 4a).”

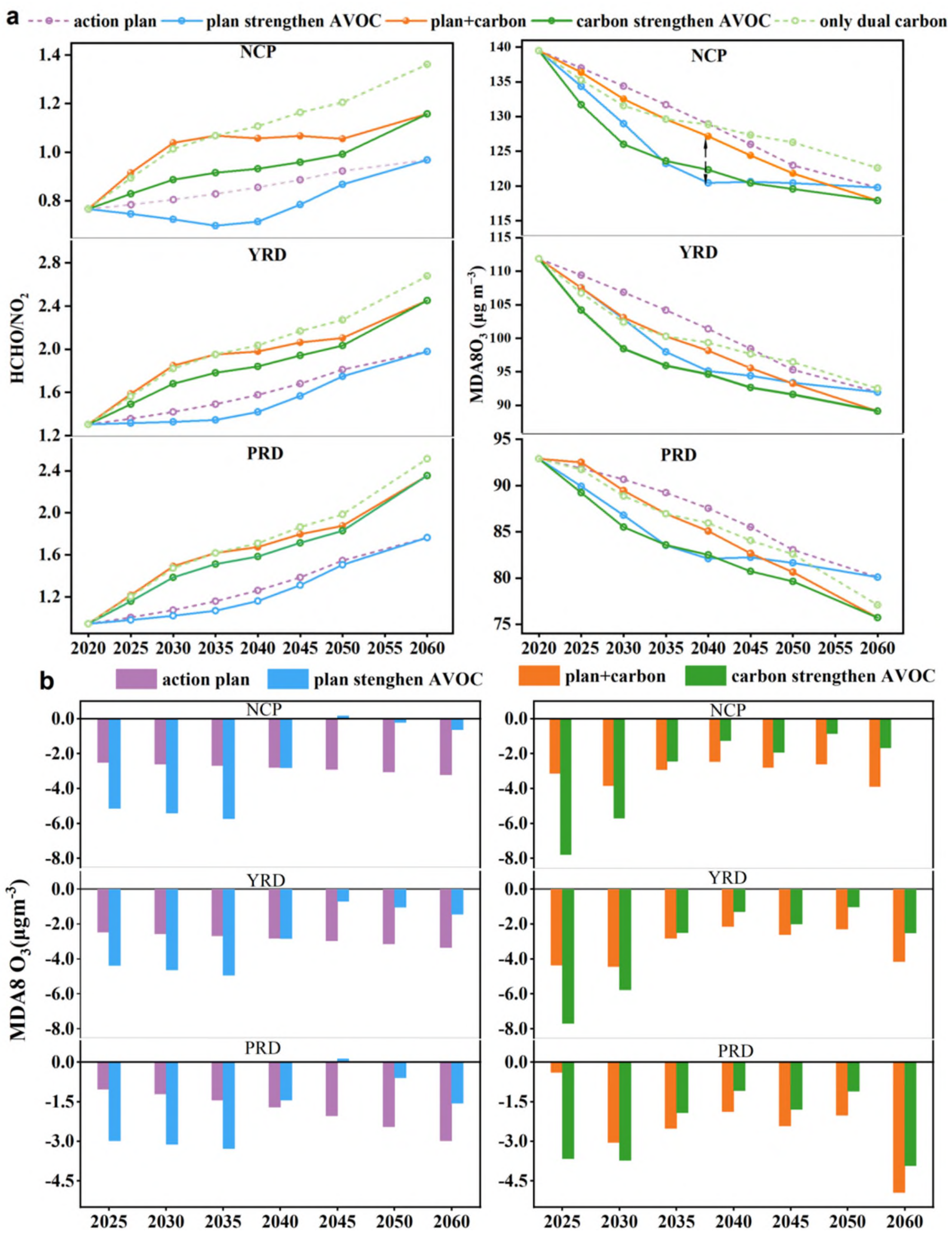

Fig. 4. Coupled dynamic trend response of the model-derived MDA8 O_3 concentration and sensitivity from 2020–2060 quantitatively reveals path dependency and confirms the optimality of early AVOC reductions. a, Projected changes in the interannual summer variations in model-derived FNR values and the corresponding MDA8 O_3 concentrations across the three megacity clusters from 2020–2060. The black arrow conceptually illustrates the diminishing advantage of reducing equivalent proportions of AVOCs over NO*x* on the MDA8 O_3 decrease, shown here for NCP in 2040 as an example quantified in Table 2. **b,** Projected MDA8

O_3 concentration reductions relative to the previous five years under the paired scenarios, highlighting the initial phase differences. The meteorological field and BVOC emission inputs are fixed at the 2020 level.

b. Quantification of the "AVOCs Reduction Bonus Window" Duration and the crossing of the O_3 isopleth 'ridge line' : We have added a dedicated sub-section in the "**Methods**" part: "Quantification method for the duration of the AVOCs reduction bonus window" (Methods, lines 742-780). This section elaborates in detail on how to quantitatively calculate and define the duration and closure points of the "AVOCs reduction bonus window" through a series of subtraction operations (comparing the MDA8 O_3 reduction benefits between paired scenarios). The specific calculation involves three steps: the O_3 reduction benefit from individual NO*x* reductions (Supplementary Fig. 7c), the O_3 reduction benefit from individual AVOCs reductions (Supplementary Fig. 7b), and the "bonus" from AVOCs reductions relative to equal proportion of NO*x* reductions (Main Text, Fig. 5). The results presented in Supplementary Fig. 7c indicate that, by 2025, the NCP and PRD have crossed the O_3 isopleth 'ridgeline', entering a transitional regime where reductions in either AVOCs or NO*x* leads to O_3 concentration decreases. The YRD region is projected to reach this tipping point slightly later. The results presented in Supplementary Fig. 5 indicate that, the 'bonus window' for AVOC control is projected to close asynchronously (earliest for the PRD around 2045, followed by the YRD around 2050. the NCP region maintains a persistent, though diminishing, AVOC control bonus beyond 2050).

Methods, lines 742-780

"**Quantification method for the duration of the AVOCs reduction bonus window**

To quantitatively assess the duration 'bonus window' of a prioritized AVOCs control strategy, we calculated the difference in O_3 reduction efficacy resulting from equivalent proportional reductions of AVOCs versus NO*x* (Fig. 5). This calculation is based on a comparative analysis of simulation results from three scenarios, whose O_3 photochemical system evolutionary trajectories are shown in Supplementary Fig. 7a. Specifically, the 'plan+carbon' scenario shares identical AVOC reduction trajectory with the 'action plan' scenario but implements greater NO*x* reductions. Therefore, subtracting the 'plan+carbon' scenario from that of the 'action plan' scenario isolates the MDA8 O_3 reduction attributable to the additional NO*x* abatement in that year .

Conversely, the ‘carbon strengthen AVOCs’ scenario maintained the same NO*x* reduction targets as the ‘plan+carbon’ scenario but implements greater AVOC reductions. Thus, subtracting the ‘carbon strengthen AVOCs’ scenario from that of the ‘plan+carbon’ scenario isolates the MDA8 O_3 reduction attributable to the additional AVOC abatement. The 'AVOCs reduction bonus window' closes when the MDA8 O_3 mitigation benefit from equiproportional NO*x* reduction exceeds that of AVOCs reduction. We quantify this by subtracting the MDA8 O_3 benefit caused by more AVOCs reduction (Extended Data Fig. 2b) from those under the parallel more NO*x* reduction (Extended Data Fig. 2c). The point at which this difference (Fig. 5) becomes negative marks the transition to a NO*x*-limited regime, where NO*x* control becomes the more effective strategy.

This derivation, explicitly illustrated in Supplementary Fig. 7 and Fig. 5, involves the following three steps:

1. Isolating the O_3 reduction benefit from NO*x* reduction: We subtracted the ‘plan+carbon’ scenario from those of the ‘action plan’ scenario. This isolates the net MDA8 O_3 reduction solely attributable to the additional NO*x* abatement (Supplementary Fig. 7b). The percentages shown in the figure represent the proportion of additional NO*x* reduction for that year.

2. Isolating the O_3 reduction benefit from AVOCs reduction: We subtracted the ‘carbon strengthen AVOCs’ scenario from those of the ‘plan+carbon’ scenario. This difference represents the net MDA8 O_3 reduction solely attributable to the enhanced AVOC control measures (Supplementary Fig. 7c). The percentages shown in the figure represent the proportion of additional AVOC reduction for that year.

3. Calculating the difference in benefits: Finally, we subtracted the O_3 reduction benefit derived from AVOC control (result of Step 2) from the benefit derived from NO*x* control (result of Step 1). The resulting value, ΔMDA8 O_3(AVOCs) − ΔMDA8 O_3(NO*x*), represents the net advantage of prioritizing an equivalent reduction in AVOCs over NO*x*—the ‘bonus’. This final time series constitutes the data presented in Fig. 5 of the main text.

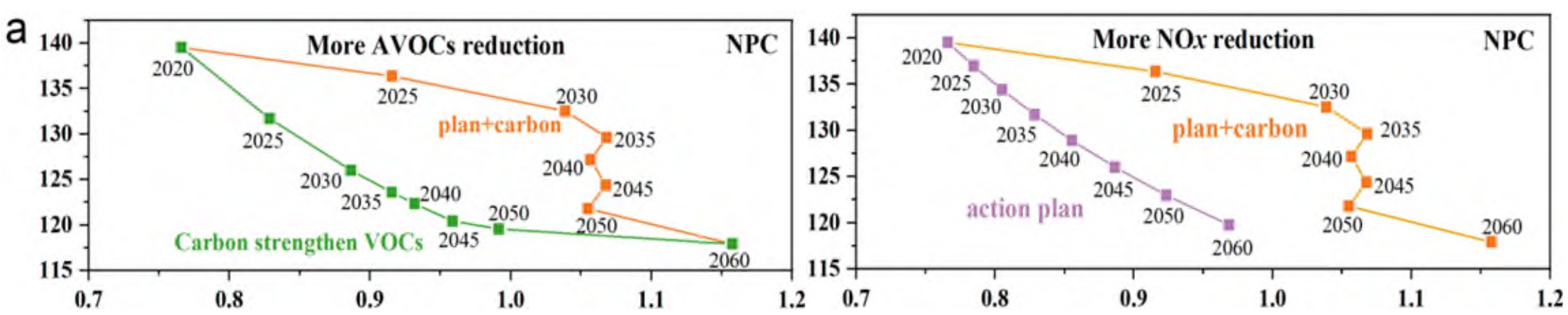

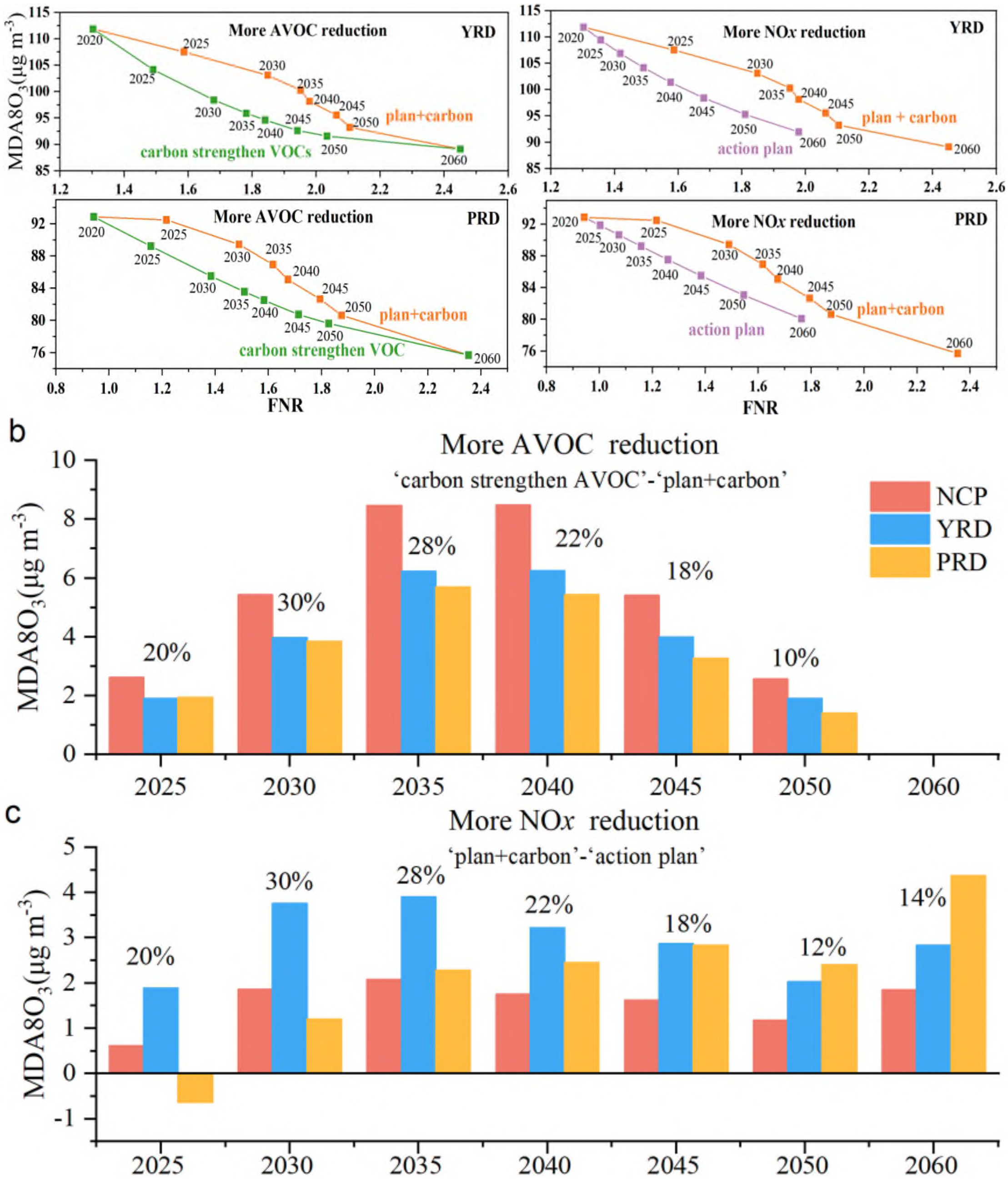

Supplementary Fig. 7. Derivation of isolated O$_3$ reduction benefits from AVOC versus equivalent proportional NO*x* reduction. a, the evolution trajectory of O$_3$ photochemical system (O$_3$-FNR) of paired-scenario designed to decouple the effects of AVOC versus NO*x* reductions in China's three megacity clusters from 2020 to 2060. The 'More AVOCs reduction' comparison isolates the impact of AVOCs by comparing the 'carbon strengthen AVOCs' and 'plan+carbon' scenarios, which share an identical NO*x* trajectory. The 'More NO*x* reduction' comparison isolates the impact of NO*x* by comparing the 'plan+carbon' and 'action plan' scenarios, which share an identical AVOC trajectory. **b, Isolated MDA8O$_3$**

reduction from dedicated AVOC controls, calculated as the MDA8O_3 difference between the 'carbon strengthen AVOCs' and 'plan+carbon' scenarios. Percentages indicate the additional AVOC reduction for that year. **c, Isolated MDA8 O_3 reduction from dedicated NO*x* controls**, calculated as the MDA8 O_3 difference between the 'plan+carbon' and 'action plan' scenarios. Percentages indicate the additional NO*x* reduction for that year.

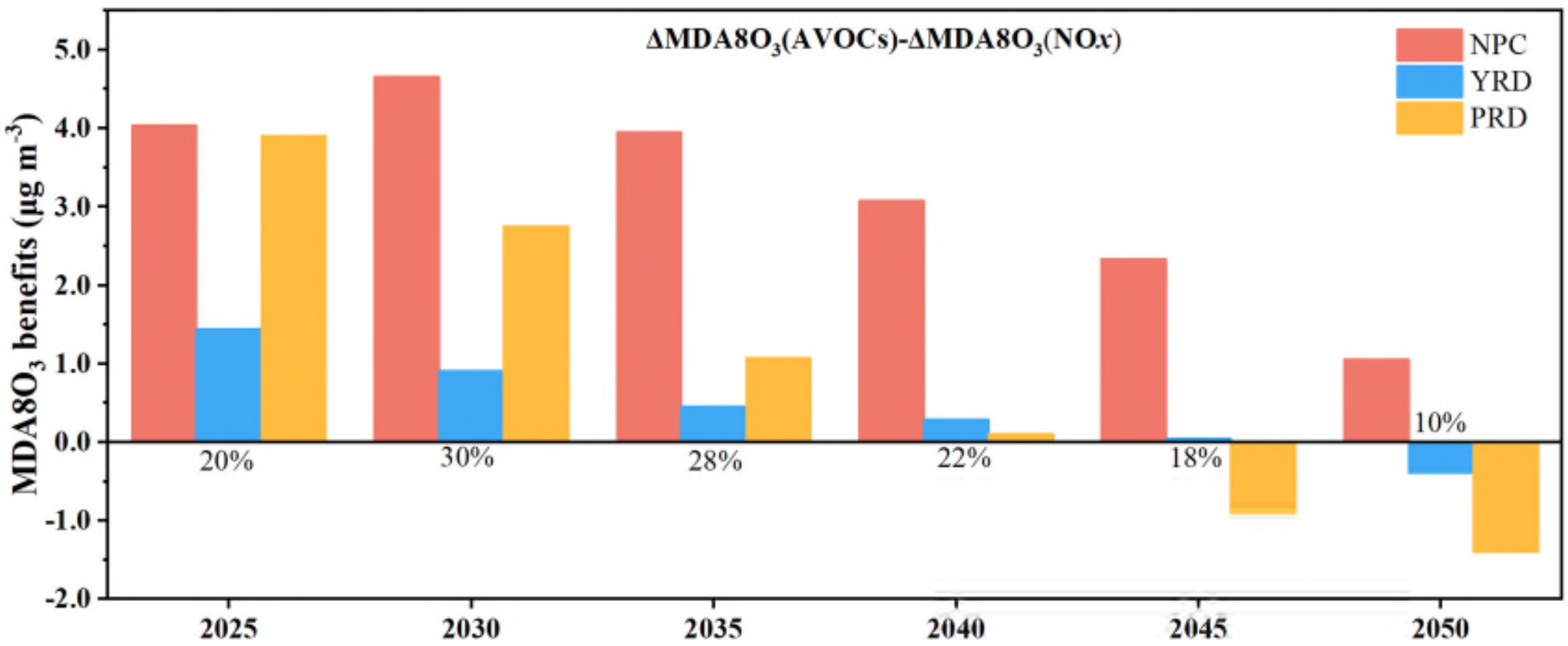

Fig. 5 | Dynamics of the AVOC reduction bonus window in China's three megacity clusters. The bonus window for prioritizing AVOCs is considered closed when its net MDA8 O_3 mitigation benefit—defined as the difference in O_3 reduction between reduction AVOCs versus an equivalent proportion of NO*x*—becomes negative. Percentages denote the precursor reduction fraction for the corresponding year."

We have explicitly highlighted the innovation and capabilities of the CDTR framework in the **Abstract** (lines 50-52), **Introduction** (lines 92-101), **Results** (lines 140-143), and **Discussion** (lines 424-439) sections of the Main Text, and have more clearly referenced the detailed explanations in the "**Methods**" section.

We hope that these detailed clarifications and revisions have fully addressed the reviewer's questions and provided the necessary details for a clear understanding and reproducible application of the CDTR analytical framework. We thank the reviewer for prompting us to improve this crucial aspect of our manuscript.

Some additional comments:

2. It is difficult to follow all the different scenarios i.e. "action plan" vs. "plan

strengthen VOC" vs. "plan + carbon". It would be helpful to include a table in the manuscript which contains each scenario, and a brief description of how this scenario is unique / what it means from an emissions perspective.

Reply: We sincerely thank the reviewer's suggestion. We agree that the narrative descriptions of individual scenarios in the original manuscript may have been difficult to track, and a more structured presentation will significantly enhance the clarity of the article.

To address this, we have implemented two main revisions to the manuscript:

(1) We have introduced Table 1 in the main text. This table concisely and quantitatively summarizes all five emission reduction scenarios, clearly presenting the NO*x* and AVOC reduction percentages for each key year from 2020 to 2060. This enables readers to readily compare the distinct emission pathways of each scenario.

(2) For detailed descriptions of each scenario, we have included a dedicated subsection in the Methods section, titled "Setting scenarios" (lines 642-700 in the revised manuscript). This subsection systematically explains the design rationale, policy underpinnings (e.g., existing clean air policies, DPEC model projections towards carbon neutrality), and unique characteristics of each scenario. Specifically, it clarifies how the 'action plan' scenario represents current policies, the 'only dual carbon' scenario reflects climate action-driven pathways, and the 'plan + carbon' scenarios are designed to investigate the specific efficacy of AVOC and NO*x* control, etc. In addition, a brief description of individual scenarios in the revised manuscript was provided in Lines 278-293.

We are confident that the combined addition of Table 1 and the detailed "Setting scenarios" section provides the essential clarity and comprehensive reference needed to understand the design and implications of each scenario, thereby fully resolving the concern raised by the reviewer.

Specific revisions are provided below:

Lines 278-293:

"Five precursor reduction scenarios (Table 1) were designed to represent different reduction pathways (detailed in Methods). In brief, two benchmark scenarios, the 'action plan', reflect current clean air policies[22], and the 'only dual carbon', refer to an ambitious NO*x*-targeted pathway designed to meet China's climate neutrality goals

within the "Dynamic Projection Model for Emissions (DPEC)"[28,29]. In light of historical observations findings that underscore the significance of AVOC reduction for O_3 mitigation at current stage, and considering the 'only dual carbon' scenarios already adopted the most ambitious NO*x* reduction measures, we designed two additional scenarios: the 'plan strengthen AVOCs' and the 'carbon strengthen AVOCs'. These scenarios maintain the respective NO*x* reduction trajectories of the benchmark scenarios but incorporate intensified reductions in AVOCs. Finally, a 'hybrid' scenario ('plan + carbon') was designed to directly compare the efficacy of AVOC versus NO*x* controls on MDA8 O_3 reduction. This scenario combines the NO*x* emission reduction trajectory from the 'only dual carbon' (climate-driven) scenario with the AVOC reduction trajectory from the 'action plan' scenario.

Table 1. The reduction proportions of NO*x* : AVOCs from 2020 to 2060 under 5 different scenarios. The base emission amount is fixed at 2020.

Year	action plan	plan strengthen AVOCs	plan+carbon	carbon strengthen AVOCs	only dual carbon
2025	10%:10%	10%:20%	30%:10%	30%:30%	30%:15%
2030	20%:20%	20%:40%	50%:20%	50%:50%	50%:25%
2035	30%:30%	30%:60%	58%:30%	58%:58%	58%:30%
2040	40%:40%	40%:70%	62%:40%	62%:62%	62%:31%
2045	50%:50%	50%:70%	68%:50%	68%:68%	68%:34%
2050	60%:60%	60%:70%	72%:60%	72%:70%	72%:36%
2060	70%:70%	70%:70%	84%:70%	84%:70%	84%:42%

Setting scenarios(lines 643-700 in the revised manuscript)

"Five emission reduction scenarios (Table 1) were developed to align with China's ongoing clean air policy[22] and its climate action commitments (carbon neutrality target)[27]. These scenarios are based on two distinct approaches. Two scenarios, based on current air quality policies ('action plan' and 'plan strengthen AVOCs'), adopt a conventional static reduction proportion relative to a base year (2020). In contrast, our three climate-oriented scenarios ('only dual carbon', 'carbon strengthen AVOCs' and 'plan + carbon') employ dynamic precursor reduction proportions relative to 2020 levels from the DPEC in China[27,28]. These dynamic

reduction proportions reflect anticipated future emission changes driven by socioeconomic development, climate actions, and advancements in pollution control technology. Specifically, because NO*x* emissions are more closely linked to energy transitions towards renewables under carbon neutrality actions than to AVOCs[10], we determined NO*x* emission reduction trajectories by referencing the *Ambitious-Pollution-Neutral-Goals* (APNG) scenario within the DPEC model[27,28]. The DPEC-APNG scenario projects that China will achieve carbon neutrality by 2060, with a CO_2 emission trajectory between representative concentration pathways 1.9 and 2.6. This approach ensures that our emission reduction scenarios better represent potential real-world dynamics. The following describes the design of these five emission reduction scenarios.

We designed two scenarios to represent pathways based on current air quality policies. The 'action plan' scenario simulates the implementation of China's current clean air policy[22]. It assumes a linear reduction of 10% every five years for both NO*x* and AVOCs relative to 2020 levels, extending through 2060. This establishes a baseline policy pathway with a constant 1:1 reduction ratio between the two precursors. The 'plan strengthen VOCs' scenario investigates the impact of prioritising AVOC abatement. It retains the same NO*x* reduction trajectory as the 'action plan' but doubles the AVOC reduction rate, thereby enforcing a 1:2 NO*x*-to-AVOC reduction ratio. This intensified AVOC control is applied until the cumulative reduction reaches 70% (projected for 2040). This scenario explores the impact of prioritising AVOC control within the scope of the clean air policy.

We further defined two scenarios that represent pathways based on climate actions towards carbon neutrality. The 'only dual carbon' scenario represents a future where emission reductions are driven by decarbonisation. The NO*x* emission reduction trajectory is derived from the DPEC-APNG scenario within the DPEC model, which adopted the most ambitious NO*x* pollution control measures. Under this scenario, the ratio of NO*x* to AVOC emissions was set to 2:1, reflecting partially shared emission sources. This ratio was set for two reasons: (1) Shi et al.[37] projected that the projected emission reductions of NO*x* and AVOCs would be 90% and 61% in 2060 compared with 2020, respectively; and (2) US EPA historical data show NO*x* and AVOC emission reductions of 71% and 48%, respectively, between 1990 and 2020 in the U.S. (https://gispub.epa.gov/air/trendsreport/2023/, accessed on 29 April 2024). The 'carbon strengthen AVOCs' scenario builds upon the NO*x* reductions of

the ‘only dual carbon’ scenario but includes aggressive AVOC control, resulting in a 1:1 NO*x* to AVOC reduction ratio. This scenario represents a synergistic climate and clean air policy.

Finally, a hybrid scenario (the ‘plan + carbon’) was designed for comparison. This scenario combines the NO*x* emission reduction trajectory from the ‘only dual carbon’ scenario (climate-driven) with the AVOC reduction trajectory from the ‘action plan’ scenario. Compared with the ‘plan strengthen AVOCs’ scenario, this scenario allows a comparison of the efficacy of AVOC and NO*x* reductions on O_3 reduction. This occurred because the total reduction amounts of AVOCs and NO*x* in these two scenarios from 2030–2050 are similar, despite the different relative emphasis on NO*x* versus AVOCs. Crucially, the design of this hybrid emission reduction scenario enables clear visualisation of the “AVOC bonus window” for O_3 mitigation.

The grounding of these scenarios in policy-relevant pathways synergistic with clean air and climate actions, combined with the CDTR’s ability to evaluate the resulting long-term dynamic responses, provides the foundation for its adaptability and relevance. ”

3. The focus of the VOC reductions is anthropogenic, as emissions of these are easier for policy makers to control. However, it is important that the authors comment on the impact biogenic VOCs may have in the future on their results. How sensitive are the author’s results to potential future changes in biogenic VOCs?

Reply: We sincerely thank the reviewer for this insightful comment. We agree on the importance of considering the future impact of BVOCs on O_3 formation and the mitigation strategies. We have indeed addressed this crucial aspect in the Discussion section (lines 464-477 in the revised manuscript).

As explicitly stated in the Discussion section: "To isolate the independent effects of anthropogenic emission abatement, our future projections fixed meteorological fields and BVOC emissions at 2020 baseline levels. However, future climate change, characterized by projected rising temperatures and more frequent heatwaves, is expected to increase BVOC emissions[35]. This would drive the photochemical environment towards more VOC-rich and thus more NO*x*-limited state. This means future increases in BVOCs would likely accelerate the transition to a NO*x*-limited regime and thus result in a shorter period for AVOC reduction bonus window.

However, this prospect does not undermine our core conclusion; rather, it magnifies the strategic urgency of capitalizing on this window of opportunity for AVOCs abatement before it closes. More complex climate-O_3 interactions, such as temperature effects on agricultural NO*x* and AVOCs emissions[35], and anthropogenic NO*x* and AVOCs emission pattern change caused by heatwave-driven shifts in energy structure[36], remain critical areas for future investigation. "

This discussion confirms that our results are indeed sensitive to future changes in BVOCs. We acknowledge that increased BVOCs due to climate change would likely accelerate the transition to a NO*x*-limited regime and, consequently, shorten the "AVOC reduction bonus window." Crucially, this potential acceleration of the O_3 system towards a NO*x*-limited state reinforces our core conclusion about the strategic urgency of leveraging the early AVOC reduction bonus window before it closes. We hope this clarification, already present in our revised manuscript, fully addresses the reviewer's important point.

4. Line 316: "The meteorological field and BVOC emission inputs are fixed at the 2020 level". Is this realistic? What is the sensitivity of your study to changes in this?

Reply: We sincerely thank the reviewer for raising this important point regarding the realism and sensitivity of our model projections. We agree that a discussion on the potential impacts of future changes in BVOC emissions and meteorology is essential for contextualizing our long-term projections. In the revised manuscript, we have added a dedicated paragraph in the **Discussion** section (lines 464-477 in the revised manuscript, detailed in the last reply) to thoroughly address this issue.

Our response is structured as follows: First, we explicitly state our initial assumption of fixing BVOC emissions and meteorology at 2020 levels, clarifying that this was a deliberate choice to isolate the effects of anthropogenic abatement, which is a common practice in such projection studies. Second, we discuss the likely consequence of future climate change—namely, that rising temperatures will increase BVOC emissions. Third, we connect this back to our core findings by explaining that such an increase would accelerate the transition of the photochemical system towards a NO*x*-limited regime, thereby shortening the 'AVOC reduction bonus window'.

Crucially, we argue that this prospect does not undermine our central conclusion. Instead, it magnifies the strategic urgency of capitalizing on this time-limited

opportunity for AVOC abatement before it closes. We hope this clarification not only resolves the reviewer's concern but also strengthens the paper's central message.

5. Line 361: Completely out of the blue, the authors state that further considerations into O_3 seasonality suggest that a year-round VOC strengthening is a win-win strategy for China, but I don't really know what this means. There is no additional reference to seasonality in the results, and "VOC strengthening" sounds like VOCs should be increases, which is contradictory to the overall message. The manuscript is very "conclusions" heavy, with not enough attention given to presenting and discussing the data itself.

Reply: We are deeply grateful for the constructive comments from the reviewer, which have significantly helped us to enhance the clarity, logical structure, and depth of our manuscript. We fully concur with all three points raised by the reviewer: (1) the abrupt introduction of the seasonality argument, (2) the ambiguous phrasing of "VOC strengthening," and (3) the original manuscript being "too conclusion-heavy," necessitating a more comprehensive discussion.

We have thoroughly revised the manuscript to address these critical issues.

(1) Regarding the abrupt introduction of the "seasonality" argument and the ambiguity of the "VOC strengthening" terminology:

We agree that the original phrasing was abrupt, and the term "VOC strengthening" could be misinterpreted. We sincerely apologize for this lack of clarity in expression.

- Clarification of "VOC strengthening": In the revised manuscript, we have explicitly clarified that "AVOC reduction strengthening", which refers to "intensifying control measures for AVOCs," not increasing their emissions.
- Establishing a logical connection for "seasonality": To address the "out-of-the-blue" seasonality argument, we have revised the paragraph by adding an introductory sentence ("Finally, placing our findings in a broader spatiotemporal context reveals further strategic implications.") at the beginning to clearly establish a logical bridge. It is a well-established scientific fact that O_3 sensitivity in mid-latitude regions like China exhibits strong seasonality, typically being VOC-limited in winter (due to lower biogenic emissions and temperatures) and trending towards NO_x-limited in summer. Since our work quantitatively demonstrates a long-term

AVOCs prioritization strategy in summer extending for approximately 30 years (to ~2050), and O_3/$PM_{2.5}$ synergistic control in winter and spring inherently requires AVOCs reductions, a year-round, strengthened AVOCs control strategy logically emerges as a beneficial "win-win" approach. The revised text is as follows (lines 534-543):

"Finally, placing our findings in a broader spatiotemporal context reveals further strategic implications. Our finding of a long-term summer AVOC control priority extending to approximately 2050, coupled with the recognized seasonality in O_3 sensitivity (e.g., VOC-limited in winter and increasingly NO*x*-limited in summer due to elevated biogenic emissions at higher temperatures, etc.[43]), collectively suggests that implementing year-round AVOC strengthening constitutes a win-win strategy for China, fostering both sustained air quality improvement and climate change mitigation. This year-round AVOC intensification offers cobenefits to address increasingly frequent high O_3 episodes in late spring and persistent $PM_{2.5}$ exceedances in winter[6,7,44]. "

(2) Regarding the structural issue of the manuscript being "too conclusion-heavy":

We are deeply thankful for this crucial feedback, which prompted us to undertake significant structural adjustments and expansions of the manuscript to provide a more balanced and in-depth discussion.

In response, we have added a comprehensive, multi-paragraph **Discussion** section (revised manuscript, lines 399-552). This new section goes far beyond merely stating conclusions; it now elaborates extensively on the methodology, and their profound implications, including:

- Integrating our findings into a dynamic policy blueprint (Fig. 6).
- Discussing in greater detail the methodological innovations of our CDTR framework, contrasting it with the limitations of traditional static zoning methods, and exploring potential discrepancies in adopting FNR absolute values (lines 424-463).
- A dedicated paragraph addressing the key limitations and fundamental assumptions in our modeling work, such as fixed meteorological fields, BVOCs emissions, and background CH_4 concentrations. We now explicitly discuss how these factors might influence the 'AVOCs reduction bonus window' and highlight them as critical directions for future research (lines 464-485).

- An entirely new paragraph exploring the implications of evolving AVOCs speciation and reactivity-based reduction control, demonstrating the adaptability of the CDTR framework (lines 486-533).
- Expanded discussion placing our findings in a broader spatiotemporal context, including year-round strategies and spatial heterogeneity (lines 534-552).

We hope that these extensive revisions directly address the reviewer's concern. The manuscript is no longer "conclusion-heavy," but instead offers a deep, contemplative, and critically thought-out discussion befitting a high-impact research paper. We believe that these substantial revisions significantly enhance the quality of the manuscript and once again thank the reviewer for the invaluable guidance in this process.

Response to Reviewer #4

Zhang et al. investigate the long-term effectiveness of ozone mitigation strategies in China up to 2060. Focusing on how emission pathways shape the relative benefits of VOC versus NOx controls, the study challenges the conventional NOx-first control paradigm and shows that early VOC reductions are particularly effective for lowering regional ozone. It also critiques the commonly used "sensitivity zoning," which relies on steady-state and short-timescale assumptions, and proposes an alternative method, the "coupled dynamic trend response." Despite addressing a relevant topic, this manuscript requires major revisions before it can be considered for publication.

Reply: We thank the Reviewer for the thorough review and insightful summary of our work.

1. First, several previous studies have extensively investigated O_3 mitigation strategies. It would therefore be valuable for the authors to clearly highlight the unique findings or novel contributions of this work.

Reply: We are grateful to the reviewer for this insightful and constructive suggestion. We fully agree that, within the context of the extensive literature on O_3 mitigation strategies, clearly and explicitly articulating the unique contributions of our study is crucial. This valuable advice has prompted a comprehensive refinement of our manuscript's scientific narrative to better accentuate its novelty.

While previous studies have indeed offered valuable insights into O_3 sensitivity and control strategies, our work makes novel contributions on the following interconnected fronts:

(1) Conceptual innovation: We move beyond the traditional, static partitioning of 'VOC-limited' versus 'NO*x*-limited' regimes by introducing the CDTR, an observation-driven projection framework, and quantifying the concept of 'path dependency'. The duration of the time-limited 'AVOCs bonus window', coupled with the inherent adaptability of our CDTR framework, provides a quantitative basis for the strategic timing of control policies—a critical dimension that, to our knowledge, has not been previously reported. Detailed in revised manuscript lines 298-304 and lines 342-352.

(2) Policy Relevance: Our study is the first to leverage insights from two decades of field observations to guide air quality modeling of optimal O_3 control strategies under climate-air quality policy scenarios. Through a unique, policy-relevant control scenario design (Result, lines 275-293; also reflected in Method, lines 642-700), we provide a quantitative, long-term strategic blueprint for achieving O_3 attainment in synergy with both short-term air quality targets and long-term climate goals.

lines 275-293:

"To quantify the optimal O_3 mitigation strategy under synergistic climate action and clean air policies, we applied our CDTR framework, implemented via WRF-CAMx simulations, to project the long-term (2020–2060) co-evolution of MDA8 O_3 concentration and sensitivity (Fig. 4a). Five precursor reduction scenarios (Table 1) were designed to represent different reduction pathways (detailed in Methods). In brief, two benchmark scenarios, the 'action plan', reflect current clean air policies[22], and the 'only dual carbon', refer to an ambitious NO*x*-targeted pathway designed to meet China's climate neutrality goals within the "Dynamic Projection Model for Emissions (DPEC)"[28,29]. In light of historical observations findings that underscore the significance of AVOC reduction for O_3 mitigation at current stage, and considering the 'only dual carbon' scenarios already adopted the most ambitious NO*x*

reduction measures, we designed two additional scenarios: the 'plan strengthen AVOCs' and the 'carbon strengthen AVOCs'. These scenarios maintain the respective NO_x reduction trajectories of the benchmark scenarios but incorporate intensified reductions in AVOCs. Finally, a 'hybrid' scenario ('plan + carbon') was designed to directly compare the efficacy of AVOC versus NO_x controls on MDA8 O_3 reduction. This scenario combines the NO_x emission reduction trajectory from the 'only dual carbon' (climate-driven) scenario with the AVOC reduction trajectory from the 'action plan' scenario."

To explicitly highlight these contributions, we have undertaken substantial revisions to the manuscript:

- We have significantly revised the final two paragraphs of the **Introduction** (now at lines 92–121 in the revised manuscript) to clearly articulate the novelty of our study at the outset.

"Here, we address this challenge through the lens of path dependency: early choices in precursor emission controls decisively shape the multi-decadal trajectory of O_3 photochemistry (deweathered concentration and sensitivity), thereby dictating the synergistic achievement of both air quality and climate goals. To capture this critical dynamic, we develop and apply a Coupled Dynamic Trend Response (CDTR) analytical framework (Methods). This approach moves beyond ambiguous sensitivity 'zones' by focusing on the robust, coupled trends of deweathered O_3 concentrations and key sensitivity indicators (e.g., satellite-derived formaldehyde-to NO_2 ratios, FNR) over multidecade timescales. It thus provides a tool to dynamically evaluate how the O_3 photochemical system responds to different emission control pathways.

In this study, we first leverage the policy-driven emission changes in China over a two-decade period (2005–2024) as a unique large-scale 'natural experiment' to probe the evolution of summer regional O_3 photochemistry. This field observation reveals a previously undocumented synchronous plateau in both regional O_3 deweathered concentrations and their sensitivity trends from 2020–2023, providing empirical validation for our CDTR framework's ability to capture dynamic signals inaccessible to traditional sensitivity zoning methods. Analysis of underlying drivers of the O_3 photochemistry trajectories reveals real-world evidence of path dependency, indicating that prioritizing reductions in AVOCs is now the more effective strategy for mitigating summer O_3 in China's three megacity clusters (NCP, YRD, and PRD;

Supplementary Fig. 1). Building on this observation-derived directive, we apply the CDTR framework to quantitatively project optimal future O_3 mitigation strategies in the context of China's climate actions and clean air policies[22]. These projections quantitatively identify a critical, time-limited 'AVOC reduction bonus window,' establishing that an early and strengthened AVOC control strategy is imperative. These findings challenge the conventional strategy of rapidly transitioning O_3 chemistry to a NO*x*-limited regime. This study reframes the understanding of long-term O_3 control strategies in a climate-changing world by highlighting the role of path dependency, and establish a new, globally applicable analytical framework for designing adaptive air quality-climate co-governance."

- In the revised **Results** and **Discussion** sections (e.g., lines 140–142, 300–306, 345–355, 486–533), we present several novel findings that are distinct from previous research:

(1) Real-world Evidence and Visual Presentation of Path Dependency: Our field observations reveal an unprecedented synchronous plateau in both regional de-weathered O_3 concentrations and their sensitivity trends from 2020–2023, providing empirical validation for our CDTR framework's ability to capture dynamic signals missed by conventional sensitivity zoning methods. "This field observation of a cooccurring plateau in the evolutionary trends of the de-weathered O_3 concentration and sensitivity indicators provides real-world validation of the feasibility of our proposed CDTR analytical method for capturing dynamic O_3 photochemical system behaviour signals. " (lines 140–143).

Visual Presentation of Path Dependency (Lines 298– 304):

"Supplementary Fig. 6 provides a visual demonstration of the path dependency inherent in O_3 abatement, revealing that the choice of emission reduction pathway dictates the evolution trajectory of the O_3 photochemical system. Specifically, under identical reduction pathways, the evolution trajectories across the three megacity clusters exhibit convergence; however, when different precursor reduction scenarios (i.e., distinct reduction pathways) are implemented, their O_3-FNR evolution trajectories significantly diverge. "

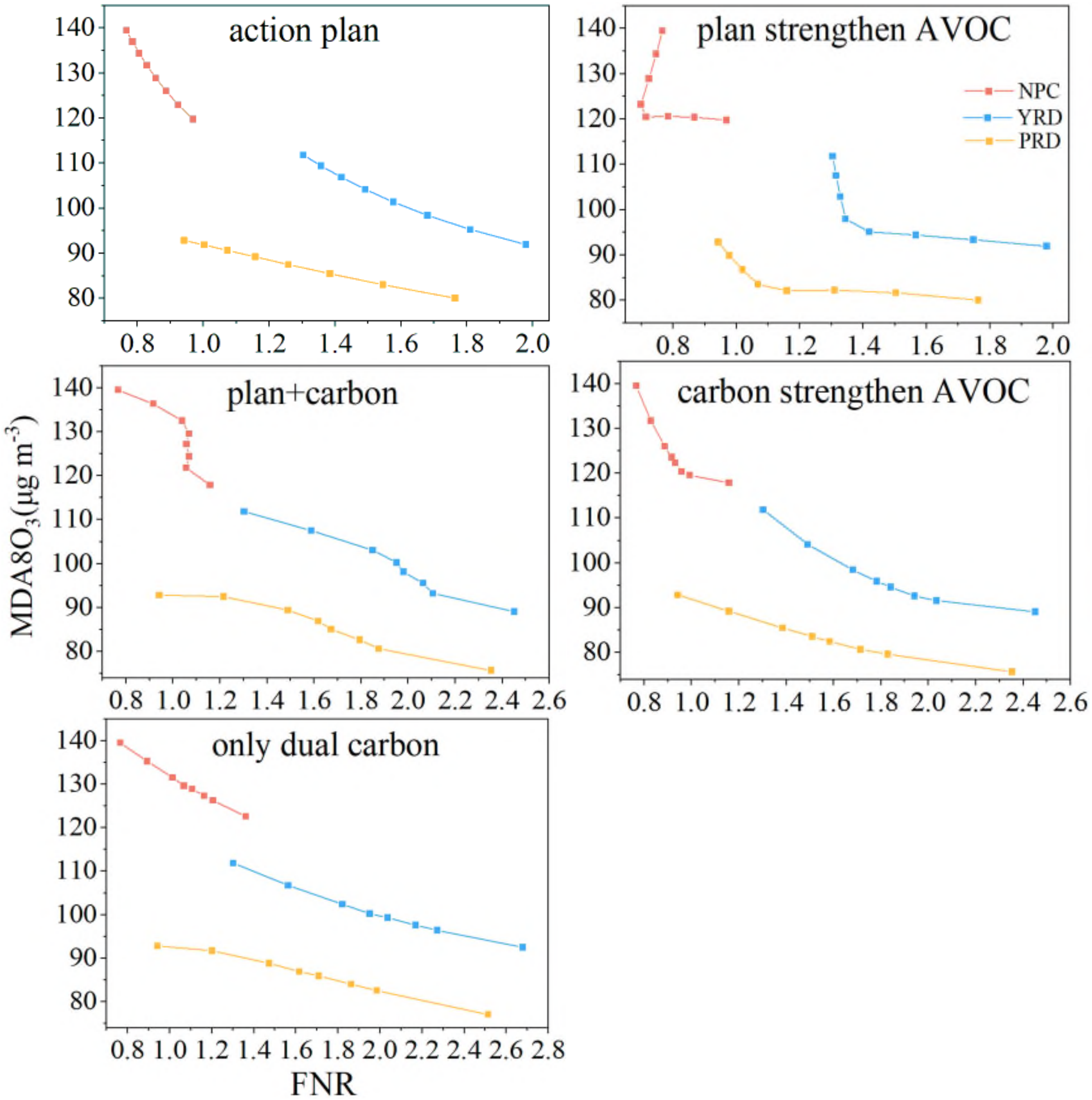

Supplementary Fig. 6. Path dependency in O_3 mitigation revealed by 2020–2060 model simulations: Distinct emission reduction pathways drive O_3 photochemical system into divergent evolution trajectory. To isolate the impact of anthropogenic emission changes, meteorological conditions and BVOC emissions were fixed at 2020 levels.

(2) Quantification of the 'AVOCs Reduction Bonus Window' and Critical Tipping Points: We quantitatively project future optimal O_3 mitigation strategies and, crucially, identify and quantify two critical tipping points in the evolution of the O_3 photochemical system(Supplementary Fig. 7c and Fig.5), thereby determining the duration of the 'AVOCs reduction bonus window' (lines 345–355, Fig. 5). This nuanced understanding of the optimal timing for precursor reductions under climate-air quality synergy is a significant innovative contribution.

“Our projections (Fig. 5 and Supplementary Fig. 7) reveal that China's megacity clusters cross these tipping points asynchronously. The NCP and PRD have already crossed the 'ridgeline' by 2025 , entering the transition regime, while the YRD follows suit later (Supplementary Fig. 7c). More critically, the 'bonus window' for AVOC control is projected to close earliest for the PRD around 2045, followed by the YRD around 2050. In contrast, the NCP region maintains a persistent, though diminishing, AVOC control bonus beyond 2050, indicating a more prolonged window of opportunity (Fig.5). This quantitative comparison is made possible by paired scenarios to disentangle the respective contributions of equiproportional AVOC and NO*x* reductions on MDA8 O_3 mitigation (see Methods and Supplementary Fig.7 for detailed derivation).

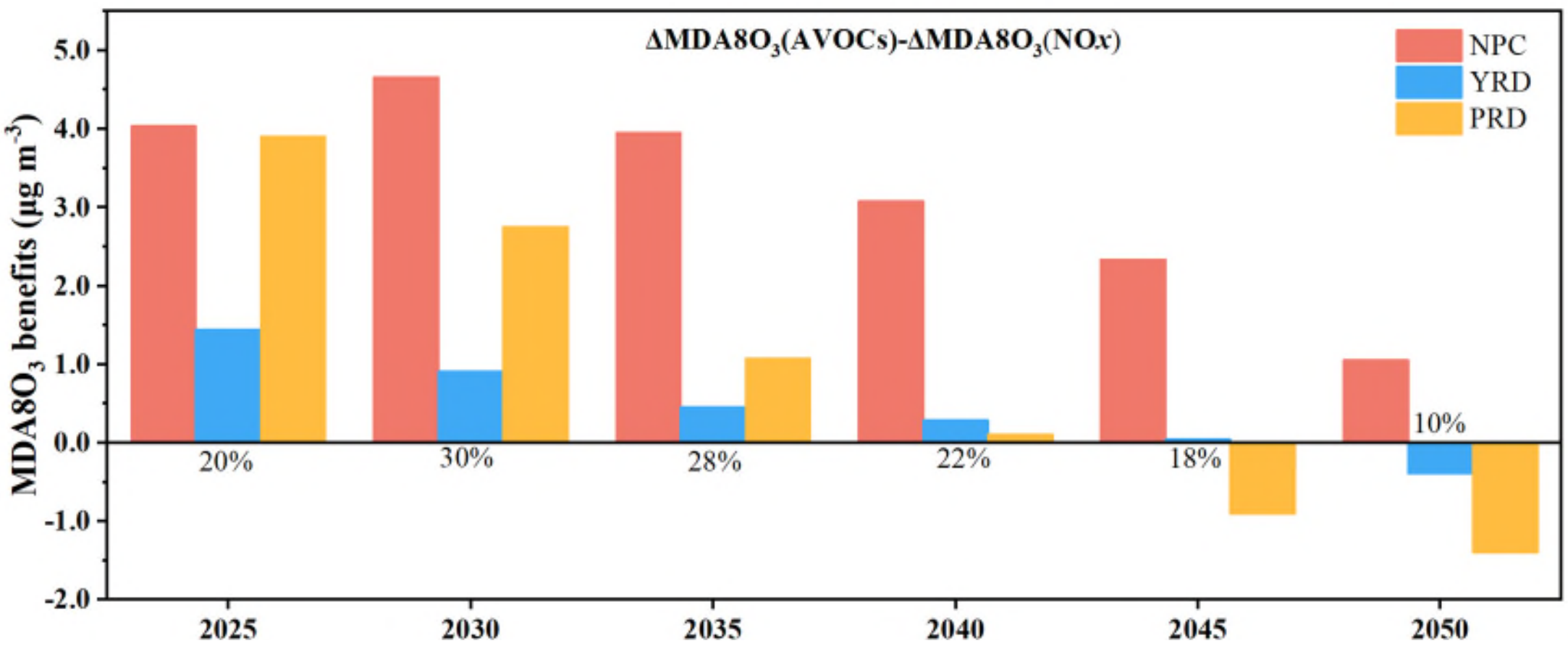

Fig. 5. Dynamics of the AVOC reduction bonus window in China’s three megacity clusters. The bonus window for prioritizing AVOCs is considered closed when its net MDA8 O_3 mitigation benefit—defined as the difference in O_3 reduction between reduction AVOCs versus an equivalent proportion of NO*x*—becomes negative. Percentages denote the precursor reduction fraction for the corresponding year.

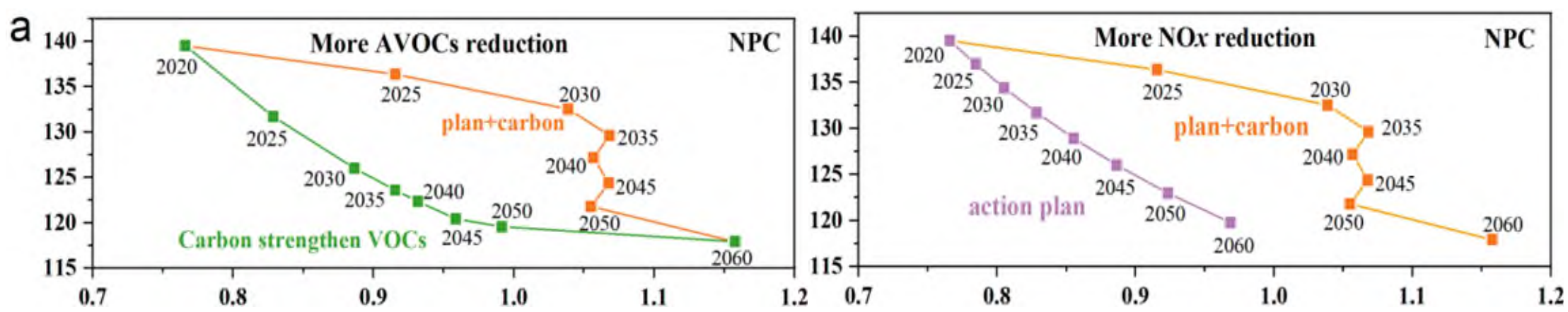

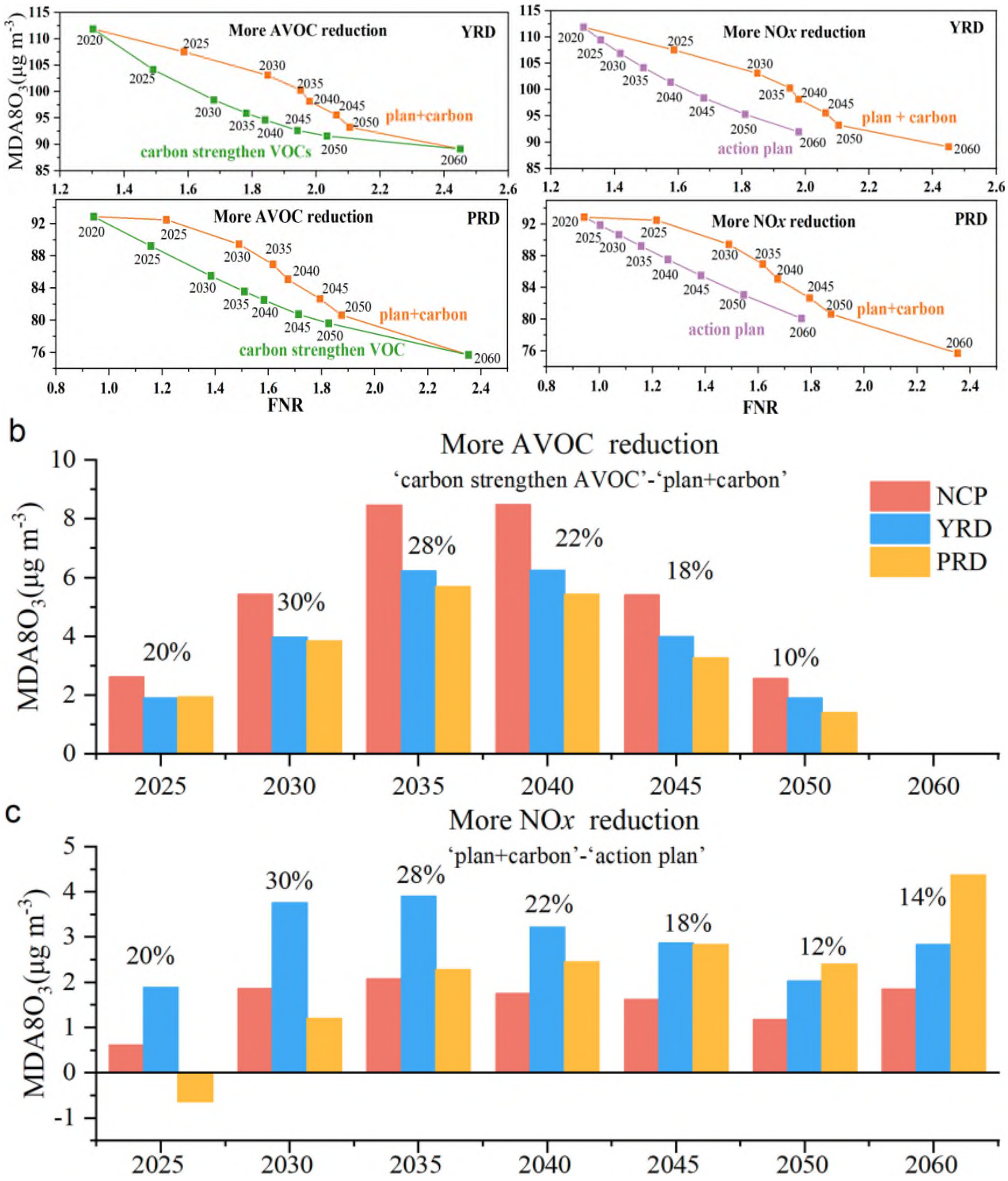

Supplementary Fig. 7. Derivation of isolated O_3 reduction benefits from AVOC versus equivalent proportional NO*x* reduction. a, the evolution trajectory of O_3 photochemical system (O_3-FNR) of paired-scenario designed to decouple the effects of AVOC versus NO*x* reductions in China's three megacity clusters from 2020 to 2060. The 'More AVOCs reduction' comparison isolates the impact of AVOCs by comparing the 'carbon strengthen AVOCs' and 'plan+carbon' scenarios, which share an identical NO*x* trajectory. The 'More NO*x* reduction' comparison isolates the impact of NO*x* by comparing the 'plan+carbon' and 'action plan' scenarios, which share an identical AVOC trajectory. **b, Isolated MDA8O_3**

reduction from dedicated AVOC controls, calculated as the MDA8O_3 difference between the 'carbon strengthen AVOCs' and 'plan+carbon' scenarios. Percentages indicate the additional AVOC reduction for that year. **c, Isolated MDA8 O_3 reduction from dedicated NO*x* controls**, calculated as the MDA8 O_3 difference between the 'plan+carbon' and 'action plan' scenarios. Percentages indicate the additional NO*x* reduction for that year."

(3) Adaptive CDTR Framework: We demonstrate how the CDTR framework can continuously assess and refine the optimal control pathway as the real-world atmospheric composition evolves (including changes in BVOC concentrations), providing timely and actionable feedback for the adaptive management of O_3 governance (lines 486–533). This establishes a new, globally applicable analytical framework for designing adaptive air quality-climate co-governance schemes.

"Similarly, we acknowledge that the evolving speciation of AVOCs can influence the predicted efficacy of O_3 abatement. Given the differential photochemical reactivities among VOC species[39-41], the strategic focus of reduction—for instance, prioritizing highly ozone formation potential species (high -OFP) like alkenes or aromatics—would undoubtedly influence the duration of the 'AVOC reduction bonus window' and the MDA8 O_3 mitigation effect. Although the emission scenarios in Fig. 4 were simulated by applying mass-based reductions to the 2020 AVOC emission inventory, the inherent adaptability of the our CDTR framework equips it to address the challenge posed by evolving species. It is not a static, one-off prediction tool but a dynamic analytical framework. Its observational component continuously integrate multi-source real-time data, including satellite and ground-based monitoring, to evaluate the response of deweathered MDA8 O_3 concentrations and sensitivity trends to evolving precursors caused by different policy and activity pattern (e.g. COVID-19). Simultaneously, its modeling component can be iteratively updated by incorporating updated emission inventories and leveraging the DPEC model. The DPEC model is specifically tasked to predict dynamic future emissions by integrating anticipated socioeconomic development, technological advancements, and evolving clean air and climate policies into the CDTR analytical framework (see Methods). To illustrate this, we employed the CDTR framework to further explore the impacts of reactivity-based AVOC control strategies. We calculated precursor reduction ratios of prioritizing completely removal of high-OFP

species (alkenes and aromatics), and then compared the outcomes with those from the mass-based AVOC reductions control strategies with equal-proportion reduction of total AVOCs (see Methods, Supplementary Figs. 8–9, and Supplementary Tables 9–11). The results indicate that prioritizing high-OFP species significantly accelerates the evolution of O_3 sensitivity towards the NO*x*-limited regime, as evidenced by markedly higher FNR values across all scenarios compared to equal-proportion reduction (Supplementary Fig. 8). However, this did not uniformly translate into superior O_3 concentration mitigation benefits (Supplementary Fig. 9). Specifically, only under the 'plan+carbon' scenario, which involves deep NO*x* reductions, did this strategy show a clear advantage in reducing MDA8 O_3 concentrations in the NCP and YRD. Conversely, in scenarios with less aggressive NO*x* control, its efficacy was even inferior to that of the mass-based control strategy. In the PRD megacity cluster under the 'plan+carbon' scenario, the MDA8 O_3 mitigation benefit of reactivity-based reduction proved less effective than that of the mass-based reduction, potentially due to the PRD entering the O_3 formation transition regime later than the other two megacity clusters (Supplementary Fig. 7c). The varying effectiveness of the reactivity-based reduction strategy reveals a strong path dependency, quantitatively demonstrating that the efficacy of control strategies is highly contingent on the evolutionary stage of the O_3 photochemical system. In the early stages of the AVOC reduction bonus window (Fig. 5), the concentrated reduction of high-OFP species may be less efficient than the broad reduction of a larger mass of various AVOCs. Therefore, these findings do not negate the value of reactivity-based strategies but rather underscore the necessity of precision timing in their implementation. This validates the adaptive nature of our proposed CDTR framework: it provides an essential dynamic diagnostic capability to assess the state of the photochemical system in time, thereby determining the optimal timing to transition from mass-based control to more refined, reactivity-based control, ensuring scientific and efficient policy adjustments."

We are confident that these comprehensive revisions now clearly highlight the unique findings and innovative contributions of our work, demonstrating how it advances the understanding and strategic planning of O_3 control in the context of a changing climate.

2. Furthermore, the quality of the figures needs significant improvement. Each figure contains multiple subpanels, but they are not labeled with numbers or letters, which makes them difficult to reference in the text. The axes should be clearly defined and large enough to be easily legible, and the figure legends should be enlarged for readability. In addition, some figures include shaded areas, but these are not explained in the figure captions and should be clarified.

Reply: In response to the reviewer's comments, we have undertaken a comprehensive revision of all figures in the manuscript to significantly improve their clarity, legibility, and professional presentation.

Other general comments:

3. When defining the emission reduction scenarios, for the "strengthen VOC" scenario is the reduction is set over total VOCs? If so, wouldn't it be interesting to explore the impact of targeting specific VOC species, particularly the most reactive ones that contribute most to ozone formation, or specific sectors? Several studies in China and elsewhere have highlighted the benefits of reactivity-based mitigation strategies. For example, Ou et al. (2015) and Derwent et al. (2007) demonstrated that focusing on high-OFP species provides more effective ozone control than emission-based approaches. It would be interesting to see the impact when incorporating such an approach, as it could strengthen the narrative of the paper by demonstrating even greater effectiveness of VOC control strategies.

Refs:

Jiamin Ou, Junyu Zheng, Rongrong Li, Xiaobo Huang, Zhuangmin Zhong, Liuju Zhong, Hui Lin, Speciated OVOC and VOC emission inventories and their implications for reactivity-based ozone control strategy in the Pearl River Delta region, China, Science of The Total Environment, Volumes 530–531, 2015, Pages 393-402, ISSN 0048-9697, [https://doi.org/10.1016/j.scitotenv.2015.05.062.](https://doi.org/10.1016/j.scitotenv.2015.05.062)

R.G. Derwent, M.E. Jenkin, N.R. Passant, M.J. Pilling, Reactivity-based strategies for photochemical ozone control in Europe, Environmental Science & Policy, Volume 10, Issue 5, 2007, Pages 445-453, ISSN 1462-9011, [https://doi.org/10.1016/j.envsci.2007.01.005.](https://doi.org/10.1016/j.envsci.2007.01.005)

Reply: We are sincerely grateful to the reviewer for this insightful and constructive suggestion. We fully agree that exploring the impact of reactivity-based control strategies, which target high Ozone Formation Potential (OFP) species, is a critical extension to our analysis. The reviewer rightly points out that our original scenarios were based on mass-based reductions of total AVOCs. In response to this excellent suggestion, and to further strengthen the scientific narrative of our paper, we have conducted a new set of simulation experiments to quantitatively assess the efficacy of reactivity-based controls.

This extensive new analysis is now detailed in the revised manuscript, including:

1. A new, dedicated paragraph in the **Discussion** section (lines 503-533).

2. A new, detailed methodological section, "Derivation of NO*x*-to-AVOC reduction ratios for reactivity-based O_3 control strategy tests" in the **Methods** (lines 702-740).

3. New Supplementary Figures 8 and 9, which visualize the FNR and MDA8 O_3 trajectories under reactivity-based control scenarios.

4. New Supplementary Tables 9-11, which provide the detailed reduction ratios for these tests.

Our analysis reveals a complex and path-dependent outcome that goes beyond a simple conclusion of "reactivity-based control is always more effective." The results show that the efficacy of prioritizing high-OFP species (alkenes and aromatics) is highly contingent on the evolutionary stage of the O_3 photochemical system.

Specifically, as now detailed in the **Discussion** (lines 503-533) and shown in Supplementary Fig. 9, the reactivity-based strategy demonstrated a clear advantage in reducing MDA8 O_3 concentrations only under the 'plan+carbon' scenario, which features deep NO*x* reductions. Conversely, in scenarios with less aggressive NO*x* control, its efficacy was sometimes even inferior to the mass-based strategy.

Crucially, this nuanced finding does not negate the value of reactivity-based strategies. Instead, it powerfully underscores the central thesis of our paper: the critical need for precision timing and adaptive management in O_3 control. This result validates the adaptive nature of our proposed CDTR framework. As we now state in the manuscript (lines 530-533), the CDTR framework "provides an essential dynamic diagnostic capability to assess the state of the photochemical system in time, thereby

determining the optimal timing to transition from mass-based control to more refined, reactivity-based control, ensuring scientific and efficient policy adjustments".

We believe this major addition, prompted by the reviewer's valuable feedback, has substantially strengthened our manuscript. It transforms the discussion from a static comparison of strategies into a dynamic demonstration of how our CDTR framework can guide adaptive, multi-stage policymaking. We once again thank the reviewer for motivating this significant improvement.

Specific revisions are provided below:

Revised main text lines 503-533:

"To illustrate this, we employed the CDTR framework to further explore the impacts of reactivity-based AVOC control strategies. We calculated precursor reduction ratios of prioritizing completely removal of high-OFP species (alkenes and aromatics), and then compared the outcomes with those from the mass-based AVOC reductions control strategies with equal-proportion reduction of total AVOCs (see Methods, Supplementary Figs. 8–9, and Supplementary Tables 9–11). The results indicate that prioritizing high-OFP species significantly accelerates the evolution of O_3 sensitivity towards the NO*x*-limited regime, as evidenced by markedly higher FNR values across all scenarios compared to equal-proportion reduction (Supplementary Fig. 8). However, this did not uniformly translate into superior O_3 concentration mitigation benefits (Supplementary Fig. 9). Specifically, only under the 'plan+carbon' scenario, which involves deep NO*x* reductions, did this strategy show a clear advantage in reducing MDA8 O_3 concentrations in the NCP and YRD. Conversely, in scenarios with less aggressive NO*x* control, its efficacy was even inferior to that of the mass-based control strategy. In the PRD megacity cluster under the 'plan+carbon' scenario, the MDA8 O_3 mitigation benefit of reactivity-based reduction proved less effective than that of the mass-based reduction, potentially due to the PRD entering the O_3 formation transition regime later than the other two megacity clusters (Supplementary Fig. 7c). The varying effectiveness of the reactivity-based reduction strategy reveals a strong path dependency, quantitatively demonstrating that the efficacy of control strategies is highly contingent on the evolutionary stage of the O_3 photochemical system. In the early stages of the AVOC reduction bonus window (Fig. 5), the concentrated reduction of high-OFP species may be less efficient than the broad reduction of a larger mass of various AVOCs. Therefore, these findings do not

negate the value of reactivity-based strategies but rather underscore the necessity of precision timing in their implementation. This validates the adaptive nature of our proposed CDTR framework: it provides an essential dynamic diagnostic capability to assess the state of the photochemical system in time, thereby determining the optimal timing to transition from mass-based control to more refined, reactivity-based control, ensuring scientific and efficient policy adjustments.

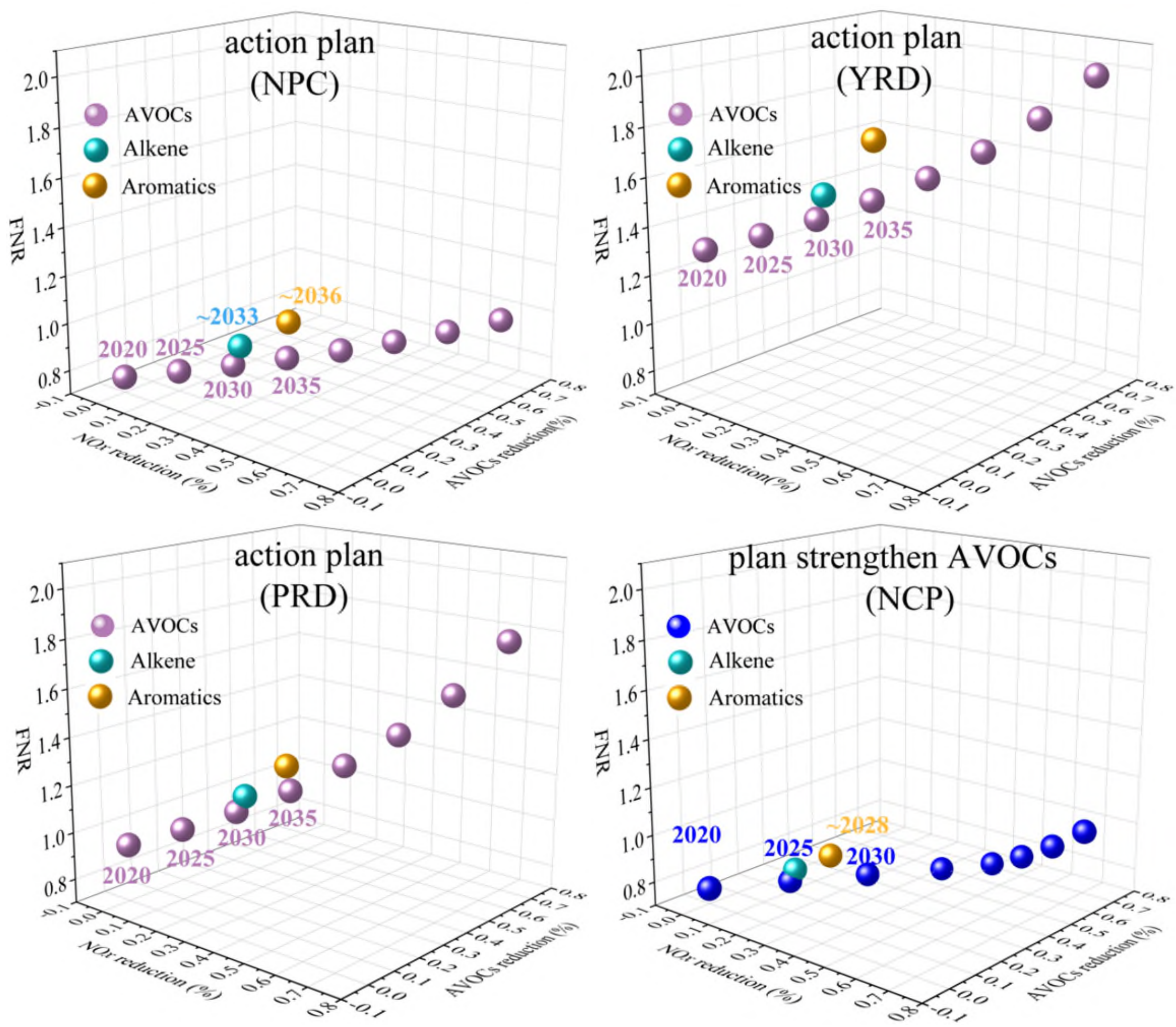

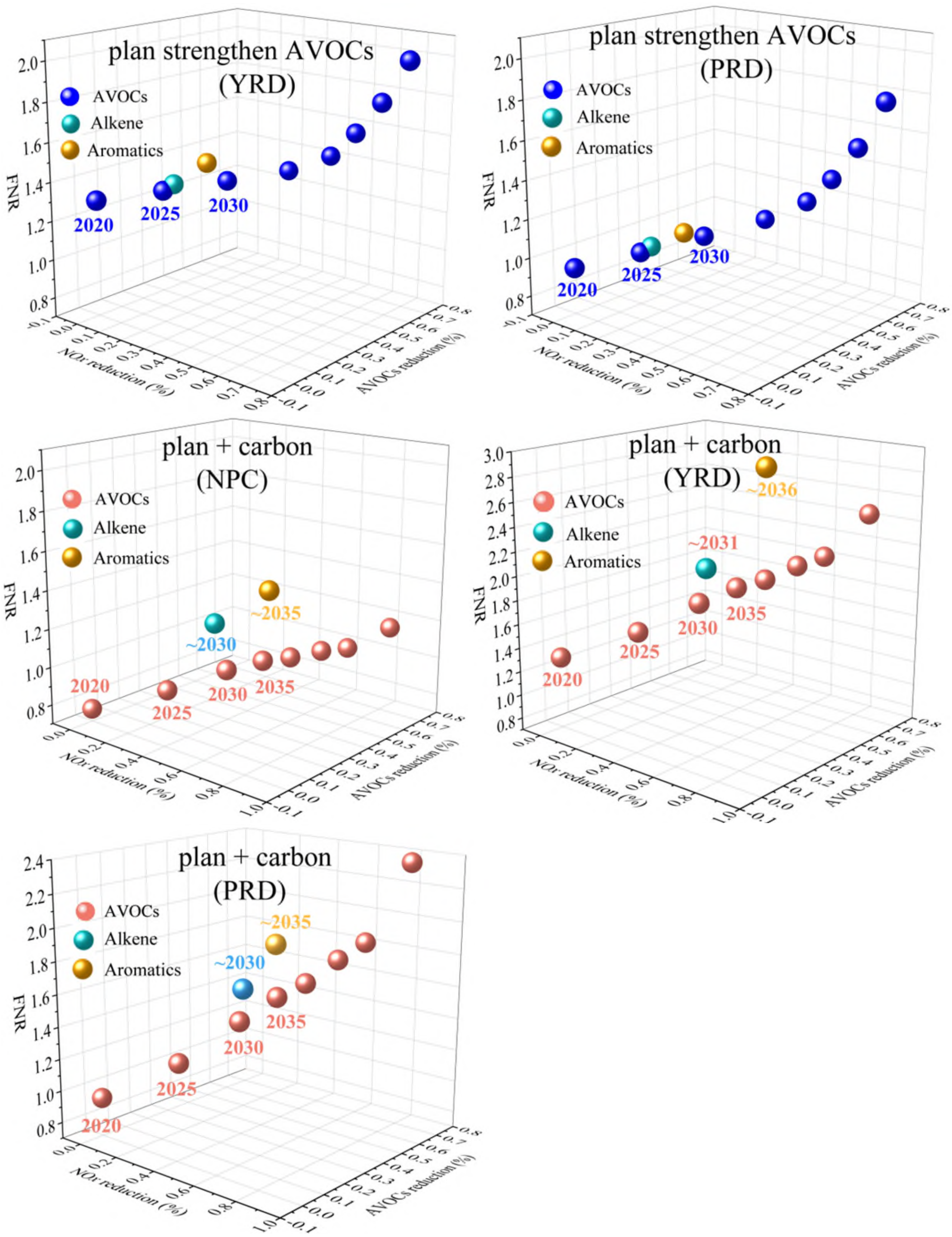

Supplementary Fig. 8. The FNR trajectory of different megacity clusters under different NOx:AVOCs reduction. AVOCs represent equal reduction all species, Alkene represents all alkene species of MEIC inventory were reduced, and Aromatics represent all aromatic compounds were reduced. The BVOCs emission and meteorological field input are fixed at the 2020 level.

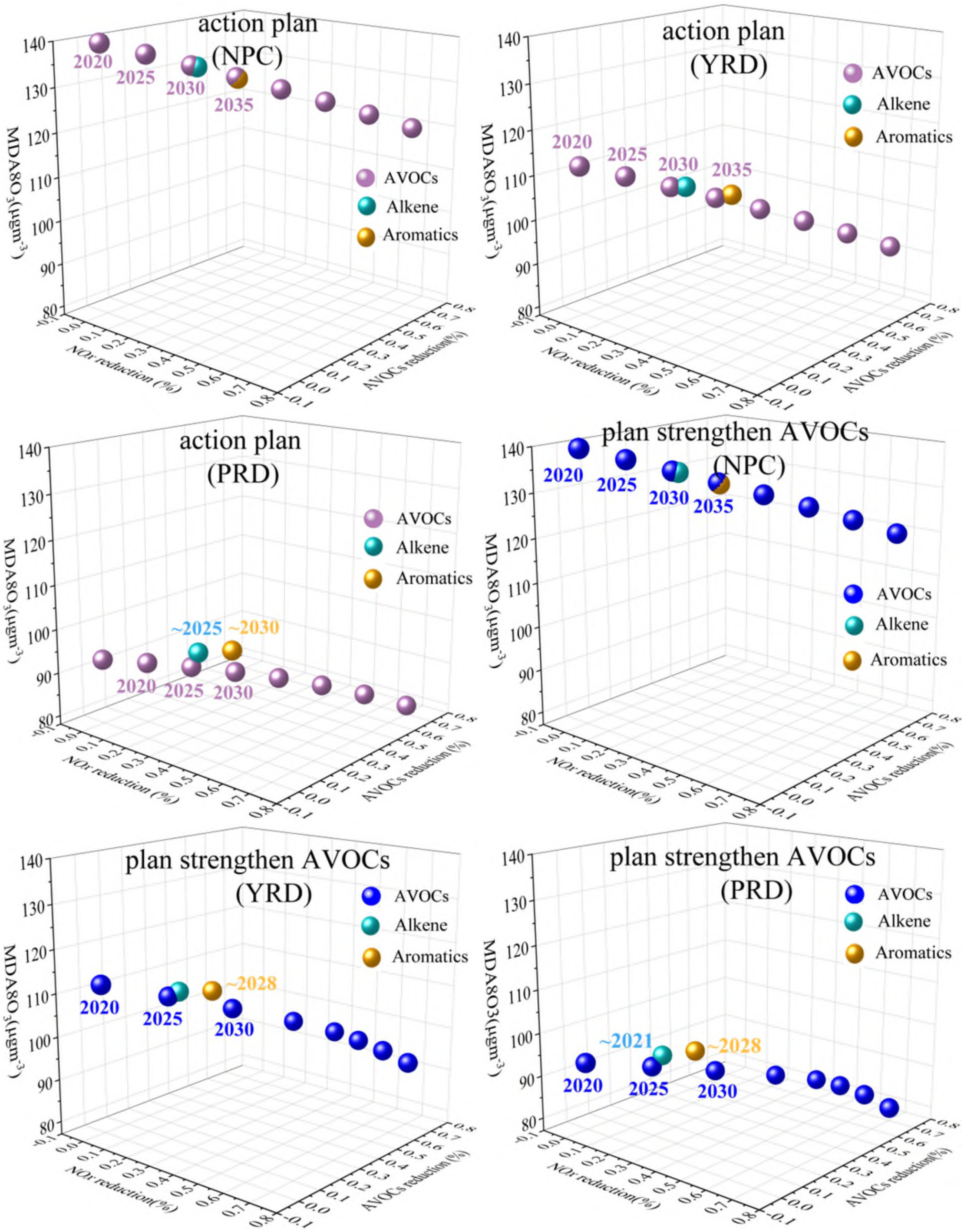

action plan
(NPC)
2020
2025
2030
2035
AVOCs
Alkene
Aromatics
MDA8O3(μgm-3)
NOx reduction (%)
AVOCs reduction(%)
action plan
(YRD)
2020
2025
2030
2035
AVOCs
Alkene
Aromatics
MDA8O3(μgm-3)
NOx reduction (%)
AVOCs reduction (%)
action plan
(PRD)
~2025
~2030
2020
2025
2030
AVOCs
Alkene
Aromatics
MDA8O3(μgm-3)
NOx reduction (%)
AVOCs reduction (%)
plan strengthen AVOCs
(NPC)
2020
2025
2030
2035
AVOCs
Alkene
Aromatics
MDA8O3(μgm-3)
NOx reduction(%)
AVOCs reduction(%)
plan strengthen AVOCs
(YRD)
2020
2025
~2028
2030
AVOCs
Alkene
Aromatics
MDA8O3(μgm-3)
NOx reduction(%)
AVOCs reduction(%)
plan strengthen AVOCs
(PRD)
~2021
~2028
2020
2025
2030
AVOCs
Alkene
Aromatics
MDA8O3(μgm-3)
NOx reduction(%)
AVOCs reduction(%)

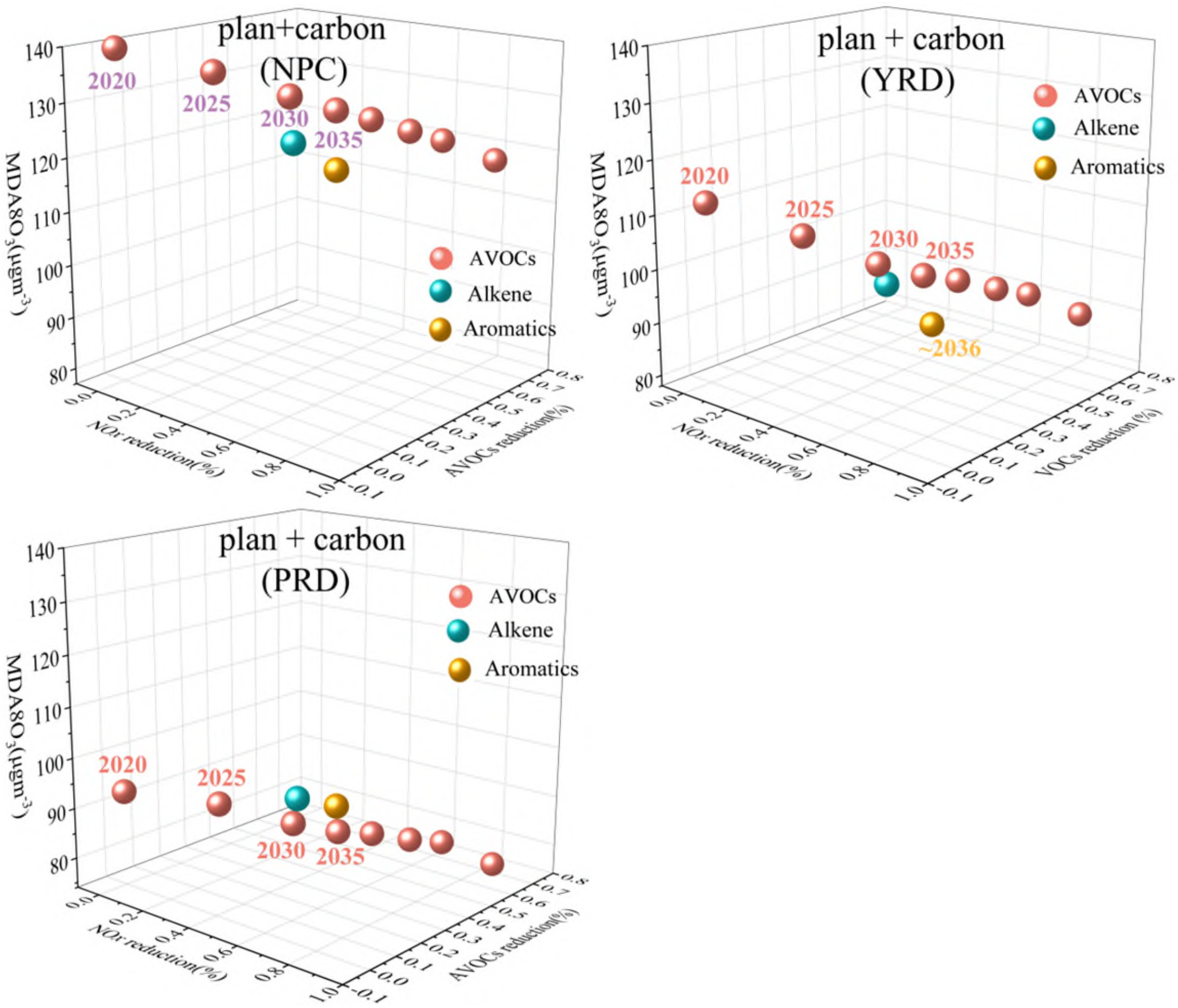

Supplementary Fig. 9. The MDA8O$_3$ concentrations trajectory of different megacity clusters under different NOx:AVOCs reduction. AVOCs represent equal reduction all species, Alkene represents all alkene species of MEIC inventory were reduced, and Aromatics represent all aromatic compounds were reduced. The BVOCs emission and meteorological field input are fixed at the 2020 level.

Methods (lines 702-740):

Derivation of NO*x*-to-AVOC reduction ratios for reactivity-based O$_3$ control strategy tests

To quantitatively assess the differential impacts of reactivity-based versus mass-based O$_3$ control strategies from 2020 to 2060, we designed a series of tests to compare the effects of the two strategies on O$_3$ sensitivity trends and on MDA8 O$_3$ concentrations under fixed meteorological and biogenic emission conditions (Supplementary Figs. 8–9) across different scenarios (‘action plan’, ‘plan strengthen AVOCs’, and ‘plan + carbon’). In the tests of reactivity-based strategies, we targeted the reduction of high Ozone Formation Potential (OFP) species (alkenes and

aromatics), and determined the corresponding NO_x and AVOC reduction ratios according to the following procedure:

First, based on the MEIC inventory for June–August 2020, we calculated the total emissions of alkenes and aromatics in the three megacity clusters (NCP, YRD, and PRD). Note that alkenes here refer exclusively to anthropogenic sources, excluding biogenic isoprene.

Subsequently, the emissions of these high-OFP species were divided by the total AVOC emissions for the same period to derive the mass fraction for alkenes and aromatics in each megacity clusters.

Finally, based on the NO_x:AVOC reduction ratios for different scenarios as defined in Table 1, we determined the corresponding NO_x reduction targets when prioritizing the reduction of these high-OFP species (see Supplementary Tables 9–11):

1. ‘action plan’ scenario: This scenario adheres to a 1:1 coordinated NO_x:AVOC reduction ratio. Accordingly, when alkenes (or aromatics) were preferentially abated, the NO_x reduction percentage was set to be equal to the mass fraction of that specific species group.

2. ‘plan strengthen AVOCs’ scenario: This scenario adheres to a 1:2 coordinated NO_x:AVOC reduction ratio. The NO_x reduction percentage was therefore set to half the mass fraction of the targeted alkene (or aromatic) group.

3. ‘plan + carbon’ scenario: As the NO_x reduction pathway for this scenario is derived from the ‘only dual carbon’ scenario, we employed a "trajectory mapping" approach. Specifically, we matched the 2020 mass fraction of alkenes or aromatics to the year in the ‘only dual carbon’ scenario’s AVOC reduction trajectory with the closest reduction percentage. The NO_x reduction percentage from that matched year was then adopted as the corresponding NO_x reduction target. For instance, in the summer of 2020, aromatics in the NCP region constituted 30.3% of total AVOC emissions. This value most closely matches the AVOC reduction level for the year 2030 in the ‘only dual carbon’ scenario. Therefore, the corresponding NO_x reduction target of 58% for that year was adopted.

Supplementary Table 9. The reduction proportions of NO*x* : AVOCs from 2020 to 2060 under 3 different scenarios in NPC megacity clusters. AVOCs represent equal reduction all species, Alkene represents all alkene species of MEIC inventory were reduced, and Aromatics represent all aromatic compounds were reduced. The base emission amount is fixed at 2020.

Year	Species reduction	action plan	Year	Species reduction	plan strengthen AVOCs	Year	Species reduction	plan+carbon
2025	AVOCs	10%:10%	2025	AVOCs	10%:20%	2025	AVOCs	30%:10%
2030	AVOCs	20%:20%		Alkene	10.6%:21.3%	2030	AVOCs	50%:20%
	Alkene	21.3%:21.3%		Aromatics	15.2%:30.3%		Alkene	50%:21.3%
2035	AVOCs	30%:30%	2030	AVOCs	20%:40%	2035	AVOCs	58%:30%
	Aromatics	30.3%:30.3%	2035	AVOCs	30%:60%		Aromatics	58%:30.3%
2040	AVOCs	40%:40%	2040	AVOCs	40%:70%	2040	AVOCs	62%:40%
2045	AVOCs	50%:50%	2045	AVOCs	50%:70%	2045	AVOCs	68%:50%
2050	AVOCs	60%:60%	2050	AVOCs	60%:70%	2050	AVOCs	72%:60%
2060	AVOCs	70%:70%	2060	AVOCs	70%:70%	2060	AVOCs	84%:70%

Supplementary Table 10. The reduction proportions of NO*x* : AVOCs from 2020 to 2060 under 3 different scenarios in YRD megacity clusters. AVOCs represent equal reduction all species, Alkene represents all alkene species of MEIC inventory were reduced, and Aromatics represent all aromatic compounds were reduced. The base emission amount is fixed at 2020.

Year	Species reduction	action plan	Year	Species reduction	plan strengthen VOCs	Year	Species reduction	plan+carbon
2025	AVOCs	10%:10%	2025	AVOCs	10%:20%	2025	AVOCs	30%:10%
2030	AVOCs	20%:20%		Alkene	11.6%:23.3%	2030	AVOCs	50%:20%
	Alkene	23.3%:23.3%		Aromatics	16.8%:33.6%		Alkene	50%:23.3%
2035	AVOCs	30%:30%	2030	AVOCs	20%:40%	2035	AVOCs	58%:30%
	Aromatics	33.6%:33.6%	2035	AVOCs	30%:60%		Aromatics	58%:33.6%
2040	AVOCs	40%:40%	2040	AVOCs	40%:70%	2040	AVOCs	62%:40%
2045	AVOCs	50%:50%	2045	AVOCs	50%:70%	2045	AVOCs	68%:50%
2050	AVOCs	60%:60%	2050	AVOCs	60%:70%	2050	AVOCs	72%:60%
2060	AVOCs	70%:70%	2060	AVOCs	70%:70%	2060	AVOCs	84%:70%

Supplementary Table 11. The reduction proportions of NO*x* : AVOCs from 2020 to 2060 under 3 different scenarios in PRD megacity clusters. AVOCs represent equal reduction all species, Alkene represents all alkene species of MEIC inventory were reduced, and Aromatics represent all aromatic compounds were reduced. The base emission amount is fixed at 2020.

Year	Species reduction	action plan	Year	Species reduction	plan strengthen VOCs	Year	Species reduction	plan+carbon
2025	AVOCs	10%:10%	2025	AVOCs	10%:20%	2025	AVOCs	30%:10%
2030	AVOCs	20%:20%		Alkene	10.8%:21.6%	2030	AVOCs	50%:20%
	Alkene	21.6%:21.6%		Aromatics	14.6%:29.3%		Alkene	50%:21.6%
	Aromatics	29.3%:29.3%	2030	AVOCs	20%:40%	2035	AVOCs	58%:30%
2035	AVOCs	30%:30%	2035	AVOCs	30%:60%		Aromatics	58%:29.3%
2040	AVOCs	40%:40%	2040	AVOCs	40%:70%	2040	AVOCs	62%:40%
2045	AVOCs	50%:50%	2045	AVOCs	50%:70%	2045	AVOCs	68%:50%
2050	AVOCs	60%:60%	2050	AVOCs	60%:70%	2050	AVOCs	72%:60%
2060	AVOCs	70%:70%	2060	AVOCs	70%:70%	2060	AVOCs	84%:70%

4. Assuming fixed biogenic VOC (BVOC) emissions up to 2060 represents an important limitation, as BVOCs are strongly temperature-dependent. Under warming scenarios, their contribution to ozone formation is likely to increase, particularly as anthropogenic VOCs are reduced, which could alter the long-term sensitivity of ozone to VOC controls. Could the authors comment on this? Additionally, L.504 the authors mentions that the method relies on dynamic emission changes, it should be clarified that these dynamics apply only to AVOCs.

Reply: We sincerely thank the reviewer for their insightful comments on our manuscript, We have carefully addressed each point, and our detailed responses are provided below.

(1) Regarding fixed biogenic VOC (BVOC) emissions:

We fully agree that BVOCs exhibit a strong temperature dependency, and future climate warming is likely to increase their contribution to O_3 formation, potentially altering the long-term sensitivity of O_3 to AVOC controls.

We have already addressed this important limitation and its implications in detail within the **Discussion** section of our revised manuscript (Main Text, Lines 464-477):

"To isolate the independent effects of anthropogenic emission abatement, our future projections fixed meteorological fields and BVOC emissions at 2020 baseline levels. However, future climate change, characterized by projected rising temperatures and more frequent heatwaves, is expected to increase BVOC emissions[35]. This would drive the photochemical environment towards more VOC-rich and thus more NO*x*-limited state. This means future increases in BVOCs would likely accelerate the transition to a NO*x*-limited regime and thus result in a shorter period for AVOC reduction bonus window. However, this prospect does not undermine our core conclusion; rather, it magnifies the strategic urgency of capitalizing on this window of opportunity for AVOCs abatement before it closes. More complex climate-O_3 interactions, such as temperature effects on agricultural NO*x* and AVOCs emissions[35], and anthropogenic NO*x* and AVOCs emission pattern change caused by heatwave-driven shifts in energy structure[36], remain critical areas for future investigation."

As elaborated above, we acknowledge this was a simplifying assumption made to isolate the independent effects of anthropogenic emissions. We explicitly state that increased BVOCs due to climate change could accelerate the transition to a

NO_x-limited regime, potentially shortening the AVOC reduction 'bonus window'. Importantly, this prospect does not undermine our core conclusion; rather, it reinforces it by intensifying the strategic urgency of early AVOC abatement. We have also designated these more complex climate-O_3 interactions as a critical area for future investigation.

(2) Regarding the statement in Line 504: "the method relies on dynamic emission changes."

We thank the reviewer for their request for clarification regarding the statement in Line 504. The statement, "the method relies on dynamic emission changes," refers to the inherent capability of the CDTR framework, which is designed to process and analyze the long-term, comprehensive dynamic changes in precursor emissions (including both NO_x and VOCs) driven by multiple policy objectives (clean air and climate actions). This does not imply that these dynamic changes apply only to AVOCs. In our revised manuscript, we have revised this to "To address the limitations of traditional static sensitivity zoning methods in formulating effective O_3 mitigation strategies amidst multi-decadal, dynamic changes in precursor emission (including both NO_x and VOCs), ...(Lines 556-558)"

In our 'Scenario Setting' section (Revised Methods, Lines 648-660, Lines 607-611), we explicitly elaborate on how NO_x and AVOC emission reductions are dynamically handled in climate-oriented scenarios:

Revised Methods, Lines 648-660：

"In contrast, our three climate-oriented scenarios ('only dual carbon', 'carbon strengthen AVOCs' and 'plan + carbon') employ dynamic precursor reduction proportions relative to 2020 levels from the DPEC in China[27,28]. These dynamic reduction proportions reflect anticipated future emission changes driven by socioeconomic development, climate actions, and advancements in pollution control technology. Specifically, because NO_x emissions are more closely linked to energy transitions towards renewables under carbon neutrality actions than to AVOCs[10], we determined NO_x emission reduction trajectories by referencing the *Ambitious-Pollution-Neutral-Goals* (APNG) scenario within the DPEC model[27,28]. The DPEC-APNG scenario projects that China will achieve carbon neutrality by 2060, with a CO_2 emission trajectory between representative concentration pathways 1.9

and 2.6. This approach ensures that our emission reduction scenarios better represent potential real-world dynamics. ”

Revised Methods, Lines 607-611

“The integration of the DPEC model into our scenario design also equips the CDTR analytical framework with the capability for iterative updates, allowing it to dynamically reflect evolving socioeconomic trajectories, advancements in pollution control technologies, and evolving clean air polices and climate actions.”

Furthermore, the revised Table 1 clearly illustrates the dynamic reduction percentages for both NO*x* and AVOCs across all five projected scenarios. For instance, in the 'only dual carbon' scenario, NO*x* reductions increase from 30% in 2025 to 84% in 2060, while AVOC reductions rise from 15% to 42%, highlighting the dynamic evolution of both precursors and their shifting ratio.

Table 1. The reduction proportions of NO*x* : AVOCs from 2020 to 2060 under 5 different scenarios. The base emission amount is fixed at 2020.

Year	action plan	plan strengthen AVOCs	plan+carbon	carbon strengthen AVOCs	only dual carbon
2025	10%:10%	10%:20%	30%:10%	30%:30%	30%:15%
2030	20%:20%	20%:40%	50%:20%	50%:50%	50%:25%
2035	30%:30%	30%:60%	58%:30%	58%:58%	58%:30%
2040	40%:40%	40%:70%	62%:40%	62%:62%	62%:31%
2045	50%:50%	50%:70%	68%:50%	68%:68%	68%:34%
2050	60%:60%	60%:70%	72%:60%	72%:70%	72%:36%
2060	70%:70%	70%:70%	84%:70%	84%:70%	84%:42%

Therefore, the CDTR framework is designed to capture the coupled dynamic responses to dynamic changes in both NO*x* and AVOC emissions, not just AVOCs. To further clarify our conclusions, we have explicitly specified 'AVOCs' in the revised manuscript wherever it was intended.

5. ***Several studies have shown that future increases in CH_4 can significantly influence O_3 production. Could you clarify how CH_4 was treated in your modeling***

framework? For instance, was CH_4 held constant, or varied according to projected scenarios?

Reply: We are sincerely grateful to the reviewer for this insightful and important question regarding the treatment of methane (CH_4) in our modeling framework. We agree that clarifying the handling of this crucial global O_3 precursor is essential for methodological transparency and for contextualizing our findings. In the original manuscript, this detail was indeed not explicitly stated.

To address this, we have added a dedicated paragraph in the **Discussion** section of our revised manuscript. This new section not only clarifies our approach but also discusses its implications, thereby strengthening the overall narrative of our study. The new text can be found on lines 477-485 of the revised manuscript:

"Similarly, methane (CH_4) concentration in our simulations adopted the CAMx model's default setting of 1.85 ppm. This simplification aims to focus on the direct impacts of regional precursor controls. CH_4 is reported to influence global O_3 background levels[36]. If future global CH_4 concentrations rise as predicted[37], this will inevitably increase background O_3, adding additional pressure on O_3 attainment. This rising baseline reinforces the necessity of leveraging the AVOC 'bonus window' for maximum abatement efficacy. While our simplification facilitates a clear assessment of regional strategies, future work could integrate coupled Earth System Models to explore the synergy between global CH_4 evolution and regional O_3 policies"

In this new section, we explicitly state that CH_4 was held constant at the model's default value. We explain that this simplification was a deliberate choice to isolate and clearly quantify the effects of regional NO_x and AVOC control strategies, which is the core focus of our study. Furthermore, we discuss the critical implication of this choice: a potential rise in global CH_4 concentrations would increase background O_3 levels, which does not weaken our conclusion but rather reinforces the strategic urgency and value of capitalizing on the 'AVOC reduction bonus window' we have identified.

We are confident that this addition not only clarifies our methodology but also enriches the discussion by contextualizing our regional findings within the broader picture of global atmospheric change. We thank the reviewer for prompting this valuable improvement to our manuscript.

6. Model validation for key VOCs is essential when analyzing NO_x–VOC–O_3 chemistry. In the Supplementary Information, you state that "The OBM simulations were constrained by hourly observational data for key atmospheric constituents (O_3, NOx, CO, VOCs)." If these observational data (e.g., VOC and CO) are available, why are they not used to validate the model performance statistics and included in Supplementary Table 10?

Reply: We thank the reviewer for this insightful comment and for highlighting the importance of VOC model validation. The reviewer is correct in noting that we possess hourly VOC observation data from several urban sites, which were used to constrain the Observation-Based Model (OBM).

The primary reason we did not include a direct statistical validation of WRF-CAMx-simulated VOCs in original Supplementary Table 10 (revised Supplementary Table 14) is due to the fundamental mismatch in spatial representativeness between the point-based observations and the regional model grid. Specifically, the available hourly VOC data are from a limited number of urban core monitoring sites (seven cities, as detailed in Supplementary Text 6). These point measurements capture highly localized emission characteristics and may not be representative of the average conditions across a 36 km × 36 km model grid cell, which encompasses a complex mix of urban, suburban, and sometimes rural land uses. A direct comparison could therefore lead to misleading conclusions about the model's regional performance due to the high spatial heterogeneity of VOC sources.

However, we fully agree with the reviewer that this is an important point that warrants clarification. To enhance the manuscript's transparency and rigor, we have added a dedicated paragraph in the WRF-CAMx performance evaluation section of the Supplementary Information to explicitly discuss this limitation.

This addition can be found in the revised manuscript's Supplementary Information, Text 5, Lines 401-412:

"Furthermore, while hourly observational data for key VOC species were available for several urban sites and used to constrain the OBM, a direct statistical validation of WRF-CAMx-simulated VOCs was not included in Supplementary Table 14. This is primarily due to the mismatch in spatial representativeness: the

point-based urban measurements may not accurately reflect the average concentrations across a 36 km × 36 km model grid cell, which integrates a heterogeneous mix of emission sources. Nevertheless, the model's robust performance in simulating O_3 and NO_2, which are intrinsically linked to VOC chemistry, provides indirect confidence in the overall representation of the regional photochemical environment. This approach is consistent with prior regional modeling studies where comprehensive VOC validation is often limited by data availability and representativeness.”

We believe this clarification and addition, prompted by the reviewer's valuable suggestion, adequately address this concern and strengthen our manuscript.

Minor comments:

7. Lines 61-63 “This outcome contrasts sharply…”: The statement is quite strong and could be softened or better supported with references. For example, studies in Europe have shown mixed O_3 responses to NOx-targeted controls across different regions.

Reply: We thank the reviewer for this insightful comment. We agree that the initial statement on lines 61-63 was quite strong and could benefit from a more nuanced phrasing, particularly regarding the complexity of O_3 responses in Europe.

Our original references (Refs. 8, 9) primarily support the effectiveness of NO*x*-targeted controls in the United States, and Ref. 3 provides a broader context of O_3-climate control. We acknowledge that O_3 responses to NO*x* reductions in Europe have indeed exhibited regional variability and complex dynamics in certain areas. To address this, we have revised the sentence to soften the "contrasts sharply" phrasing without altering the role of this sentence.

Specifically, we have modified lines 61-63 in the manuscript (Introduction section) as follows:

Original Text (Lines 61-63):

"This outcome contrasts sharply with experiences in the United States and Europe, where NO*x*-targeted controls have proven effective for O_3 mitigation[3,8,9]."

Revised Text (Lines 67-68):

"This outcome contrasts with the general experiences in the United States, where NO*x*-targeted controls have proven effective for O_3 mitigation[3,8,9]"

We hope this revision now provides a more precise perspective, addressing the reviewer's suggestion.

8. Lines 77-79 "This inconsistency…": This sentence appears to repeat the idea from the previous lines and could be removed to improve conciseness.

Reply: We thank the reviewer for this valuable suggestion to improve the conciseness and clarity of our manuscript. We agree that eliminating redundancy is crucial for a strong narrative.

While the two related sentences served distinct and progressive logical roles, they can indeed be more concisely integrated. In the original text, the first sentence (lines 73-76) stated the core problem: the "inconsistent conclusions" present in the literature. The subsequent sentence (lines 76-79) was intended to dissect the deeper reason for this issue, by distinguishing superficial methodological limitations from the more 'fundamental', inherent flaws of the traditional "sensitivity zoning" approach. The newly revised version now merges these two points into a single sentence.

Original Text（lines 73-76）：

"However, its application in China has been fraught with inconsistent conclusions about summer regional O_3 sensitivity, irrespective of its basis in ground observations, satellite remote sensing, or model simulations (Supplementary Table 1)[13-18]. This inconsistency arises partly from the limitations of different methodologies and data sources[12,16], but more fundamentally, an often overlooked issue persists with traditional "sensitivity zoning" (Supplementary Text 1)."

Revised Text（Lines 79-83）：

"However, its application in China has yielded inconsistent conclusions of summer regional O_3 sensitivity across ground, satellite, and model-based studies (Supplementary Table 1)[13-18], a discrepancy stemming not only from methodological limitations[12,16], but, more fundamentally, from an inherent flaw in the traditional 'sensitivity zoning' (Supplementary Text 1)."

9. Figure 1: too big (it might be better to split into different figures). Also, in Fig 1c the x-axis does not align between the three regions, which makes the figure confusing. Consider plotting all three regions in the same panel (i.e. sharing the same x-axis) for easier comparison. Additionally, why the PRD region shows earlier trends (increase starting in 2008 versus 2011 for NCP and YRD) and also an earlier decrease?

Reply: We are grateful for the reviewer's valuable suggestions regarding figure presentation.

(1) For Fig. 1c, we fully concur with the reviewer's point that plotting trends on a shared x-axis will significantly enhance inter-regional comparability. In the revised manuscript, we have accordingly modified Fig. 1c, consolidating the FNR trends of the three key regions into a single, unified panel. This revised visualization more clearly elucidates the asynchronicity of the first trend turning point, thereby highlighting the O_3 photochemical system's capacity to promptly capture dynamic response signals stemming from divergent control pathways. We are grateful for this excellent suggestion, which has significantly improved the clarity of our presentation.

(2) Regarding the overall dimensions of Fig. 1, we wish to elaborate on our rationale for maintaining its integrated structure. Figure 1 serves as a pivotal piece of evidence in our study; it was strategically designed to simultaneously present the coupled dynamic responses of two critical yet distinct metrics: namely, de-weathered O_3 concentrations (Fig. 1a) and O_3 sensitivity trends (Fig. 1b-c). We contend that presenting these interconnected trends within a single, comprehensive figure is essential for intuitively conveying the 'synchronous plateau' – a central phenomenon of our study – to the readership. Disaggregating it into separate figures could potentially disrupt this crucial narrative and obscure the direct visual linkage between the evolution of O_3 levels and its underlying photochemical sensitivity. We hope the reviewer will appreciate our consideration in this matter.

(3) Regarding the earlier trend shift observed in the PRD region: We appreciate the reviewer prompting us to clarify this crucial point, which delves into the fundamental regional drivers underpinning our observed trends. In our revised manuscript, we have incorporated a dedicated discussion (Main Text, lines 143-154) to explicitly elucidate the reasons for the asynchronous evolution of O_3 sensitivity in the PRD region. The primary cause of this regional disparity lies in the asynchronous

implementation of precursor control policies across the various megacity clusters. As elaborated in the newly added text and corroborated by the emission inventory presented in Fig. 3, policy-driven emission trajectories exhibited notable divergences:

(a) Earlier NO*x* Reductions in PRD: Robust NOx abatement measures in the PRD region resulted in a discernible decline in summer NO*x* emissions commencing after 2008.

(b) Later NO*x* Reductions in NCP and YRD: In contrast, the NCP and YRD regions experienced substantial reductions in NO*x* emissions only after 2011, coinciding with the implementation of national-level policies of the '12th Five-Year Plan'.

(c) Consistent AVOCs Trends: Concurrently, AVOCs emissions across all three megacity clusters demonstrated sustained growth until approximately 2017.

Consequently, the earlier and more pronounced NO*x* reduction effectiveness in the PRD region served as the primary driver for its O_3 photochemical system's earlier transition towards a NO*x*-limited regime, thereby precipitating the earlier appearance of the first turning point in its FNR trend. We believe this clarification substantially enhances our analysis of regional heterogeneity and path dependency.

Specific revisions are provided below:

Added text (Lines 143-154):

"The PRD's O_3 sensitivity trend change point occurred earlier than in the other two megacity clusters. We primarily attribute this to the more synchronized implementation of precursor control policies in the geographically adjacent NCP and YRD, which led to a more consistent evolution of their O_3 sensitivity trends. This explanation is corroborated by the following presented regional emission inventories (Fig.3). Specifically, the PRD experienced a significant decline in summer NO_2 emissions post-2008, whereas a similar declining trend in the other two regions emerged after 2011. In contrast, AVOCs emissions generally increased across all regions until 2017. Therefore, the pronounced post-2008 reduction in NO_2 emissions was the principal driver for the turning point observed in the PRD's O_3 sensitivity trend around 2007. "

10. Line 259: In the text you are refering to "Methods", but then the section is named "Online methods".

Reply: We thank for this keen observation regarding the inconsistency in the naming of the methods section. We agree with the reviewer and have corrected this oversight. Throughout the revised manuscript, the section is now consistently referred to and titled as ‘Methods’. This standardization enhances the manuscript's structural clarity and consistency. The main section heading has been updated accordingly in the revised manuscript (Line 554 in the clean version).

11. Do you miss the y-axis in Figure 5? Please give more information in caption, this figure is the main figure to describe this study.

Reply: The original Fig. 5 was revised as following:

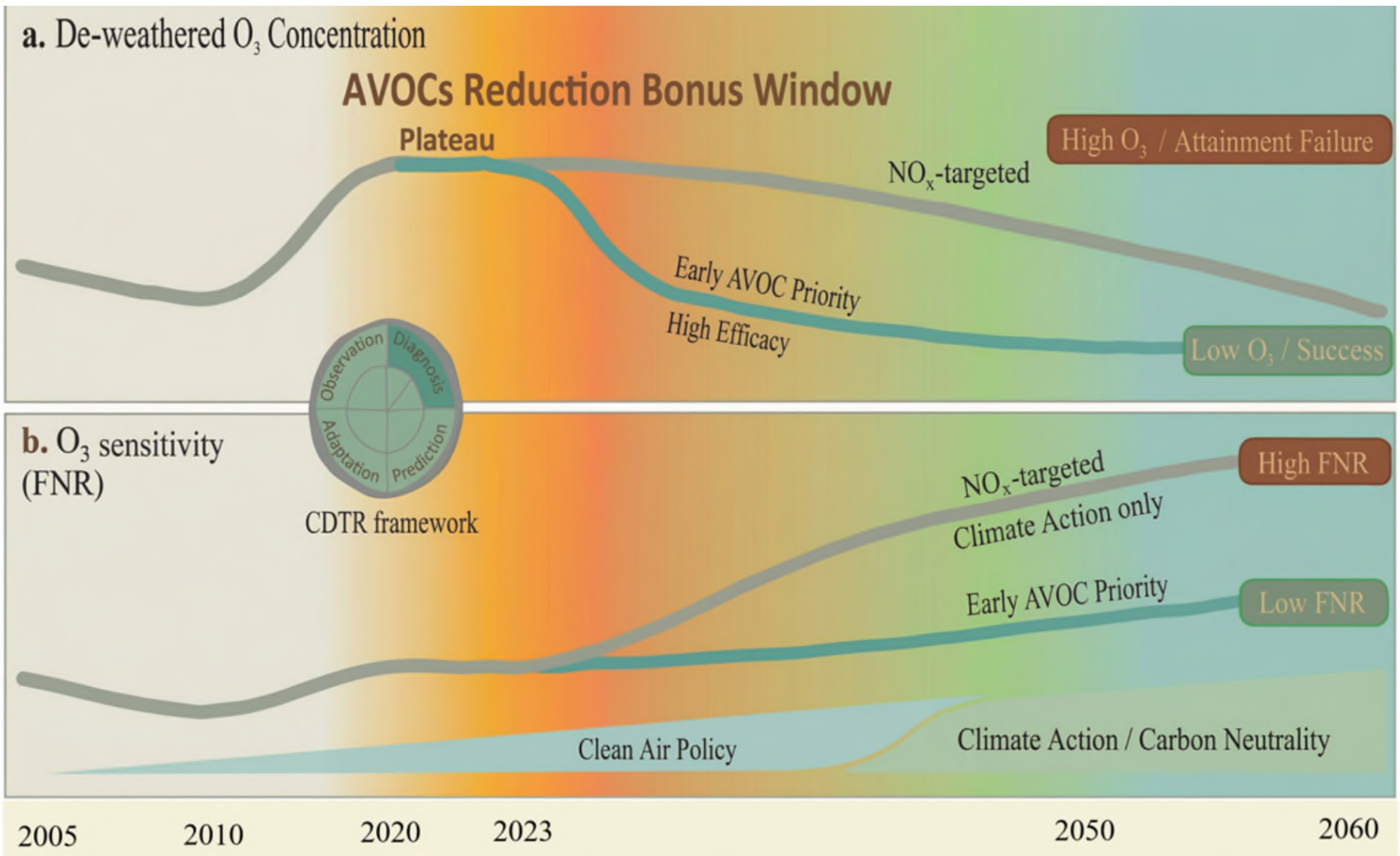

Fig. 6. Schematic of the optimal O_3 control strategy oriented towards synergistic climate and air quality goals grounded in historical field observations.

In the supplement:

12. Lines 221-226: Could you provide additional details on the methodology used to determine the threshold?

Reply: We thank the reviewer for this constructive comment, which has prompted us to elaborate further on the determination of the O_3 formation sensitivity thresholds. To address this, we have now expanded the relevant section to provide a detailed, step-by-step description of the established and validated model-based and observation-driven statistical methods used to define these thresholds.

We have revised the corresponding section in Supplementary Text 4. The updated text now reads as follows (lines 249-275 of the revised Supplementary Information):

"Two methodologies exist for determining these thresholds: (1) derivation from chemical transport models (CTM) that explicitly link $HCHO/NO_2$ ratios to surface O_3 sensitivity[3, 4, 20, 21], and (2) empirical derivation from concurrent ground-based observations using non-linear statistical models [7,9,10]. The first approach[3,4], utilize a CTM as a "virtual laboratory." First, driven by emission inventories and meteorological fields, the CTM simulates the dynamic evolution of the O_3-NO*x*-VOC photochemical system in a three-dimensional domain. Second, through specific numerical techniques, the response of O_3 concentrations to precursor changes was quantified within the model environment to identify the transitional state between NO*x*-limited and VOC-limited regimes. Finally, the FNR value simulated by the model at this specific transitional state is extracted and defined as the sensitivity threshold. Therefore, the fundamental commonality of these methods is their intrinsic dependence on the model's representation of chemical mechanisms, the accuracy and completeness of emission inventories, and the parameterization of atmospheric physical processes. The reliability of the derived threshold is entirely contingent on the model's fidelity in reproducing the complex, non-linear relationships of atmospheric chemistry.The latter approach[7,9,10], involves calculating the probability of surface O_3 exceeding a certain concentration threshold (e.g., 70 ppbv) for given satellite-derived FNR values. This is achieved by examining their statistical relationship, typically using a third-order polynomial model. The peak of this polynomial curve is assumed to mark the transition point between VOC-limited and NO*x*-limited O_3 formation regimes, and the transitional regime is subsequently defined as the range of FNR values spanning the top 10% of this high-O_3 probability distribution. To align with satellite overpass times, ground-based O_3 observations from 13:00 to 14:00 local time were selected, after eliminating invalid daily O_3

monitoring data. The FNR thresholds employed in this study were derived using this observation-based statistical method, as detailed in our prior publications[9,10]."

We are confident that these revisions now provide sufficient detail within the Supplementary Text 4, while also appropriately guiding interested readers to the more complete methodological descriptions in the cited literature.

13. Supplementary Figs 4 and 5: could you use the same range in the y-axis?

Reply: Revised as suggested. The current figure numbers are Supplementary Fig. 16 and 17.

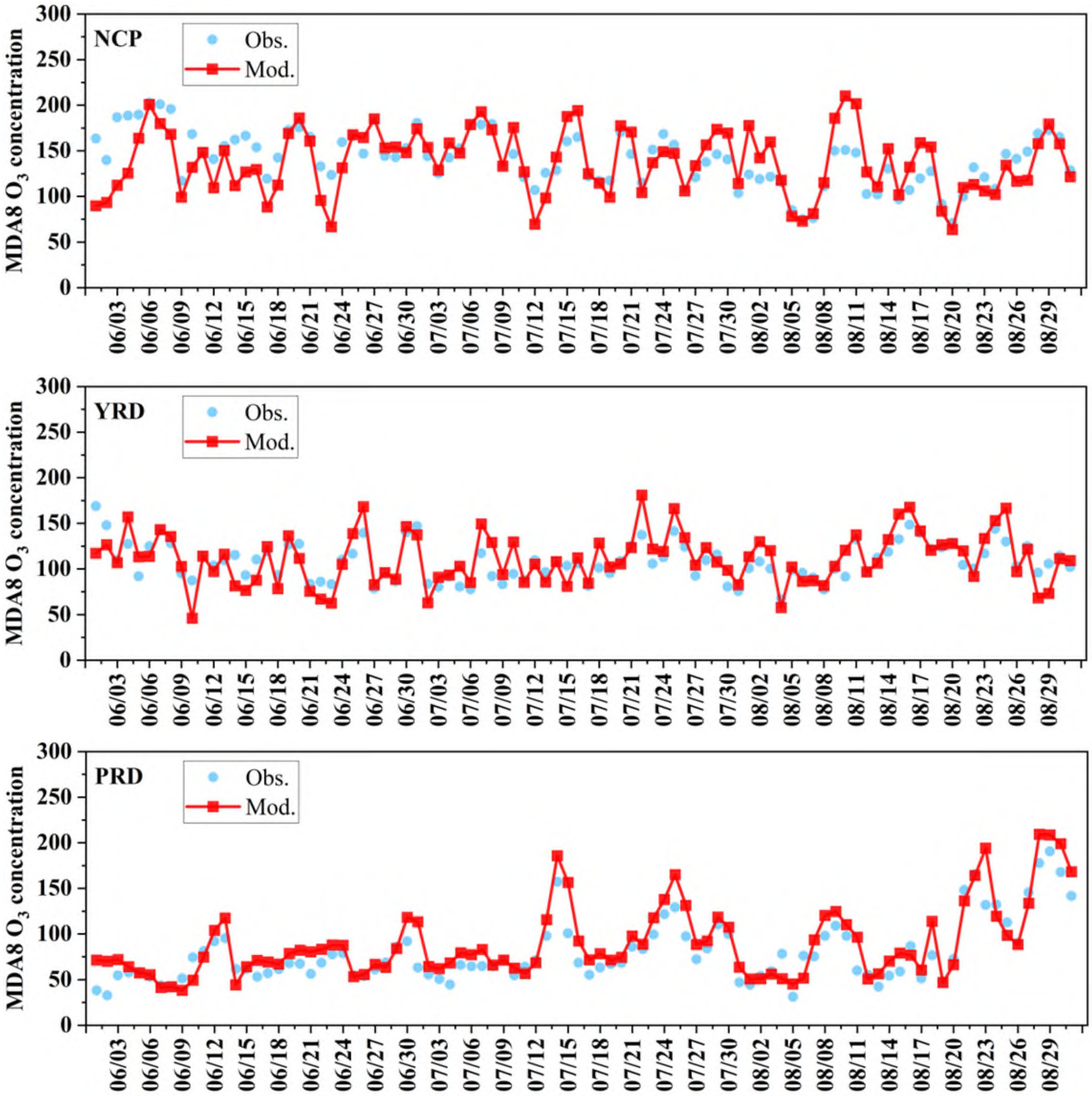

Supplementary Fig. 16. Comparison of monitored and simulated daily MDA8 O_3 concentration during the summer of 2020.

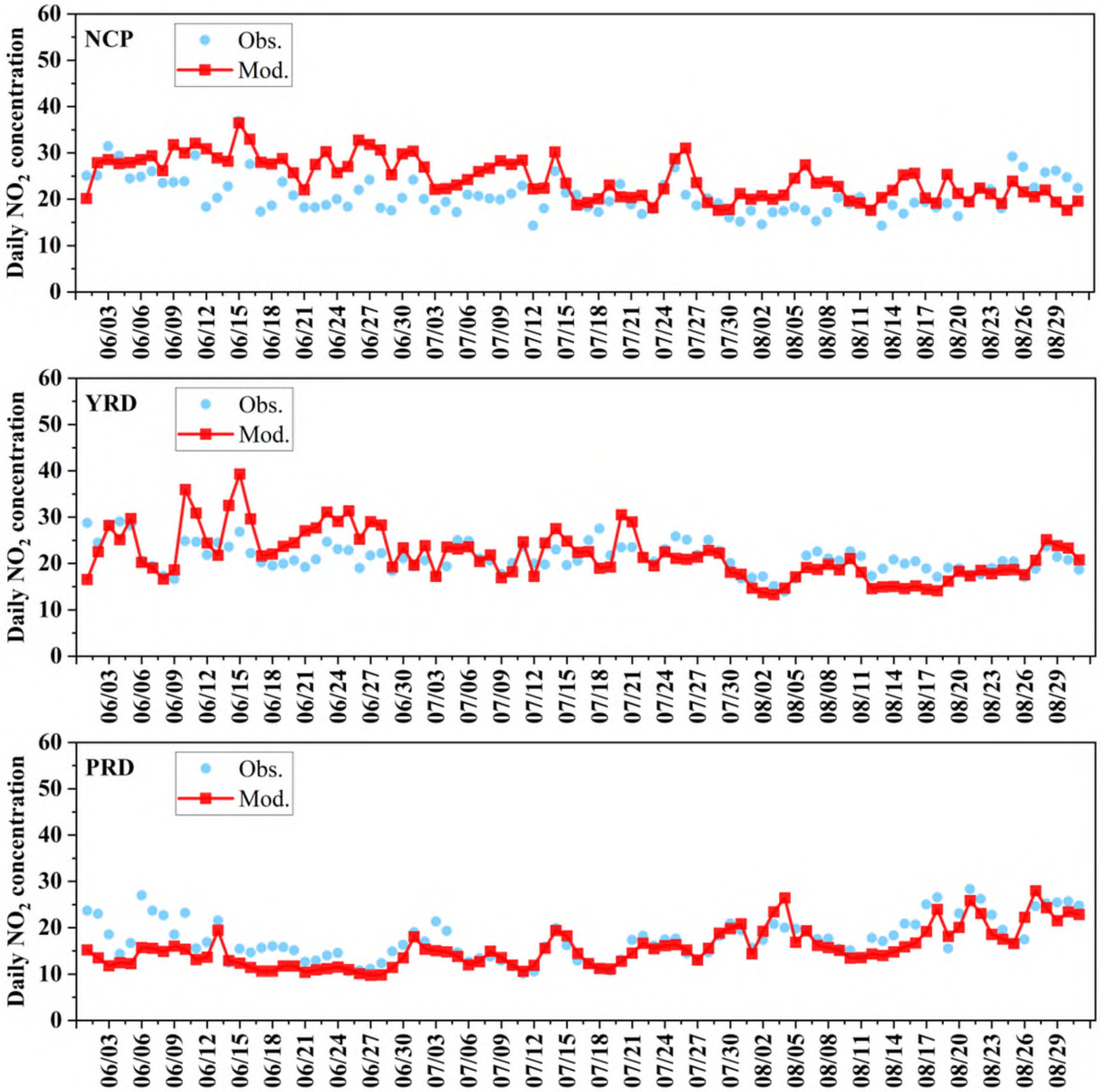

Supplementary Fig. 17. Comparison of monitored and simulated daily NO_2 concentration during the summer of 2020.

14. Line 302 "Emission data for 2021 were subsequently refined by incorporating the latest local measurements". Could you elaborate on that?

Reply: We appreciate the reviewer's attention to the details of the 2021 emission data. As the official MEICv1.4 inventory was only updated to 2020 at the time of this study (The aggregate emissions of AVOCs and NO_x for the 2021–2023 period, as used in Fig. 3c, were provided by the MEIC team at Tsinghua University. However, the corresponding spatially and sectorally resolved inventories for this period were not available for this study), we constructed the 2021 emission inventory using a robust and widely-accepted scaling-factor approach. The detailed process involved:

(1) Baseline Inventory: We utilized the MEIC2020 inventory, which possesses fine spatial and sectoral resolution, as our foundation. This inventory provides a detailed spatial distribution of emissions.

(2) Scaling Data: We collected the latest available official provincial total emission data for key pollutants (e.g., NO_x, VOCs) for 2021.

(3) Refinement Procedure: For each province and each precursor, we calculated a province-specific scaling factor by dividing the 2021 provincial aggregate emissions by the corresponding 2020 provincial total, which was aggregated from the gridded MEIC 2020 data. This scaling factor was then applied to every grid cell within that respective province in the MEIC 2020 inventory.

(4) Rationale: This approach effectively updates the emission magnitudes to reflect actual activity levels in 2021 while preserving the validated and finely resolved spatial allocation pattern of the MEIC inventory. In the transitional period before new official high-resolution inventories are formally released, this constitutes a standard and scientifically reasonable method for emission estimation.

To provide this methodological detail, we have incorporated this elaboration into the Supplementary Information, specifically in Supplementary Text 5: WRF-CAMx Model Configuration and Performance Evaluation (lines 351-363)：

"......Emissions outside of China were derived from the Regional Emission Inventory in ASia version 2.1 (REAS v2.1) (original resolution 0.25° × 0.25°)[25]. For 2021, as the official MEIC v1.4 inventory was updated only up to 2020 at the time of this study, we refined the emission data to reflect the activity levels of that year. This involved using the MEIC 2020 inventory as a spatial distribution baseline and

collecting provincial-level total atmospheric pollutant emission data for 2021. We then calculated the provincial total change ratios for each major precursor (2021 vs. 2020) based on these latest statistics. These ratios served as scaling factors, which were applied to adjust the grid-level emissions of the corresponding provinces within the MEIC 2020 inventory. This approach updated the total emissions to levels more representative of 2021 while preserving the validated fine spatial distribution of the MEIC inventory. This method assumes relatively stable internal spatial emission distributions within each province over a single year, a common and reasonable approximation when high-resolution, updated inventories are unavailable. These inventories were converted to the LCC projection coordinate system using geographic distribution factors......"

15. Lines 350-353: "Daily average simulated temperature and atmospheric pressure showed high consistency with observations, with r values consistently above 0.75 and 0.97, respectively" and then in Supplementary Table 11, NCP correlation for the temperature is 0.57. Could you check those statistics, please?

Reply: We are grateful to the reviewer for the meticulous scrutiny of our reported statistics and for highlighting this potential inconsistency. We have thoroughly re-examined both the textual statement (Supplementary Information, lines 350-353) and the corresponding values in original Supplementary Table 11. We confirm that the Pearson's correlation coefficient r for simulated temperature in NCP, as presented in the original Supplementary Table 11 (row 'Temperature', column 'R'), is 0.82. The statement in the text (original Supplementary Information, lines 350-351) "Daily average simulated temperature... showed high consistency with observations, with r values consistently above 0.75..." accurately reflects this value and other regional temperature correlations (YRD: 0.89; PRD: 0.76), all of which are indeed above 0.75. We believe the reviewer may have inadvertently referred to the 'Wind speed' row for NCP in the original Supplementary Table 11, where the r value is 0.57.

To further enhance the clarity and precision of our model evaluation section, we have revised the relevant sentences in the manuscript. Instead of using a vague lower bound, we now provide specific ranges for the correlation coefficients of each key

meteorological variable, consistent with the data in original Supplementary Table 11 (revised Supplementary Table 14).

Specifically, we have revised the description in the Supplementary Information, Text 5, Model Performance Evaluation section (revised Supplementary Information lines 419-427) to read:

"...The WRF model reasonably reproduces the spatiotemporal variations of these meteorological variables within the study regions. Daily average simulated temperature and atmospheric pressure exhibit high consistency with observations, with Pearson's correlation coefficients (r) ranging from 0.76 to 0.89 and 0.97 to 0.99 for the three regions, respectively, although NMB indicated a slight underestimation. Simulated relative humidity also shows good consistency ($r \approx 0.66–0.88$, average MB $\approx -3.75\%$). Although wind speed correlations are slightly lower ($r \approx 0.57–0.60$), the overall robust simulation performance provides reliable meteorological field inputs for the chemical transport model."

We hope that this more precise and transparent description now accurately reflects our model's performance, fully addresses the reviewer's valid concern, and enhances the rigor of the manuscript.

Response to Reviewer #5

This study offers a fresh and highly significant perspective on controlling ozone (O_3) pollution in China, with both crucial scientific and practical implications. By introducing the Coupled Dynamic Trend Response (CDTR) analytical method, the authors successfully overcome the limitations of traditional static sensitivity analysis. I believe this manuscript is innovative, its findings are highly significant, and it is well-suited for publication in Nature Communications. However, to further strengthen its scientific rigor and persuasive power, I have several technical questions and suggestions for the authors to consider during the revision process.

Reply: We are deeply grateful for the thorough review and encouraging assessment. We are especially gratified that the reviewer recognized the innovativeness of our proposed Coupled Dynamic Trend Response (CDTR)

analytical method, its success in overcoming the limitations of static analysis, and the overall scientific and practical significance of our findings. We are also profoundly grateful for the reviewer's subsequent valuable suggestions aimed at further enhancing the scientific rigor of our manuscript. We have carefully considered all comments point-by-point and have provided detailed responses below. The manuscript has also been revised accordingly in response to these valuable comments.

Specific Questions

1. What do the shaded areas in Fig. 1b represent? Please clearly label this in the figure caption or legend.

Reply: Labeled as suggested.

Specific modifications are as follows (Lines171-173):

"**b,** Interannual variation of summer satellite-based FNR ($HCHO/NO_2$) in the three megacity clusters of China during 2005-2024. The shaded area highlights the duration of the plateau period."

2. The main text mentions using TROPOMI data instead of OMI data. Please explain the rationale behind this choice. If OMI data were used as the FNR indicator, given its longer historical record, how would the trend change? Please provide a supplementary analysis or explanation.

Reply: We sincerely thank the reviewer for this constructive comment, which highlights the critical importance of ensuring data continuity and robustness in long-term trend analysis.

(1) Rationale for transitioning from OMI to TROPOMI data:

our decision to use TROPOMI data from 2018 onwards was a deliberate methodological choice driven by critical considerations of data quality and analytical fidelity. The OMI instrument, after more than a decade in orbit, has experienced sensor degradation. This aging is particularly problematic for the retrieval of trace gases with weak optical absorption, such as HCHO. In recent years (especially

post-2020), this has manifested as severe striping noise in the OMI HCHO product, critically compromising its reliability for the precise trend analysis our study requires.

To provide the reviewer and readers with direct, visual evidence of this issue, we have included a key figure in the revised Supplementary Information (Supplementary Fig. 5). This figure explicitly compares OMI HCHO retrievals from 2020 and 2022 against the much cleaner retrievals from TROPOMI for 2022. This issue is also documented in the literature (Vazquez Santiago et al., 2024, Nat. Commun.).

Given that one of the cornerstones of our paper—the 2020–2023 'synchronous plateau'—serves as key empirical evidence for 'path dependency,' ensuring the highest data fidelity during this recent period was paramount. The superior signal-to-noise ratio and spatial resolution of TROPOMI were therefore essential for reliably resolving the subtle dynamics of precursor concentrations during this critical phase.

To ensure this methodological rationale is fully transparent, we have now explicitly stated this justification in the main text of the revised manuscript and in the caption for Fig. 1 (main text, lines 154-158; Supplementary Fig. 5 and Supplementary Text 4).

The specific revision are as follows:

Revised main text (Lines 154-158):

"Owing to the well-documented issue of the OMI satellite sensors aging[23], characterized by pronounced striping noise in HCHO retrievals (Supplementary Fig. 5), TROPOMI data have been adopted for the period since 2018, and the FNR trends derived from TROPOMI and OMI were approximately consistent (Fig. 1 and Supplementary Tables 2, 4, 6).

23. Vazquez Santiago, J., Hata, H., Martinez-Noriega, E.J. et al. Ozone trends and their sensitivity in global megacities under the warming climate. *Nat Commun* 15, 10236 (2024). "

Revised Supplementary Text 4 (Lines 227-240):

"The transition from OMI to TROPOMI data in 2018 is a deliberate and critical methodological choice driven by stringent data quality control. While the OMI instrument has provided an invaluable long-term record, its performance has degraded after more than a decade of operation. The retrieval of trace gases with weak optical absorption, such as HCHO, necessitates an exceptionally high signal-to-noise ratio. In recent years, particularly post-2020, detector aging has manifested as severe striping

noise in OMI HCHO products (Supplementary Fig. 2), which critically compromises the usability of the data for precise trend analysis. Consequently, we transitioned to TROPOMI data for the period of 2018–2024. As the successor to OMI, TROPOMI features significantly improved signal-to-noise ratios and finer spatial resolution (7 × 7 km^2 versus 13 × 24 km^2 for OMI). Therefore, to ensure the highest possible accuracy and reliability for our analysis, particularly for resolving the fine-scale dynamics of O_3 sensitivity after 2018, we exclusively use the TROPOMI dataset for the recent period.

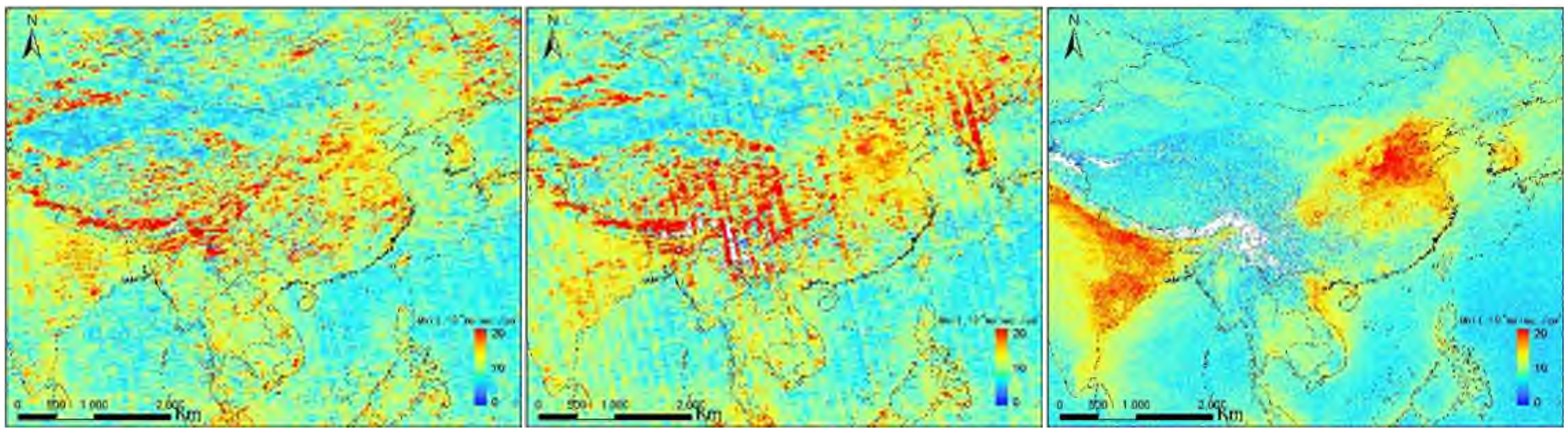

OMI HCHO 202006 | OMI HCHO 202206 | TROPOMI HCHO 202206

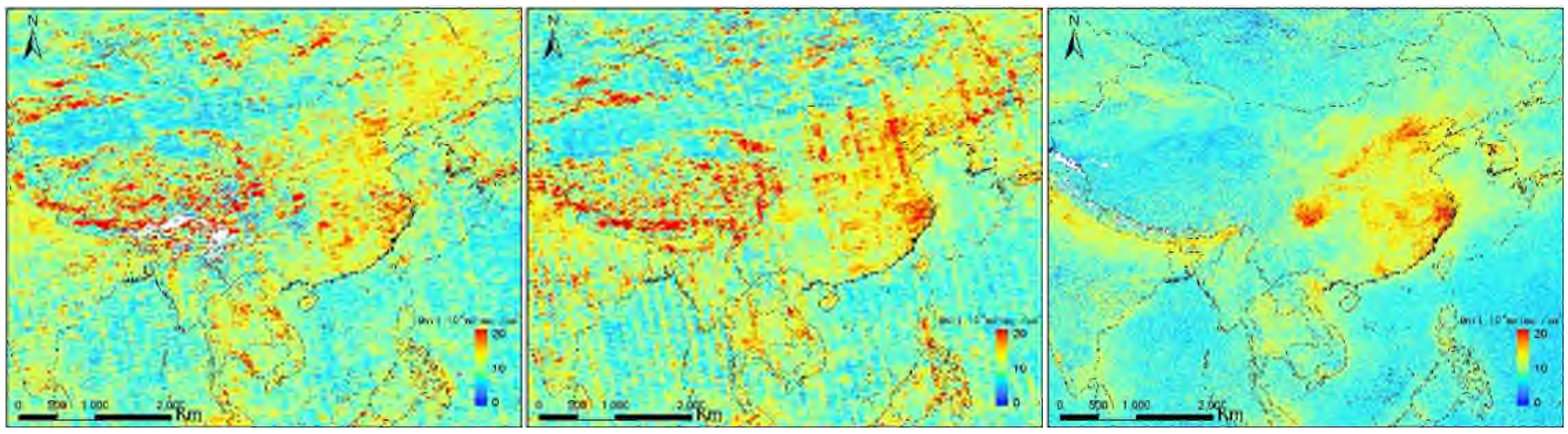

OMI HCHO 202007 | OMI HCHO 202207 | TROPOMI HCHO 202207

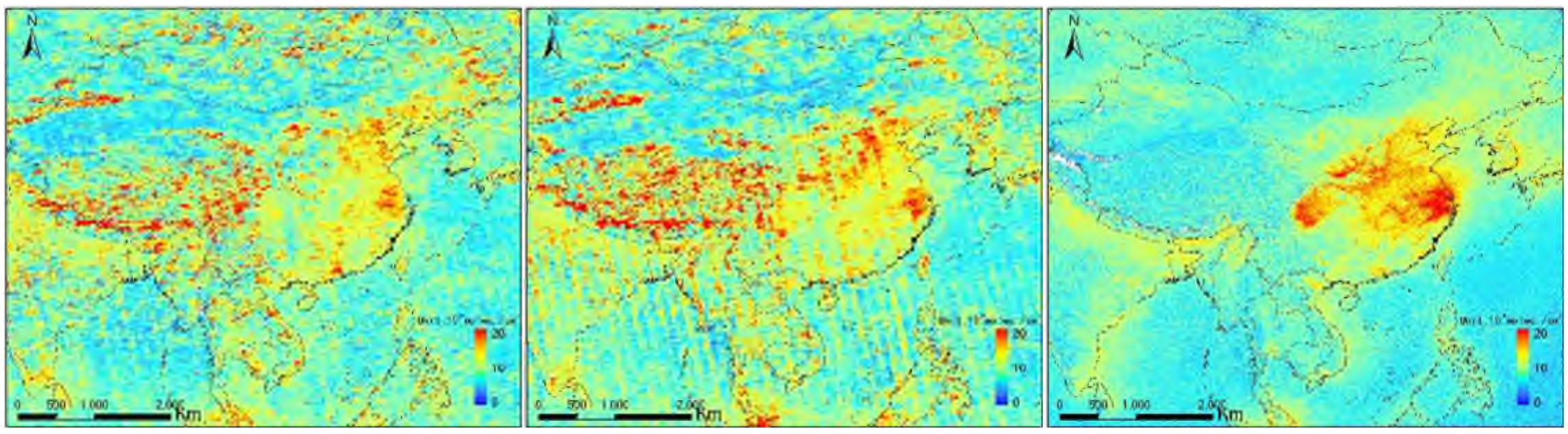

OMI HCHO 202008 | OMI HCHO 202208 | TROPOMI HCHO 202208

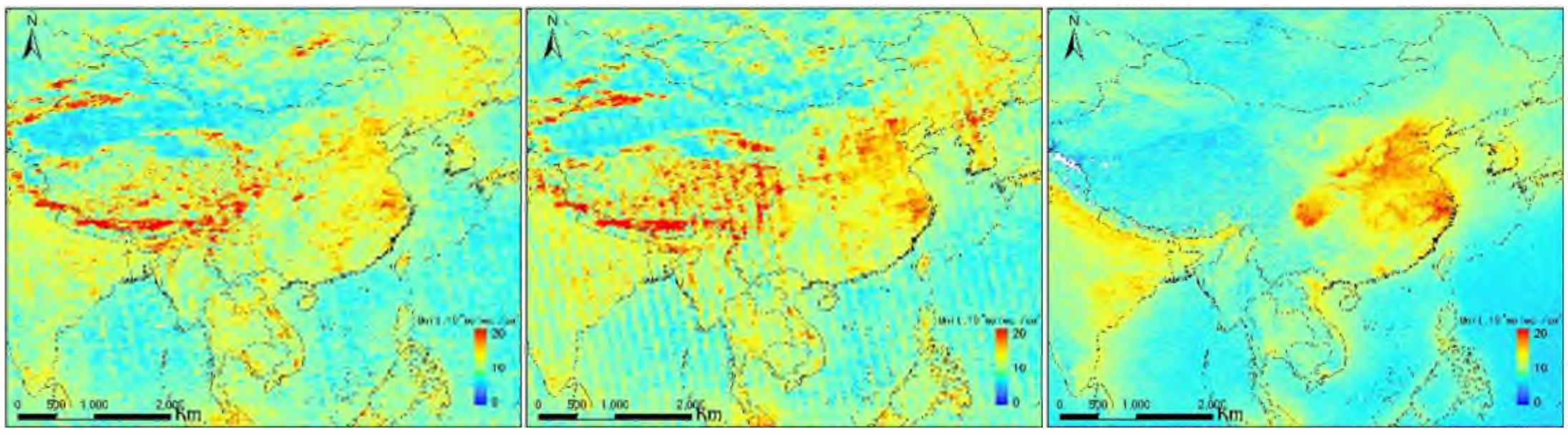

OMI HCHO 202006-08 Mean OMI HCHO 202206-08 Mean TROPOMI HCHO 202206-08 Mean

Supplementary Fig. 5. Visual comparison of OMI and TROPOMI HCHO column densities illustrating OMI sensor aging. Monthly mean HCHO column densities for June, July, and August, and the summer mean for 2020 and 2022. Distinct striping noise is evident in the OMI data from 2022 (middle column) compared to the cleaner retrievals from OMI in 2020 (left column) and TROPOMI in 2022 (right column)."

(2) Analysis of FNR trend consistency between OMI and TROPOMI:

Both our analysis and publications confirm that while absolute FNR values and the area fractions of sensitivity regimes may differ between the two sensors (owing to differences in resolution, retrieval algorithms, etc.), the crucial long-term evolutionary trends are highly consistent.

In the main text (Lines 156-158), we now directly state this conclusion:

"...and the FNR trends derived from TROPOMI and OMI were approximately consistent (Fig. 1 and Supplementary Tables 2, 4, 6)."

We substantiate this consistency through several evidence:

In Supplementary Text 4 ("The robustness of sensitivity trends investigation"), we provide a detailed discussion on the robustness of our trend analysis. We first establish, based on prior literature, that trend-based analyses are inherently robust against satellite product changes:

Supplementary Text 4, Lines 293-301:

"Crucially, the trend in FNR is less sensitive to the choice of satellite product, as long-term multi-year averaging attenuates inter-product differences, and different products generally exhibit high consistency in their long-term spatiotemporal variations[3,23]. Similarly, while areal fractions may differ, the trend in the areal fraction of a specific sensitivity regime within a given region and season is expected to be robust to the choice of threshold. This premise is supported by literature showing

similar evolutionary patterns of regime shifts between years despite the use of different thresholds derived from various satellite products and criteria[23].

3. Du, X. et al. Modeling of spatial and temporal variations of ozone-NO*x*-VOC sensitivity based on photochemical indicators in China. *J. Environ. Sci.* **114**, 454 – 464 (2022)

23. Wang, Y. et al. Sustained emission reductions have restrained the ozone pollution over China. *Nat. Geosci.* **16**, 967 – 974 (2023)."

Furthermore, our own validation work confirms this robustness, specifically through a direct comparison of the interannual variations in sensitivity regime area fractions derived from both OMI and TROPOMI (Supplementary Tables 2, 4, 6):

Supplementary Text 4, Lines 302-311:

"We further validated this premise by: (1) comparing trends in regime area fractions derived from OMI and TROPOMI data (summers 2005–2024) using a uniform FNR threshold......While areal fractions differed across these comparisons, the trends in the variation of areal fractions remained consistent. For instance, the maximum areal fraction of the VOC-limited regime consistently coincided with the first FNR trend inflection point (e.g., ~2010 for NCP and YRD), and the maximum NO*x*-limited regime areal fraction aligned with the second FNR inflection point (e.g., ~2020)

Supplementary Table 2. Annual average area fractions of ozone formation regimes in NCP region during summers of 2005–2024, with uniform thresholds of transition regime of [2.3,4.2]

NCP-OMI	VOC-limited regime （%）	Transitional regime （%）	NO*x*- limited regime （%）
2005	20.09	49.88	30.02
2006	29.10	48.15	22.75
2007	26.21	46.65	27.14
2008	30.83	48.38	20.79
2009	29.79	47.69	22.52
2010	35.10	42.38	22.52
2011	33.26	49.08	17.67
2012	32.45	47.23	20.32
2013	23.56	45.38	31.06
2014	33.37	43.42	23.21
2015	19.63	59.47	20.90
2016	11.32	58.55	30.14
2017	9.01	61.09	29.91
2018	3.00	60.28	36.72

2019	2.89	60.39	36.72
2020	1.27	51.73	47.00

NCP-TROPOMI	VOC-limited regime（%）	Transitional regime（%）	NO*x*- limited regime（%）
2018	2.40	42.71	54.89
2019	1.25	28.91	69.84
2020	0.89	24.48	74.63
2021	1.51	42.13	56.36
2022	0.39	44.82	54.79
2023	0.16	53.12	46.67
2024	0.02	25.08	74.70

Supplementary Table 4. Annual average area fractions of ozone formation regimes in YRD during summers of 2005–2024, with uniform thresholds of transition regime of [2.3,4.2]

YRD-OMI	VOC-limited regime（%）	Transitional regime（%）	NO*x*- limited regime（%）
2005	12.92	43.54	43.54
2006	15.87	46.31	37.82
2007	16.97	45.39	37.64
2008	19.19	47.60	33.21
2009	16.79	45.39	37.82
2010	21.59	50.92	27.49
2011	19.00	48.71	32.29
2012	18.27	50.37	31.37
2013	8.86	40.96	50.18
2014	20.11	54.24	25.65
2015	12.92	54.06	33.03
2016	3.87	44.28	51.85
2017	7.56	50.00	42.44
2018	5.35	47.79	46.86
2019	5.17	50.92	43.91
2020	2.03	41.51	56.46

YRD-TROPOMI	VOC-limited regime（%）	Transitional regime（%）	NO*x*- limited regime（%）
2018	3.73	31.54	64.73
2019	3.48	21.21	75.31
2020	2.33	16.87	80.79
2021	6.03	35.90	58.07
2022	0.79	22.19	77.02
2023	1.94	37.87	60.13
2024	0.49	14.01	85.45

2022	0.27	17.34	82.40
2023	0.80	31.15	68.05

2024	0.21	10.98	88.76

Supplementary Table 6. Annual average area fractions of ozone formation regimes in PRD during summers of 2005–2024, with uniform thresholds of transition regime of [2.3,4.2]

PRD-OMI	VOC-limited regime (%)	Transitional regime (%)	NO*x*- limited regime (%)
2005	37.50	41.25	21.25
2006	37.50	33.75	28.75
2007	36.25	38.75	25.00
2008	40.00	38.75	21.25
2009	28.75	35.00	36.25
2010	40.00	37.50	22.50
2011	28.75	45.00	26.25
2012	26.25	45.00	28.75
2013	26.25	48.75	25.00
2014	26.25	43.75	30.00
2015	18.75	37.50	43.75
2016	17.50	42.50	40.00
2017	21.25	50.00	28.75
2018	1.25	41.25	57.50
2019	6.25	42.50	51.25
2020	8.75	41.25	50.00
**PRD-TROPOMI**	**VOC-limited regime (%)**	**Transitional regime (%)**	**NO*x*- limited regime (%)**
2018	8.48	28.63	62.89
2019	4.03	25.36	70.61
2020	4.79	27.88	67.34
2021	5.96	29.05	64.99
2022	4.95	31.49	63.56
2023	5.79	36.78	57.93
2024	3.02	29.14	68.36

”

In summary, we have addressed the reviewer's concern by providing a clear rationale for the data transition and by presenting a robust quantitative analysis that validates the consistency of the key sensitivity trends between OMI and TROPOMI.

4. How were the inflection points in Fig. 1c identified, particularly the start of the plateau phase around 2020? Please provide a detailed description of the

quantitative method used, such as time series analysis or a specific change point detection algorithm.

Reply: We deeply appreciate the reviewer for the constructive suggestion regarding the identification of the inflection point in Fig. 1c, particularly the onset of the plateau phase around 2020. We acknowledge that the combined satellite FNR time series (OMI: 2005–2020; TROPOMI: 2018–2024), despite its long span, presents specific challenges for the application of formal statistical change-point detection algorithms (e.g., optimal break-point identification to minimize the overall residual sum of squares). Specifically, the well-documented aging of the OMI sensor (Ref. 23, Ziemke et al., 2022), which led to increased noise in HCHO retrievals over time, introduces complexities for robust statistical detection in the later part of the OMI operational period. Furthermore, the limited data volume from the high-quality TROPOMI period (2018–2023 used in our analysis) constrains the robustness of such algorithms in independently identifying a new inflection point within this specific window.

Consequently, our approach was not based on a purely statistical change-point algorithm but rather on a convergent, multi-evidence framework. In the context of our study, the determination of the inflection point around 2020 is scientifically robust due to the following points of converging evidence:

(1)Policy-Driven Breakpoint: The primary anchor for the ~2020 inflection point is the significant shift in China's national air pollution control policy. The summer of 2020 marked the conclusion of the "Three-Year Action Plan" and the nationwide implementation of the "Action plan on VOCs reduction in the summer (2020)" (Ref. 27). This policy transition provided a strong, externally-defined a priori basis for examining a potential shift in the O_3 photochemical system, because the main subjects of our study is the path dependence of O_3 control strategies. The response of O_3 photochemical system (de-weathered O_3 concentration and sensitivity) to different clean air polices should be studied.

(2)Corroboration from Independent Literature: Our analysis builds upon recent pivotal studies that have already established a plateau in China's de-weathered O_3 concentrations beginning around 2020 (e.g., Wang et al., 2023, Ref. 13;). Our novel contribution was to investigate whether the O_3 sensitivity trend exhibited a concurrent

shift, thereby validating the 'coupled response' hypothesis. The synchronicity we observed provides validation for the selection of this breakpoint.

(3)Visual Confirmation and Segmented Analysis: With the ~2020 breakpoint established by policy and prior literature, the segmented linear fits shown in Fig. 1c were employed not to discover the inflection point, but to quantify and visualize the change in the FNR trend before and after this externally-defined transition. The distinct change in slope visually confirms the shift. We have also added an explanation in the revised manuscript for the earlier first inflection point in the PRD, attributing it to the earlier implementation of NO*x* control policies.

(4)Consistency of Trends from OMI and TROPOMI: It is noteworthy that the FNR trends from OMI and TROPOMI are fundamentally consistent during their overlapping period (2018–2020) (as shown in Fig. 1 and Supplementary Tables 2, 4, 6). The following statement has been added to the revised manuscript:

Revised manuscript (Lines 154-158)

" Owing to the well-documented issue of the OMI satellite sensors aging[23], characterized by pronounced striping noise in HCHO retrievals (Supplementary Fig. 5), TROPOMI data have been adopted for the period since 2018, and the FNR trends derived from TROPOMI and OMI were approximately consistent (Fig. 1 and Supplementary Tables 2, 4, 6)"

(5)Validation of the Sensitivity Trend with Multiple Independent Methods: To confirm that the stagnation in the trend of summer O_3 sensitivity towards a NO*x*-limited regime around 2020, as revealed by satellite FNR (Fig. 1), was not an artifact of a single metric, we conducted cross-validation using two widely-accepted, independent methods: CTM and OBM. The convergence of conclusions from these multiple methods further corroborates the occurrence of the sensitivity trend inflection point around 2020. This comprehensive analysis is now detailed in the revised manuscript as follows:

Revised manuscript (Lines 175-204):

"To ensure that the sensitivity trend plateau revealed by satellite FNR (Fig. 1) was not an artefact of a single metric, we further cross-validated the O_3 sensitivity trend from 2019–2021 using two widely applied independent methods (Fig. 2). Chemical transport model (CTM, the WRF-CAMx detailed in Supplementary Text 5) simulations revealed that NO*x*-limited areas peaked and that VOC-limited areas were

minimised in 2020 (NCP) or 2019 (PRD, YRD). Additionally, to complement the regional-scale satellite and model analyses, and to resolve the trends in O_3 sensitivity at the urban-core scale—a domain where satellite observation and CTM simulation have known limitations[15]—we employed an Observation-Based Model (OBM, detailed in Supplementary Text 6). Constrained by in-situ hourly measurements of VOCs and NO*x* (Supplementary Text 2), the model diagnoses sensitivity via relative incremental reactivity (RIR). This analysis (Fig. 2b) was conducted in seven cities, selected from the three megacity clusters (Supplementary Fig. 1), possessing robust data foundations, and representing diverse economic development levels in China. The inter-annual RIR trends corroborated this stagnation: the progression towards a NO*x*-limited regime stalled in 2020 for Beijing, Tianjin, Jinan, Xiong'an (NCP), and Guangzhou (PRD), and as early as 2019 for Shanghai (YRD). In the case of Zhengzhou, although its O_3 sensitivity continued to trend towards NO*x*-limited conditions during the 2019–2021 summers, it remained persistently within the VOC-limited regime, thus still necessitating the prioritization of AVOC controls. While differing in sensitivity regime classification (Supplementary Table 8, Supplementary Figs. 2-4 and Fig. 2a), which are the limitations of static sensitivity zoning methods, all three approaches (satellite FNR, WRF-CAMx, OBM) converged on the same sensitivity trend: the progression towards the NO*x*-limited regime stalled around 2020 in the three megacity clusters. This multi-method convergence strengthens the robustness of the observed sensitivity trend plateau phenomenon. The specific period (2019-2021) was selected to scientifically bracket the 2020 inflection point and was determined by the availability of comprehensive, ground-based speciated VOC measurements required for the OBM analysis, as well as the emission inventory data available for CTM simulations.

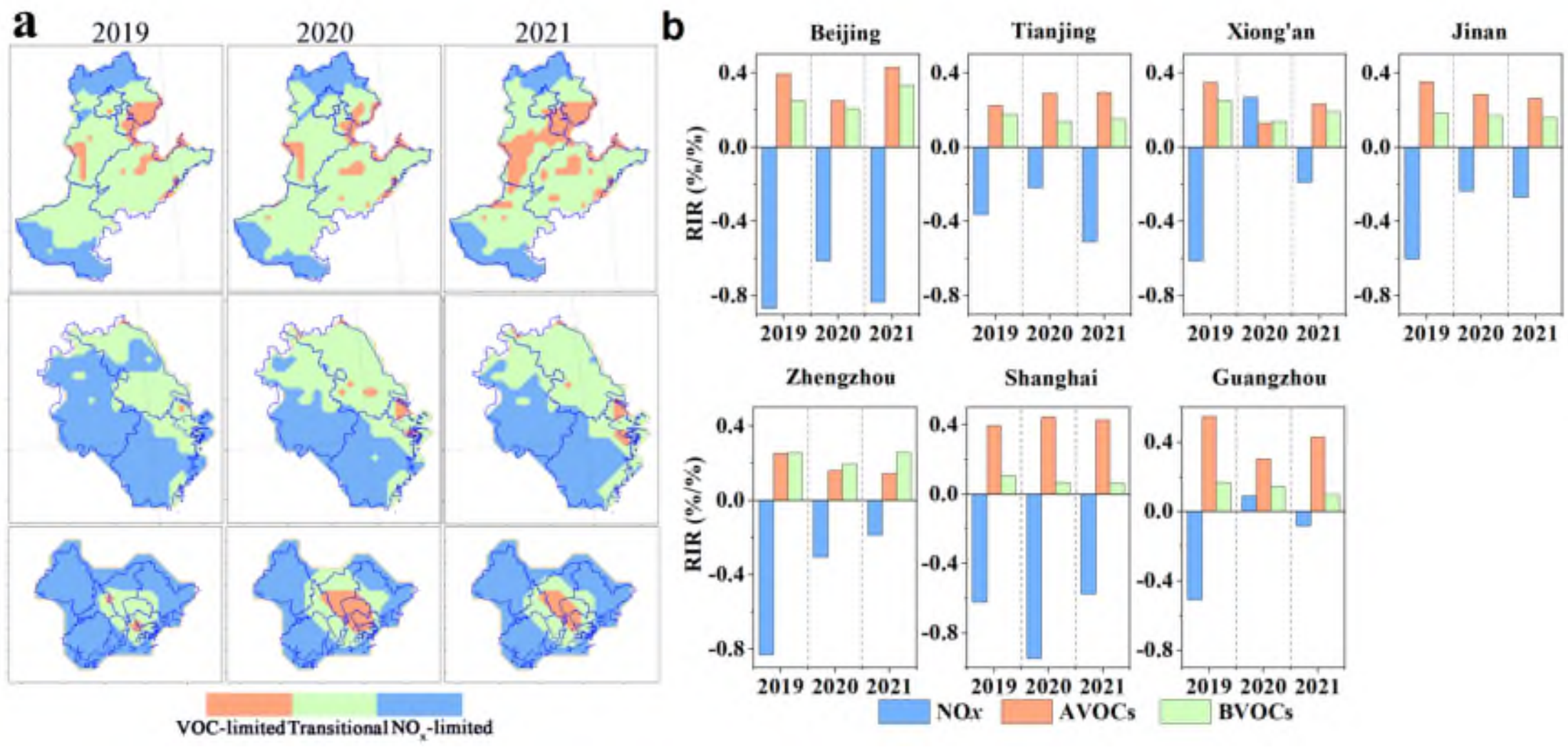

Fig. 2. Multi-source evidence confirms the robustness of the sensitivity trend stagnation . a, Maps illustrating O_3 formation regimes across the three megacity clusters (NCP, YRD and PRD from top to bottom) from 2019–2021 investigated by the WRF-CAMx model (the simulation resolution is 36km). **b,** relative increment reactivity (RIR) calculated by OBM model for O_3 precursors (specifically, NO*x*, AVOC, and Biogenic VOC (BVOC)) of typical cities during the summers of 2019-2021."

4. Liu, Y. et al. Drivers of increasing ozone during the two phases of clean air actions in China 2013–2020. *Environ. Sci. Technol.* **57**, 8954–8964 (2023).

23. Vazquez Santiago, J., Hata, H., Martinez-Noriega, E.J. et al. Ozone trends and their sensitivity in global megacities under the warming climate. *Nat Commun* **15**, 10236 (2024).

We hope this detailed explanation enhances the methodological transparency and rigor of our study.

5. Figure 2 is used to cross-validate the satellite data. Why were the years 2019, 2020, and 2021 specifically chosen for this validation, especially since Fig. 1 shows the plateau period extending beyond these years?

Reply: We are grateful to the reviewer for raising this important question regarding the selection of the specific time frame for our cross-validation analysis in Fig. 2. Our

choice of the 2019–2021 period was indeed a deliberate strategy jointly determined by a precise scientific objective and objective data constraints.

(1) Scientific Objective: Our primary scientific goal for the analysis in Fig. 2 was not to replicate the entire duration of the O_3 sensitivity plateau observed in Fig. 1. Instead, it was to rigorously test the most critical feature revealed by the long-term satellite record: the inflection point around 2020 that marks the onset of the stagnation period. The 2019–2021 time frame was the optimal choice for this purpose, as it allows us to capture the photochemical system's state immediately before (2019), during (2020), and after (2021) this critical transition. The convergent evidence from three independent methods (satellite FNR, WRF-CAMx, and OBM) across this specific window provides the strongest possible validation for the robustness of our central observation.

(2) Data Availability Constraint of the Observation-Based Model (OBM): The primary and most stringent constraint was the availability of high-quality, high-temporal-resolution speciated VOC data, which are essential for running the OBM. At the time of our analysis, the comprehensive and consistent hourly concentration dataset, covering 57 VOC species across our selected representative cities, was available only for the summers of 2019, 2020, and 2021. We were unable to obtain similarly quality-controlled VOC speciation data for all seven cities for 2022 and 2023, which prevented us from extending the OBM analysis beyond 2021.

(3) Methodological Consistency and Emission Inventory Limitation: To ensure a rigorous, 'apples-to-apples' cross-validation, the simulation period for the WRF-CAMx model was aligned with the OBM analysis period (2019–2021). Furthermore, the WRF-CAMx simulations in Fig. 2a utilized the MEIC v1.4 inventory for 2019–2020, as this version was only updated to 2020 at the time of our study. The 2021 emission inventory was refined from the 2020 data based on provincial-level total atmospheric pollutant emission data collected for 2021. Detailed in Supplementary Text 5: WRF-CAMx Model Configuration and Performance Evaluation (lines 349-363)：

"......Emissions outside of China were derived from the Regional Emission Inventory in ASia version 2.1 (REAS v2.1) (original resolution 0.25° × 0.25°)[25]. For 2021, as the official MEIC v1.4 inventory was updated only up to 2020 at the time of this study, we refined the emission data to reflect the activity levels of that year. This

involved using the MEIC 2020 inventory as a spatial distribution baseline and collecting provincial-level total atmospheric pollutant emission data for 2021. We then calculated the provincial total change ratios for each major precursor (2021 vs. 2020) based on these latest statistics. These ratios served as scaling factors, which were applied to adjust the grid-level emissions of the corresponding provinces within the MEIC 2020 inventory. This approach updated the total emissions to levels more representative of 2021 while preserving the validated fine spatial distribution of the MEIC inventory. This method assumes relatively stable internal spatial emission distributions within each province over a single year, a common and reasonable approximation when high-resolution, updated inventories are unavailable. These inventories were converted to the LCC projection coordinate system using geographic distribution factors......"

These clarifications have been incorporated into our revised manuscript, as stated in lines 200–204 of the main text:

"The specific period (2019-2021) was selected to scientifically bracket the 2020 inflection point and was determined by the availability of comprehensive, ground-based speciated VOC measurements required for the OBM analysis, as well as the emission inventory data available for CTM simulations."

We hope that this explanation and the corresponding revision fully address the reviewer's valid concern, clarifying that our choice of years was based on methodological rigor and data-driven constraints, rather than arbitrary selection. We are grateful to the reviewer for prompting this important clarification.

6. In Fig. 2b, four cities in the NCP are used for corroboration, but Zhengzhou is not described. Furthermore, only one city in the YRD (Shanghai) and no cities in the PRD are used. To enhance the generalizability of your conclusions, please explain the selection process for these cities and why some regions have fewer data points, such as due to data availability.

Reply: We sincerely thank the reviewer for the meticulous comments regarding the city selection criteria and the interpretation of the Observation-Based Model (OBM) results in Fig. 2b. The reviewer accurately identified that the rationale for city

selection and the cross-regional representativeness needed further clarification. We fully agree that this is crucial for substantiating the generalizability of our urban-scale validation.

In response to your valuable feedback, we have made substantial revisions to the manuscript to enhance its clarity and rigor.

(1) Clarification of City Selection Criteria and Regional Representation:

We have now explicitly stated the selection criteria for the cities used in the OBM analysis. The primary and most stringent criterion was the availability of robust, continuous, and high-quality hourly speciated VOC monitoring data for the summers of 2019-2021, a prerequisite for reliable OBM simulations. Such robust, continuous, multi-precursor datasets are rare at a broad spatiotemporal scale. This scarcity was particularly pronounced as most cities relied on manual VOCs sampling (3 samples per day) in 2020, which explains why it was not feasible to include a larger, more evenly distributed set of cities that meet these stringent criteria. Notably, during the revision process, we successfully obtained high-quality hourly data for Guangzhou, representing the Pearl River Delta (PRD) megacity clusters. In the NCP megacity clusters, where high-quality hourly VOC data were relatively more abundant, the four selected cities represent diverse levels of economic development: Beijing as a highly developed metropolis, Xiong'an as an emerging city, and Zhengzhou and Jinan as moderately developed cities. While the number of cities is limited, they provide critical urban-scale insights that complement our regional-scale satellite and chemical transport model analyses.

These revisions have been incorporated into our manuscript, as shown in Lines 180-194 of the revised main text:

"Additionally, to complement the regional-scale satellite and model analyses and to resolve the trends in O_3 sensitivity at the urban-core scale—a domain where satellite and chemical transport model simulation have known limitations[15]—we employed an Observation-Based Model (OBM, detailed in Supplementary Text 6). Constrained by in-situ hourly measurements of VOCs and NO_x (Supplementary Text 2), the model diagnoses sensitivity via relative incremental reactivity (RIR). This analysis (Fig. 2b) was conducted in seven cities, selected from the three megacity clusters (Supplementary Fig. 1), possessing robust data foundations, and representing diverse economic development levels in China. The inter-annual RIR trends

corroborated this stagnation: the progression towards a NO*x*-limited regime stalled in 2020 for Beijing, Tianjin, Jinan, Xiong'an (NCP), and Guangzhou (PRD), and as early as 2019 for Shanghai (YRD). In the case of Zhengzhou, although its O_3 sensitivity continued to trend towards NO*x*-limited conditions during the 2019 – 2021 summers, it remained persistently within the VOC-limited regime, thus still necessitating the prioritization of AVOC controls. "

[Figure Redacted]

Supplementary Fig. 1. Schematic diagram of the master grid (36 km × 36 km) for CAMx simulations, the regions of three megacity clusters designated by the Chinese government as targets for air pollution abatement, the location distribution of the national control station monitoring stations for conventional pollutants (O_3 and NO_2 etc.), and monitoring stations with VOC monitoring (red dots). The three megacity clusters are as follows: the North China Plain (NCP, coloured blue, 31°–42° N, 112°–123° E, 368 sites), the Yangtze River Delta (YRD, coloured pink, 27°–35° N, 114°–122° E, 223 sites) and the Pearl River Delta (PRD, coloured green, 21.5°–25° N, 111°–115.5° E, 81 sites). The map data are provided by the Resource and Environment Science and Data Center © Institute of Geographic Sciences and Natural Resources Research, Chinese Academy of Sciences (https://doi.org/10.12078/2023010103, 2023)."

Furthermore, in Supplementary Text 2, we now explicitly list the seven cities and their respective data sources, transparently addressing the reviewer's point on "data availability."

Supplementary Text 2 (Lines 117-127):

"Hourly concentrations of 57 speciated VOCs, encompassing 29 alkanes, 10 alkenes, 1 alkyne, and 17 aromatics, were obtained for the summers (June–August) of 2019–2021 from seven representative cities: Beijing, Tianjin, Xiong'an, Zhenzhou and Jinan within the NCP region, Shanghai in the YRD region, and Guangzhou in the PRD region. In the NCP megacity clusters, where high-quality hourly VOC data were relatively more abundant, the five selected cities represent diverse levels of economic development: Beijing as a highly developed metropolis, Xiong'an as an emerging city, and Tianjin, Zhengzhou and Jinan as moderately developed cities. Data were sourced

from the CNEMC, the Shanghai Academy of Environmental Sciences and Guangzhou Ecological and Environmental Monitoring Center, respectively. ”

(2) Detailed Interpretation of Zhengzhou's and PRD's Results:

We are grateful to the reviewer for pointing out the lack of description for Zhengzhou and the PRD city. In the revised manuscript, we have now included a detailed interpretation for all seven cities, including a nuanced discussion of the findings for Zhengzhou. This not only addresses the reviewer's concern but also strengthens our argument by demonstrating the robustness of our framework across varied local conditions.

The following text has been added to the main text (Lines 191-194 of the revised manuscript):

"In the case of Zhengzhou, although its O_3 sensitivity continued to trend towards NO*x*-limited conditions during the 2019－2021 summers, it remained persistently within the VOC-limited regime, thus still necessitating the prioritization of AVOC controls. "

This addition now fully explains the data presented in Fig. 2b. The consistent stagnation signal in six of the seven cities, and the persistent VOC-limited nature of the seventh, collectively provide strong, ground-level evidence supporting our regional-scale findings.

We hope these comprehensive revisions directly and thoroughly address the reviewer's concerns, significantly enhancing the transparency and scientific soundness of our urban-scale validation. We thank you again for the opportunity to make these improvements.

7. Why was OMI data not used in Fig. 3? Using OMI, which has a longer time series (e.g., from 2005 onwards), would align better with the MEIC emission inventory data (2005–2020) shown in Fig. 3a, providing a more complete historical trend analysis.

Reply: We appreciate the reviewer’s insightful suggestion regarding the temporal alignment of satellite observations with the MEIC emission inventory. We carefully considered utilizing the longer OMI record (2005–present) for Fig. 3 but ultimately

decided to retain TROPOMI for the specific analysis of the recent "plateau period" for the following reasons:

1. Distinct Roles of Fig. 1 and Fig. 3: We agree that a complete historical trend analysis is essential. This is why Fig. 1b already presents the full long-term time series of satellite-derived FNR from 2005 to 2024, utilizing OMI data for the earlier period and TROPOMI for the recent period. This provides the "complete historical trend analysis" the reviewer rightly seeks, aligning with the long-term emission trends.

2. Data Fidelity and Sensor Degradation: The primary objective of Fig. 3 is to scrutinize the driving mechanisms, particularly for the newly identified "synchronous plateau" (2020–2023). Precise diagnostics of this recent phenomenon require high-fidelity data. As noted in the literature and our manuscript, the OMI sensor has suffered significant degradation in recent years, manifesting as severe striping noise in HCHO retrievals. We have added Supplementary Fig. 5 in the revised Supplementary Information to visually demonstrate this issue. It clearly shows that OMI data in 2022 exhibits distinct striping noise compared to the cleaner retrievals from TROPOMI, justifying our choice to rely on TROPOMI's superior signal-to-noise ratio and spatial resolution (7×7 km^2) for the detailed recent analysis in Fig. 3.

3. Sensitivity Trend Consistency: As shown in Fig. 1 and Supplementary Tables 2, 4, and 6, the trends derived from TROPOMI and OMI during their overlap period (2018-2020) are consistent, ensuring that the switch in sensors does not introduce artifacts into our trend analysis.

This critical detail has been added to the main text of the revised manuscript (lines 154-158) , the caption of Fig. 1b, Supplementary Fig. 5 and Supplementary Text 4.

8. The WRF-CAMx model's performance was evaluated using only the year 2020. What is the model's generalization ability for other years? Please provide evidence that the model is reliable for long-term simulations spanning from 2020 to 2060.

Reply: We sincerely thank the reviewer for raising this question regarding the generalization ability and long-term reliability of our model in future predictions. We fully agree that providing robust model performance validation is paramount for a predictive study spanning decades.

While Supplementary Text 5 (lines 377-433) details the performance evaluation for the base year 2020, it is essential to emphasize that the WRF-CAMx system utilized in this study is a mature platform, rigorously developed, continuously refined, and extensively validated by our research team over many years. The long-term reliability and generalization ability of this system have been unequivocally demonstrated in our prior publications. Specifically, we have successfully applied this identical modeling framework to simulate atmospheric pollutant concentrations across diverse years, with comprehensive validation against observed data showing robust performance in key metrics, thereby confirming its credibility for multi-year simulations[3,31,32]. Moreover, this system has been successfully employed for scenario-based simulations exploring various NO*x* and VOCs emission reduction ratios, consistently yielding satisfactory performance evaluations[33].

The model configuration, physicochemical schemes, and parameterization settings employed in this study are entirely consistent with those of these previously validated works. This consistency provides strong assurance for the model's reliability when extrapolating from validated past years to our projected future scenarios (2020–2060).

To make this explicit for the reader, we have added the following sentence to Supplementary Information, Text 5 (SI, lines 428-433):

'In summary, the model version, physicochemical schemes, and parameterization configurations used in this study are fully consistent with our previous work[3, 31-33], and this extensively validated WRF-CAMx system has demonstrated robust performance in simulating NO_2 and O_3 concentrations across multiple years and various emission control scenarios, providing strong evidence of its generalization ability and reliability under diverse conditions.'

References:

3 Du, X. et al. Modeling of spatial and temporal variations of ozone-NO*x*-VOC sensitivity based on photochemical indicators in China. J. Environ. Sci. **114**, 454 – 464 (2022).

31 Du, X. et al. Sensitivity modeling of ozone and its precursors over the Chengdu metropolitan area. Atmos. Environ. **277**, 119071 (2022).

32 Du, X. et al. Improving photochemical indicators for attributing ozone sensitivities in source apportionment analysis. J. Environ. Sci. **143**, 235 – 246 (2024).

33 Du, X. et al. Responses of ozone concentrations to the synergistic control of NO*x*

and VOCs emissions in the Chengdu metropolitan area. Front. Environ. Sci. **10**, 1024795 (2022).

We are confident that this clarification, coupled with the explicit references in the Supplementary Information, fully addresses the reviewer's concerns regarding the model's generalization ability and long-term simulation reliability.

9. In Supplementary Text, on line 108, please correct "UT+8" to the standard "UTC+8."

Reply: Revised as suggested.

10. In Supplementary Table 9, there are two "R" values. Please clarify in the table caption what each R value represents, for example, one for the training set and one for the testing set. The significant difference between these two R values suggests a potential overfitting issue. Please address this and discuss its potential impact on the robustness of your model's conclusions.

Reply: We are deeply grateful for the reviewer's insightful comment concerning original Supplementary Table 9, which has enables us to enhance the clarity and transparency of our Generalized Boosting Machine (GBM) performance evaluation. The reviewer highlighted the need for a clearer explanation of the 'R' values and raised a pertinent concern regarding potential overfitting.

The values presented in original Supplementary Table 9 (R, RMSE, and MB) are indeed for the test set (comprising 20% of the total data, as detailed in Supplementary Text 3, lines 175-178), not the training set. The format 'Mean (min, max)' indicates the average performance statistics calculated over the entire 12-year analysis period (e.g., average Pearson R value), with the minimum and maximum observed values during that period provided in parentheses. This signifies a consistent and robust predictive performance across unseen data for each region throughout the entire analysis period.

Regarding the concern about overfitting, the robust performance metrics on the test set directly address this issue. Average R values for the major megacity clusters range between 0.78 and 0.83, demonstrating the strong generalization capability of

our GBM model to effectively adapt to new, unseen meteorological conditions. This indicates that the model effectively accounts for meteorological variability when capturing emission-driven O_3 trends, without overfitting the training data. Furthermore, the GBM model itself intrinsically incorporates mechanisms to mitigate overfitting through its ensemble learning approach and controlled learning rates, thereby yielding a more robust and stable model. The high correlation coefficients on the test data underscore the reliability of our de-weathering procedure in isolating emission-driven O_3 trends.

To address the reviewer's comment, we have made the following specific revisions:

(1) Original Supplementary Table 9 Caption:

Original: "Supplementary Table 9. Mean GBM model performance statistics for the analysis"

Revised: "**Supplementary Table 12. Mean GBM model performance statistics for the testing set in each megacity cluster.** Values are presented as mean (minimum, maximum) over the 12-year analysis period (2013-2024), where 'R' denotes Pearson’s correlation coefficient, 'RMSE' is Root Mean Square Error (μg m^{-3}), and 'MB' is Mean Bias (μg m^{-3})."

(2)Supplementary Text 3 Lines 188-195:

Added: "These statistics specifically reflect the model's performance on the 20% testing subset, demonstrating its strong generalization capability and absence of severe overfitting. The high R values (ranging from 0.78 to 0.83 for the mean) and low RMSE/MB values for the testing set confirm the model's robustness in capturing emission-driven O_3 trends while accounting for meteorological variability. The ensemble nature of the GBM algorithm, which combines multiple weak learners, inherently mitigates the risk of overfitting by aggregating diverse predictions

Supplementary Table 12. Mean GBM model performance statistics for the testing set in each megacity cluster. Values are presented as mean (minimum, maximum) over the 12-year analysis period (2013-2024), where 'R' denotes Pearson’s correlation coefficient, 'RMSE' is Root Mean Square Error (μg m^{-3}), and 'MB' is Mean Bias (μg m^{-3})

	R	RMSE	MB
NCP	0.83(0.66, 0.9)	24.31(19.58, 31.25)	-0.06(-3.95, 4.53)

YRD	0.82(0.72, 0.9)	23.58(14.39, 30.29)	0.37(-2.22, 5.47)
PRD	0.78(0.67, 0.87)	24.62(17.39, 34.64)	-0.22(-2.73, 5.26)

"

We hope these additions and clarifications fully address the reviewer's valuable comments, enhancing the methodological transparency and the confidence in our model's conclusions.

A point-by-point response to the reviewers' comments

We are very thankful to the reviewers for the valuable comments and thoughtful suggestions. We have carefully addressed all of the reviews' comments and revised the manuscript accordingly. The followings are our point-by-point responses to the comments. The comments of the reviewers are shown in black in *italics*, our responses to the comments are presented in black, and the added or revised texts are provided in blue.

Response to Reviewer #1

Reviewer #1 (Remarks to the Author):

I appreciate the authors' thorough response to my comments. The additional data and revised discussion have clarified the previous ambiguities. I have no further concerns and support the publication of this paper in its current form.

Reply: We are very grateful for the reviewer's positive feedback and the support for the publication of our manuscript.

Response to Reviewer #2

Reviewer #2 (Remarks to the Author):

Second Review: Aligning climate and air quality goals demands early VOC control for China's ozone pollution
Authors: Y. Zhang et al.

***Summary:** This study combines historical observations of ozone response to NOx/VOC concentrations with future emissions pathways to analyze approaches to reduce O_3 concentrations while simultaneously achieving climate action goals. The major conclusion of the study is that early VOC reductions are the most effective pathway to achieve these objectives.*

Authors have successfully revised the manuscript to better explain the CDTR method and they have also performed additional analysis to explore how evolving VOC composition could affect the results. These updates address two of the major issues with the original manuscript, but several key issues remain.

Overall Recommendation: Additional work should be performed to run model calculations at an appropriate spatial resolution for urban studies and to resolve discrepancy between formaldehyde to NO_2 ratios observed by TROPOMI and predicted by models.

Reply: We thank the reviewer for the rigorous and thought-provoking comments. We have carefully addressed each of the three major points raised through substantial revisions to the manuscript and Supplementary Information, as detailed below.

Comments:

1. Figure 1 shows that formaldehyde to NOx ratios are increasing over time. This would suggest that the O_3 formation chemistry is shifting to a NOx-limited chemical regime, where additional NOx control would reduce O_3 concentrations. The authors acknowledge this in the revised manuscript but go on to define a target where VOC controls are preferred in the "AVOCs reduction bonus window" where one kg of AVOC reduction is more effective at reducing O_3 concentrations than one kg of NOx reduction. This analysis seems overly simplistic since the effort required to reduce AVOC emissions by one kg is not equal to the effort required for each kg of NOx reduction. Emissions control programs typically encounter increasing costs per kg of pollutant reduction since they take advantage of easy controls first. The definition of the "AVOCs reduction bonus window" is overly simplistic and does not account for the increasing cost of AVOCs controls. Since the goal of the

manuscript is to map out an optimal O_3 control pathway, this issue merits more discussion. It isn't clear to the reader that the recommendation for heavy reliance on AVOC controls through the end of the "AVOCs reduction bonus window" is truly the optimal path. Furthermore, calculating the size of the "AVOC reduction bonus window" requires very accurate modeling tools but significant uncertainties remain in the validation of the WRF/Chem model. See comments below.

Reply: We deeply appreciate the reviewer's critical and highly insightful perspective regarding the economic dimensions of emission control strategies. We fully agree that determining a truly "optimal" real-world policy requires a holistic assessment that bridges both photochemical efficacy and techno-economic feasibility.

The reviewer is absolutely correct in pointing out that our definition of the "AVOCs reduction bonus window" is evaluated purely on the basis of photochemical efficacy (i.e., the net MDA8 O_3 mitigation benefit achieved per equivalent proportional reduction in precursors), without incorporating techno-economic costs. We wholeheartedly agree that real-world abatement programs encounter increasing costs per kg of pollutant reduced, as the "low-hanging fruits" are harvested first. This economic reality is particularly challenging for AVOCs, where practical abatement poses steeper hurdles and relies on fragmented, sector-specific technologies, unlike NO_x controls that naturally synergize with climate-driven energy transitions. Furthermore, it is important to contextualize these findings within the specific realities of China's current emission control landscape. Comprehensive AVOC control is a relatively recent endeavor, lagging significantly behind the decades-long efforts dedicated to NO_x abatement. Consequently, substantial "low-hanging fruits" for AVOC reductions remain accessible across various industrial sectors. From a techno-economic perspective, this suggests that prioritizing AVOC controls at this nascent stage does not inherently represent a higher-cost pathway. This is particularly relevant when weighed against the escalating marginal costs of further NO_x reductions, given that the most cost-effective NO_x abatement technologies have already been deployed.

However, we wish to clarify our strategic intent: identifying this chemically optimal pathway is the indispensable scientific prerequisite for any subsequent cost-benefit analysis. While practical AVOC abatement is complex and costly, our

findings reveal that the photochemical advantage of AVOC reductions is progressively diminishing over time. This transient nature dictates that the temporal urgency of AVOC control (AVOCs reduction bonus window) must be dynamically integrated into future economic modeling. If economic models fail to first establish the chemically correct target and timing, they risk falling into the pitfall of what we term ‘precise inaccuracy’—mathematically optimizing economic costs at extreme precision for a strategy that is suboptimal or invalid in atmospheric chemistry.

A compelling example of this “precise inaccuracy” is demonstrated in our ‘only dual carbon’ scenario. If we miss the AVOCs reduction bonus window and rely heavily on the more tractable NO_x controls, even a massive 84% reduction in NO_x emissions relative to 2020 levels fails to bring regional summertime MDA8 O_3 concentrations in the NCP down to the WHO guideline of 100 μg m^{-3}. This highlights that relying on NO_x controls without seizing the AVOC window—regardless of how economically optimized the NO_x reduction path might be—is chemically insufficient to achieve the ultimate health guidelines.

Following the reviewer’s constructive suggestion, we have added a dedicated discussion on this critical issue to clarify that our “optimal path” serves as a photochemical blueprint.

Modifications in the revised manuscript (Lines 421-426):

Fig. 6 synthesises our findings into a dynamic policy blueprint for an optimal O_3 mitigation strategy that aligns clean air and climate objectives...... The optimal O_3 mitigation strategy proposed in our study is defined on the basis of photochemical efficacy (i.e., the MDA8 O_3 abatement achieved per equivalent proportional reduction in precursors), independent of techno-economic complexities. However, for the design and implementation of real-world emission control policies, integrating this photochemical optimization with practical economic cost assessments is essential.

We thank the reviewer again for prompting this crucial discussion, which has significantly elevated the macroscopic policy value of our manuscript. Regarding the uncertainties in the model validation, we have addressed them in our response to your subsequent comments below.

2. WRF-CAMx simulations conducted to analyze the effects of various emissions scenarios were carried out at 36km resolution (see extended data Fig 1). This is very coarse for a city-specific analysis of ozone trends. The authors show results from observational based methods based on point measurements made at major cities to build confidence in those model results, but they do not directly compare model predictions to measurements. At best, the current analysis shows that model results and measurements predict qualitatively similar trends for the effectiveness of AVOC vs. NOx controls, but they do not prove that the modeling system is capable of the refined analysis needed for the analysis of "AVOCs reduction bonus windows" that are used in the rest of the analysis. A direct quantitative comparison should be made between model predictions for O_3 sensitivity and measurements of O_3 sensitivity. This will likely require a nested model approach that scales down to 4km or even 1km resolution over major cities. The WRF/Chem model is perfectly capable of these calculations.

Reply: We are profoundly grateful for the reviewer's sustained and insightful suggestions regarding model resolution and the quantitative validation of O_3 sensitivity. The reviewer's probing into whether our modeling system can support the "refined analysis" necessary for our conclusions touches upon the methodological underpinning of our study. This has provided us with an invaluable opportunity to systematically clarify the core logic of our research. Herein, we wish to first address the reviewer's most fundamental concern: whether the model retains the capacity to robustly quantify outcomes like the "AVOCs reduction bonus window" despite utilizing a 36 km resolution and exhibiting baseline FNR offsets in 2020 (the detailed results of the direct quantitative comparison of O_3 sensitivity are thoroughly elucidated in our response to Comment 3). We provide the following structural clarifications, which have now been fully integrated into the revised manuscript:

(I) The Prudent Choice of 36 km Resolution: Balancing Regional Scope, Long-term Timescales, and Computational Feasibility

We wish to clarify that the primary objective of this study is to investigate the path dependency of O_3 photochemistry at the macro-scale of China's megacity clusters over a multi-decadal span (2005–2060). We focus on how macro-scale energy and emission transitions, driven by synergistic climate and clean-air policies, shape long-term O_3 formation response across broad geographical domains. For this specific

macro-objective, 36 km is a widely adopted and scientifically sound resolution choice, striking a necessary balance between capturing regional transport and synoptic-scale chemical processes versus computational feasibility. For instance, among our references, Liu et al. (*Environ. Sci. Technol.*, 2023) employed a WRF-CMAQ resolution of 36 km × 36 km; Li et al. (*Nat. Geosci.*, 2019) utilized GEOS-Chem at 0.5°×0.625° (a typical mesoscale resolution, equating to approx. 56 km × 53 km in the NCP); Li et al. (*PNAS*, 2021) investigated O_3 in the NCP using GEOS-Chem at 0.25°×0.3125° (approx. 28 km × 27 km); and Qin et al. (*Nat. Geosci.*, 2024) used WRF-Chem at 27 km × 27 km.

(II) The Purpose of City-Scale Analysis: A "Stress Test" for the Regional Dynamic Signal

We completely agree with the reviewer that a 36 km resolution is too coarse for precise urban-level analysis. It is well-documented that O_3 formation in urban cores exhibits stronger VOCs sensitivity compared to the broader regional scale. Consequently, if even the most stringently VOC-limited urban centers exhibit the identical sensitivity evolutionary trend as the broader region, this serves as the "stress test." It proves, from the rigorous perspective, that the temporary stagnation in the transition towards a NO*x*-limited regime—captured by our regional satellites and model method—is a genuine and robust signal (our urban OBM results indicate that the interannual trend of O_3 sensitivity in the majority of cities aligns with the regional trend). We thank the reviewer for the reminder.

To articulate this to the readership, we have revised the ambiguous phrasing in Lines 175-184 of the original manuscript. The revision articulates our regional-scale objective and clarifies the role of the urban-scale RIR trend analysis as a stress test.

Original Manuscript Lines 175-184:

"Additionally, to complement the regional-scale satellite and model analyses, and to resolve the trends in O_3 sensitivity at the urban-core scale—a domain where satellite observation and CTM simulation have known limitations[15]—we employed an Observation-Based Model (OBM, detailed in Supplementary Text 6). Constrained by in-situ hourly measurements of VOCs and NO*x* (Supplementary Text 2)......thus still necessitating the prioritization of AVOC controls."

Revised Manuscript Lines 175-187:

"To ensure that the regional sensitivity trend plateau revealed by satellite FNR (Fig. 1) was not an artefact of a single metric, we further conducted a multi-scale cross-validation spanning 2019–2021 using two widely applied independent methods (Fig. 2). First, at the regional scale congruent with our primary satellite-based finding, chemical transport model (CTM, the WRF-CAMx detailed in Supplementary Note 4) simulations revealed that NO*x*-limited areas peaked and that VOC-limited areas were minimised in 2020 (NCP) or 2019 (PRD, YRD). Furthermore, to stress-test these regional findings, we deployed an Observation-Based Model (OBM; detailed in Supplementary Note 5) at the urban-core scale. Given the well-documented spatial heterogeneity wherein urban centers typically exhibit a stronger sensitivity to VOCs relative to the broader regional background, observing an evolutionary trend across these local sites that aligns with the broader regional trajectory provides compelling auxiliary validation."

4. Liu, Y. et al. Drivers of increasing ozone during the two phases of clean air actions in China 2013–2020. *Environ. Sci. Technol.* **57**, 8954–8964 (2023).

6. Li, K. et al. A two-pollutant strategy for improving ozone and particulate air quality in China. *Nat. Geosci.* **12**, 906–910 (2019).

10. Qin, Y. et al. Amplified positive effects on air quality, health, and renewable energy under China's carbon neutral target. *Nat. Geosci.* **17**, 411–418 (2024).

44. Li, K. et al. Ozone pollution in the North China Plain spreading into the late-winter haze season. *Proc. Natl. Acad. Sci. USA* **118**, e2015797118 (2021).

(III) The Inevitability of Absolute FNR Discrepancies and the Robustness of Our Quantitative Conclusions

The reviewer acutely points out: without a direct quantitative matching of FNR measurements, how can the model accurately calculate the "AVOCs reduction bonus window"? This question strikes at the core innovation of our proposed CDTR framework, which was designed to bypass the well-documented systematic biases inherent in the absolute values of sensitivity indicators.

(1) The Systemic Discrepancies of Absolute Sensitivity Indicators: As elaborated in Supplementary Note 1, absolute sensitivity indicators (such as FNR values or the areal proportion of different sensitivity regimes) exhibit significant, systemic discrepancies across different methodologies (satellite vs. model vs. in-situ). Substantial absolute value offsets even persist among different official retrieval products from the same satellite sensor. We argue that relying on comparisons of these inconsistent absolute values for static "sensitivity zoning" is a major source of the contradictory conclusions present in the literature regarding regional summer O_3 sensitivity in China.

(2) Our Solution—The CDTR Analysis Framework: Our framework shifts the focus from fragile absolute values toward two robust relative metrics:

- **Robustness of the Sensitivity Trend:** As the reviewer correctly noted, FNR in our study serves a dual role—as a qualitative trend-diagnostic tool and a demonstrator of O_3 formation path dependency—but never as a basis for quantitative determination:

a. In historical analysis (CDTR Stage 1): We analyze the coupled trend between satellite FNR and de-weathered O_3 to identify dynamic signals, such as the "synchronous plateau." This provides qualitative directional guidance for designing future policy scenarios (i.e., indicating that intensified AVOCs control is currently more efficacious). It dictates when to pay attention to what phenomenon and the implications thereof, rather than relying on the precise numerical value of FNR.

b. In future projections (CDTR Stage 2): We simulate the evolution of FNR to graphically illustrate the evolutionary trajectory of the photochemical system under varied emission reduction pathways (Fig. 4a, and Supplementary Fig. 6). This enables us to visualize and challenge the conventional paradigm, demonstrating that an accelerated transition to a high-FNR (NO*x*-limited) state does not necessarily yield the maximum O_3 abatement.

- **Robustness of the MDA8 O_3 Differential Analysis:** Most importantly, our core conclusion—the quantification of the "AVOCs reduction bonus window"—successfully bypasses any sensitivity indicators. This window is defined via a differential calculation across paired scenarios: comparing the direct change in O_3 concentrations (ΔMDA8 O_3) under varying pathways (Fig. 5 and Supplementary Fig. 7). The quantitative capacity of the CDTR framework relies exclusively on the relative differential response of MDA8 O_3. Because all scenarios operate from the

exact same model baseline, this paired differential analysis mathematically and intrinsically mitigates systematic model biases (e.g., those arising from underlying chemical mechanisms or baseline emission inventories). Consequently, the model's capability to accurately project the relative effectiveness of different control pathways remains robust.

To ensure this critical logical step is transparent to the readership, we have revised the manuscript at Lines 471–476 and added Lines 805-814 in the Methods section, explicitly elucidating the advantages of our paired-scenario differential analysis.

Original Manuscript Lines 460-463:

"Ultimately, we define the closure of the "bonus window"—the tipping point into a NO*x*-limited regime—by comparing the O_3 abatement efficacy of equivalent proportional reductions in AVOCs versus NO*x*."

Revised Manuscript Lines 471-476:

"Ultimately, we define the closure of the "bonus window"—the tipping point into a NO*x*-limited regime—by comparing the O_3 abatement efficacy of equivalent proportional reductions in AVOCs versus NO*x*, successfully bypassing the utilization of FNR absolute values (Fig. 5). Consequently, the robustness of our conclusions regarding the optimal mitigation strategy is independent of systematic methodological offsets in absolute baseline FNR determinations."

Added in Revised Manuscript Lines 805-814 (Methods):

"Therefore, in this study, we emphasize that the shift in regional summer O_3 formation regimes—and the definition of the "AVOCs reduction bonus window"—is quantified based exclusively on the relative mitigation efficacy of equivalent proportional reductions in precursors on simulated MDA8 O_3 concentrations within paired emission scenarios. Crucially, this quantification relies entirely on the direct response of surface O_3 concentrations (ΔO_3), thereby not only circumventing the uncertainties associated with absolute FNR baseline values, but also intrinsically mitigating systematic model biases—such as those originating from underlying chemical mechanisms or baseline emission inventories—as these systemic errors largely cancel out through the differential analysis of paired scenarios."

(IV) Embracing High-Resolution Modeling as a Key Implication and Future Direction

Inspired by the reviewer's constructive suggestions, we acknowledge that while our framework captures macro-scale shifts in regional sensitivity, ultra-high resolution is indispensable for resolving urban O_3 control strategies due to the spatiotemporal heterogeneity of O_3 photochemistry. We have expanded the Discussion section (Lines 557–576) to elaborate that urban centers require stronger VOCs control measures compared to broader regions. As illustrated in Supplementary Fig. 13, although the broader PRD region is projected to pioneer the transition to a NO*x*-limited regime by 2045, the drastic NO*x* reductions driven by climate actions paradoxically sustain localized MDA8 O_3 rebounds (relative to 2020) in sporadic urban hotspots even as late as 2060, indicating that O_3 formation in these micro-environments remains VOC-limited. The dynamic adaptability and iterative nature of the CDTR allow it to incorporate higher-resolution models in the future, as computational power increases and terminal control technologies evolve, to design differentiated urban-versus-regional control strategies.

Specific revisions in the manuscript (Lines 557-576):

"Furthermore, the spatial heterogeneity of O_3 sensitivity indicates that urban centers will require aggressively differentiated AVOC control efforts to counteract the O_3 rebound risks triggered by the substantial NO*x* reductions inherent to climate actions (Supplementary Figs. 11–13). A compelling illustration is observed in the PRD (Supplementary Fig. 13), although the broader PRD region is projected to pioneer the transition to a NO*x*-limited regime by 2045, these drastic NO*x* curtailments paradoxically sustain localized MDA8 O_3 rebounds—relative to the 2020 baseline—in sporadic urban hotspots even as late as 2060, underscoring that such micro-environments remain stubbornly entrenched in a VOC-limited state. While our current regional modeling framework captures these macro-scale chemical transitions, future iterations of the CDTR framework will benefit from deploying nested air quality simulations at ultra-high urban-centric resolutions (e.g., 4 km or 1 km) to precisely resolve such fine-scale spatial heterogeneity in O_3 responses and to design differentiated urban vs. regional strategies. The future emission scenarios and model setups used in this study (e.g., fixed 2020 meteorology and biogenic emissions; maximum VOC and NO*x* reduction potentials set at 70% and 84% of 2020 levels,

respectively, based on literature[45,46]) represent reasonable estimates based on current knowledge. The adaptability of the CDTR method provides a robust tool for navigating future uncertainties by allowing projections to be updated with new observational data, different air quality model and future technological advancements."

We once again thank the reviewer for these profound insights, which have prompted us to make these critical structural clarifications. Our detailed discussion regarding the direct quantitative comparison of O_3 sensitivity is provided in the response to the subsequent comment.

3. The formaldehyde to NO_2 ratios observed by TROPOMI at NCP, YRD, and PRD are in the range of 4-4.5 for the year 2020 (see Fig 1). The formaldehyde to NO_2 ratio predicted for these same locations using the WRF-CAMx framework in 2020 are in the range 0.8 – 1.3 (see Fig 4). This calls into question the accuracy of the analysis for the future pathways, since they appear to be starting much deeper in the VOC-limited regime than what measurements would indicate. The authors state that this model vs. measurement discrepancy stems from the TROPOMI column measurement vs. the WRF/Chem surface prediction. This explanation is problematic for two reasons. First, the TROPOMI measurements serve as the basis for the observational analysis of O_3 sensitivity inherent in the CDTR method. If the satellite measurements are not representative of surface conditions, then how can they be used to diagnose O_3 chemical regime at the surface. Second, the WRF/Chem model is a 3D simulation of the atmosphere. The column concentrations of formaldehyde and NO_2 can be easily extracted from WRF/Chem results for comparison to TROPOMI results. This critical validation of the modeling system is necessary before the paper can be published, since the accuracy of the model calculations is a critical determinant of the validity of the study conclusions.

Reply: We express our sincere gratitude to the reviewer for this profound and highly incisive comment. The reviewer correctly pointed out that our previous explanation was oversimplified and lacked a critical direct "column-to-column" validation step. The systematic offset in absolute FNR values is one of the most

foundational challenges in O_3 sensitivity research. The rigorous demand for a direct "column-to-column" validation compelled us to restructure our methodological narrative, thereby profoundly enhancing the scientific rigor and methodological transparency of our manuscript.

Guided by the exceptional suggestions, we have performed the direct "column-to-column" validation and systematically revised the manuscript. Our detailed responses are structured as follows:

(1) Direct "Column-to-Column" Validation: absolute FNR offsets persist, yet evolutionary trends remain consistent

We extracted the monthly mean tropospheric vertical column densities (VCDs) of HCHO and NO_2 from the 3D WRF-CAMx outputs for typical cities during the summer of 2020 (the baseline year). We then conducted a direct "column-to-column" comparison against TROPOMI column FNR values processed via Google Earth Engine (TROP-GEE), as well as the TROPOMI column FNR values derived from the European Space Agency L2 products (TROP-ESA) originally used in our manuscript. The detailed methodologies and results are now presented in the newly added Supplementary Fig. 8 and Supplementary Note 3.

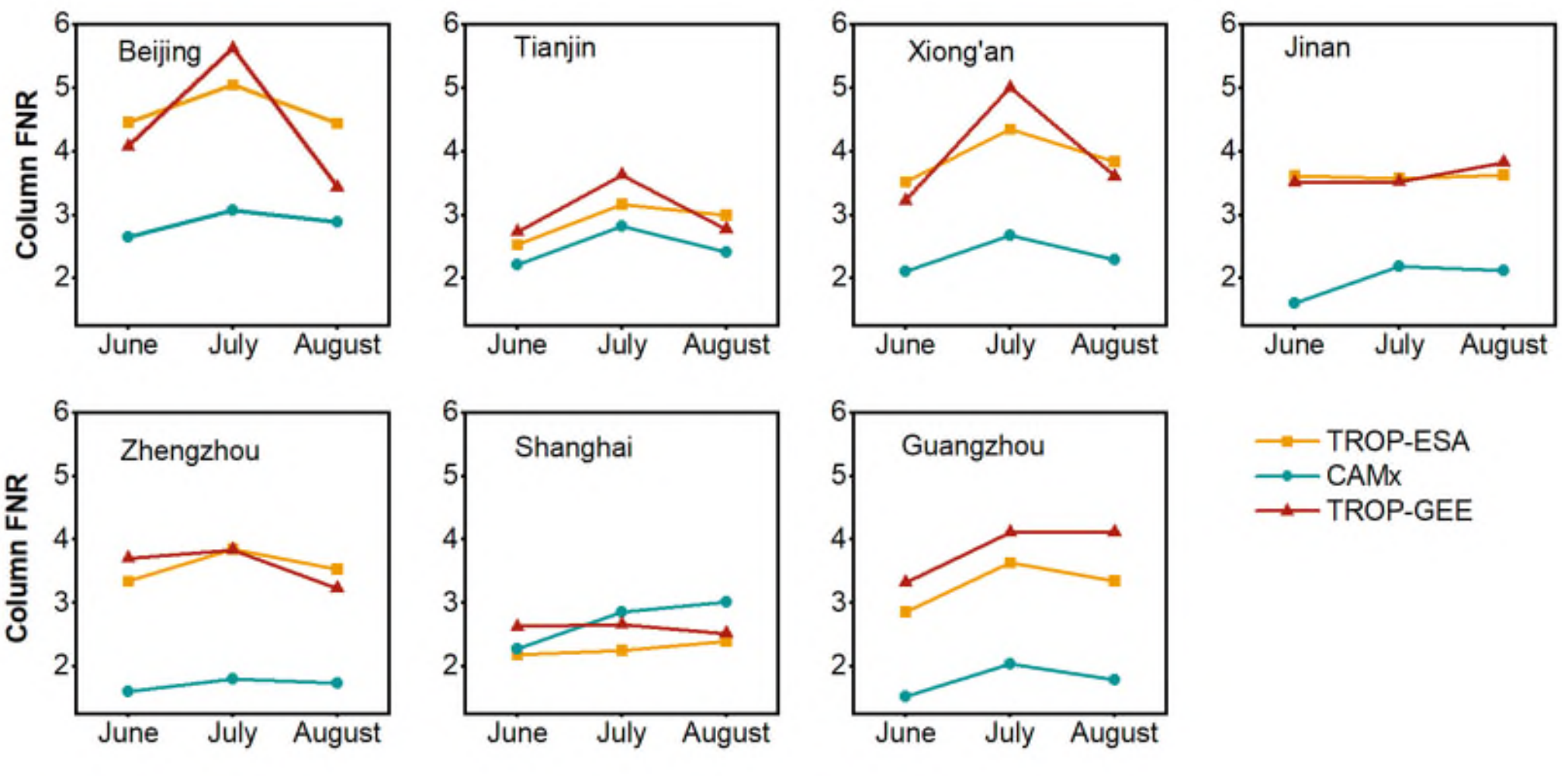

Supplementary Fig. 8. Consistent monthly variations in column FNR derived from CMAx simulations and TROPOMI observations across typical cities during 2020 summer. The satellite data were processed via two approach (TROP-ESA and TROP-GEE) **.**

This rigorous validation reveals a well-documented phenomenon: although the direct "column-to-column" comparison substantially narrows the discrepancy compared to the "column-to-surface" assessments, a systematic offset in absolute FNR values still persists between the model and satellite retrievals, and even between different satellite data processing pathways (TROP-GEE vs. TROP-ESA). Critically, however, all three datasets—the model-derived column FNR and both TROPOMI-derived FNRs—exhibit consistent temporal (month-to-month) evolutionary trends.

This persistent discrepancy in absolute FNR values does not imply a fundamental flaw in the model or the satellite; rather, it reflects inherent, multi-sourced systematic biases widely reported within the field. As demonstrated by Jin et al. (2017, JGR; Reference 35 in the manuscript), even absolute FNR values derived from different official retrieval products of the same satellite sensor (e.g., pairing SAO HCHO with DP NO_2 *vs.* BIRA HCHO with SP NO_2) can exhibit massive offsets (differences of 3 to 4) over the same region and time, yet they all capture the identical monthly evolutionary trends as the model-simulated columns.

(2)How satellite columns diagnose surface conditions

The reviewer raised a vital question: "*If satellite measurements are not representative of surface conditions, how can they be used to diagnose the surface O_3 chemical regime?*" The answer lies in the fact that the relationship between column and surface concentrations is dynamic, rather than static. The vertical distributions of HCHO and NO_2 are strongly modulated by the boundary layer mixing height and the variable lifetime of NO*x*. Jin et al. (2017) derived monthly column-to-surface FNR conversion factors across different regions (East Asia, Europe, and North America), demonstrating that these conversion factors exhibit stable relative evolutionary trends across different months within the same region.

This column-to-surface relationship highlights a fundamental insight: the diagnosis of O_3 sensitivity should rely on the evolutionary trends of satellite indicators, rather than on whether their absolute values cross a static threshold. Our study demonstrates that, despite systematic differences in absolute values, the evolutionary trends of sensitivity indicators derived from the same methodology could reflect the shifting reality of surface photochemistry.

(3) The core logic of the CDTR framework: Focusing on robust relative evolutionary trends to bypass the absolute FNR baseline

The reviewer expressed a highly valid concern: *If the model's absolute FNR starts deeper in the VOC-limited regime, it might skew future pathway projections.* This exact dilemma—the systematic discrepancies in static absolute FNR thresholds—is the challenge our Coupled Dynamic Trend Response (CDTR) framework was designed to overcome. Historically, the prevailing reliance on static FNR thresholds has likely been a major contributing factor to the contradictory conclusions regarding regional O_3 sensitivity in China (summarized in Supplementary Note 1).

By shifting the analytical focus from static absolute thresholds to relative evolutionary trends, our proposed CDTR framework intentionally insulates its core policy insights from these absolute baseline biases. The reviewer's valid concern highlights the absolute necessity of this paradigm shift.

(4) Ensuring the validity of quantitative future calculations: a calculation independent of any FNR value

To resolve concerns regarding the quantitative accuracy of the CDTR framework during the future projection phase, we explicitly clarify in the revised manuscript that the calculation of our optimal mitigation strategy and the duration of the "AVOCs reduction bonus window" is independent of absolute FNR values.

Specifically, we evaluate the optimal mitigation strategy by quantifying the direct response of simulated surface MDA8 O_3 concentrations *(*ΔMDA8 O_3) to equivalent proportional reductions in precursors under paired emission scenarios. Therefore, mathematically, this calculation bypasses FNR values. Consequently, the systematic bias in the absolute FNR baseline in no way undermines the robustness of our predictive conclusions.

To ensure transparency and clarity for the readers, we have made the following significant revisions:

- Methods Revision: We explicitly stated that the quantification of the AVOCs bonus window relies entirely on the relative ΔO_3 mitigation efficacy in paired scenarios, independent of absolute FNR values. (detailed in the section "Quantification method for the duration of the AVOCs reduction bonus window" in the Methods).
- Discussion Revision: We expanded the discussion to transparently address the systematic differences in absolute FNR values across different methodologies

(including the column-to-column comparison) and emphasized how our CDTR framework insulates policy insights from these absolute baseline biases.

- SI Revision: We added Supplementary Fig. 8 and corresponding text to demonstrate that despite the baseline offset, the monthly evolutionary trends between model and satellite column FNRs remain consistent. (see Supplementary Note 3).

The specific revisions in the manuscript are as follows:

Methods Lines 805-814：

"Therefore, in this study, we emphasize that the shift in regional summer O_3 formation regimes—and the definition of the "AVOCs reduction bonus window"—is quantified based exclusively on the relative mitigation efficacy of equivalent proportional reductions in precursors on simulated MDA8 O_3 concentrations within paired emission scenarios. Crucially, this quantification relies entirely on the direct response of surface O_3 concentrations (ΔO_3), thereby not only circumventing the uncertainties associated with absolute FNR baseline values, but also intrinsically mitigating systematic model biases—such as those originating from underlying chemical mechanisms or baseline emission inventories—as these systemic errors largely cancel out through the differential analysis of paired scenarios. "

Discussion Lines 447-476:

"A critical methodological consideration in O_3 sensitivity assessment is that discrepancies in the absolute values of derived FNR across different methodologies—whether from satellite retrievals or chemical transport models—remain systematic, multi-sourced, and inherently challenging to reconcile[13-18]. Even among different retrieval products derived from the same satellite sensor, substantial FNR value offsets persist (e.g., as demonstrated by Jin et al.[35]). Our direct 'column-to-column' FNR evaluation (Supplementary Fig. 8) during summer 2020 (baseline year) confirms that while such discrepancies are substantially reduced

compared to column-to-surface assessments—with both maintaining consistent temporal variations—a systematic baseline offset persists. Rather than viewing this inherent FNR variability as a limitation, it essentially highlights the necessity and the core rationale of our CDTR analytical framework. We posit that historical reliance on static absolute FNR thresholds for ‘sensitivity zoning’ largely drives the inconsistent conclusions regarding summer regional O_3 sensitivity in China (Supplementary Note 1). By shifting focus to the robust relative evolutionary trends of sensitivity indicators (Supplementary Note 3), our CDTR framework insulates core policy insights from these absolute FNR baseline biases. This dynamic perspective yields fundamental insights: by aligning with the sensitivity evolution direction, the observed synchronous plateau provides an empirical directive that prioritising AVOC control is the more effective strategy at present and guides the intensification focus of our future scenarios. Crucially, our simulations (Fig. 4) quantitatively demonstrate that O_3 mitigation efficacy does not necessarily align with the speed of transition towards a NO*x*-limited regime (the ‘only dual carbon’ scenario in Fig. 4a), thereby challenging the traditional paradigm that advocates for the fastest possible entry into NO*x*-limited conditions. Ultimately, we define the closure of the “bonus window”—the tipping point into a NO*x*-limited regime—by comparing the O_3 abatement efficacy of equivalent proportional reductions in AVOCs versus NO*x*, successfully bypassing the utilization of FNR absolute values (Fig. 5). Consequently, the robustness of our conclusions regarding the optimal mitigation strategy is independent of systematic methodological offsets in absolute baseline FNR determinations.”

Supplementary Note 3：Satellite NO_2 and HCHO data products.

“Tropospheric NO_2 and HCHO vertical column densities (VCDs) spanning the summers of 2018–2024 were sourced from the TROPOspheric Monitoring Instrument

(TROPOMI) onboard the Sentinel-5 Precursor (S5P) satellite. To rigorously validate our findings, data were compiled through two distinct processing pathways (Supplementary Fig. 8 and Supplementary Fig. 19). The first pathway (TROP-ESA) utilized official Level-2 (L2) offline products obtained from the European Space Agency (ESA, https://disc.gsfc.nasa.gov/; accessed: 9 January 2025). Data were filtered using a highly stringent quality assurance (QA) threshold (QA > 0.75) for both NO_2 and HCHO, which provides a selection of observations that is considered optimal by effectively excluding retrievals compromised by heavy cloud cover or aerosol pollution. These filtered L2 orbital data were subsequently regridded onto a uniform 7 × 7 km spatial resolution for regional mapping. Alternatively, the second pathway (TROP-GEE) employed operational Level-3 (L3) offline products accessed via the Google Earth Engine (GEE) cloud computing platform (https://earthengine.google.com/). Within the GEE framework, the original time-binned L2 orbital swaths are converted into L3 spatial grids utilizing the harpconvert tool (via the bin_spatial operation), with antimeridian-crossing orbits systematically split to preserve spatial continuity. Quality filtering for this GEE-derived dataset is standardized, restricting observations to pixels where QA values exceed 0.75 for NO_2 and 0.50 for HCHO. Compared to the highly rigorous filtering applied in the TROP-ESA method, the TROP-GEE approach offers a streamlined and reproducible pipeline; however, we note that the GEE data record is available from 2019 onwards. A side-by-side comparison of the results derived from both data processing pathways is presented in Supplementary Fig. 19. It can be seen that although the absolute FNR values across the three megacity clusters differ slightly from 2019 to 2024 due to divergent filtering criteria, they exhibit concordant interannual variations. This alignment demonstrates that the relative FNR

trend—rather than its static absolute value—provides a more robust indicator for diagnosing O_3 sensitivity.”

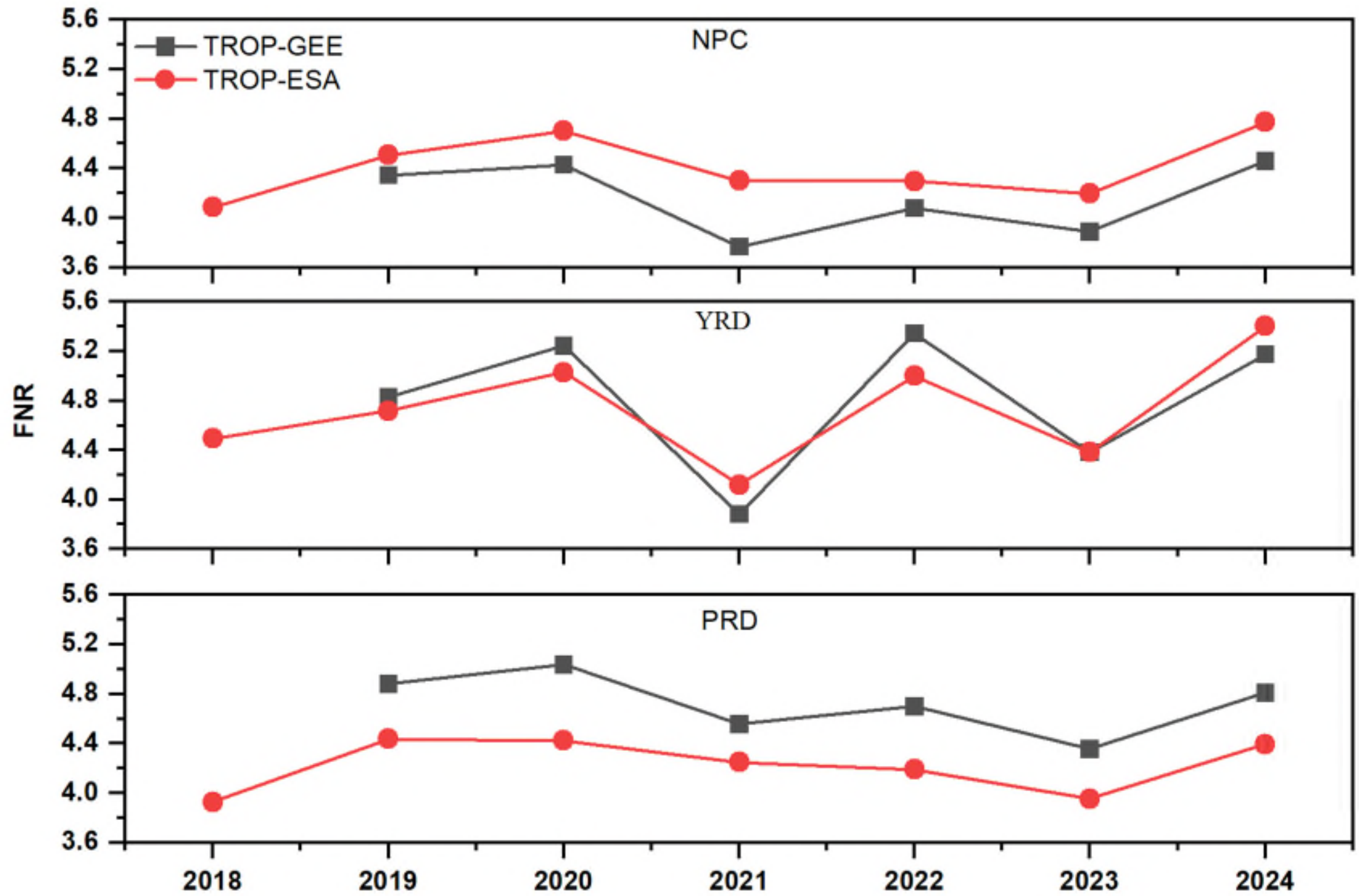

Supplementary Fig. 19. Interannual variation of summer TROPOMI satellite-based FNR ($HCHO/NO_2$) derived from two process pathway in the three megacity clusters of China during 2018-2024. The TROPOMI data from GEE platform start from 2019.

Supplementary Note 3：The robustness of sensitivity trends investigation.

“Previous studies have demonstrated that the trend in FNR is less sensitive to the choice of satellite product, as long-term multi-year averaging attenuates inter-product differences, and different products generally exhibit high consistency in their long-term spatiotemporal variations[3,23]. The robustness of the FNR trends in this study is corroborated by two key lines of evidence: (1) the consistent month-to-month evolution of column FNR between TROPOMI observations and air quality model simulations across representative cities in the summer of 2020 (Supplementary Fig. 8); and (2) the consistent interannual variations in TROPOMI FNR derived via two independent data processing pathways across the three megacity clusters during the

summers of 2019－2024 (Supplementary Fig. 19).”

We trust that these revisions, prompted by the reviewer’s highly constructive comments, have enhanced the scientific rigor and methodological transparency of our manuscript.

Reviewer #3 (Remarks to the Author):

Comment: In Aligning climate and air quality goals demands early VOC control for China’s ozone pollution, the authors present an interesting study which reveals that O_3 trends in China are starting to plateau, as well as highlighting the opportunity for a short “VOC window” in which reducing VOCs would be most effective.

Whilst reducing VOCs now to reduce O_3 most effectively is not a new idea, this study brings together multiple different techniques from the literature, to more fully describe the chemical regimes leading to O_3 formation in China. A more robust way of bringing together all these techniques, such as the one presented here, is of great interest to the air quality community since using one technique alone can often lead to conflicting or inconsistent results. This study is ambitious. As well as combining multiple analysis approaches, it isn’t observation-site-specific, covering multiple megacity clusters across China. Whilst individual techniques employed are not particularly novel, the way they have been combined, coupled with spatial coverage of the study, make for a very interesting manuscript to the air quality community.

The authors have made comprehensive changes to the manuscript in response to my previous reviewer comments, and there is now much more detail in the description of the methodology. I am satisfied that the work presented here is detailed enough for the work to be reproduced and would like to thank the authors for their detailed response and subsequent revisions, leading to a greatly improved manuscript.

Minor correction:

The authors describe policy-driven emission changes across China as a “natural experiment”, a term often used to describe phenomena such as the COVID-19 pandemic. I don’t think this terminology is quite correct in the

context of this study and suggest that this is simply removed.

Reply: We thank the reviewer for the positive assessment of our work and for this thoughtful suggestion. We have removed this term from the manuscript.

Reviewer #4 (Remarks to the Author):

Comment: The authors have adequately addressed the comments made by the reviewers in the revised version of the manuscript. Therefore, I have no further comments.

Reply: We thank the reviewer for the endorsement and for their previous feedback, which helped strengthen the manuscript.

Reviewer #5 (Remarks to the Author):

Comment: I co-reviewed this manuscript with one of the reviewers who provided the listed reports. This is part of the Nature Communications initiative to facilitate training in peer review and to provide appropriate recognition for Early Career Researchers who co-review manuscripts.

Reply: We thank the reviewer and the co-reviewer for their valuable contribution to the peer-review process and for their positive evaluation of our work.

A point-by-point response to the reviewers' comments

Response to Reviewer #2

Reviewer #2's Final Comment:
***The authors have adequately addressed my previous comments. I do not completely agree with their interpretation of the study results, but the updated manuscript now contains sufficient information for a knowledgeable reader to draw their own conclusions. I have no further comments and support publication in the current form*.**

Reply:
We sincerely thank the Reviewer #2 for the ultimate support of the publication of this manuscript, and more importantly, for the exceptional academic professionalism and broad perspective demonstrated throughout the review process.

We thank the reviewer for this constructive perspective and for acknowledging that the revised manuscript provides sufficient information for the scientific community to draw informed conclusions. We would like to take this opportunity to explicitly acknowledge that the rigorous and in-depth inquiries raised by Reviewer #2 during the previous rounds of revision were instrumental in elevating the quality of this work. Most notably, the constructive challenges directly prompted us to deeply explore and successfully quantify the duration of the "AVOCs reduction bonus window." This quantification has become one of the most robust and strategically significant improvements in our manuscript.

We are deeply grateful for the time, expertise, and rigorous scientific spirit the reviewer has dedicated to our work.

This study offers a fresh and highly significant perspective on controlling ozone (O_3) pollution in China, with both crucial scientific and practical implications. By introducing the Coupled Dynamic Trend Response (CDTR) analytical method, the authors successfully overcome the limitations of traditional static sensitivity analysis. I believe this manuscript is innovative, its findings are highly significant, and it is well-suited for publication in ***Nature Communications***. However, to further strengthen its scientific rigor and persuasive power, I have several technical questions and suggestions for the authors to consider during the revision process.

Specific Questions

1. What do the shaded areas in Fig. 1b represent? Please clearly label this in the figure caption or legend.
2. The main text mentions using TROPOMI data instead of OMI data. Please explain the rationale behind this choice. If OMI data were used as the FNR indicator, given its longer historical record, how would the trend change? Please provide a supplementary analysis or explanation.
3. How were the inflection points in Fig. 1c identified, particularly the start of the plateau phase around 2020? Please provide a detailed description of the quantitative method used, such as time series analysis or a specific change point detection algorithm.
4. Figure 2 is used to cross-validate the satellite data. Why were the years 2019, 2020, and 2021 specifically chosen for this validation, especially since Fig. 1 shows the plateau period extending beyond these years?
5. In Fig. 2b, four cities in the NCP are used for corroboration, but Zhengzhou is not described. Furthermore, only one city in the YRD (Shanghai) and no cities in the PRD are used. To enhance the generalizability of your conclusions, please explain the selection process for these cities and why some regions have fewer data points, such as due to data availability.
6. Why was OMI data not used in Fig. 3? Using OMI, which has a longer time series (e.g., from 2005 onwards), would align better with the MEIC emission inventory data (2005–2020) shown in Fig. 3a, providing a more complete historical trend analysis.
7. The WRF-CAMx model's performance was evaluated using only the year 2020. What is the model's generalization ability for other years? Please provide evidence that the model is reliable for long-term simulations spanning from 2020 to 2060.
8. In Supplementary Text, on line 108, please correct "UT+8" to the standard "UTC+8."
9. In Supplementary Table 9, there are two "R" values. Please clarify in the table caption what each R value represents, for example, one for the training set and one for the testing set. The significant difference between these two R values suggests a potential overfitting issue. Please address this and discuss its potential impact on the robustness of your model's conclusions.
